# “We all have a responsibility”: a narrative discourse analysis of an information campaign targeting help-seeking in first episode psychosis

**DOI:** 10.1186/s13033-019-0289-4

**Published:** 2019-05-09

**Authors:** Hege Hansen, Signe Hjelen Stige, Christian Moltu, Jan Olav Johannessen, Inge Joa, Sveinung Dybvig, Marius Veseth

**Affiliations:** 1 Department of Welfare and Participation, Western Norway University of Applied Sciences, Postbox 7030, 5020 Bergen, Norway; 2 Department of Clinical Psychology, University of Bergen, Bergen, Norway; 3 Department of Psychiatry, District General Hospital of Førde, Førde, Norway; 4 TIPS-Network for Clinical Research in Psychosis, Stavanger University Hospital, Stavanger, Norway; 5 Network for Medical Sciences, Faculty of Health, University of Stavanger, Stavanger, Norway

**Keywords:** First-episode psychosis, Psychosis, Help-seeking, Early detection, Early intervention, Information campaigns, Discourse, Social responsibility

## Abstract

**Background:**

Intervening at an early stage of psychosis improves the chances of recovery from first-episode psychosis. However, people who are experiencing distress and early psychotic symptoms generally seem to delay seeking help. Therefore, multifaced information campaigns targeting help-seeking behavior of potential patients and their network are considered important tools within early detection and intervention strategies. In this study, we aimed to explore which discursive meaning content, including roles and actors, such information campaigns build on and construct. Our intention was not to provide objective answers, but to contribute to a discursive debate about potential conflicts in messages conveyed in such campaigns.

**Methods:**

A broad sample of information material utilized by TIPS Stavanger University Hospital (Norway) was examined. The material consisted of posters, booklets and brochures, newspaper ads, Facebook ads, and TIPS Info’s website, representing various campaigns from 1996 to April 2018. A narrative discursive approach was applied at an epistemological level. At a practical level, a team-based thematic analysis was utilized to identify patterns across data.

**Results:**

Diversity and several changes in strategy were recognized throughout the information material. Furthermore, three main themes and four subthemes were found to constitute the meaning content built in the information campaigns: knowledge is key; (almost) an illness among illnesses; and we all have a responsibility (comprising of the subthemes; to respond quickly; to step in; to provide an answer; and to tag along).

**Conclusion:**

Our findings pointed to common dilemmas in mental health services: How to combine professional expert knowledge with collaborative practices that emphasize shared decision-making and active roles on behalf of patients? How to combine a focus on symptoms and illness and simultaneously express the importance of addressing patients’ recourses? And how can we ask for societal responsibility in help-seeking when professionals are placed in expert positions which may not be optimal for dialogue with potential patients or their network? We discuss whether highlighting practices with more weight on resources and active roles for patients and their surroundings in information campaigns could promote earlier help-seeking.

**Electronic supplementary material:**

The online version of this article (10.1186/s13033-019-0289-4) contains supplementary material, which is available to authorized users.

## Background

Psychotic symptoms may include delusions, hallucinations (auditory, visual or tactile) and disorganized cognition [1]. Psychosis can be a single episode or recurring as part of a psychotic disorder, e.g. schizophrenia. First-episode psychosis often occurs in late adolescence or early adulthood and greatly influences the lives of those who are affected and their families. Long-term studies have documented considerably better prognoses for psychotic disorders than early research indicated [2]. Substantial research during the last decades also documents that intervening at an early stage of psychosis plays an important role in improving the chances of recovery [3–7]. In consequence, early detection and intervention strategies are now implemented in several local and some national mental health services in most parts of the world [8]. One important aim of these strategies is to reduce the ‘duration of untreated psychosis’ (DUP)—as well as shortening the length of the psychotic episode and preventing relapses [7, 9]. However, it is still considered a major problem that people generally seek help too late [10]. People who develop psychosis often go untreated for as long as 2 to 3 years before they get in contact with the mental health system [11].

Research has revealed that people may experience longer trajectories of subjective distress than what was emphasized in the focus on DUP [12]. A recent systematic review and meta-synthesis explored distinct sources of distress in the first-person accounts of 33 qualitative studies of first-episode psychosis. It emphasized the diverse and multifaceted nature of both interpersonal and intrapersonal strains in the lives of people experiencing psychosis [13]. Furthermore, qualitative studies have focused on how people experienced contact with early intervention services for psychosis. In a literature review and meta-synthesis of 17 qualitative studies, five broad themes described the process of meeting with and going through such services: something is wrong; do for myself, it’s about people; a price to pay; and ongoing vulnerability [14]. Similarly, Tindall, Simmons, Allott and Hamilton [15] explored help-seeking processes and initial engagement and found five key topics based on a summary and analysis of nine interview studies: experiences of finding help; factors promoting engagement; the therapeutic relationship; the role of caregivers in supporting engagement; and factors impacting ongoing engagement. Moreover, a qualitative sub-study of the ‘Early Treatment and Intervention in Psychosis Study’ (TIPS-2), focusing on obstacles to care in first-episode psychosis patients with long DUP, identified five themes: participants’ failure to recognize symptoms of psychosis; difficulties expressing their experiences; concerns about stigma; poor psychosis detection skills among health-care professionals; and participants’ lack of awareness or understanding of information campaigns [16].

One explanation for the treatment delay may be that early signs of psychosis, such as social withdrawal, anxiety, sleep difficulties, concentration and memory problems, partly overlap with natural challenges in youth and symptoms of other mental health problems [17]. There is also considerable stigma associated with psychosis and mental health services which impacts help-seeking behavior [18, 19]. Additionally, public knowledge (mental health literacy) and beliefs about psychosis and specific symptoms are regarded as lacking or inadequate [20, 21]. Information campaigns therefore represent a central part of early detection and intervention strategies and are considered an important tool to alter help-seeking behavior [22, 23]. In a systematic review and meta-analysis, Lloyd-Evans et al. [24] found promising evidence for interventions applying intensive, multi-targeted campaigns—combined with easy accessible early detection (ED) teams. The TIPS study, the Scandinavian early intervention in psychosis, demonstrated that it was possible to reduce DUP significantly. Multi-targeted information campaigns played a significant role in this work [7, 9]. Hence, we do have some knowledge about how young adults and their families experience the period before and during contact with ED-services. Research also indicates little public knowledge about psychotic symptoms, and that multi-targeted information campaigns are the most effective way to provide information directed at help-seeking behavior [20, 21, 24]. However, we lack knowledge about the messages that are conveyed to potential patients and their surroundings in such multi-targeted campaigns.

At the same time, there are clearly challenges related to the implementation of ED and information campaigns. For example, psychosis is not a straightforward concept. In fact, scientists do not yet fully understand the nature of this condition. It is still debated what role different factors such as biological, psychological and social aspects play [25]. As a consequence, summarizing our knowledge to the general public through information campaigns is a complex enterprise as there is no real consensus on what psychosis is or what causes it [26]. In addition, multi-targeted information campaigns communicate slightly different messages to several audiences simultaneously—the person who may be affected, family, friends, teachers, health- and social workers, as well as the public in general—adding multiple facets to this challenge. Given the central role information campaigns play in early detection and intervention efforts and considering the demanding landscape they have to maneuver, we aimed to research which meaning content is built into such multi-targeted campaigns. Further, we aimed to explore what actors and roles information campaigns build on and construct.

## Methods

To explore the research questions we examined all the information material utilized by TIPS Stavanger University Hospital (Norway) from 1996 to April 2018. TIPS Stavanger is particularly interesting as their program is one of the most comprehensive interventions internationally, providing a broad and diverse spectrum of material to select from. They have also documented significant reduction in DUP, and explicitly and systematically used information campaigns in this work [7, 9, 22, 23].

The present article is a composite of interrelated methodological and theoretical approaches. On an epistemological level, we used a discursive [27–29] and narrative-inspired [30–32] perspective to explore the meaning content of the information material. A discursive approach involves studying the action-oriented nature of language [27, 29]. Through a focus on the functional aspects of language, reality is regarded as constructed, and at the same time constructive, as language builds various versions of the world, actions, and events [29, 33–35]. A narrative approach rests on the premise that people live and/or understand their lives in storied forms, meaning that events are connected together as a plot and played out in a context, e.g. in a particular society, culture and period of time [34, 36]. We used the discursive-language element to examine and discuss how messages built in language (and pictures) seemed to construct certain roles and actors, and how these constructs aimed at eliciting certain functions. The narrative element provided us with a perspective on how various messages or language elements could be integrated into more coherent stories or narratives told in the data material.

We chose thematic analysis [37] as the practical tool for analysis. This is a flexible method that seeks to identify, describe and analyze patterns across data and may be applied across various theoretical and epistemological qualitative approaches [37]. We used a team-based approach to this analysis [38] to emphasize reflexivity [39, 40]. This means we noted our own presence in the study and how our positions influenced the research processes and outcome.

### Settings for the present study

TIPS in Stavanger, Rogaland (Norway) started their work in 1996 and was initially designed to test whether early timing of treatment could improve trajectories in first-episode psychosis [7]. The ED part of the program is made up of two main elements: broad and multi-targeted information campaigns directed at the general public, schools and professional health personnel, in combination with accessible early detection teams located in the specialized mental health care system [7, 23]. The program aims to change the help-seeking behavior of the population in the designated catchment area [10, 22, 23]. Second, early treatment is offered as a standardized 2-year standard protocol, which includes anti-psychotic medication, supportive psychotherapy and multi-family psychoeducation [7, 41]. This combination of using multi-targeted campaigns and ED-teams was groundbreaking when the intervention was first developed and implemented.

### Data material and data collection

The data material consisted of all newspaper and Facebook ads, TIPS Info’s website (http://www.tips-info.com), posters, booklets and brochures (including one brochure directed at mental health workers) developed and used by TIPS from when they started their work in 1996 up until April 2018. The data was collected by the first author (H.H.) in collaboration with TIPS, Stavanger University Hospital. First, we arranged a meeting between J.O.J. and S.D. from TIPS Stavanger and H.H. to get an overview of the potential data material. TIPS provided both physical ads and posters, in addition to a data file containing relevant information material from their campaigns. Second, H.H. collected digital data through TIPS Info’s Facebook site (available from 2009 to 2018) and website. The complete data material was then checked by three of the authors, H.H., S.H.S., and M.V., for the following inclusion criteria: The data material had to (a) be directed at early detection/intervention in relation to first-episode psychosis (text and/or picture), (b) be marked with TIPS Stavanger’s logo and (c) be TIPS Stavanger’s (original) material. To avoid considerable incongruity in the material, we excluded longer texts, such as newspaper articles and radio- and video clips from TIPS Stavanger. Links to other Facebook pages (non-TIPS-material), courses, conferences or other institutions’ information posted on TIPS’ Facebook page were also excluded. The included data material was thereafter sent to J.O.J. and S.D. to assess whether it reflected the diversity and nuances in TIPS’ information campaigns. The final data material consisted of a total of 149 pages (collected between January and April 2018) representing various campaigns from 1996 to 2018.

### Researchers

H.H., S.H.S., C.M., and M.V. were a part of the analytical team, and H.H., J.O.J. and S.D collected the data. I.J. related the study to the literature on early intervention. H.H. is a social worker and research fellow at Western Norway University of Applied Sciences and principle investigator of this study. S.H.S., C.M., and M.V. are clinical psychologists. S.H.S. and M.V. are associate professors at the University of Bergen, whilst C.M. is a professor at Western Norway University of Applied Sciences. J.O.J. is a psychiatrist and professor, and I.J. is a nurse and associate professor, both working at TIPS, Stavanger University Hospital and University of Stavanger. S.D. is a communication advisor at TIPS, Stavanger University Hospital. The researchers from TIPS have contributed to developing the TIPS intervention and information campaigns, and provided scientific oversight in the present study. All authors have commented on the paper and contributed to the final concept development.

### Data analysis

The analysis was performed by the four researchers (outsiders to the data material) in the analytical team (H.H., S.H.S., C.M., and M.V.). Six steps from Braun & Clarke [37] were applied to guide the analysis:

1. To become *familiarized* with the data, the material was read and re-read. Preliminary features relevant to our research questions were also considered. In addition, we discussed how the analysts’ positions as socially oriented researchers would influence what we were looking for, as well as the directions of our interpretations [38, 40].
2. *Codes* identifying semantic and/or latent content were initially noted by the individual researcher in the analytical team. We used an open-ended and explorative approach [38, 40] to catch different and nuanced messages in the data—and identify the actors involved (e.g. mental health professionals, patients, family, etc.). Text and associated pictures were viewed as a whole and we focused on the way they communicated together as a discursive resource [34].
3. In an analytic-meeting, the team searched for *preliminary themes* by sorting the different codes into potential themes. Notes and tape recordings were taken during this interpretive process. A flip-over was used to record tentative themes.
4. The preliminary themes were *reviewed* by going back and forth between the data material and the themes to assure they were grounded in the data—and to extract relevant quotations and pictures to underline the content within each theme [38].
5. The themes were *defined* through the writing of a ‘story’ for each theme, and considering how it fit into a broader ‘story’ of the data. They were *refined* through examining possible requirements for additional subthemes. As an integral step of the analytic process, the result of the analysis was sent to J.O.J., I.J. and S.D., who read and commented on it. This provided important information about the context of the present study and also held an important reflexive function in making us aware of different positions that may follow insider and outsider perspectives.
6. H.H. wrote a *report* in collaboration with the other researchers from the analytical team. The report was written to ensure sufficient evidence of the themes within the data, but also by going beyond descriptions to provide an analytic narrative which illustrated the story we (the outsiders) were telling about the data. The outcome of our analysis was three main themes and four subthemes which we saw as central meaning patterns in the information material. Yet, in discursive and interpretative approaches, many additional questions can be asked. Analysis is therefore never regarded as exhaustive [34].

## Results

In analyzing the data, the analytical team recognized diversity in target groups throughout the material. The ads and brochures were directed toward the general public, as well as more strategic target-groups, i.e. students, teachers, GPs, health- and social workers, etc. In addition, several changes in strategies were identified. We describe this diversity before we turn to the three themes found to constitute the meaning content: (1) *Knowledge is key*, (2) *(Almost) an illness among illnesses* and (3) *We all have a responsibility*.

### Differentiated target group communications and changes over time

In the early phase of the information campaign (1996–1997), the information material was distributed to all households in the county of Rogaland. At the same time, a range of more demarcated target groups were addressed. This included at risk youth, persons who had already developed early signs of severe mental illness, as well as people who were likely to interact with them: friends/peers, parents, school counsellors/teachers, health- and social workers etc. The ads often had slightly different content depending on who the recipient was meant to be. An example of this is a quote from an ad directed at teachers: “It isn’t easy being a teacher when a student changes and you don’t know why” (1999). Another example is found in an ad directed at social workers: “Everyone who works at the social office will now and then meet clients with mental health problems” (1999).

The early ads (1996–1999) were directed both at people in general as well as people surrounding those who were vulnerable. More often than not, parents, or mothers in particular, appeared to be the most important target group. For example, in one of the ads the heading said, “He or she who meets them [the problems] face on will most easily put them behind them” (1998). The text in the ad continued: “Rarely will the person who is struggling with mental health problems be able to contact a doctor. Most of the time it is relatives, friends or coworkers who have to do it” and “You can get help in order to help”.

Another variation we discovered in the data material was that in the beginning (1996–1997) ads started out with an open invitation to contact the early detection team independent of their degree of concern. In time, the ads communicated a slightly more restrictive service—meaning that people should contact them according to the supposed degree of risk. For example, in several Facebook ads (2017) as well as the website (2018) three levels of danger were utilized: green, yellow and red—representing the severity of signs/symptoms. In addition, the later ads (approximately from 2009 to April 2018) also seemed less authoritative than the early ones, as they often used a more suggestive tone, e.g. “If you’re worried about yourself or someone else you can call TIPS” (2017).

Moreover, we found diversity in the way the concepts *mental illness*, *serious mental illness* and *psychosis* were utilized. In many ads, *mental illness* was used in the heading followed by a description of psychosis in the text. In some ads, both *mental illness* and *serious mental illness* were applied, while in others the term *psychosis* was used alone.

Three core categories, or themes, emerged when analyzing this diverse and dynamically changing pool of data for thematic content across ads, across time periods and across target groups. Here, a theme is understood as a basic pattern of communication across contexts. In the following, we present and detail the thematic structure that resulted from the outsiders’ analyses.

### Themes

#### Knowledge is key

Throughout the information material, knowledge was presented as key to increasing public awareness and altering help-seeking behavior. It was communicated that the content provided in the information material may assist people in understanding what is going on. For example, one ad said: “Read and become wiser…” (1997). As such, knowledge was presented as something that professionals within the mental health services hold. This is also illustrated through a quotation from one of the ads: “Experienced professionals will tell you what you should do, how you can move forward to figure out the situation and how you can get the right treatment or assessment if that’s necessary” (1999). This knowledge was often depicted as specific and objective. An example from one brochure pointed out: “Good treatment is based on solid knowledge and not just viewpoints and ideological beliefs” (1999). In one of the brochures (1999), the right treatment was described as consisting of mainly three parts: consulting a doctor, psychiatrist or psychologist (psychotherapy), combined with family-oriented therapy and medical treatment. At the same time, it was underlined that one type of treatment does not fit all. Hence, treatment has to be individually tailored.

Furthermore, it is not sufficient that the mental health system holds knowledge. One of the main messages in the information material was that people who meet persons at risk of developing psychosis should know what to look for. Through the information provided, people were told to attain a position in which they are able to help others, illustrated by the following quotation from one of the ads: “The more you know about them [mental illnesses] the easier it is to help the one affected” (1997). It was communicated that the reason why people do not act, is that they lack or do not have the right knowledge. In consequence, a core challenge is to get the information out to the public. An ad stated that “TIPS has a goal of increasing knowledge about mental illnesses so that more people contact health care services earlier and get treatment before the patient develops a serious psychosis” (1999).

Symptoms or signs of psychosis were presented as information that should be an essential part of common knowledge. Almost every ad in the data material listed signs to be aware of. They were presented as early signs of psychosis, serious mental illness (particularly the early ads) and mental health problems, or simply listed without any label. People were told to be alert if a person withdrew from his or her family, friends and colleagues, or if he or she isolated him- or herself. Other signs to look out for were if he or she slept poorly and ate little, stopped taking care of themselves, spoke or wrote about meaningless stuff or expressed inappropriate emotional reactions (e.g. laughing when hearing about sad news). Furthermore, reasons to be on guard included if the person became expressionless or did not react at all, felt persecuted, controlled by voices from outside or believed they had magical capabilities.

Symptoms and diagnoses were also presented on Facebook through a Christmas calendar (2016–2017). Each day, knowledge about one mental health issue was presented, starting with milder symptoms at day one, and gradually increasing the level of severity in the signs presented, e.g. day nine presented hallucinations. An excerpt from the accompanying text stated: “Hallucinations are perceptions which aren’t caused by outer sensations”. The calendar ends with psychosis on day 22—followed by early intervention and prognosis on day 23 and 24. The calendar can be interpreted as a gift to the public, comprising of knowledge as well as hope portrayed through the possibility of improving the prognosis through early intervention.

In the early ads (1996–1997), knowledge was also seen as key to breaking down old myths about mental health services. References to (and pictures from) the movie “One Flew Over the Cuckoo’s Nest” were, for example, used to illustrate the stigma and myths surrounding mental health institutions. One of the ads expressed: “A lot has happened in treatment of mental illness the last decades, but old myths still endure” (1996) (Additional file 1: Figure S1).

#### (Almost) an illness among illnesses

Many ads communicated that severe mental illnesses had a lot in common with physical illnesses. In this sense, it was communicated that psychosis has to be understood and treated in similar ways to physical illnesses. Several ads explicitly expressed: “Mental illnesses are like other illnesses…” (1996–1997). Occasionally, ads shed light on diagnostic labels, for example “bipolar disorder” or “schizophrenia” (1996–1997). Furthermore, in one of the ads a metaphor of a particular type of cancer, melanoma, was used. This is a type of cancer which the public knew little about and, consequently, many died from. After applying campaigns that raised public awareness of what to look for, experts were able to save many lives because people now sought treatment at an earlier stage. The same rationale seemed to be applied to psychosis, with the exception that the latter is said to be even more difficult to detect. As such, public awareness needs to be raised so that early intervention can be successful.

In the same way as with cancer/melanoma, psychosis was seen as developing in stages. One brochure said, “We see the psychosis as a process, where the psychotic breakthrough or breakdown is a stage of the illness’ development” (1999). As such, psychosis appears to be understood as developing from an early sense of anxiety, with a risk of converting to psychosis if not detected and treated at an early stage. Timely intervention can, however, impede this development. For instance, one ad expressed that: “Psychotic episodes can be prevented” (2015). In addition, psychosis was in part described as a breakdown in rationality and meaning, e.g. one of the signs of psychosis listed in the ads was to “talks and writes about meaningless things” (1998).

However, some ads had a slightly different twist. Instead of comparing mental and physical illness, overlap between these was highlighted at just one point—the need for early intervention. For instance, one ad showed a picture of a bandaged thumb pointing upwards, followed by the heading: “At one point, mental illnesses are just like other illnesses—when the help is provided early there is a greater chance of getting healthy” (1997) (Additional file 2: Figure S2).

Simultaneously, psychosis was also defined as something different from physical illnesses. For instance, one ad communicated how psychosis or mental illnesses are valued differently than physical illness: “Mental illnesses don’t have the same « status » [as physical illnesses]” (2000–2001), and the same ad stated: “Still, they’re hard to talk about” (2000–2001), which seems to point to the stronger stigma related to mental illnesses.

In addition, it was communicated that psychosis was seen as something other than physical illness, as there is a fine line between psychosis and problematic, yet normal behavior or feelings, e.g. a quote from one ad said: “That teenagers change is natural. When teenagers change noticeably over a short period of time- parents often worry. If teenagers isolate themselves, are silent or seem depressed, it is natural to be on guard” (2012). As such, natural emotions and behavior were expressed as something worrisome if they lasted over a period of time. Accordingly, signs of illness may also be signs of normality, as mental illness is more complicated to uncover than many physical illnesses.

#### We all have a responsibility

A vital message throughout the information material was the importance of reducing the duration of untreated psychosis. Many of the ads emphasized that treatment is often delayed. This was pointed out in the following quotation from one of the ads: “One of the biggest problems in treatment of serious mental illnesses- psychoses-is that the patients come to treatment too late” (1997). As a consequence, we all have a responsibility to detect people with, or at high risk of developing, psychosis, and help them get in contact with mental health services. By doing so, they can receive the treatment they need to get well. In the material, this task was not communicated as a responsibility only for mental health services. Psychosis, or mental illness, was presented as something that concerns everyone. In one of the brochures it said, “All Norwegians know someone with mental health problems” and “Every 15th Norwegian has a serious mental illness” (1998). This underlined a personal as well as a societal responsibility. Both caring for the people who are affected, as well as saving costs for society were presented as justification for the broad approach and its urgency claims. In this line of communication, mental health is not a private issue, it affects us all—mothers, fathers, friends and health workers. Everyone has to play a role to help prevent and alleviate mental health suffering, albeit with different role instructions. The societal responsibility was demonstrated through the next two quotations from one of the ads: “If the sick get help earlier there is a greater chance of getting healthy and they need less time at the hospital” (1998) and “It saves the society money and the affected will have a better life” (1998).

In our analysis, we developed four subthemes to summarize how we have a shared responsibility (a) to respond quickly; (b) to step in; (c) to provide an answer and (d) to tag along.

##### a. To respond quickly. Responsible: All

It was held forth as a fundamental problem that those it may concern rarely contact the mental health services themselves, e.g. as expressed in the following quotation from one ad: “The person affected is rarely able to contact a doctor themselves. That’s why it’s often someone close to the person who has to do it. That can feel hard and difficult, but it’s important to act quickly” (1998). A perpetuating message was conveyed saying that one should contact early detection services promptly if symptoms or signs of psychosis are observed. For instance, several ads used the sentence: “Seek help as soon as possible, that’s when you have the greatest chance of getting healthy” (1996–1997). The call for instant action underscored that it is possible to prevent, relieve or delay the onset of serious mental disorders, and that early treatment will be more effective.

In some ads, psychosis was presented as something that evolves gradually and almost unnoticeable. In a sense, it creeps up on you. As a consequence, the ads tell people to be on guard, and that there is a need for constant vigilance. What might seem like normal behavior does not have to be. This is articulated in one of the ads: “Serious mental illnesses often begin innocently. The person affected acts a little strange, but that can be temporary. It’s the recurrence which signals danger” (1996–1997).

Another way of communicating the need to respond quickly is to stress the negative consequences of delayed action. Such effects were illustrated in several ads using metaphors from children’s songs/plays expressing the poor outcomes of late responses, e.g. “Snip, snap, snout, this tale’s told out” (2000). By using single lines from children’s songs literally, and thus juxtaposing mental images of innocent children playing and severe mental health suffering, the ads employed poignant rhetorical strategies toward immediate action. Another example of possible negative consequences if people do not respond quickly can be found in an ad presenting a picture of domino tiles that have begun falling. Once the domino tiles have started to topple over, one thing leads to another in a downward spiral. Both images stressed the need for early detection and treatment, as well as the need for public awareness and response (Additional file 3: Figure S3).

##### b. To step in. Responsible: all

This is closely related to the previous subtheme. However, while the former refers to the need for understanding what one observes, being vigilant regarding signs of mental illness and swift action when such signs are observed, this subtheme assigns the responsibility to act even in ways you would not normally do in regards to another person’s health. This seemed to indicate that some young adults being at risk or suffering from psychosis are hesitant to contact services and sometimes even refuse, thus others need to cross boundaries they otherwise would not to step in. For example, one of the ads stated: “That’s when it’s important that others take responsibility and seek help” (2000). A central message in the ads is that people have a moral obligation as citizens to step in. This is illustrated through the following quotation from one of the ads: “If you know somebody who is affected by mental health problems, do as you would with other illnesses, contact a doctor, psychologist or the emergency room (ER) if you need advice” (1996–1997).

In many ads, this responsibility is more directly placed on people’s close relatives. For example, one ad stated: “This year many will worry about friends and family developing mental health problems… make sure the worry passes as soon as possible (2000). In this way, the commitment to take action is placed on the reader. As such, the messages conveyed through this strategy seem to suggest that one should go further than one ordinarily would with a young adult. One possible implication of this strategy is that many with mental illnesses are unable to take proper care of themselves, needing others to step in and make good choices for them. The legitimization for this breach of autonomy boundaries seems related to the images presented with regard to the roles of the suffering person and the responsible other.

Attention seems to be given to the importance of early intervention and that people around should step in, while the person’s own preferences at this stage are secondary. To lower the limit for contacting the services anonymous calls was presented as an option, e.g. from one of the ads: “You can get help in order to help… call TIPS, feel free to do it anonymously…” (1998) (Additional file 4: Figure S4).

##### c. To provide an answer. Responsible: the mental health professionals

Most of the ads described the ED team as a resource to the problems concerning youth at risk of developing a severe mental illness, as such the services readiness to help and to provide proper treatment and/or advice was highlighted. This was illustrated through the following quotation found in one of the brochures: “Everyone has specialized education/training and knows what should be done when a mental illness is developing” (1999). In the same way as with physical illness, mental health professionals seem to be regarded as experts who can provide knowledge. E.g. from one of the brochures: “We have created a brochure which talks about mental illnesses, what it is and how they are treated” (1999). This expert position was particularly emphasized in the early ads (1996–1999), for example it was said that: “… we will give a true picture of today’s options”, or in one of the brochures: “The patients get help quickly and are offered the help that is considered the best” (1999). Moreover, it was expressed that mental health issues can be severe and that people should take them seriously. One of the ads underscored that several hundred people have already called and received help and advice (1998). Accordingly, it was communicated that the early detection and intervention services are successful in their mission and that people can count on them. In some ads and brochures, photos and professional titles seemed to serve as a way of putting a face to mental health workers (1996–1999) (Additional file 5: Figure S5).

##### d. To tag along. Responsible: patients

An indirect message expressed in the ads was that people with, or at risk of developing, psychosis should seek help and follow professional advice. It was underlined that if someone feels uneasy or confused, they should contact health professionals. In one ad they say: “When you feel like something is wrong, you go to the doctor” (1996–1997). As such, their own interpretations or meaning-making capabilities were toned down, especially in the early ads (1996–1999). The main responsibility of the patient is therefore to go along with the treatment offered by professionals when in crisis and extreme distress, in order to get well and be able to continue on with their lives (Additional file 6: Figure S6).

The mental health professionals are not able to provide appropriate help unless patients share detailed information about their problems, as expressed in one ad: “We need your help to get where we want” (1996–1997). If potential patients (or others around them) do not contact the services and tell them about their afflictions, there is, in fact, nothing the mental health services can do. In this way, responsibility was also given to the patient.

In some of the later ads, home visits were proposed as an option. Here, mental health services are stepping out of their territory and into the context of people’s everyday life. Moreover, the language seemed less authoritative as the importance of following the professionals’ advice is toned down. A quotation from one of the Facebook ads illustrates this: “If you experience that challenges are larger than what can be solved in a confidential conversation you can ask us for help” (2017).

### Insiders’ reflections on the findings

A tentative report of the outsiders’ findings was sent to J.O.J., I.J. and S.D. from TIPS to obtain the insiders´ perspectives and viewpoints on the findings. This provided us with an opportunity to utilize elements of discourse while maintaining multiple perspectives. Involving the researchers from TIPS in this process, helped clarify the intentions behind the data material, and it became clear that the outsiders’ interpretation of the material at times diverged from TIPS’ purpose and plan.

For example, the outsiders interpreted the message that psychosis is an illness in line with physical illnesses. However, the insiders wanted to underscore the same need for early intervention in mental illnesses as in physical illnesses. The insiders stressed that they did not have a “strict/traditional bio-medical” understanding, but rather a dimensional understanding of psychosis (a process, developing in stages). They also pointed out that they wanted to tone down the significance of genes or heritage as important causal factors of psychosis.

Secondly, the insiders stressed the need to consider the context when understanding the information material. E.g. in relation to the subtheme “to respond quickly”, they underscored that the mental health system in 1996/1997, before TIPS initiated low threshold and easy access, had been less accessible to people. Therefore, it was vital to change people’s attitudes towards the system by pointing out that they were welcome to seek help—which was a totally new signal from the mental health services at that time. They further pointed out that the outsiders’ understanding about negative consequences (e.g. domino) of not responding promptly differed from the insiders’ conception of psychosis as gradually developing in stages—and intervention could hinder worsening at any stage—not just the first. It should also be noted that at that time, psychosis (schizophrenia) was seen as an illness without hope of recovery.

A third remark was related to the subtheme “To step in”. The insiders pointed out that sometimes people lacked insight into their own condition/situation, meaning it could be unethical not to act against someone’s will. Finally, the outsiders’ depiction of mental health professionals’ expert position in the subtheme “To provide an answer” was incongruent with the insiders’ aspiration to convey hope and readiness to help.

## Discussion and implications

In this study, we studied a broad sample of TIPS Stavanger’s information campaigns, ranging over a period of 22 years, to explore the meaning content, roles and actors built into information campaigns targeting help-seeking in first episode psychosis. Our analysis resulted in three main themes: (1) Knowledge is key, (2) (Almost) an illness among illnesses, and (3) We all have a responsibility, with the last theme comprising four subthemes: (a) to respond quickly (all), (b) to step in (all), to provide an answer (mental health professionals), and, to tag along (patients). Our findings and research process shed light on several conflicts within the data material, and the significance of perspective and context for understanding and interpretation. We believe these tensions represent common dilemmas in the broader field of mental health. On the one side, mental health professionals clearly hold important expert knowledge that needs to be put to use for the benefit of people suffering from psychosis. At the same time, the mental health system aims to develop collaborative practices that involve more egalitarian services [42]. How then, does one go along to combine practices based on dialogue, in which the patient is also involved in decisions about his or her treatment, when the use of professional knowledge, often involve evidence-based knowledge and standardized treatment? And, how could an integration of these two perspectives be better reflected in information campaigns to promote help-seeking?

### The expert position

ED services represent an important step forward when it comes to providing prompt care for people who experience first-episode psychosis [8]. ED services vary in content, but often they combine medical treatment with psychotherapy and family interventions, thus representing a distancing from the traditional ways of understanding mental health issues based on strictly medical approaches [43]. However, as shown in the theme “knowledge is key”, professional knowledge has played, and still plays, a central role within ED services. The strategy of distributing knowledge about mental health services and symptoms of serious mental illness to the general population has been documented as successful in influencing help-seeking behavior as TIPS reduced DUP from a median of 26 weeks to 4 weeks [7, 9].

However, the dilemma we face in providing this kind of information as part of ED efforts is that it rests on an underlying assumption that potentially contradicts the competing interest of user involvement and collaborative practice. If information is the answer, lack of information or adequate knowledge (mental health literacy) must be the problem. Adequate knowledge in this regard, seems to be understood as professional, evidence-based knowledge. As such, experience-based knowledge held by people with lived experiences of mental health issues is not presented as an important part. For information to be effective in altering help-seeking behavior, health care workers then must construct an expert position—as providers of answers/solutions through their professional knowledge (and as part of a mental health system). Although the expert positioning was toned down in the later ads, our findings show that primacy was given to focusing on signs and symptoms, based on professional knowledge, indicating the recipients’ need for specific action (help-seeking). This way of providing information may enable people to make more rational choices about their health. Yet, a dilemma still emerges between how to express professional knowledge from such a position and simultaneously convey a belief in the importance of people having faith that they will be able to handle their own lives.

Moreover, by utilizing such a strategy actors involved are assigned rather distinct roles. In discursive approaches, roles can be understood as constructed through language and social interaction [44]. We have focused on how the roles have been constructed through the language (including pictures) used in the information material, and how this can be understood in the context of mental health services. As shown in the subtheme “to provide an answer”, mental health professionals can be regarded as experts holding the answers. As pointed out in “Insiders reflection on the findings”, the researchers with an insider perspective did not regard themselves as experts, their primary intention had been to communicate their willingness to help and provide hope. The outsiders’ interpretation of the data, however, was that such a position was established by e.g. portraying mental health workers as being specially trained and competent to act appropriately when mental illness was about to develop. The outsiders understood potential patient, on the other hand, to be positioned in rather passive roles as followers of the professionals’ advice. Again, this diverged from the insiders aim to contribute to people’s active engagement in their own health behavior.

These positions may partly be understood in light of the traditional understanding within mental health services, that psychotic patients often lack insight into their illness [45]. Research indicates that denial of being mentally ill is particularly evident in patients with first-episode psychosis [46]. In a qualitative study involving professionals and patients, Solbjør, Rise, Westerlund and Steinsbekk [47] found that lack of insight was one of the factors that made participation difficult. This concept has, however, been critiqued for being limited by the validity of the diagnosis [45], which, in turn, can be regarded as an inaccurate measure and highly dependent on the clinicians subjective assessment [48, 49]. Furthermore, insight is not a straightforward concept as it means different things to different people. On one side of a continuum, the patient may be expected to accept having a mental illness, and on the other side, it is expected that the patient become aware that something has gone wrong in his or her life [50, 51]. With only one part possessing the power to define this the starting-point for dialogue and shared decisions making becomes compromised.

However, new role constellations which embrace both professional and lived experience knowledge are emerging within mental health services. Collaborative practices which emphasize shared decision-making and focus on individual’s goals and life circumstances have shown promising outcomes for engagement, particularly for groups such as young adults with first-episode psychosis [52]. Implementing such models involves working with competing beliefs, values, power balancing and relational competencies [42]. Applying methods based on co-productive principles in designing information campaign messages, could contribute to present potential patients in more active roles in future information campaigns.

### Making it clear, but not simplistic

Another dilemma following the provision of evidence-based knowledge about psychosis is how to express clear messages about the nature of psychosis when several different ways of understanding and explanatory models exist side by side. In fact, the understanding of psychosis ranges from psychosis as a chemical imbalance or brain disease to psychosocial explanation models, emphasizing the role of contextual factors such as stress, trauma, poverty, racism, sexism, etc. [53]. Accordingly, formulating clear, but not simplistic messages, about such a complex matter appears as a demanding task. As our findings showed in “(Almost) an illness like other illnesses”, the diversity of interpretations are also reflected in the information material. Different ways of looking at psychosis are likely to impact the role of the involved actors. The outsiders’ interpretation was that psychosis was partly portrayed as comparable to physical illness. This seems to communicate that psychosis is something that one should not be ashamed of, and in the same way as with physical illnesses—the victim is not to blame [54]. The effects on reducing stigma by stressing that psychosis is an illness like other illnesses have later been questioned [55, 56]. A literature review concluded that this strategy raised public fear as well as social distance towards people with psychosis [55]. However, there is some support that less stigma is perceived when applying descriptions of specific experiences, e.g. voice-hearing, as opposed to labeling diagnoses such as ‘schizophrenia’ [57]. The latter has been found to increase negative beliefs and attitudes in the general public [58], and research also indicates that diagnostic labelling negatively affects self-stigma [59].

Moreover, applying an illness-focus in information campaigns can be understood as placing the patient in a ‘sick’ role, which does not acquire much effort from the patient. Thus, the patient’s own actions are not regarded as an important part of treatment beyond seeking help. As pointed out under ‘Insiders reflection on the findings’, this understanding was not in line with what the insiders intended to convey. The insiders did not intend to base their description of psychosis on a medical understanding. Their intention had been to stress similarity to physical illnesses at just one point, namely—the same need for early interventions. However, the outsiders understood the illness-focus to be more pervasive. As a consequence of comparing psychosis with illness, passive roles risked being established on behalf of patients. As pointed out in the sub-theme “to tag along”, the patient’s main task then is to follow expert advice. This message does not match the knowledge we have of lived experiences of people with first-episode psychosis who were in contact with ED services which stressed the significance of self-efficacy [14].

In addition, our findings showed that psychosis can also be communicated as something dissimilar to physical illnesses. Psychosis was for example portrayed as a process that develops in stages. As such, the underlying understanding seems to be that psychosis is a result of several factors (biological, psychological and social)—the amount of stressors combined with the individual’s vulnerability (stress-vulnerability model) causes development of psychosis, or for some very few, a psychotic disorder [25]. By portraying psychosis as a process, the similarities with physical illnesses seem to be toned down (although some physical illnesses are understood to develop in similar ways)—and other aspects of psychosis were communicated. In this sense, psychosis may be viewed as an integrated part of the person, as opposed to some kind of ‘entity’ that just needs to be medicated away. Thus, people’s own meaning making processes and actions have a more central place in treatment as their faith is not only in the professionals’ [60].

This latter way to convey psychosis, points to more active roles for potential patients, which may be a more befitting way to present psychosis in information campaigns than focusing heavily on signs and symptoms. As such, there might be something to learn from approaches which put patients in more assertive roles, such as e.g. peer support. Peer support is generally described as a model for promoting wellness, focusing on strengths and positive aspects of people as opposed to an illness model focusing on symptoms and problems [61, 62]. Peer support is already one of the approaches utilized in early intervention services [63]. Although it was not very prominent in the information material, peer support is also included in TIPS’ intervention program. Within a mental health services context, peers may participate in mutual support groups where they can share common experiences (peer-to-peer support). Peers that have come further in their recovery processes may, however, be employed by the services (peer staff support) so that they can use their lived experiences to contribute positively by engaging people and serving as a bridge between patients and staff [62, 64–66]. Drawing more on knowledge-by-experience in information campaigns could provide a more nuanced picture of what psychosis is, and insight into important aspects to convey in information campaigns in order to reach the target groups. Additionally, elements of ‘peer staff support’ approaches might be utilized as ways of informing campaign designers of what people with lived experience of psychosis find to be helpful messages in this regard.

### We all have a responsibility

In the theme “we all have a responsibility”, the outsiders in the analytical team found that psychosis was portrayed as something that should not be considered an individual concern alone, thus, the societal responsibility was emphasized. The societal level is not normally a level in which the mental health services operate on, at least not related to treatment of psychosis. By putting psychosis on the agenda, as TIPS did through the multi-targeted information campaigns, the mental health services became a visible actor in the community. The broad strategy was based on studies of available literature at the time they started, and such multi-targeted and repetitive campaigns are still regarded as effective in altering behavior [67].

Moreover, the findings showed that people in general were encouraged to take action as delayed help-seeking is considered a serious problem in treatment for first-episode psychosis. For example, TIPS stated: “We need your help…”. This inviting tone represents something unusual coming from the mental health services, as these services have not traditionally been very assertive in asking for assistance from outsiders. People surrounding the young adults were told to be on alert and ready to step in when a close one or somebody else was in need of help. Hence, the primary roles of people around the one that struggles can be seen as helpers of the mental health system. As such, family members or school teachers, GPs or others who had concerns on behalf of a young adult were asked to step in and contact the services if needed. By applying such an action-focus, TIPS goes further than some of the other mental health information campaigns, e.g. “Depression: Let’s talk” [68]. In addition, TIPS has established an accessible ED-team who are ready to answer phone calls and offer help at short notice. However, the encouragement to step in and contact the services may raise ethical considerations for people around a person who struggles if the person does not agree. In this regard, the insiders pointed out that it may also be considered unethical not to take action if the person does not realize that he or she is in need of help. Moreover, meeting such an action-oriented service may also be perceived as what people surrounding young adults who struggle need [69]. However, the roles applied to people in general in TIPS’ information campaigns seemed—from the outsiders’ point of view, primarily limited to serving as helpers for the mental health system. In this sense, the constellation of constructed roles in the information campaigns seem to sustain the conventional roles within the mental health system and between the system and its surroundings. As such, information campaigns could be improved by conveying more explicitly the importance of actors outside the mental health system—not only as helpers of the system, but also as support systems for the potential patients.

More specifically, research shows that families play a vital role in help-seeking related to psychosis [70–72] Yet, studies also show that family members often attribute early psychotic symptoms to ‘normal’ adolescent behaviors [73], temperament, drug use, or physical illnesses [74, 75]. Similarly, stigma towards mental health services seems to partly explain delayed help-seeking [76–78]. A meta-synthesis of qualitative studies also reported that family members often exposed feelings of despair and fear towards mental health services, combined with a general feeling of hopelessness [79]. This implicates that information directed at a rational level might be insufficient. Thus, the potential of direct future information campaigns also toward emotional aspects should be explored. Additionally, to make information campaigns more appropriate to different target groups in the surroundings (e.g. parents, teachers, or social workers), piloting of how different messages are perceived by various target groups, could be a way to make the information campaigns more relevant for its different receivers.

### Methodological considerations

The present study draws on material developed within one program over a period of more than two decades. Thus, a strength is that it sheds light on shifting historical and social perspectives of how to present information for the public.

Also, as evident from the divergence in perspectives between the insider and outsider perspectives on the material, a narrative discourse analysis does not establish intentions. Rather, the promise of such methodology is to describe how meaning is created and transported between different actors and roles in a social system, as a basis for constructive discussions. We consider it a strength that we could include both perspectives in the presentation of the findings. The different positions as insiders and outsiders to the data material, provided us with proximity and distance that necessarily impacted the interpretations. However, it also became clear that the perceptions of insiders and outsiders were not always congruent. As such, we tried to make our different positions a transparent discursive element in the discussion.

Furthermore, these divergent perceptions underscore that the interpretations made by the analytical team were undoubtedly colored by the lenses of socially oriented researchers with particular interest in collaborative practice. By applying such an interpretative approach based on constructive epistemology, it was not our intention to reveal an objective truth—but to discuss the constructions in light of contextual information about the services.

However, a limitation is that we did not have access to any representatives from the information campaigns’ target groups. Another limitation is that most quotes and pictures applied in the themes are retrieved from the early ads and brochures. They were chosen as they were the most distinct examples of the themes we found. It is also a limitation that our sample did not include all the various types of information provided by TIPS, such as radio programs, video clips, or dialogue-based education for youths and meetings with high school counselors, which could have provided a more nuanced picture.

## Conclusion

This is, to the best of our knowledge, the first study exploring the meaning content in information campaigns related to first-episode psychosis. A broad sample of information material made up the basis for our narrative discourse analysis. We interpreted that three main discourses were built in the material. First, professional knowledge was conveyed as key to promote help-seeking behavior. Second, psychosis was mainly presented within an illness model. Third, psychosis was conveyed as a concern for the wider society. In addition, we focused on the constructed roles and actors that we interpreted these campaigns conveyed. Professionals were usually presented in expert positions, while potential patients seemed to play rather passive roles as receivers of help, people in the surroundings were conveyed as helpers of the mental health system. The positioning of roles pointed to several dilemmas in the mental health services: How can we integrate apparently conflicting views between evidence-based professional knowledge and subjective knowledge obtained through lived experiences? How do we combine a symptom- and illness focus with the expression of belief in the persons own resources? How do we provide knowledge about psychosis when there is no consensus on what it is or what causes it? And, how can we combine the expression of a need for social responsibility with limited role constructions that seem to maintain status quo in the relationship between the mental health services and its surroundings? These are the questions that were raised and discussed based on our findings. We did not intend to provide final answers, but rather contribute to discussion by putting meaning content and discourses on the map. We have, however, tentatively pointed to several ways in which information campaigns within the context of ED services for young adults with first-episode psychosis could be advanced (Additional files 7, 8).

## Additional files

**Additional file 1: Figure S1.** MYTH AND REALITY.

**Additional file 2: Figure S2.** THUMBS UP.

**Additional file 3: Figure S3.** DOMINO.

**Additional file 4: Figure S4.** PHONE.

**Additional file 5: Figure S5.** TIPS TEAMS.

**Additional file 6: Figure S6.** EXPERT AND PATIENT.

**Additional file 7.** Data material part 1. Overview over data used in the analysis.

**Additional file 8.** Data material part 2. Overview over data used in the analysis.

## Data Availability

Data material are available by request to the corresponding author: hege.hansen@hvl.no.

## Abbreviations

DUP: duration of untreated psychosis
TIPS-2: Early Treatment and Intervention in Psychosis Study
ED: early detection
TIPS-study: the Scandinavian early intervention in psychosis
TIPS: early identification and treatment of psychosis
GP: general practitioner
