# Supplementary figures and images for: “We all have a responsibility”: a narrative discourse analysis of an information campaign targeting help-seeking in first episode psychosis
Source: Int J Ment Health Syst. 2019 May 9;13:32. doi: 10.1186/s13033-019-0289-4 (PMC6507175; doi:10.1186/s13033-019-0289-4)

## Slide 1
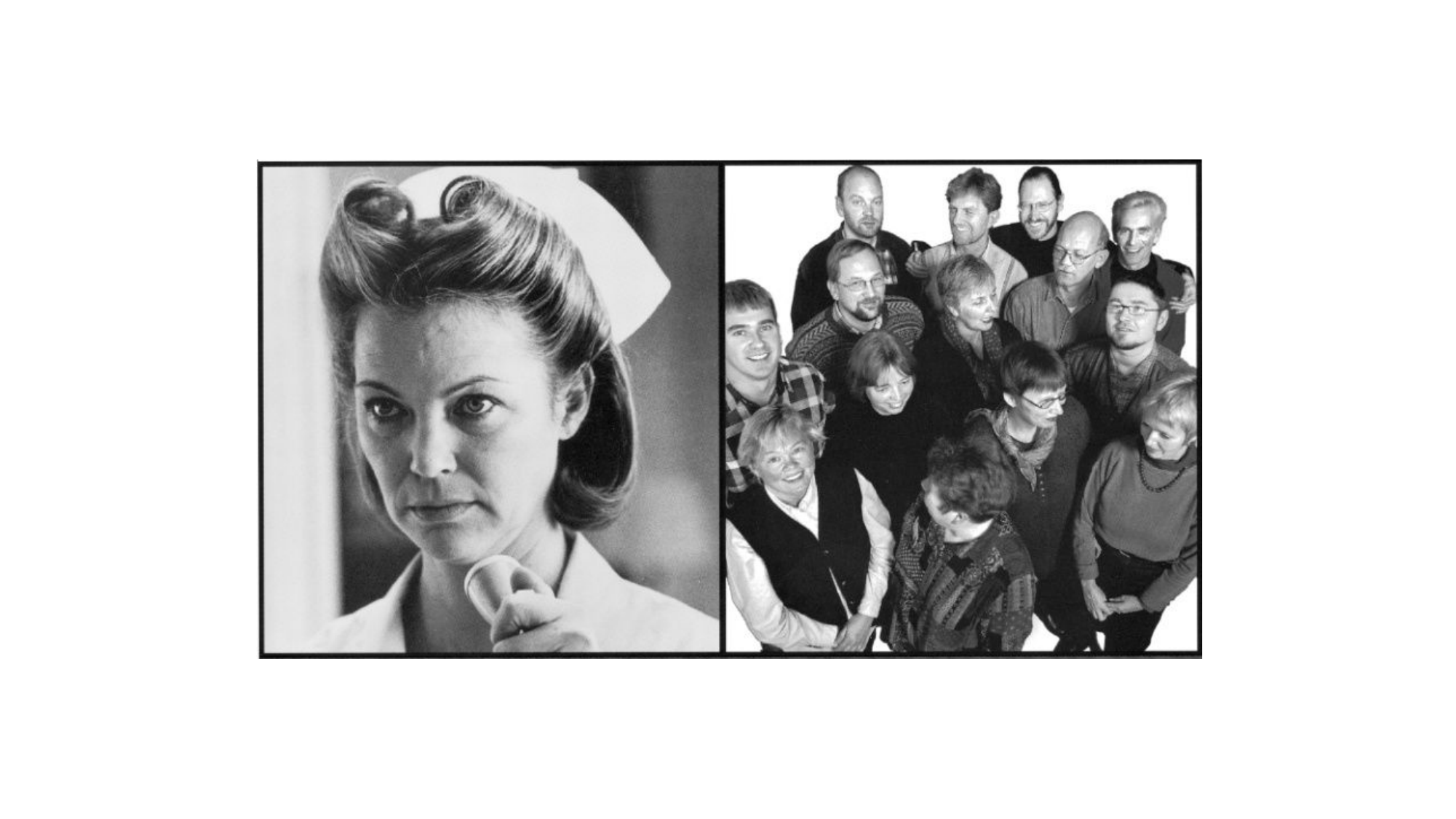

Supplement: Supplementary file 1 — Additional file 1: Figure S1. MYTH AND REALITY. [file 13033_2019_289_MOESM1_ESM.pptx]

## Slide 1
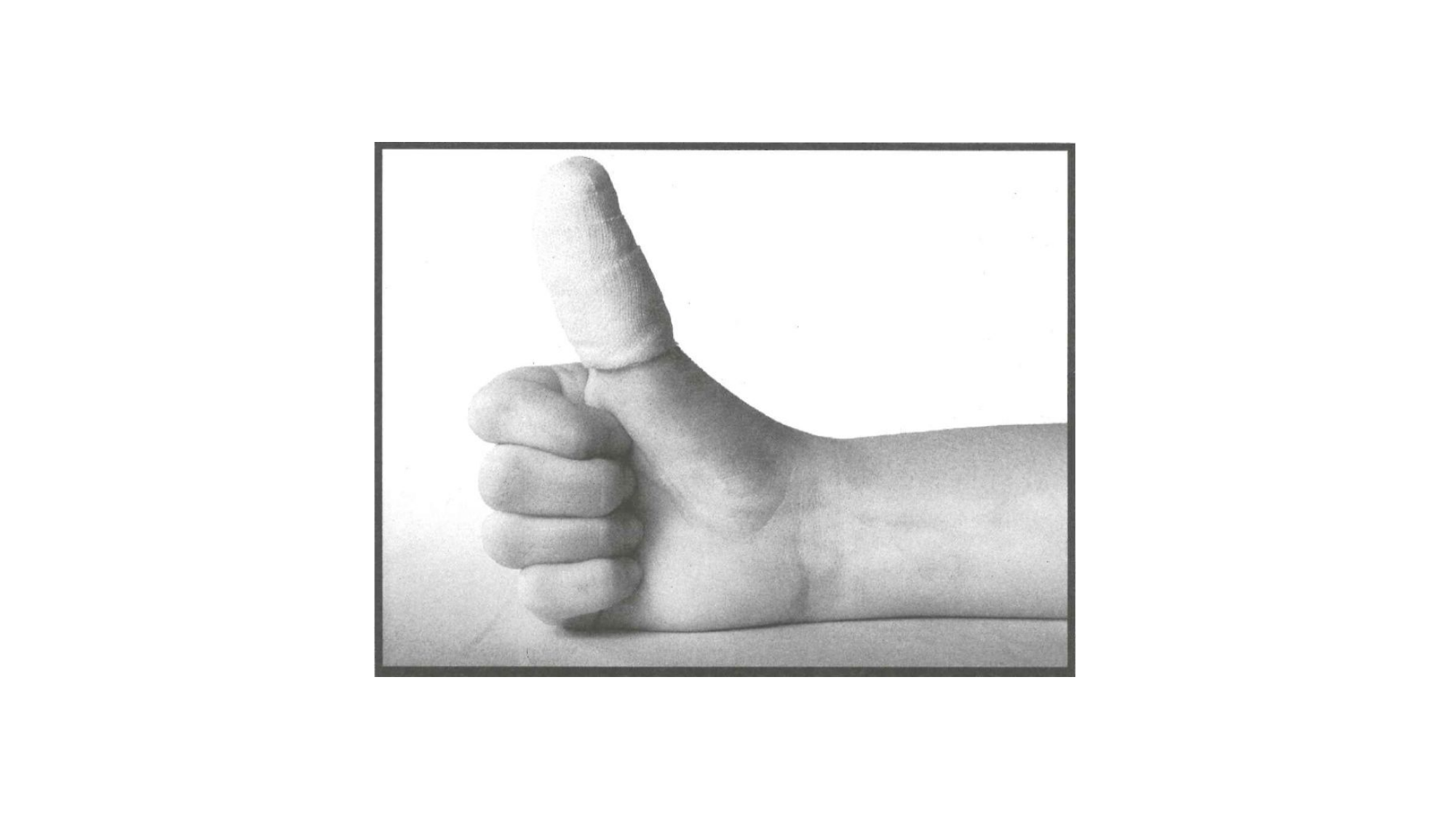

Supplement: Supplementary file 2 — Additional file 2: Figure S2. THUMBS UP. [file 13033_2019_289_MOESM2_ESM.pptx]

## Slide 1
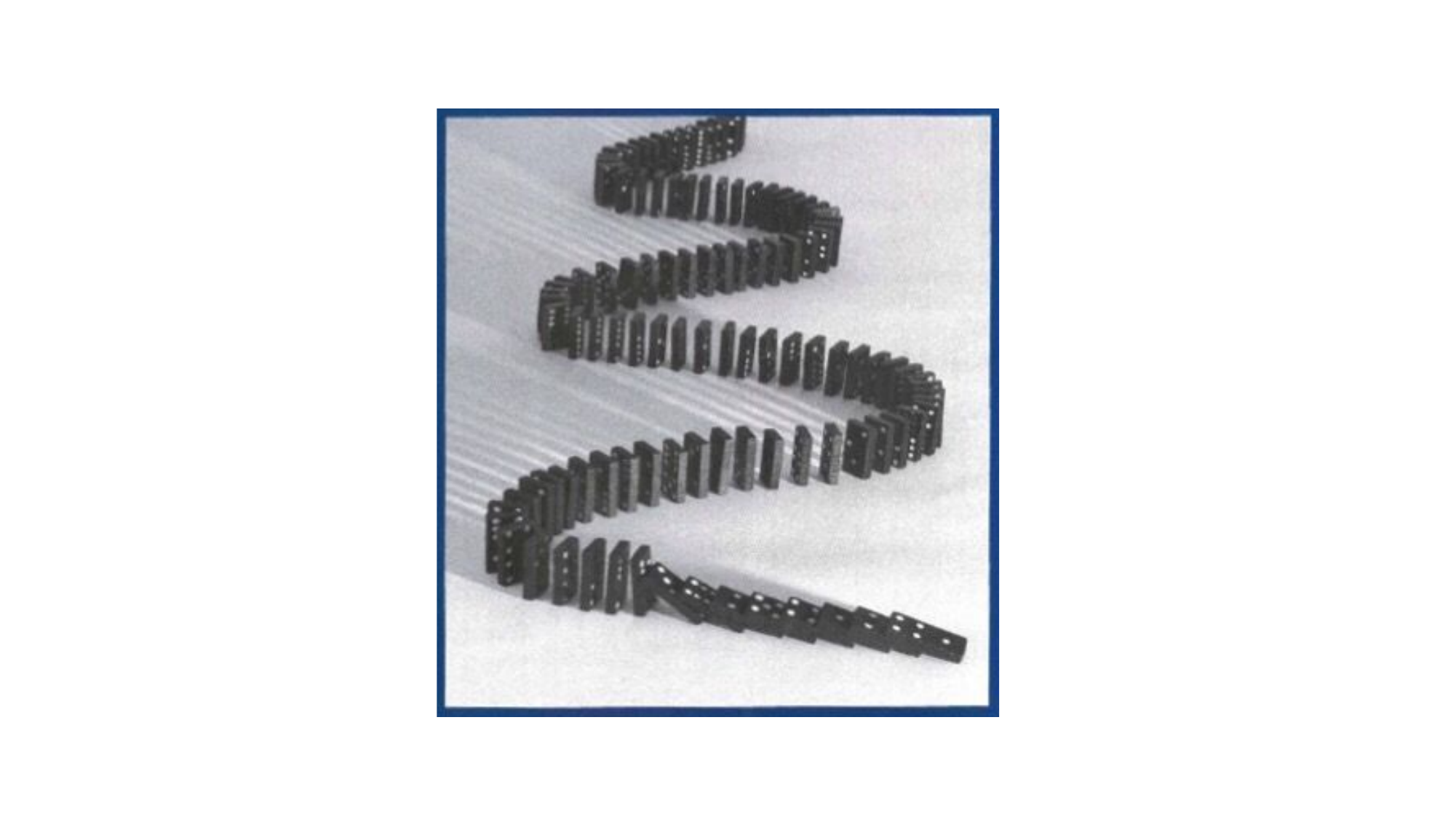

Supplement: Supplementary file 3 — Additional file 3: Figure S3. DOMINO. [file 13033_2019_289_MOESM3_ESM.pptx]

## Slide 1
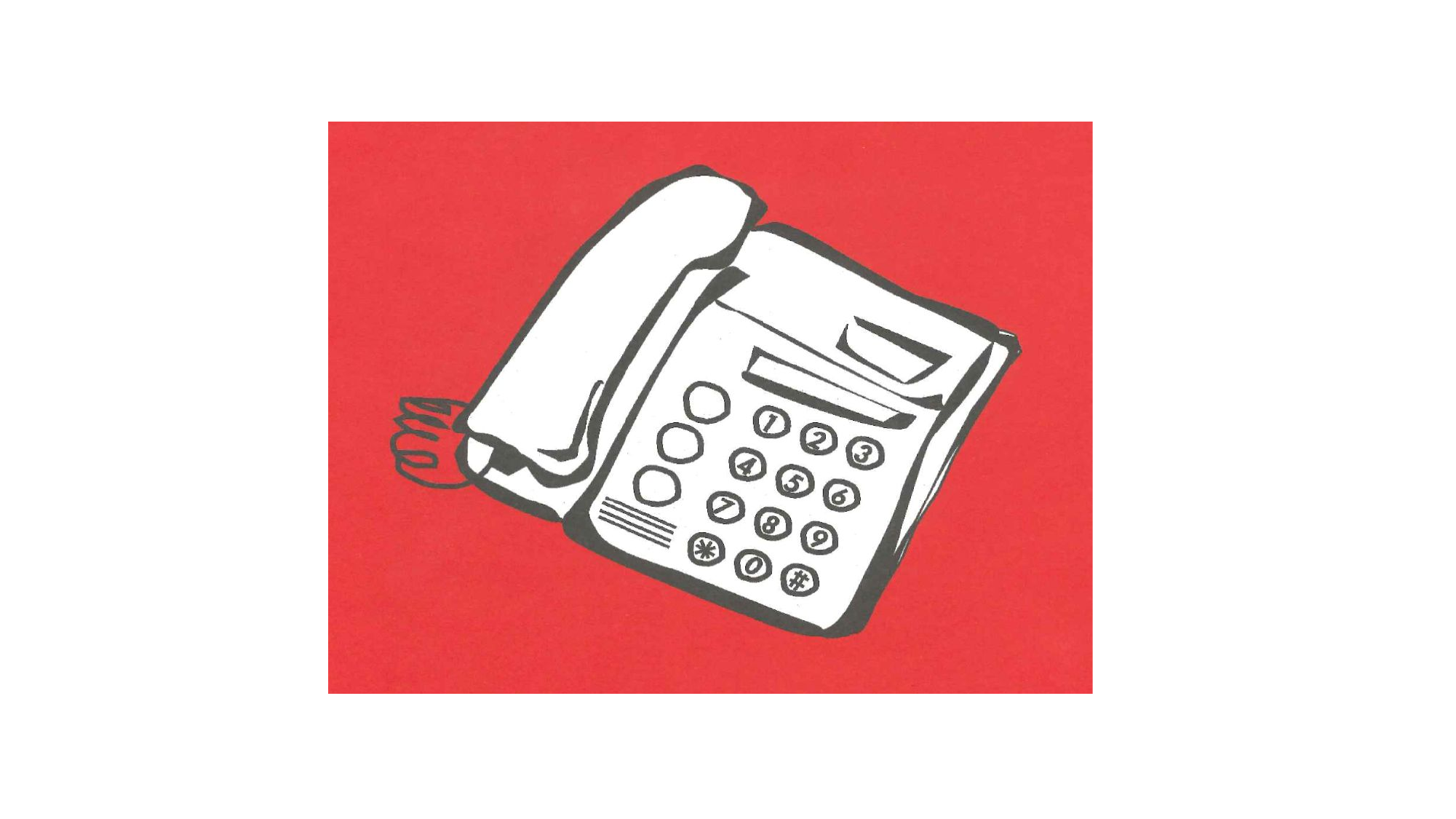

Supplement: Supplementary file 4 — Additional file 4: Figure S4. PHONE. [file 13033_2019_289_MOESM4_ESM.pptx]

## Slide 1
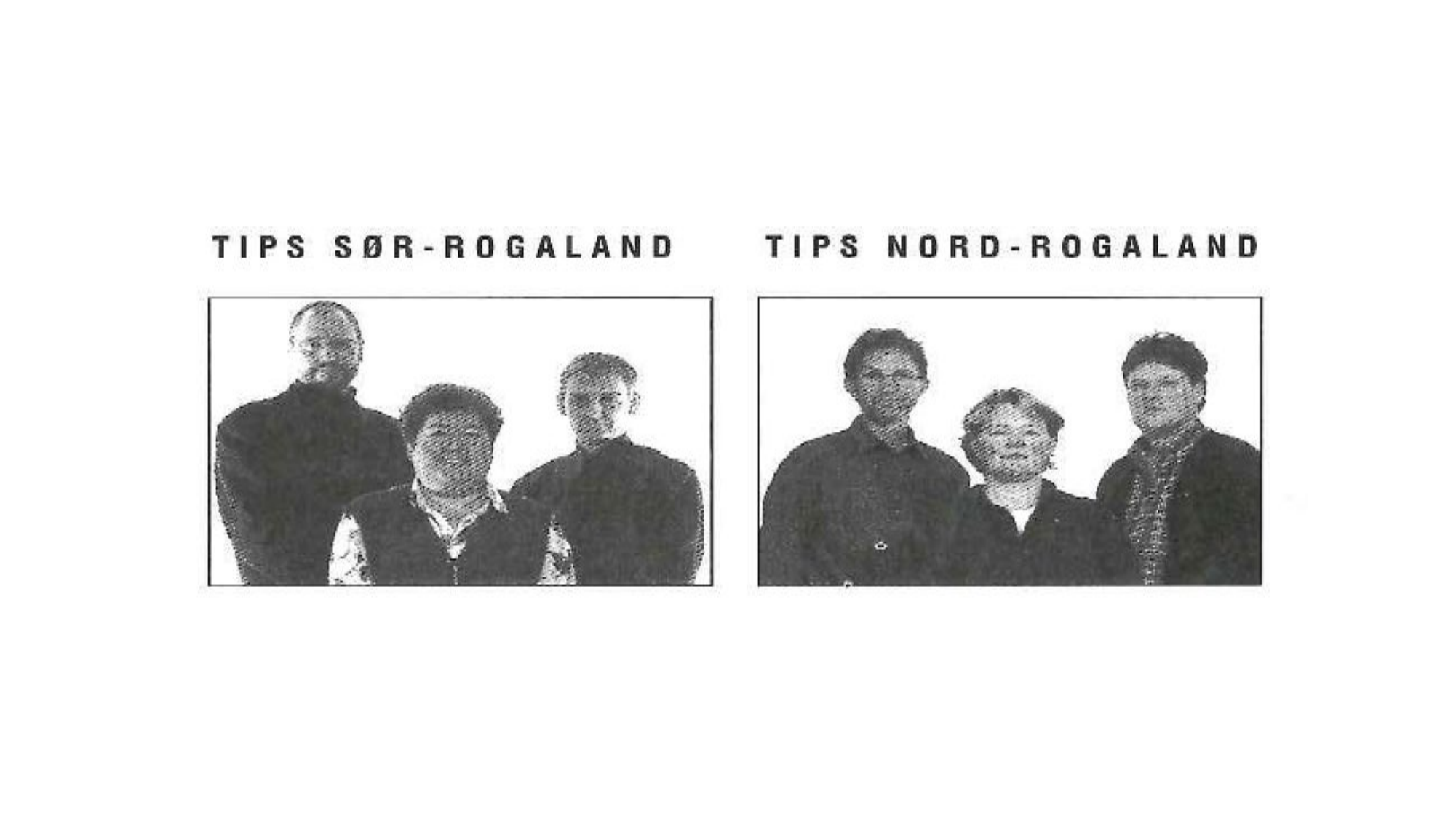

Supplement: Supplementary file 5 — Additional file 5: Figure S5. TIPS TEAMS. [file 13033_2019_289_MOESM5_ESM.pptx]

## Slide 1
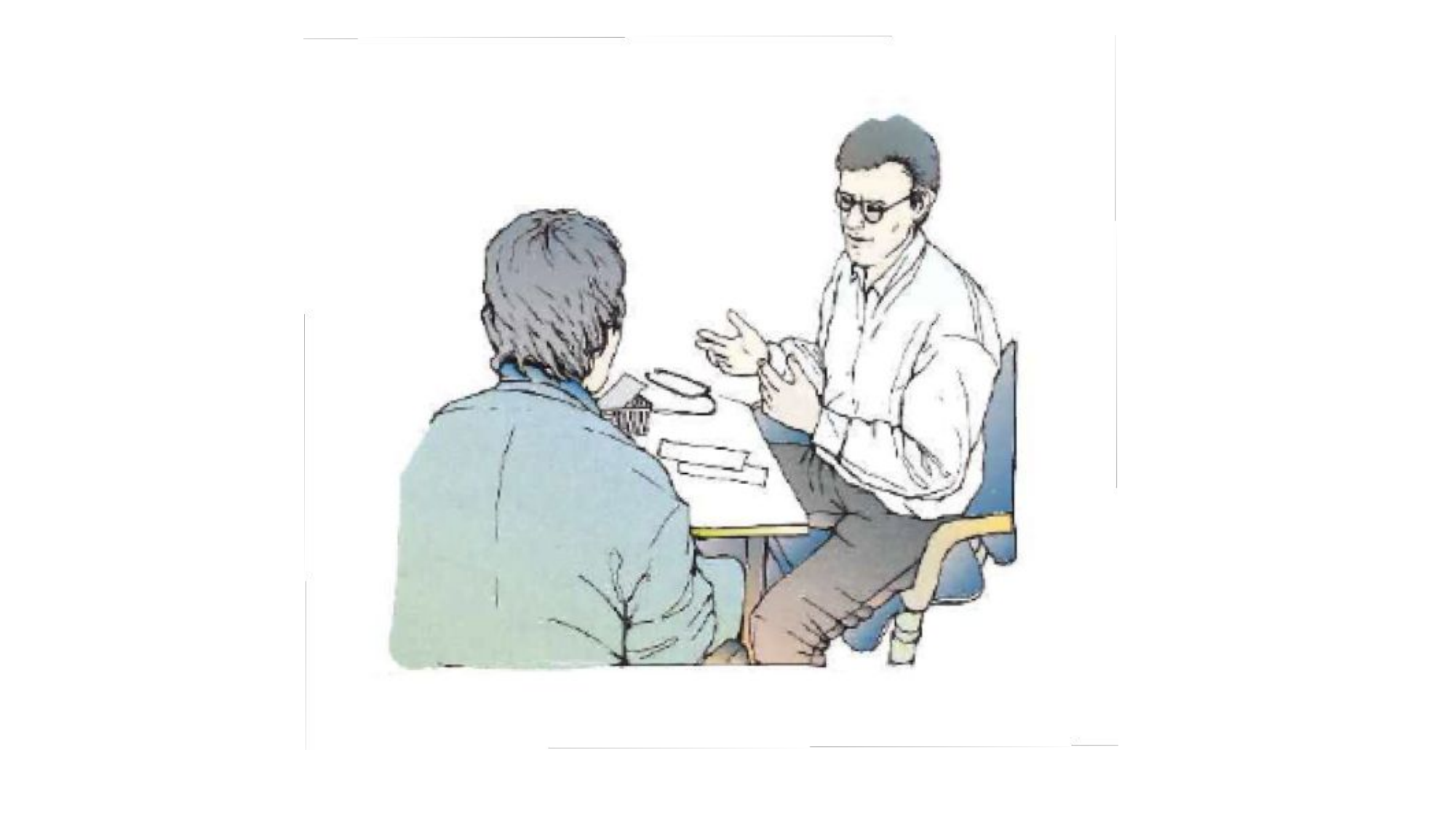

Supplement: Supplementary file 6 — Additional file 6: Figure S6. EXPERT AND PATIENT. [file 13033_2019_289_MOESM6_ESM.pptx]
