# Supplementary material for: “We all have a responsibility”: a narrative discourse analysis of an information campaign targeting help-seeking in first episode psychosis
Source: Int J Ment Health Syst. 2019 May 9;13:32. doi: 10.1186/s13033-019-0289-4 (PMC6507175; doi:10.1186/s13033-019-0289-4)
Supplement: Supplementary file 7 — Additional file 7. Data material part 1. Overview over data used in the analysis. [file 13033_2019_289_MOESM7_ESM.pdf]

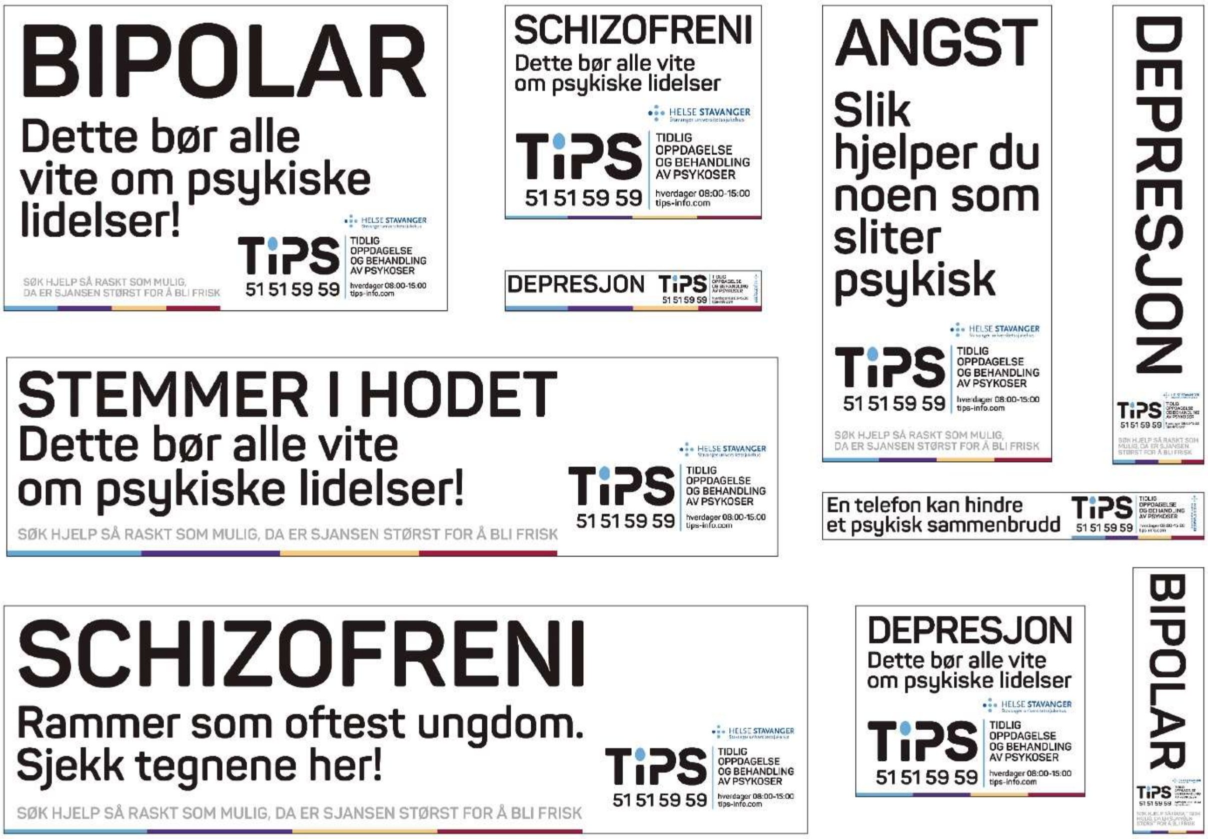
BIPOLAR
Dette bør alle vite om psykiske lidelser!
HELSE STAVANGER
TIPS
TIDLIG OPPDAGELSE OG BEHANDLING AV PSYKOSER
51 51 59 59
hverdager 08:00-15:00
tips-info.com
SØK HJELP SÅ RASKT SOM MULIG, DA ER SJANSEN STØRST FOR Å BLI FRISK
SCHIZOFRENI
Dette bør alle vite om psykiske lidelser
DEPRESJON
ANGST
Slik hjelper du noen som sliter psykisk
DEPRESJON
STEMMER I HODET
Dette bør alle vite om psykiske lidelser!
En telefon kan hindre et psykisk sammenbrudd
SCHIZOFRENI
Rammer som oftest ungdom. Sjekk tegnene her!
DEPRESJON
Dette bør alle vite om psykiske lidelser
BIPOLAR

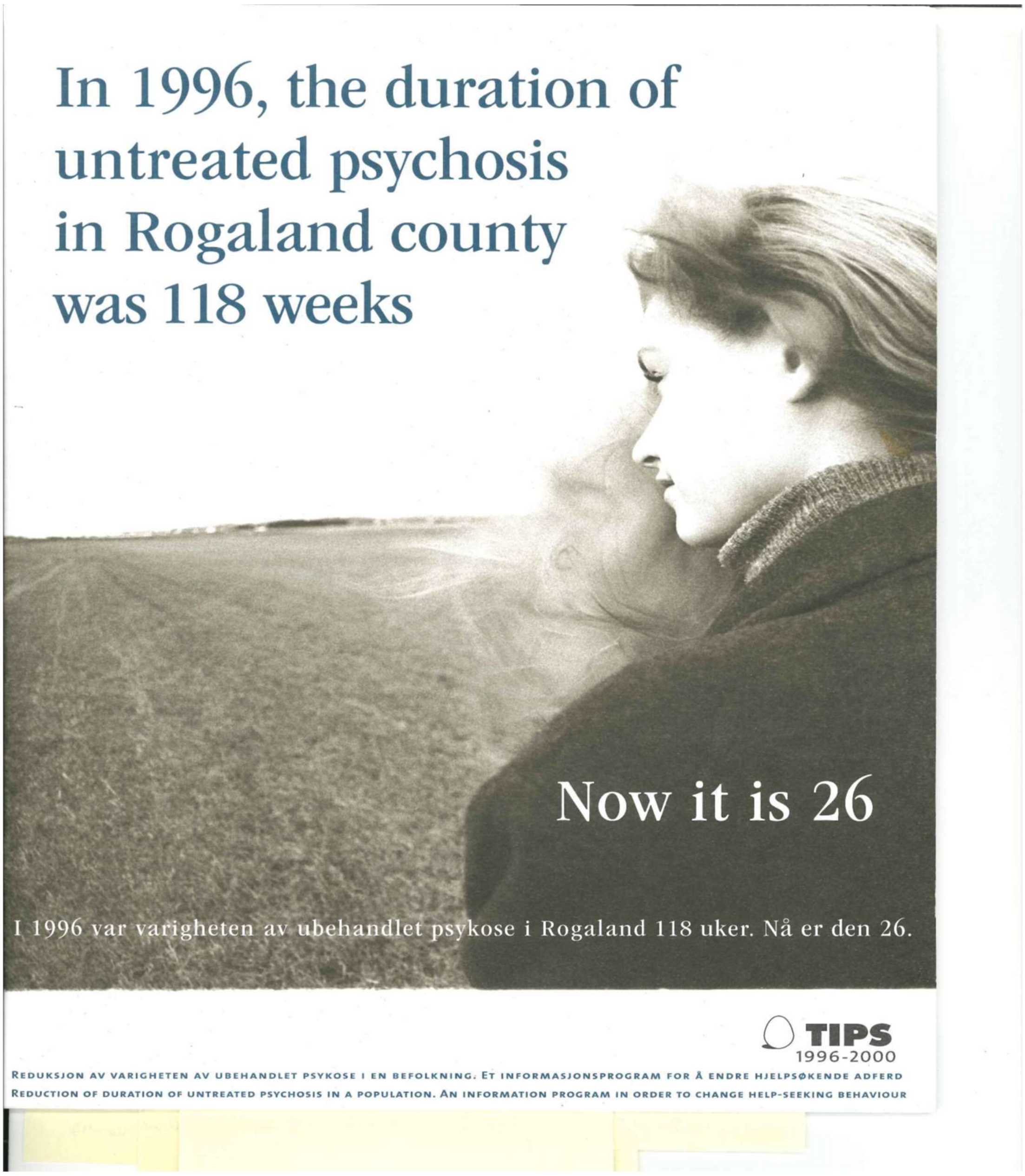
In 1996, the duration of untreated psychosis in Rogaland county was 118 weeks
Now it is 26
I 1996 var varigheten av ubehandlet psykose i Rogaland 118 uker. Nå er den 26.
TIPS
1996-2000
Reduksjon av varigheten av ubehandlet psykose i en befolkning. Et informasjonsprogram for å endre hjelpsøkende adferd
Reduction of duration of untreated psychosis in a population. An information program in order to change help-seeking behaviour

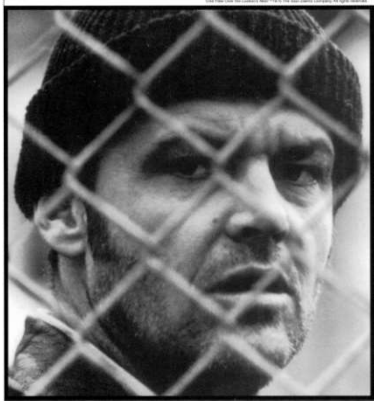

MYTEN

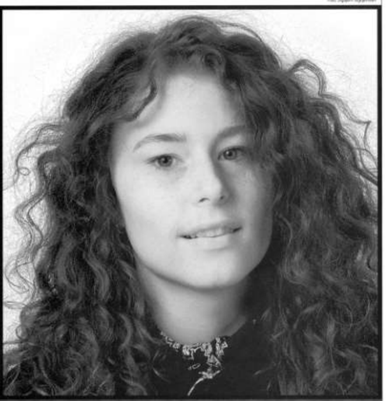

VIRKELIGHETEN

Psykiske lidelser er som andre sykdommer -jo mer du kan om dem jo lettere er det å hjelpe den som rammes

Alvorlige psykiske lidelser starter ofte uskyldig. Den som rammes oppfører seg litt merkelig, men det kan være forbigående. Det er gjentagelsen som varsler fare og som viser at det er nødvendig å søke hjelp.

Mennesker som er i ferd med å utvikle psykiske lidelser trekker seg gjerne tilbake fra familie, venner og arbeids-kolleger. De stenger seg inne, sover dårlig og spiser lite. Mange slutter å vaske og stelle seg. De er ukon-sentrerte og gjør det dårligere på skole og arbeid. De snakker om, eller skriver ting som ikke har mening. De kan ha upassende følelsesmessige reaksjoner, som å le av triste nyheter, eller bli uttrykksløse og ikke reagere i det hele tatt. Enkelte føler seg forfulgt, eller kontrollert av stemmer utenfra. Noen tror at de har magiske evner, at de er en viktig religiøs leder eller en stor politiker. Noen bruker unormalt mye penger.

Hvis disse tingene varer i flere uker er det viktig å søke hjelp raskt slik at behandlingen kan komme fort i gang. Kontakt lege, psykolog eller lege-vakten dersom du trenger råd. Hvis det gjelder skoleelever kan du snakke med skolens rådgiver. 1. januar opp-retter vi dessuten en telefon der du kommer i kontakt med fagfolk som forteller deg hvordan du får hjelp videre.

Søk hjelp så fort som mulig, da er sjansen størst for å bli frisk.

TIPS
TIDLIG OPPDAGELSE OG BEHANDLING AV PSYKOSER

STATENS HELSETILSYN ROGALAND FYLKESKOMMUNE PSYKOPP
FYLKESSJUKEHUSET I HAUGESUND ROGALAND PSYKIATRISKE SYKEHUS

HAAVE RRA76

"One Flew Over the Cuckoo's Nest" ©1975 The Saul Zaentz Company. All rights reserved.

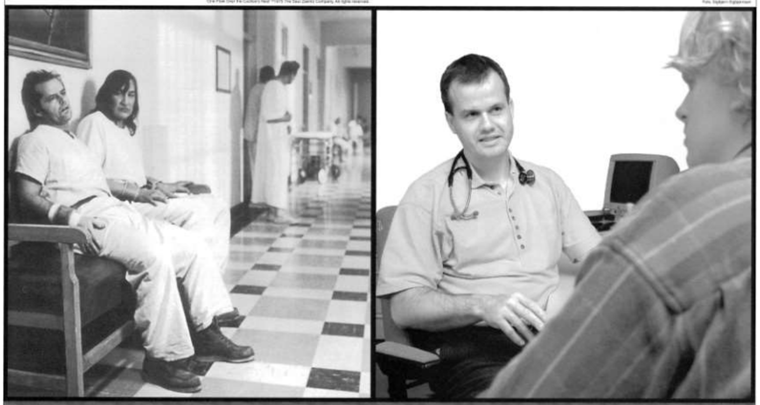

MYTEN VIRKELIGHETEN

Psykiske lidelser er som andre sykdommer –når du føler at noe er galt, går du til legen

Det finnes fortsatt mange myter om hvordan mennesker med alvorlige psykiske lidelser blir innlagt og behandlet. Sannheten er at de aller fleste oppsøker lege på vanlig måte og blir henvist videre til en spesialist.

De fleste får behandling mens de bor hjemme. Innleggelse på psykiatrisk avdeling er så godt som alltid frivillig. I de få tilfellene noen blir tvangsinnlagt er årsaken at de ikke selv sørger for at de får den hjelpen de behøver. Det er meget strenge regler for hvordan dette skal foregå.

Dersom noen du kjenner rammes av psykiske problemer, gjør som ved andre sykdommer: kontakt lege, psykolog eller legevakten dersom du trenger råd. Hvis det gjelder skoleelever kan du snakke med skolens rådgiver. 1. januar oppretter vi dessuten en telefon der du kommer i kontakt med fagfolk som forteller deg hvordan du får hjelp videre.

Tidlige tegn på alvorlige psykiske lidelser

- trekker seg tilbake fra familie, venner og arbeidskolleger
- stenger seg inne, sover dårlig og spiser lite
- slutter å vaske og stelle seg
- er ukonsentrerte
- snakker og skriver om menings løse ting
- har upassende følelsesmessige reaksjoner, som å le av triste nyheter
- blir uttrykksløse og reagerer ikke i det hele tatt
- føler seg forfulgt, eller kontrollert av stemmer utenfra
- tror de har magiske evner

Søk hjelp så fort som mulig, da er sjansen størst for å bli frisk.

TIPS
TIDLIG OPPDAGELSE OG BEHANDLING AV PSYKOSER

STATENS HELSETILSYN ROGALAND FYLKESKOMMUNE PSYKOPP
FYLKESSJUKEHUSET I HAUGESUND ROGALAND PSYKIATRISKE SYKEHUS

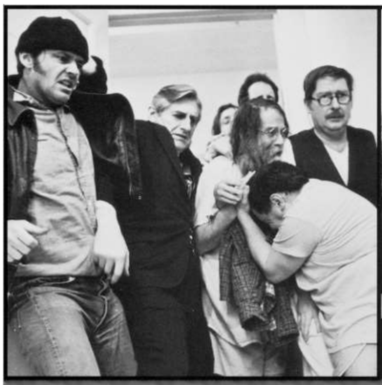

MYTEN

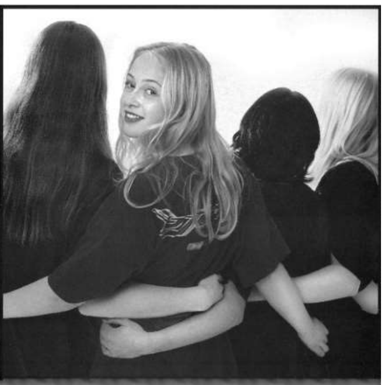

VIRKELIGHETEN

Psykiske lidelser er som andre sykdommer – starter behandlingen tidlig er sjansen stor for at du blir frisk og kan nyte livet

Det behøver ikke være noen katastrofe å bli rammet av psykiske lidelser. Hvis de oppdages tidlig og behandlingen starter raskt, er muligheten for å bli frisk stor. For de fleste betyr det at de kan skape sitt eget hjem, ta utdannelse og få en jobb.

Selv om noen få må ha behandling livet ut betyr det ikke at de skal tilbringe all sin tid på sykehus. I all behandling legges det stor vekt på at pasienten kan bo hjemme og at kontakten med venner og familie opprettholdes. Det betyr mye at venner stiller opp og holder kontakten i den tiden behandlingen pågår. Noen synes kanskje det kan være vanskelig, men ved å være ærlig, åpen og å lære litt mer om psykiske lidelser kan en slik støtte bety svært mye.

1. januar styrkes det psykiske helsetilbudet i Rogaland med nye stillinger som fordeles over hele fylket. Det betyr at vi kan gjøre en bedre jobb.

Vi har laget en brosjyre som forteller om psykiske lidelser, hva det er og hvordan de behandles. Der finner du også en rekke tips og råd som er nyttige enten du selv rammes, eller noen du kjenner behøver støtte. Du finner den i postkassen like over nyttår. Les den, ta vare på den og bli klokere.

Tidlige tegn på alvorlige psykiske lidelser

- trekker seg tilbake fra familie, venner og arbeidskolleger
- stenger seg inne, sover dårlig og spiser lite
- slutter å vaske og stelle seg
- er ukonsentrerte
- snakker og skriver om menings løse ting
- har upassende følelsesmessige reaksjoner, som å le av triste nyheter
- blir uttrykksløse og reagerer ikke i det hele tatt
- føler seg forfulgt, eller kontrollert av stemmer utenfra
- tror de har magiske evner

Søk hjelp så fort som mulig, da er sjansen størst for å bli frisk.

TIPS
TIDLIG OPPDAGELSE OG BEHANDLING AV PSYKOSER

STATENS HELSETILSYN ROGALAND FYLKESKOMMUNE PSYKOPP
FYLKESSJUKEHUSET I HAUGESUND ROGALAND PSYKIATRISKE SYKEHUS

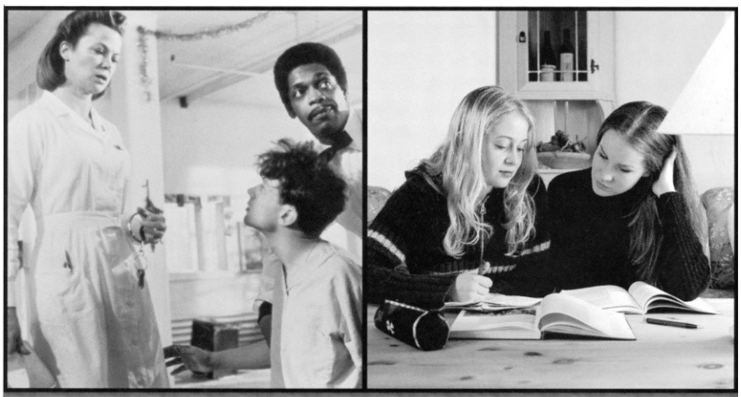

MYTEN VIRKELIGHETEN

Psykiske lidelser er som andre sykdommer –de fleste kan få behandling mens de bor hjemme

Fordi det finnes mange former for psykiske lidelser, er det også ulike behandlingsformer.

De fleste tilfellene er akutte og forbigående, noen ganger kan de vare livet ut. Den viktigste behandlingen er psykoterapi, eller samtalebehandling omkring pasientens problemer og livssituasjon. De fleste pasientene bor hjemme og kommer til sykehuset med jevne mellomrom for å motta behandling. Bare når det kreves mer omfattende behandling velges sykehusinnleggelse med miljøbehandling. Medikamentell behandling brukes først og fremst for å gjøre pasienten mottagelig for andre behandlingsformer. Målet er alltid at pasienten skal fortsette i jobb eller skole, holde kontakt med venner og familie og få en best mulig livskvalitet. I likhet med andre sykdommer behøver ikke psykiske lidelser stå i veien for et godt liv.

Dersom noen du kjenner rammes av alvorlige psykiske problemer, gjør som ved andre sykdommer: kontakt lege, psykolog eller legevakten dersom du trenger råd. Hvis det gjelder skoleelever kan du snakke med skolens rådgiver.
1. januar oppretter vi dessuten en telefon der du kommer i kontakt med fagfolk som forteller deg hvordan du får hjelp videre.

Tidlige tegn på alvorlige psykiske lidelser

- trekker seg tilbake fra familie, venner og arbeidskolleger
- stenger seg inne, sover dårlig og spiser lite
- slutter å vaske og stelle seg
- er ukonsentrerte
- snakker og skriver om menings løse ting
- har upassende følelsesmessige reaksjoner, som å le av triste nyheter
- blir uttrykksløse og reagerer ikke i det hele tatt
- føler seg forfulgt, eller kontrollert av stemmer utenfra
- tror de har magiske evner

Søk hjelp så fort som mulig, da er sjansen størst for å bli frisk.

TIPS
TIDLIG OPPDAGELSE OG BEHANDLING AV PSYKOSER

STATENS HELSETILSYN ROGALAND FYLKESKOMMUNE PSYKOPP
FYLKESSJUKEHUSET I HAUGESUND ROGALAND PSYKIATRISKE SYKEHUS

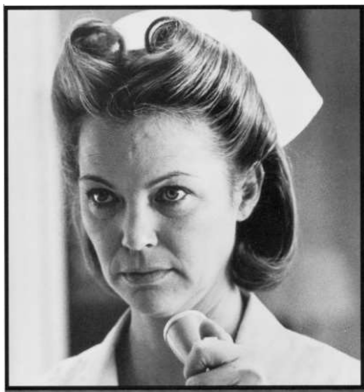

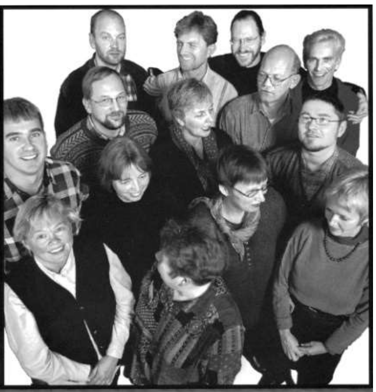

MYTEN — VIRKELIGHETEN

Den beste måten å slå hull på myten er å ta kontakt med oss

Det har skjedd mye innen behandling av psykiske lidelser de siste tiårene, men fortsatt henger det igjen gamle myter. Glem Gjøkeredet og andre skrekkfilmer som henter sin handling fra psykiatriske sykehus.

I dag er psykiatriske sykehus åpne institusjoner og de fleste som får behandling bor hjemme og arbeider eller går på skole. De menneskene du møter er fagfolk, som psykiatriske sykepleiere, sosialkonsulenter, psykologer og psykiatere. Det er krevende yrker og de som velger dem er motiverte og høyt kvalifiserte for arbeidet. I de tilfellene det er nødvendig med sykehusinnleggelse skal oppholdet være så kort som mulig.

1. januar styrkes behandlingstilbudet i Rogaland. Det er bevilget penger til flere nye stillinger som fordeles over hele fylket. Dette gjør det mulig å starte behandlingen tidligere og øker sjansene for at de som trenger behandling blir friske.

Dersom noen du kjenner rammes av psykiske problemer, gjør som ved andre sykdommer: kontakt lege, psykolog eller legevakten dersom du trenger råd. Hvis det gjelder skoleelever kan du snakke med skolens rådgiver. 1. januar oppretter vi dessuten en telefon der du kommer i kontakt med fagfolk som forteller deg hvordan du får hjelp videre.

Tidlige tegn på alvorlige psykiske lidelser

- trekker seg tilbake fra familie, venner og arbeidskolleger
- stenger seg inne, sover dårlig og spiser lite
- slutter å vaske og stelle seg
- er ukonsentrerte
- snakker og skriver om menings løse ting
- har upassende følelsesmessige reaksjoner, som å le av triste nyheter
- blir uttrykksløse og reagerer ikke i det hele tatt
- føler seg forfulgt, eller kontrollert av stemmer utenfra
- tror de har magiske evner

Søk hjelp så fort som mulig, da er sjansen størst for å bli frisk.

TIPS
TIDLIG OPPDAGELSE OG BEHANDLING AV PSYKOSER

STATENS HELSETILSYN ROGALAND FYLKESKOMMUNE PSYKOPP
FYLKESSJUKEHUSET I HAUGESUND ROGALAND PSYKIATRISKE SYKEHUS

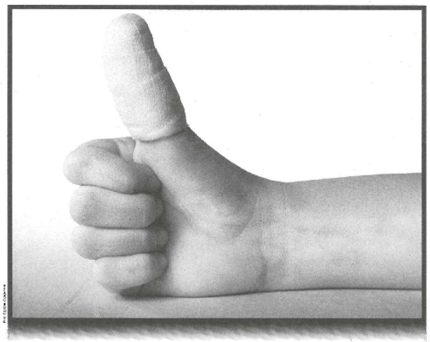

På ett punkt er psykiske lidelser helt like andre sykdommer

–når hjelpen settes inn tidlig er sjansene størst for å bli frisk.

Nå får unge mennesker med alvorlige psykiske lidelser et bedre behandlingstilbud

Et av de største problemene innen behandling av alvorlige psykiske lidelser - psykoser - er at pasientene kommer for sent til behandling. Det kan faktisk gå opptil 3 år fra de første tegnene på sykdom viser seg til pasienten kommer til behandling. Da er ofte tilstanden så alvorlig at pasienten behøver uforholdsmessig lang og ressurskrevende behandling. For unge mennesker som rammes er dette tragisk og kan få store konsekvenser dersom de ikke får hjelp. For å forsøke å rette på dette har Rogaland fått penger til et prosjekt hvor en skal få pasientene tidligere til behandling. Det blir opprettet flere nye fagstillinger som fordeles over hele fylket. Disse skal jobbe med poliklinisk behandling av pasienter og hjelpe andre deler av helsevesenet som kommer i kontakt med psykiske lidelser. De skal også drive et omfattende opplysningsarbeid for helsepersonell og på videregående skoler. Det er også laget en informasjonskampanje som er rettet mot alle som bor i Rogaland. I de neste ukene kommer det annonser i de største avisene, og i januar får du en brosjyre i postkassen. Les og bli klokere, dette er viktig.

Vi trenger din hjelp for å komme dit vi ønsker

Hvis du synes det er sløsing med penger å informere om dette prosjektet, skal du tenke på dette: Det finnes mange myter omkring psykiske lidelser og behandlingen av dem. Det virker skremmende for mange og fører til at de ikke oppsøker hjelp, derfor vil vi gi et sant bilde av dagens muligheter. Dersom de syke får hjelp tidligere er sjansen større for å bli frisk og de behøver mindre tid på sykehuset. Det sparer samfunnet penger på og den syke får et bedre liv.
Dersom du vet mer om psykiske lidelser kan du hjelpe andre til tidlig behandling. Det er ingenting å være redd for.

Forebyggende arbeid og tidlig behandling kan gjøre livet bedre for mange mennesker

Før eller siden vil du oppleve at noen som står deg nær får psykiske problemer. Det kan oppstå gradvis, eller plutselig - som en reaksjon på at noen i familien dør, skilsmisse, problemer på skole eller jobb, flytting - eller andre vanskelige situasjoner. Dette er normale reaksjoner og er som regel forbigående. Ved å snakke ut om problemene, først og fremst med de nærmeste, men også med en lege eller psykolog, vil de fleste finne veien tilbake til et normalt liv igjen. Vennskap, støtte og forståelse gjør det lettere å komme gjennom slike perioder.

Tidlige tegn på alvorlige psykiske lidelser

Alvorlige psykiske lidelser har ikke alltid en årsak som er lett å få øye på. Hos unge mennesker kan de utvikle seg gradvis og kan ligne normale reaksjoner på vanskelige situasjoner i ungdomstiden. Her er noen tegn du skal være på vakt overfor:
- trekker seg tilbake fra familie, venner og arbeidskolleger
- stenger seg inne, sover dårlig og spiser lite
- slutter å vaske og stelle seg
- er ukonsentrerte
- snakker og skriver om meningsløse ting
- har upassende følelsesmessige reaksjoner, som å le av triste nyheter
- blir uttrykksløse og reagerer ikke i det hele tatt
- føler seg forfulgt, eller kontrollert av stemmer utenfra
- tror de har magiske evner

1. januar blir det lettere å få hjelp

Dersom noen du kjenner rammes av alvorlige psykiske problemer, gjør som ved andre sykdommer: kontakt lege, psykolog eller skolens rådgiver. De vil hjelpe med å vurdere om det er behov for behandling på sykehus. 1. januar oppretter vi dessuten en telefon der du kommer i kontakt med fagfolk som kan fortelle deg hvordan du kan få hjelp videre.

Søk hjelp så fort som mulig, da er sjansen størst for å bli frisk.

TIPS

STATENS HELSETILSYN ROGALAND FYLKESKOMMUNE PSYKOPP
FYLKESSJUKEHUSET I HAUGESUND ROGALAND PSYKIATRISKE SYKEHUS

At one point psychiatric disorders are like all other illnesses -when treatment is started early there is greater chance for recovery.

annonsering / advertisement

12

s 24 -
Mand
s 38 - 42 + 44,46-47

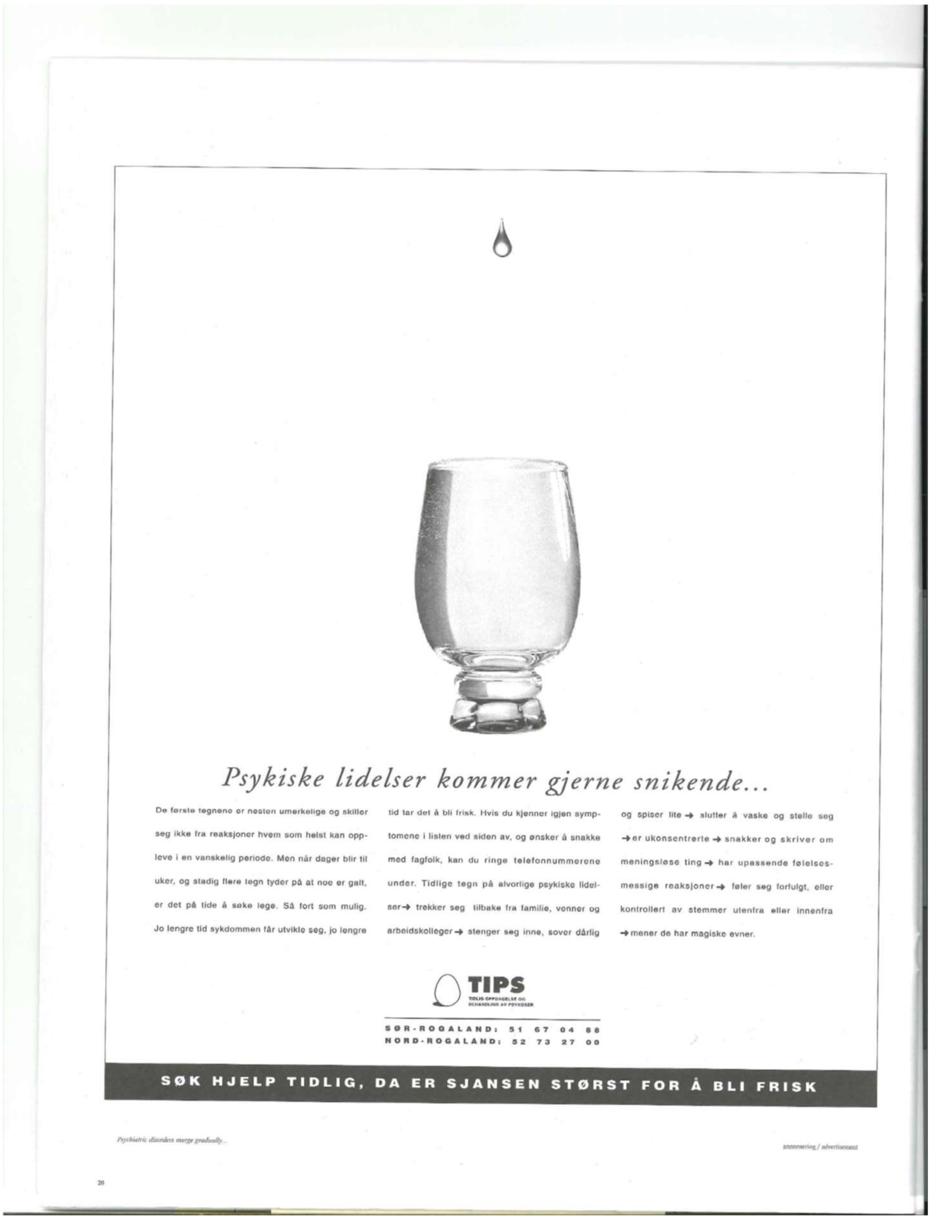
Psykiske lidelser kommer gjerne snikende...
De første tegnene er nesten umerkelige og skiller seg ikke fra reaksjoner hvem som helst kan oppleve i en vanskelig periode. Men når dager blir til uker, og stadig flere tegn tyder på at noe er galt, er det på tide å søke lege. Så fort som mulig. Jo lengre tid sykdommen får utvikle seg, jo lengre tid tar det å bli frisk. Hvis du kjenner igjen symptomene i listen ved siden av, og ønsker å snakke med fagfolk, kan du ringe telefonnummerene under. Tidlige tegn på alvorlige psykiske lidelser → trekker seg tilbake fra familie, venner og arbeidskolleger → stenger seg inne, sover dårlig og spiser lite → slutter å vaske og stelle seg → er ukonsentrerte → snakker og skriver om meningsløse ting → har upassende følelsesmessige reaksjoner → føler seg forfulgt, eller kontrollert av stemmer utenfra eller innenfra → mener de har magiske evner.
TIPS
TIDLIG OPPDAGELSE OG BEHANDLING AV PSYKOSER
SØR-ROGALAND: 51 67 04 88
NORD-ROGALAND: 52 73 27 00
SØK HJELP TIDLIG, DA ER SJANSEN STØRST FOR Å BLI FRISK
Psychiatric disorders merge gradually...
annonsering / advertisement
20

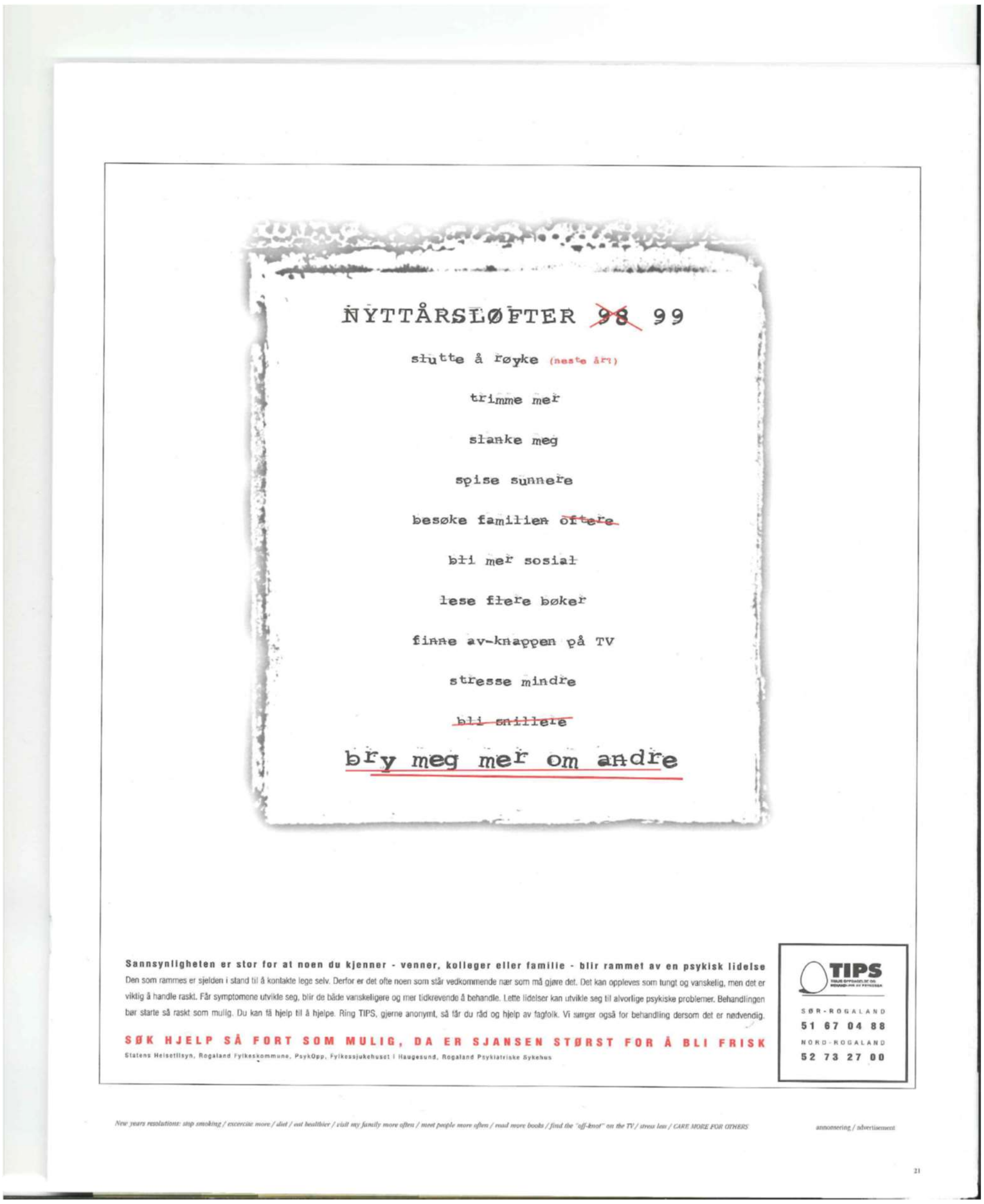
NYTTÅRSLØFTER 98 99
slutte å røyke (neste år)
trimme mer
slanke meg
spise sunnere
besøke familien oftere
bli mer sosial
lese flere bøker
finne av-knappen på TV
stresse mindre
bli snillere
bry meg mer om andre
Sannsynligheten er stor for at noen du kjenner - venner, kolleger eller familie - blir rammet av en psykisk lidelse
Den som rammes er sjelden i stand til å kontakte lege selv. Derfor er det ofte noen som står vedkommende nær som må gjøre det. Det kan oppleves som tungt og vanskelig, men det er viktig å handle raskt. Får symptomene utvikle seg, blir de både vanskeligere og mer tidkrevende å behandle. Lette lidelser kan utvikle seg til alvorlige psykiske problemer. Behandlingen bør starte så raskt som mulig. Du kan få hjelp til å hjelpe. Ring TIPS, gjerne anonymt, så får du råd og hjelp av fagfolk. Vi sørger også for behandling dersom det er nødvendig.
SØK HJELP SÅ FORT SOM MULIG, DA ER SJANSEN STØRST FOR Å BLI FRISK
Statens Helsetilsyn, Rogaland Fylkeskommune, PsykOpp, Fylkessjukehuset i Haugesund, Rogaland Psykiatriske Sykehus
TIPS
SØR-ROGALAND
51 67 04 88
NORD-ROGALAND
52 73 27 00
New years resolutions: stop smoking / excercise more / diet / eat healthier / visit my family more often / meet people more often / read more books / find the "off-knot" on the TV / stress less / CARE MORE FOR OTHERS
annonsering / advertisement
21

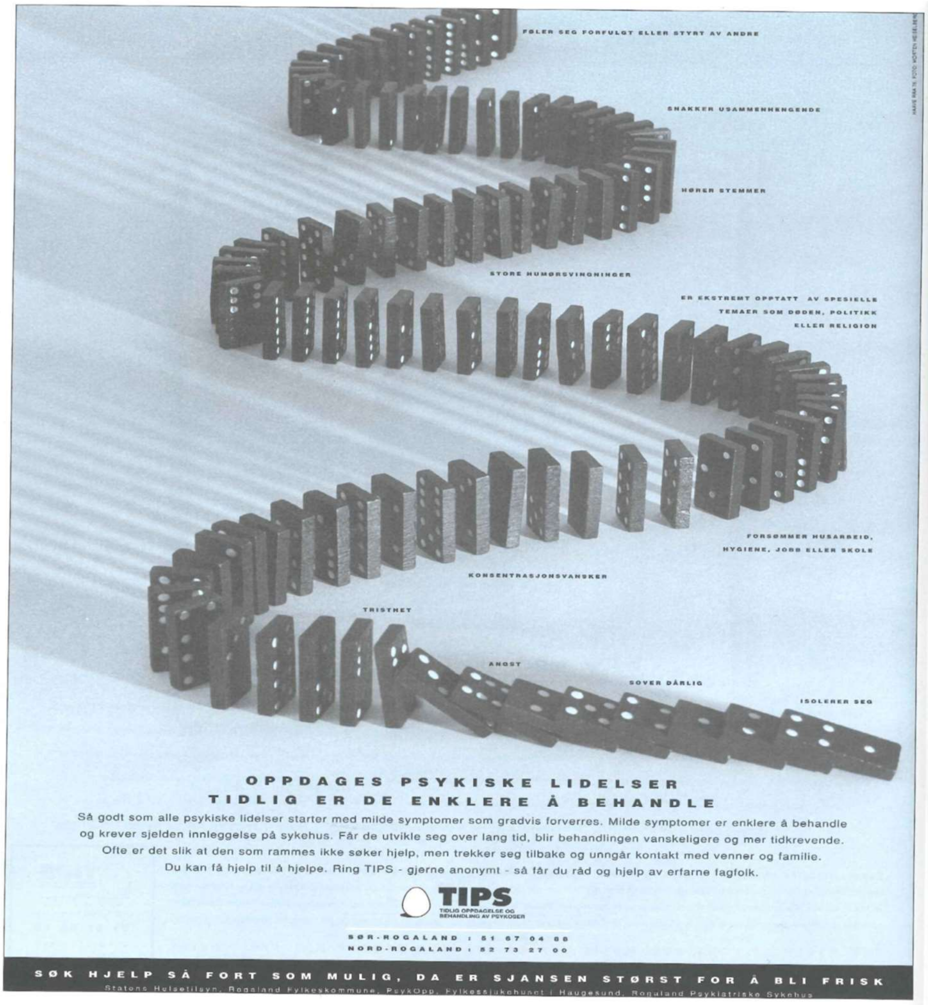

If psychiatric disorders are detected early, they are easier to treat.

annonsering / advertisement

22

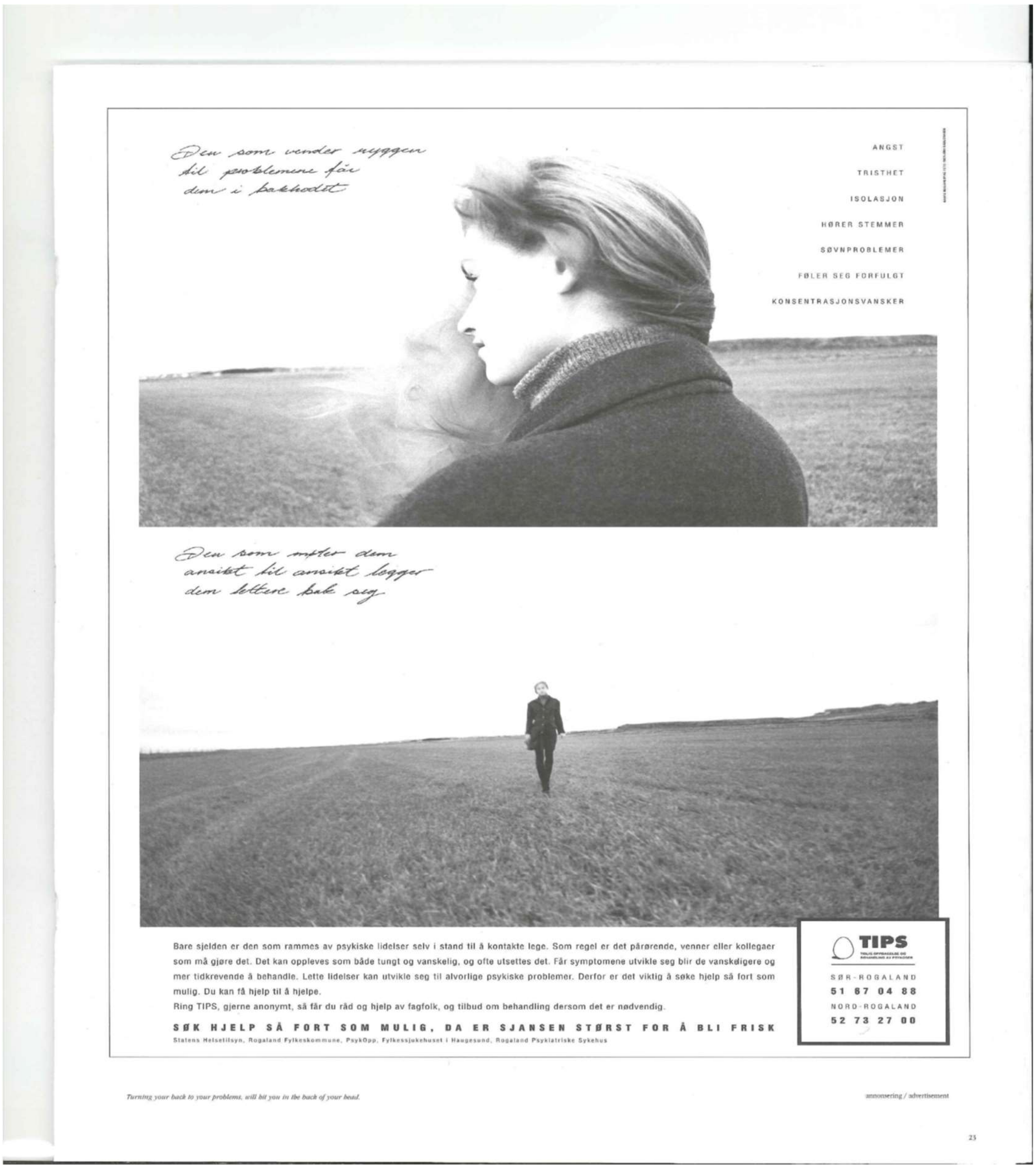

Den som vender ryggen til problemene får dem i bakhodet
ANGST
TRISTHET
ISOLASJON
HØRER STEMMER
SØVNPROBLEMER
FØLER SEG FORFULGT
KONSENTRASJONSVANSKER
Den som møter dem ansikt til ansikt legger dem lettere bak seg.
Bare sjelden er den som rammes av psykiske lidelser selv i stand til å kontakte lege. Som regel er det pårørende, venner eller kollegaer som må gjøre det. Det kan oppleves som både tungt og vanskelig, og ofte utsettes det. Får symptomene utvikle seg blir de vanskeligere og mer tidkrevende å behandle. Lette lidelser kan utvikle seg til alvorlige psykiske problemer. Derfor er det viktig å søke hjelp så fort som mulig. Du kan få hjelp til å hjelpe.
Ring TIPS, gjerne anonymt, så får du råd og hjelp av fagfolk, og tilbud om behandling dersom det er nødvendig.
SØK HJELP SÅ FORT SOM MULIG, DA ER SJANSEN STØRST FOR Å BLI FRISK
Statens Helsetilsyn, Rogaland Fylkeskommune, PsykOpp, Fylkessjukehuset i Haugesund, Rogaland Psykiatriske Sykehus
TIPS
SØR-ROGALAND
51 67 04 88
NORD-ROGALAND
52 73 27 00
Turning your back to your problems, will hit you in the back of your head.
annonsering / advertisement
23

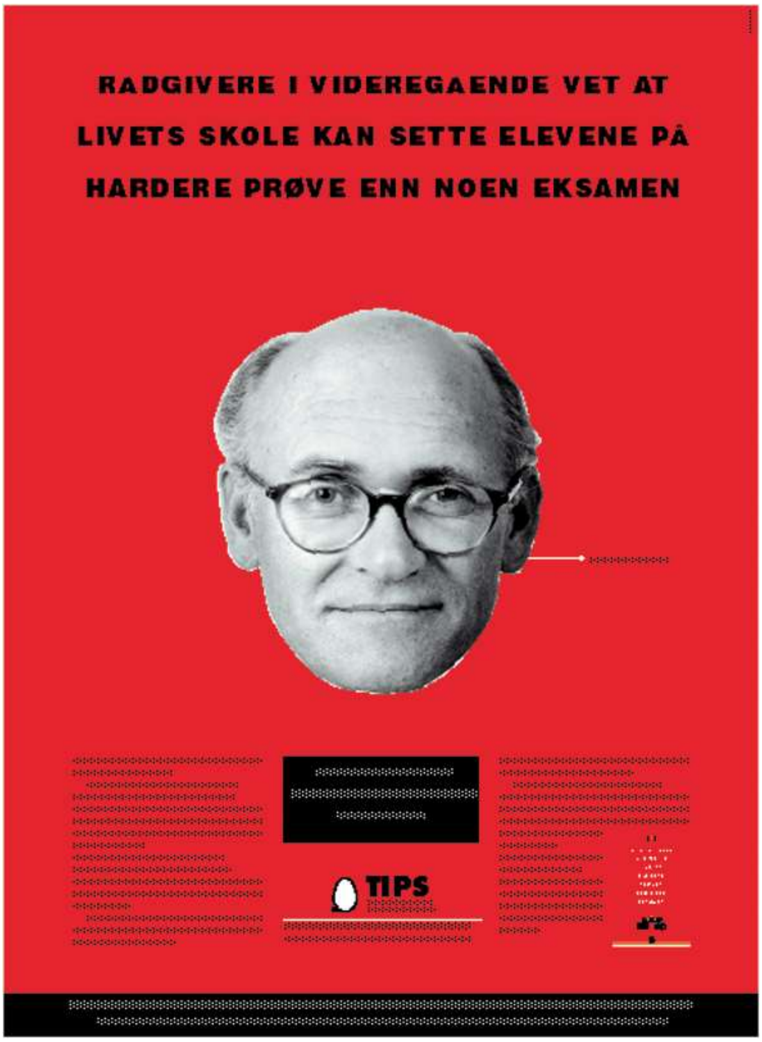
RADGIVERE I VIDEREGAENDE VET AT
LIVETS SKOLE KAN SETTE ELEVENE PÅ
HARDERE PRØVE ENN NOEN EKSAMEN
TIPS

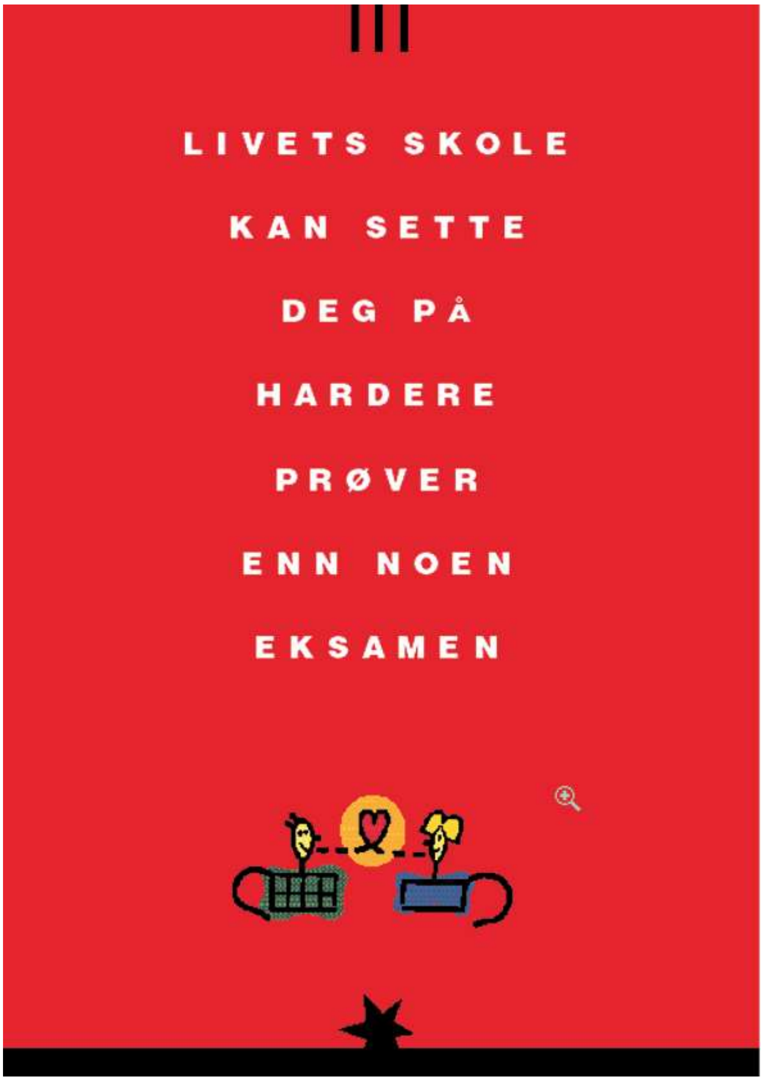
LIVETS SKOLE
KAN SETTE
DEG PÅ
HARDERE
PRØVER
ENN NOEN
EKSAMEN

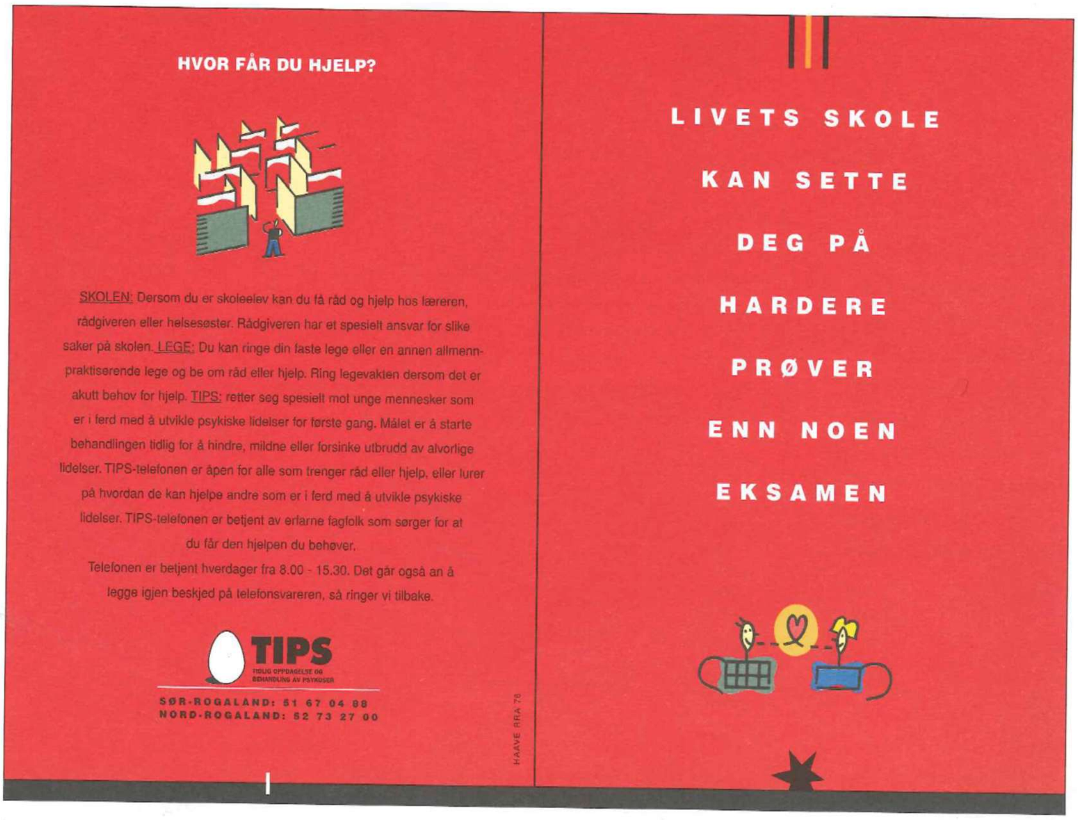

The school of life can put you on harder tests than any exam.

brosjyre / brochure

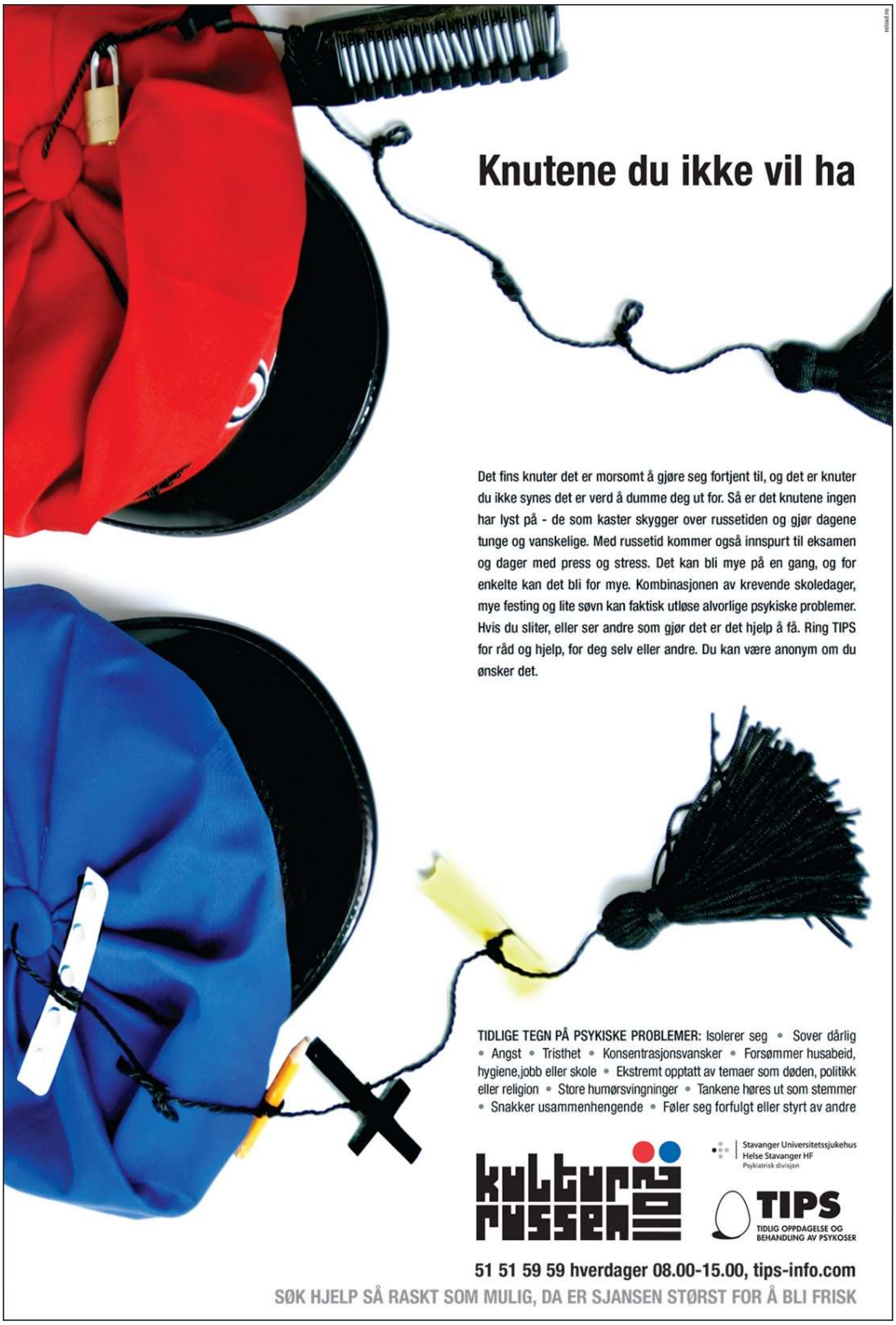

Knutene du ikke vil ha
Det fins knuter det er morsomt å gjøre seg fortjent til, og det er knuter du ikke synes det er verd å dumme deg ut for. Så er det knutene ingen har lyst på - de som kaster skygger over russetiden og gjør dagene tunge og vanskelige. Med russetid kommer også innspurt til eksamen og dager med press og stress. Det kan bli mye på en gang, og for enkelte kan det bli for mye. Kombinasjonen av krevende skoledager, mye festing og lite søvn kan faktisk utløse alvorlige psykiske problemer. Hvis du sliter, eller ser andre som gjør det er det hjelp å få. Ring TIPS for råd og hjelp, for deg selv eller andre. Du kan være anonym om du ønsker det.
TIDLIGE TEGN PÅ PSYKISKE PROBLEMER: Isolerer seg • Sover dårlig • Angst • Tristhet • Konsentrasjonsvansker • Forsømmer husabeid, hygiene,jobb eller skole • Ekstremt opptatt av temaer som døden, politikk eller religion • Store humørsvingninger • Tankene høres ut som stemmer • Snakker usammenhengende • Føler seg forfulgt eller styrt av andre
Stavanger Universitetssjukehus
Helse Stavanger HF
Psykiatrisk divisjon
TIPS
TIDLIG OPPDAGELSE OG BEHANDLING AV PSYKOSER
51 51 59 59 hverdager 08.00-15.00, tips-info.com
SØK HJELP SÅ RASKT SOM MULIG, DA ER SJANSEN STØRST FOR Å BLI FRISK

IKKE TELL PÅ KNAPPENE, BRUK DEM!

Siden januar har flere hundre mennesker ringt oss og fått hjelp og råd om psykiske lidelser

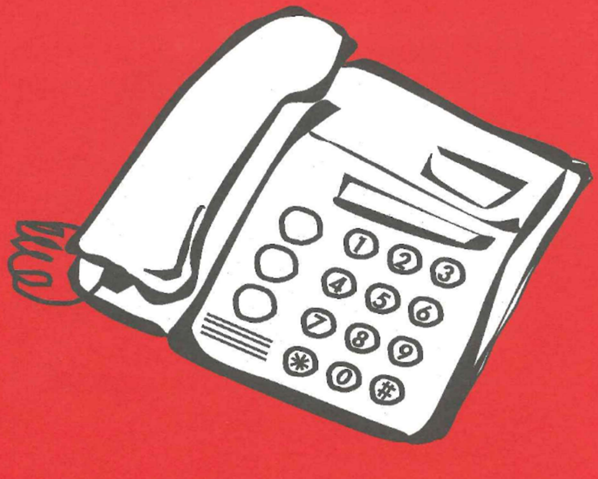

Selv om mistanken om at "noe er galt" er vekket, går det ofte lang tid før mennesker med psykiske lidelser får hjelp. Synd, for jo tidligere hjelpen settes inn, desto større er sjansen for å bli frisk. Målet med TIPS-prosjektet er å korte ned tiden fra de første problemene melder seg til behandlingen starter. Telefonen gjør det mulig å være anonym og man slipper å sette seg i kø hos en lege. Alle som betjener telefonen er erfarne fagfolk som sørger for at du får den hjelpen du behøver.

<u>En av de første</u> telefonene vi fikk var fra en mor som var bekymret for sønnen. Fra å være utadvendt og skoleflink, hadde han lukket seg inne på rommet, skulket skolen, vært mutt og innesluttet. Vi kalte gutten inn til samtale hos oss og fant ut at han ville ha utbytte av å gå til psykolog. Han går nå til ukentlige samtaler med psykolog og tar eksamen sammen med sine medelever.

<u>I februar</u> fikk vi en henvendelse fra en engstelig hybelvert. En av leietakerne gikk stadig oppe om nettene og pratet høyt og usammenhengende med seg selv. Han virket aggressiv når verten forsøkte å snakke med ham. Det var tydeligvis lenge siden han hadde vasket og stelt seg. Hybelen var rotet og luktet vondt. En av våre psykologer besøkte leieboeren sammen med hybelverten. Etter en lengre samtale kom de fram til et behandlingsopplegg i samarbeid med mannens faste lege.

TIPS
TIDLIG OPPDAGELSE OG BEHANDLING AV PSYKOSER

SØR-ROGALAND: 51 67 04 88
NORD-ROGALAND: 52 73 27 00

<u>For noen uker siden</u> fikk vi en telefon fra en ung dame, som fortalte at en eldre kvinnelig kollega plutselig hadde begynt å oppføre seg "unormalt". Kollegaen stilte på kontoret svært oppstemt, kledd som en tyveåring. Etter først å ha flørtet åpenbart med sjefen, skjelte hun ham ut og forsvant i fullt sinne. Hvordan kunne hun hjelpe? Vi ga henne råd om å kontakte kvinnens familie. Hennes bror ringte og rådførte seg med oss. Han fortalte at søsteren gikk på medisiner, men hadde sluttet å ta dem. Vi ordnet med innleggelse, og etter noen døgn på sykehus kunne hun flytte hjem og gå på jobb som vanlig.

<u>TIPS-telefonen er åpen for alle</u>, også leger, lærere, sosionomer og andre som trenger råd eller veiledning. Telefonen er betjent hverdager fra kl. 8.00 - 15.30. Det går også an å legge igjen beskjed på telefonsvareren, så ringer vi tilbake. Ikke tell på knappene - bruk dem !

SØK HJELP SÅ FORT SOM MULIG, DA ER SJANSEN STØRST FOR Å BLI FRISK

Statens Helsetilsyn, Rogaland Fylkeskommune, PsykOpp, Fylkessjukehuset i Haugesund, Rogaland Psykiatriske Sykehus

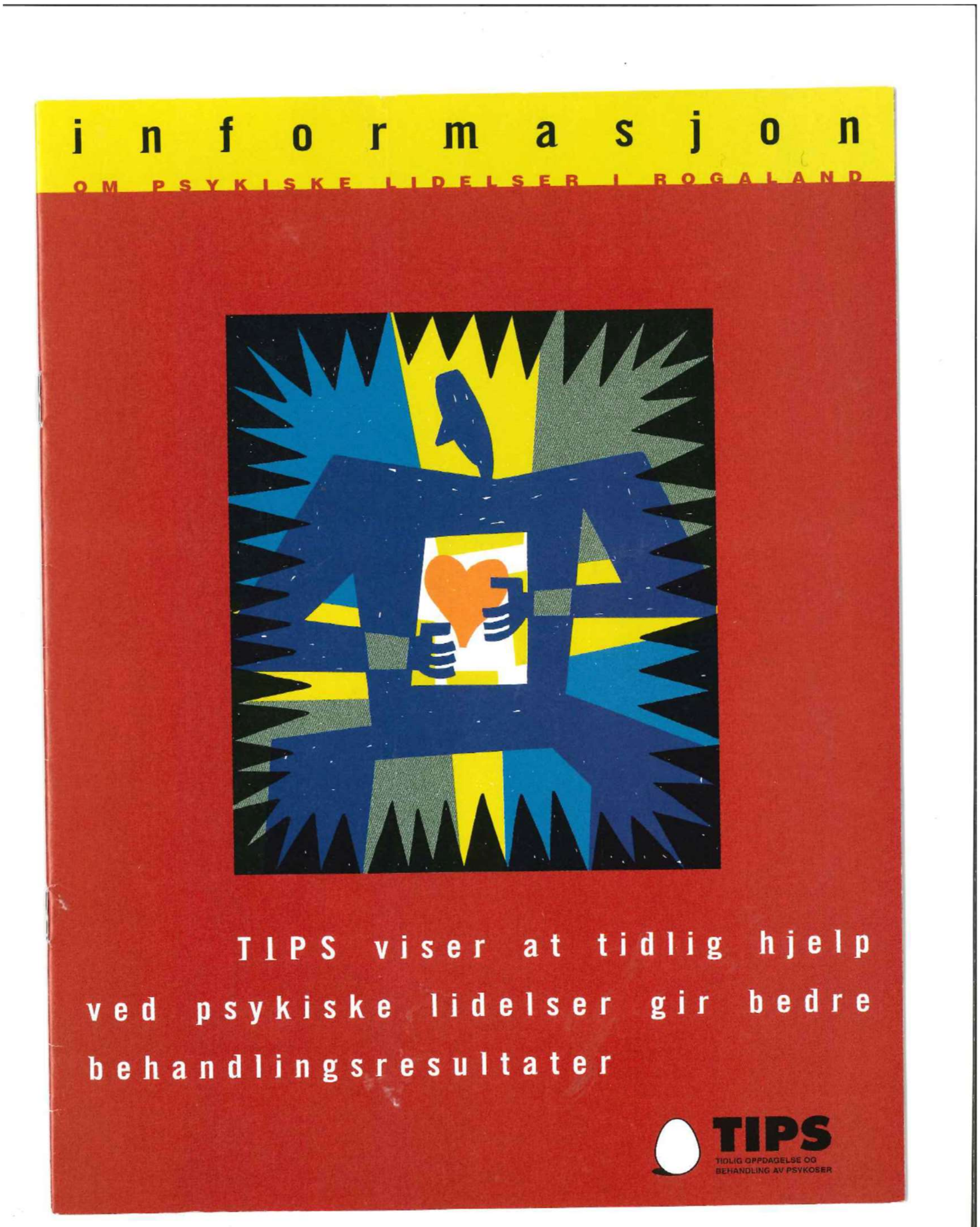

Brosjyre - fremside

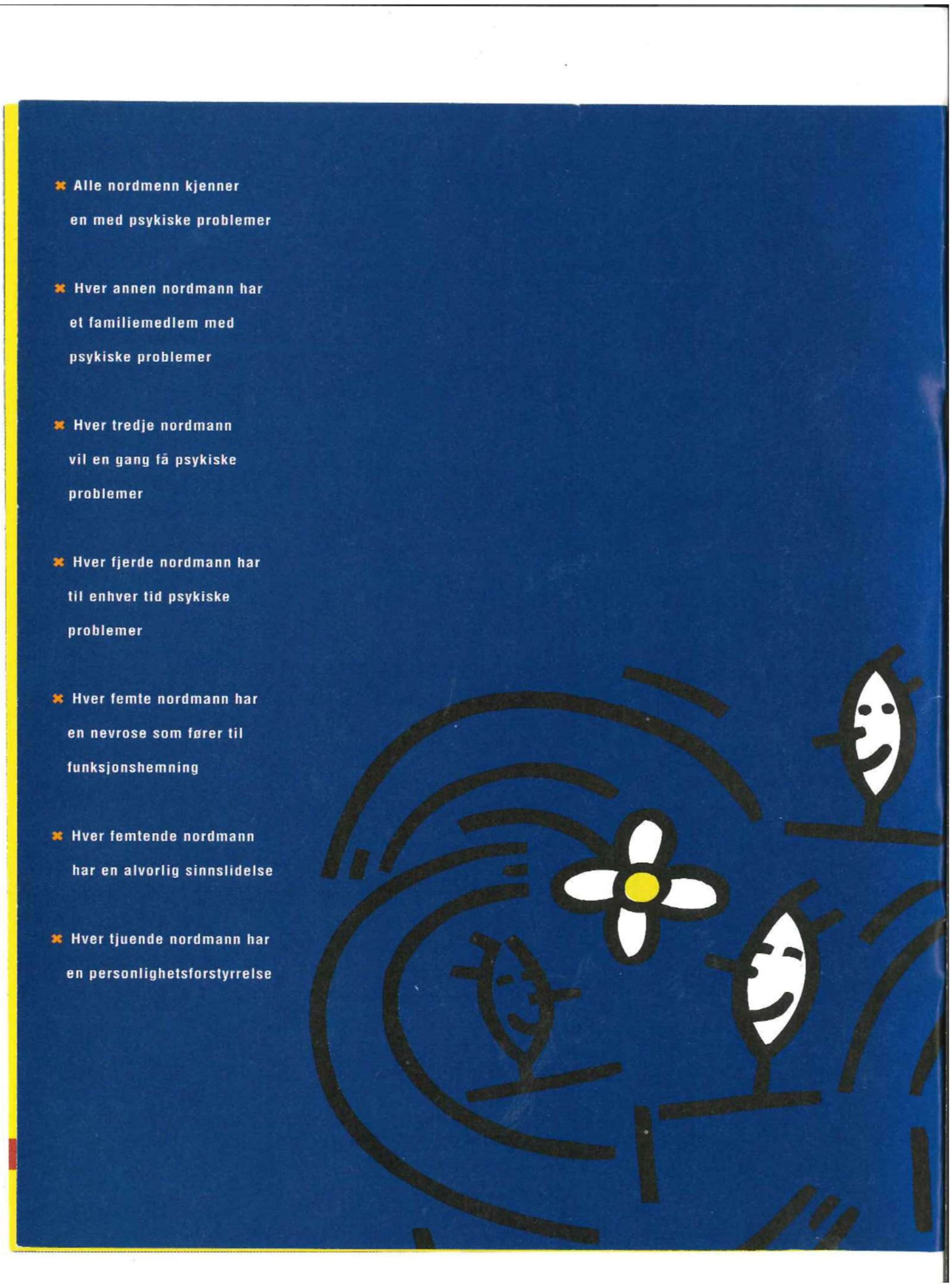

Samme brosjyre som over

Se denne siden sammen med tekst på neste side

psykiske problemer er mer utbredt enn mange tror og behandlingstilbudet er bedre enn mange er klar over

Selv om kunnskapene om psykiske lidelser har blitt bedre de siste årene, vet vi at mange sliter med problemer uten å oppsøke hjelp. Årsakene kan være mange: en tror det går over, en vet ikke hvor man får hjelp, en kvier seg for å oppsøke lege, en synes det er flaut, en vil ikke innrømme for seg selv at en har problemer, en er redd for hva andre vil tenke.
Jo lenger en venter med å ta kontakt, dess mer alvorlige kan problemene bli.

i likhet med andre sykdommer er psykiske lidelser enklere å behandle når de oppdages tidlig

Rogaland er kanskje det fylket som har satset mest på opplysningsarbeid når det gjelder psykiske lidelser, likevel ser vi at mange venter for lenge før de oppsøker hjelp. I 1996 ble TIPS-prosjektet opprettet. Målet med prosjektet er å redusere tiden fra de første symptomene på psykiske lidelser oppstår til behandlingen starter. Pasientene får rask hjelp og tilbys den hjelpen som anses for den beste. I tillegg til utstrakt opplysningsarbeid og skolering av allmennpraktiserende leger, lærere og helsearbeidere har vi opprettet to faggrupper som tar imot henvendelser fra alle som trenger råd eller hjelp. Dette tilbudet ble åpnet januar 1997 og har så langt vist gode resultater.

I løpet av de tre årene TIPS har eksistert er perioden med ubehandlet psykose i Rogaland redusert fra 114 til 26 uker. Tilbakefallsprosenten blant pasientene som følger vårt behandlingsopplegg er redusert til en tredjedel. Vi regner med at ca 20 av de pasientene som i dag bor hjemme, ville blitt innlagt på sykehus uten tidlig oppdagelse og behandling.

Denne brosjyren blir delt ut til alle husstander i Rogaland. Her kan du lese om tidlige tegn på psykiske lidelser, hvordan du kan få rask hjelp og hvilke behandlingstilbud som finnes. Ring oss hvis du trenger råd eller hjelp!

Forts. samme brosjyre

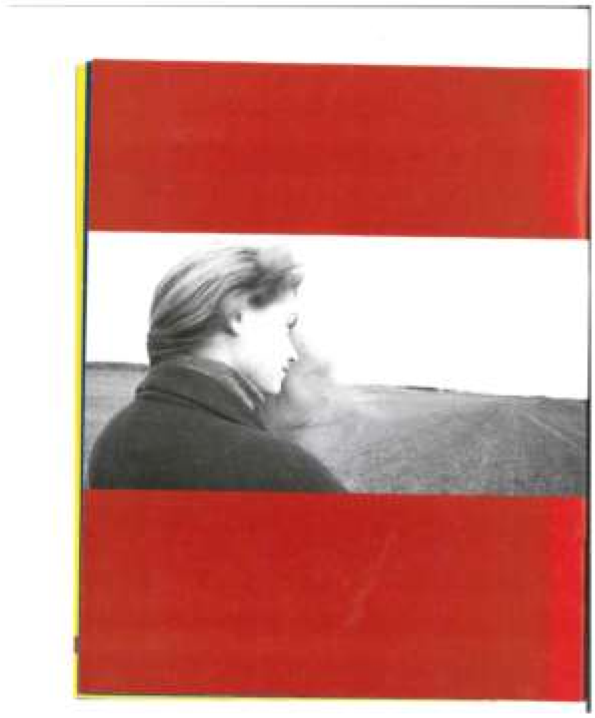

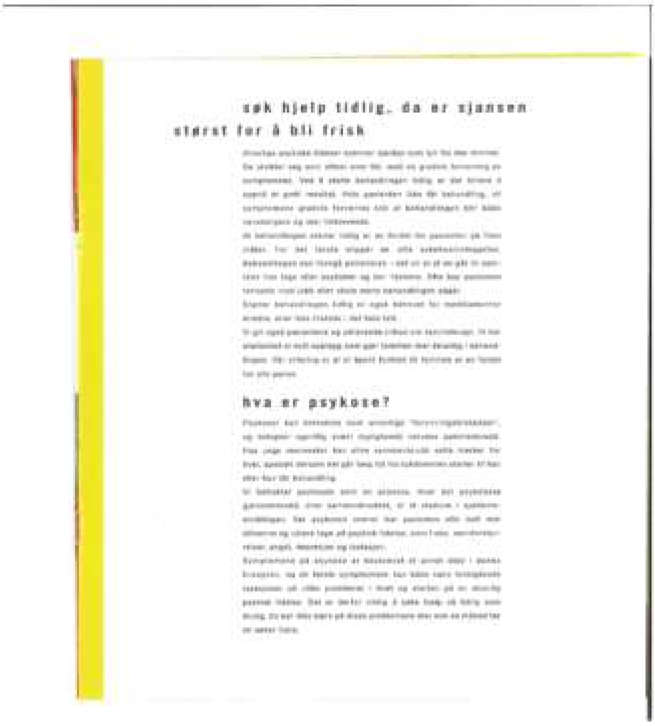

søk hjelp tidlig, da er sjansen størst for å bli frisk

[illegible]

hva er psykose?

[illegible]

Henger sammen med bildet over

Forts. samme brosjyre

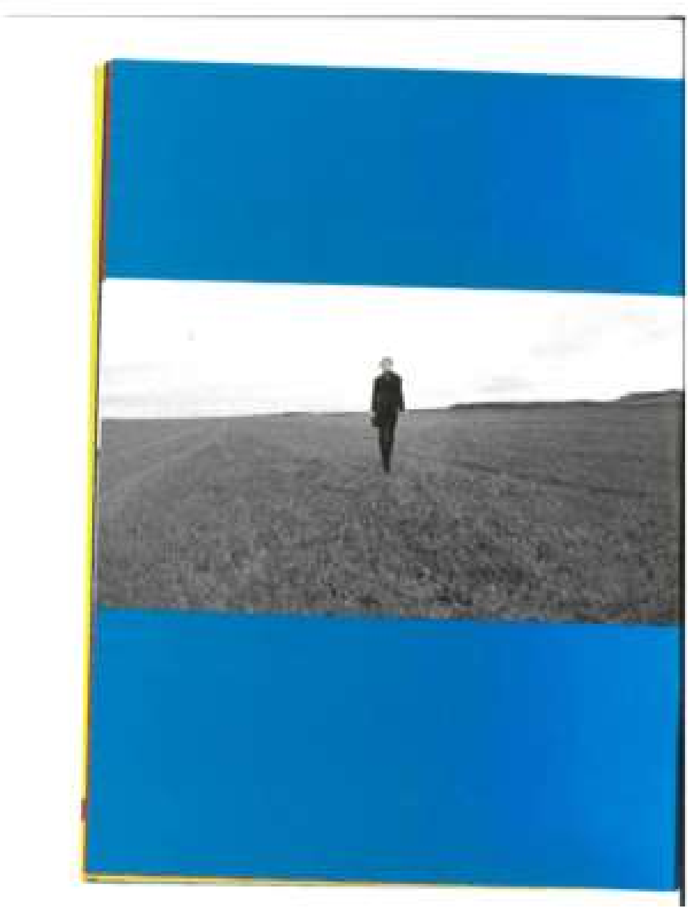

For å forstå hvordan psykiske lidelser oppstår og utvikler seg, er det greit å vite litt om hvordan menneskets psykologiske mekanismer fungerer.

Fra naturens side er vi utstyrt med visse arvelige egenskaper, det som kalles evner og anlegg. I samspill med det miljøet vi vokser opp i blir evner og anlegg formet og vi modnes og blir det enestående individet hver enkelt av oss er.

Som nyfødte skiller vi ikke mellom oss selv og verden - "alt flyter". Følelsene dominerer sinnstilstanden. Vi gråter når vi er våte eller sultne, vi er fornøyde når vi er mette og varme.

I løpet av de første leveårene oppstår bevisstheten om at "jeg" er noe eget, atskilt fra andre. Opplevelsen av omverdenen blir mer nyansert og gradvis utvikles det vi kaller psykologiske mestringsmekanismer. Andre ord for dette er forsvars- eller motstandsmekanismer. Som barn opplever vi ofte verden som "ond eller god". Vi forenkler verden og ser den fra en "enten/eller" synsvinkel. Mye av dette skjer ubevisst.

Etter som årene går modnes mestringsmekanismene. Verden blir mer nyansert. I pubertet og ungdomstid utfordres denne evnen til å skille mellom "meg" og "omgivelsene", og mange opplever dette som en vanskelig periode i livet.

Sammen med følelsene våre, vil de psykologiske mestringsmekanismene bidra til at vi kommer oss "velberget" gjennom livet psykisk sett.

Denne figuren viser at følelsene i utgangspunktet skal være en hjelp på samme måte som f.eks. temperatur- og smertesans hjelper oss fysisk sett. Dersom vi tar på noe varmt, trekker vi oss unna. På samme måten skal en følelse av redsel fortelle oss at vi skal trekke oss unna eller gjøre noe med situasjonen som gjør oss redd. Når vi ikke greier å gjøre noe med situasjonen, blir følelsene en belastning i stedet for en hjelp. Følelsene lammer evnen til å tenke fornuftig og vi går over i en nevrotisk tilstand. Dersom følelsene overmanner oss helt og vi blir forvirret, vil vi komme i grenseområdet til å utvikle en psykose. Våre psykologiske mestringsmekanismer er da overbelastet og utsatt for påkjenninger som er for store til at vi klarer å takle dem. Den endelige forvirringstilstanden, eller psykosen, innebærer at de psykologiske mestringsmekanismene har møtt påkjenninger som har vært for store for dem.

Det vi vet er at det tar svært lang tid fra en person utvikler en slik alvorlig "forvirringstilstand" for første gang, til vedkommende får behandling. I Rogaland, som i landet forøvrig, kan det gå flere år fra de første sykdomstegn viser seg til pasienten får behandling i det psykiske helsevernet.

Vi mener at det er mulig å hindre, mildne eller forsinke utbruddet av en psykose dersom behandlingen starter på et tidligere tidspunkt i sykdomsutviklingen. Vi tror også at fremtidsutsiktene, prognosen, blir mye bedre når behandlingen settes inn tidlig. Selve behandlingen blir også sannsynligvis enklere og mer kortvarig.

Det finnes flere ulike psykiske lidelser, men de første symptomene er ofte felles og det er viktig å ta kontakt med TIPS hvis du har mistanke om at noe er galt.

ANGST

DEPRESJON

MANISK DEPRESSIV PSYKOSE

SCHIZOFRENI

SPISEFORSTYRRELSER

TVANGSLIDELSER

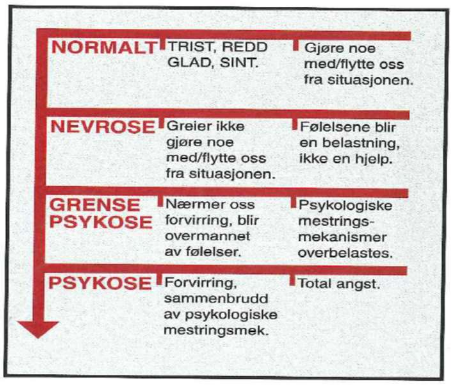

Henger sammen med bildet over

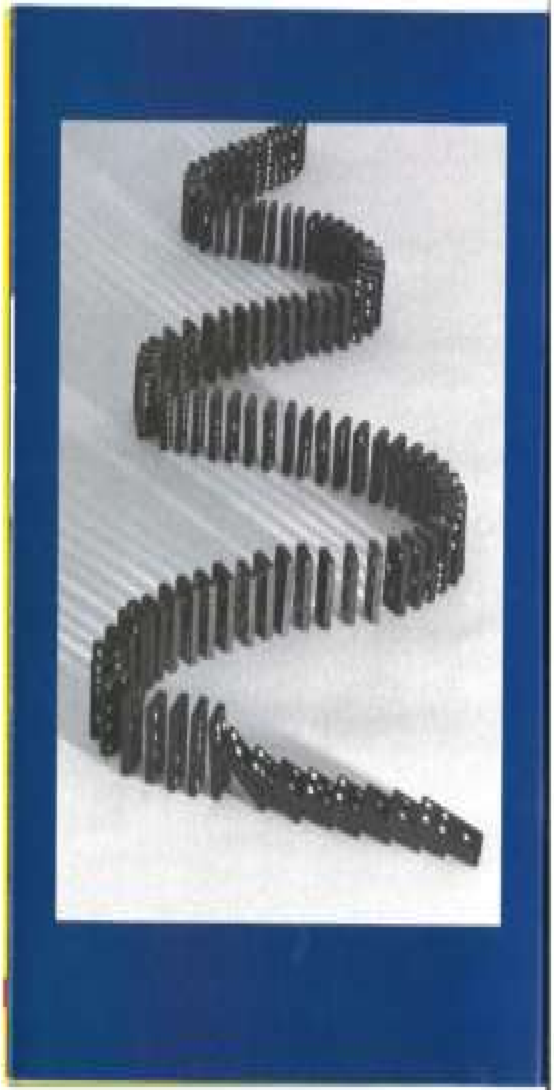

når bør en kontakte lege?

Mange nøler med å kontakte lege selv om de har mistanker om at noe er galt. Grensene mellom det en klarer å mestre selv og problemer en trenger hjelp til å løse er ofte uklare.
Her har vi satt opp to eksempler som kan være til hjelp hvis en lurer på om en skal søke hjelp.

NORMALE REAKSJONER PÅ VANSKELIGE PERIODER I LIVET

Det er mange typer oppførsel som egentlig er normal selv om den kan oppleves som det stikk motsatte av venner, familie og lærere. Vi er forskjellige og kan ha ulike reaksjoner på vanskelige perioder i livet. En person kan være:

FREKK - TANKELØS - LAT - SELVOPPTATT - OPPFARENDE - IRRITABEL KRANGLEVOREN - REBELSK - SJENERT - INNESLUTTET - OVERFØLSOM TAR LETT TIL TÅRENE

Disse måtene å oppføre seg på kan oppstå som normale og kortvarige reaksjoner på stressende hendelser eller problemer som f. eks:

BRUDD PÅ ET NÆRT FORHOLD - STRYK TIL EKSAMEN - DØDSFALL TIL EN SOM STO EN NÆR - FAMILIEKRISER - FLYTTING - FYSISK SYKDOM OG ANDRE TYPER KRISE

Sannsynligvis ingen grunn til alvorlig bekymring. Prøv å gi støtte og oppmuntring i den vanskelige perioden. Som oftest er det ikke nødvendig å søke råd og hjelp hos lege.
Men, hvis denne oppførselen strekker seg over mange uker eller måneder bør du ta kontakt med din faste lege eller ringe TIPS.

ENDRET OPPFØRSEL SOM BØR GI GRUNN TIL BEKYMRING

Når noen du kjenner plutselig oppfører seg annerledes enn de pleier, kan det være grunn til bekymring.

TREKKER SEG TILBAKE FRA FAMILIE OG VENNER · ER REDD FOR Å FORLATE HUSET · SOVER DÅRLIG OG SPISER LITE · ER EKSTREMT OPPTATT AV ET SPESIELT TEMA, SOM F. EKS. DØDEN, POLITIKK ELLER RELIGION · FORSØMMER PERSONLIG HYGIENE · OPPNÅR DÅRLIGERE RESULTATER PÅ SKOLEN · HAR PROBLEMER MED Å KONSENTRERE SEG OG HUSKE TING · SNAKKER OM, ELLER SKRIVER TING, SOM IKKE GIR NOEN MENING · FÅR PANIKK, ER EKSTREMT ENGSTELIG, MERKBART DEPRIMERT ELLER HAR SELVMORDSTANKER · MISTER VARIASJON I HUMØRET · MANGLER UTTRYKK FOR FØLELSER · HAR MARKERTE SVINGNINGER I HUMØRET · HAR UPASSENDE FØLELSESMESSIGE REAKSJONER · HØRER STEMMER SOM INGEN ANDRE KAN HØRE · TROR, UTEN GRUNN, AT ANDRE LAGER KOMPLOTT MOT, SPIONERER PÅ ELLER FØLGER ETTER EN · TROR AT EN BLIR SKADET, ELLER PÅVIRKET TIL Å GJØRE TING MOT ENS VILJE, AV FJERNSYN, DJEVELEN ETC. · TROR AT EN HAR SPESIELLE EVNER · TROR AT TANKENE BLIR PÅVIRKET ELLER AT EN KAN PÅVIRKE TANKENE TIL ANDRE

Siste side i denne brosjyren

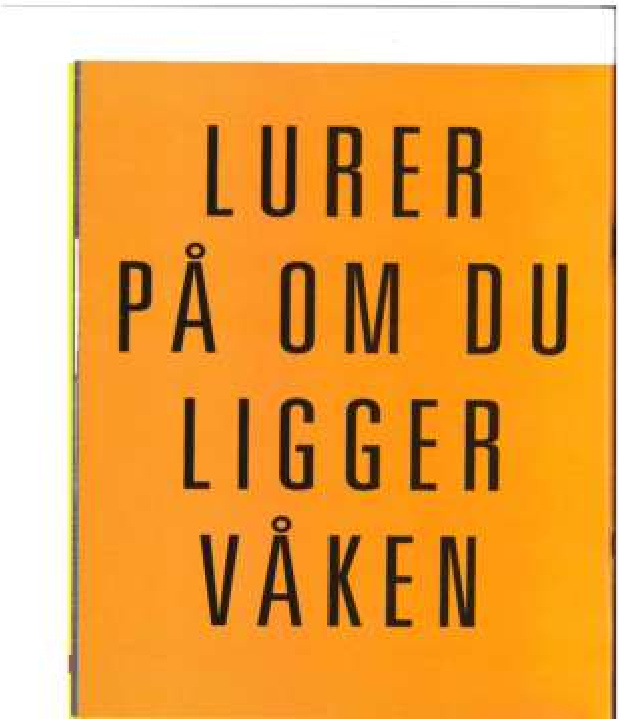

Ny brosjyre

Se sammen med tekst under

TIPS
hjelper deg når du vil hjelpe andre

Muligheten for å ringe og be om råd har vært til stor hjelp for mange. Noen ringer med egne problemer mens andre behøver råd på vegne av andre, som regel familie og venner. TIPS-telefonen er betjent av mennesker med lang og variert erfaring fra psykiatrien. Alle er spesialutdannet og vet hva som bør gjøres når en psykisk lidelse er under utvikling. Vi kjenner de ulike behandlingstilbudene og sørger for at den rette hjelpen blir gitt.
Vi formidler kontakt med lege, psykolog, psykiater, psykiatrisk sykepleier eller andre som kan gi riktig hjelp, raskt.

TIPS
sørger for rask og riktig hjelp

TIPS har kontorer i Sandnes og Haugesund som dekker hele fylket. Alle som bor i Rogaland kan ringe oss.
Telefonen er betjent hverdager fra 8.00 - 15. 30.
Utenom disse tidene kan du legge igjen beskjed på telefonsvarer, så ringer vi tilbake.
Gjennom telefonsamtale danner vi oss et bilde av problemene og gir råd om hva som bør gjøres videre. Hvis det er nødvendig kommer vi hjem og fortsetter samtalen der. Uansett sørger vi for at alle som har behov for hjelp får et tilbud om videre samtaler, utredning eller behandling i nærheten av der vedkommende bor.

TIPS SØR-ROGALAND

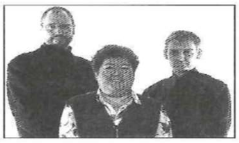

Svein Aspøy
Klinisk sosionom
Marthe Horneland
Psykiater
Inge Joa
Psykiatrisk sykepleier

TIPS NORD-ROGALAND

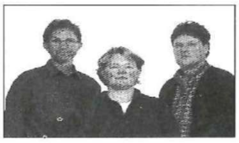

Sigurd Mardal
Psykolog
Kari Lie
Psykiatrisk sykepleier
Rune Kvebæk
Psykiatrisk sykepleier

Margrethe Tytlandsvik overtar for Svein Aspøy høsten 1999.

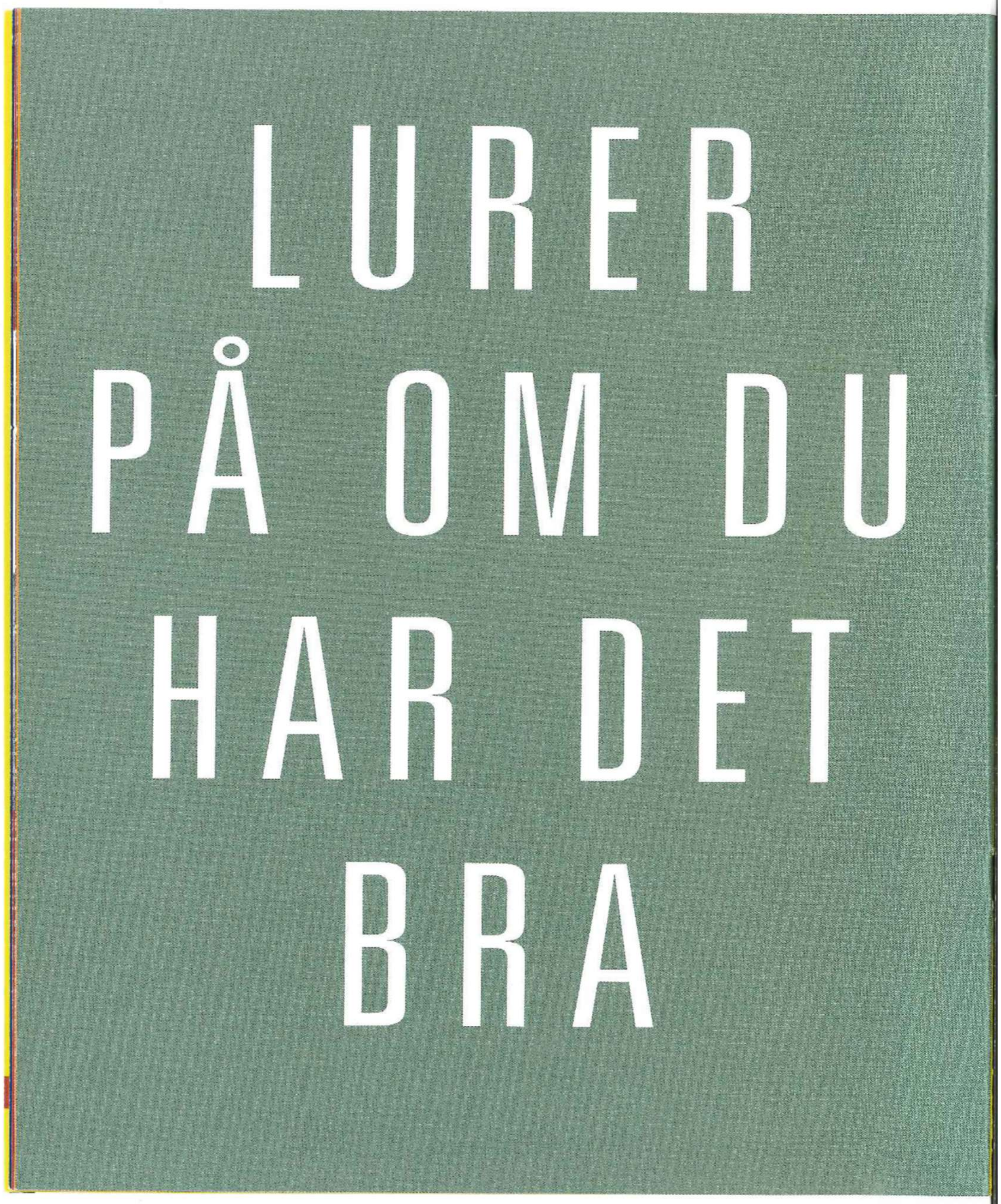
LURER
PÅ OM DU
HAR DET
BRA

Sammen med tekst under

TIPS
bidrar til å gjøre behandlingen bedre

TIPS-prosjektet søker å utvikle et best mulig behandlingstilbud. Det finnes naturligvis ikke en behandlingsform som passer for alle og den må derfor tilpasses den enkelte pasient. En kan likevel si at den behandlingen vi tilbyr består av tre deler: samtale med lege, psykiater eller psykolog, medisinsk behandling og familiearbeid.

Vi baserer oss på å bruke et minimum av medisiner. Den viktigste grunnen til å bruke medisiner er for å fjerne plagsomme symptomer, som f.eks. hallusinasjoner eller søvnproblemer. Dette gjør det lettere for pasienten å mestre dagliglivet, og øker effekten av samtaleterapi og familiearbeid. Samtaleterapi har som mål å gjøre pasienten i stand til å mestre sine egne problemer og å bidra til at pasienten kan bo hjemme og fungere i jobb eller skole.

FAMILIEARBEID

Som et ledd i å tilby et best mulig behandlingstilbud har TIPS satset på å utvikle samarbeidet med pasientens familie. Familieterapi har vært praktisert lenge, i ulike former og med varierende resultater. I løpet av de siste ti-årene har stadig flere pasienter flyttet ut fra institusjonene og hjem. Dette har ført til at familien må ta et større ansvar og at de har fått større behov for hjelp til å mestre dette.

Vårt utgangspunkt er at det ikke er familiens feil at noen blir syke, men at familien trenger hjelp og støtte for å mestre dagliglivet med pasienten på en best mulig måte.

FAMILIEARBEID I TIPS

Et av elementene i behandingsopplegget for psykosepasienter er kunnskapsbasert familiearbeid. De som får dette tilbudet er pasienten og deres nære pårørende.

Med de nære pårørende menes familiemedlemmer som vil være viktige støttepersoner for pasienten i det videre rehabiliteringsarbeidet. Det er ingen betingelse at de må bo under samme tak.

Denne kunnskapsbaserte modellen kan vise til overbevisende resultater med hensyn til å forebygge tilbakefall hos pasientene. Tilbakefallsrisikoen etter et år er blitt redusert til en tredjedel sammenlignet med poliklinisk, medisinsk behandling. Pårørende som har deltatt i disse gruppene opplever å bli trukket inn i behandlingen på en måte de setter pris på.

Fem pasienter og deres nære pårørende blir invitert til å delta i en gruppe som blir ledet av to gruppeledere.

Gruppen får undervisning om psykoser og blir deretter hjulpet til å flette forståelse inn i daglig samvær med pasienten. Undervisningen har som tema: kriseteori, psykoseforståelse, stress/sårbarhetsmodellen, tidlige tegn på sykdom, varselsignaler, behandling, pasientens behov, familiens situasjon og hva familien kan gjøre for å være til hjelp og støtte.

Gruppene møtes på ettermiddags/kveldstid over en to års periode og samlingen varer nitti minutter hver gang. Møtetiden er strukturert rundt å løse problemer som oppstår i det daglige samværet mellom pasienten og pårørende.

Gjennom å utvide nettverket rundt hver familie på en slik måte, oppnås betydelige tilleggseffekter i forhold til å tilby familier hjelp enkeltvis.

Skyld og skamfølelse knyttet opp til sykdommen blir redusert. I gruppene får deltakerne også anledning til å høste av andres erfaringer og gruppen tilbyr et fellesskap.

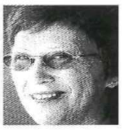

Anne Lise Øxnevad ansvarlig for familiearbeid

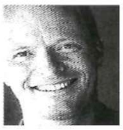

Trond Grønnestad ansvarlig for familiearbeid

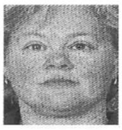

Kari Lie ansvarlig for familiearbeid

trenger du noen å snakke med?

Selv om TIPS-telefonen er åpen for alle i Rogaland når det gjelder råd og veiledning, er det først og fremst pasienter med begynnende psykose som blir tilbudt et fullstendig behandlingstilbud i prosjektet. Heldigvis finnes det en rekke organisasjoner som arbeider med og for pasienter og pårørende. Her er noen kontaktadresser som kan være til hjelp.

MENTAL HELSE ROGALAND
Madlaveien 13
4008 Stavanger
tlf: 51 56 54 45

SENTER FOR SPISEFORSTYRRELSER
Madlaveien 13
4008 Stavanger
tlf: 51 56 54 10

LANDSFORENINGEN FOR PÅRØRENDE
INNEN PSYKIATRI ROGALAND
Breigata 21
4006 Stavanger
tlf: 51 89 03 70

PÅRØRENDESENTERET
Vaisenhusgt. 39 b
4012 Stavanger
tlf: 51 53 11 11

LIVSKRISEHJELPEN
tlf: 51 51 02 02

VIL DU VITE MER?

Psykiatrisk Opplysningsfond har en rekke gode, lettleste og informative trykksaker om psykiatri. Her er et lite utvalg, men det finnes flere.

PSYKIATRISK OPPLYSNINGSFOND
Breigata 21, 4006 Stavanger,
tlf: 51 89 03 70, fax: 51 89 10 53
www.psykopp.no

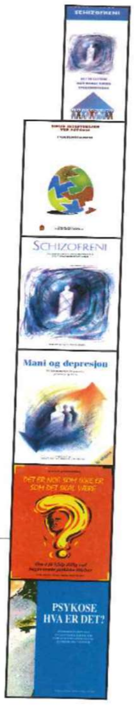

Se sammen med neste side

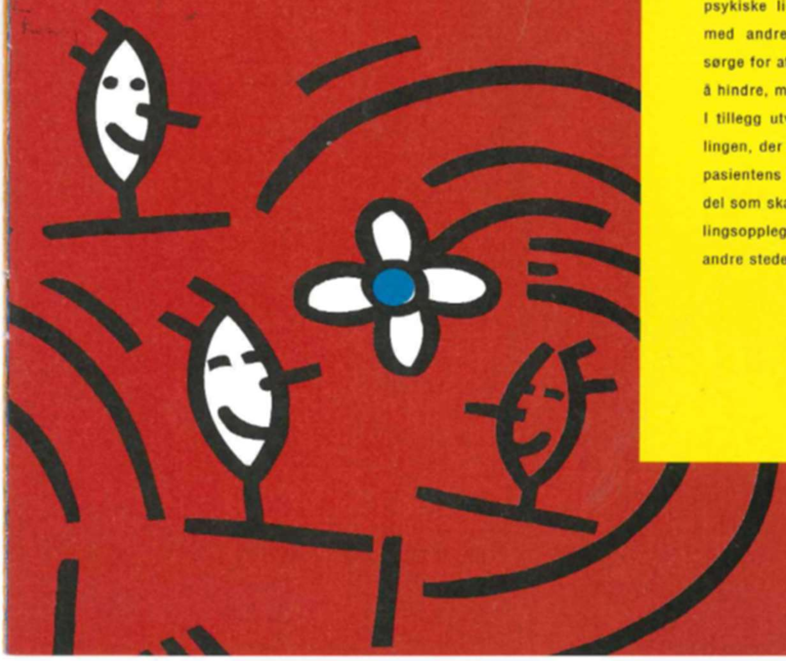

psykiatrien i endring

De siste ti-årenes reformer innen psykisk helsevern har fått vidtrekkende konsekvenser for mennesker med psykiske lidelser. Ansvaret for pasientene er overført fra stat til fylke og kommune. En rekke sentrale institusjoner er lagt ned, pasienter har flyttet til nye institusjoner nær hjemstedet, eller de har flyttet hjem.

Nye behandlingstilbud er utviklet, der særlig ett er framtredende: poliklinisk behandling. Det betyr ganske enkelt at pasientene bor hjemme og kommer til sykehuset for behandling.

Reformen kan ses på som en endring av måten å organisere behandlingen på, men det skjer også nye ting innen selve behandlingen. En prøver å utvikle bedre behandlingstilbud som øker livskvaliteten for både pasienten og de pårørende.

TIPS er et eksempel på dette. TIPS har som mål å oppdage og starte behandling av alvorlige psykiske lidelser (psykoser) tidligere. En skal med andre ord stille diagnosen tidligere og sørge for at behandlingen starter raskt. Målet er å hindre, mildne eller forsinke sykdommen.

I tillegg utvikler TIPS andre sider ved behandlingen, der det bl.a. legges stor vekt på å støtte pasientens familie. TIPS har også en forskningsdel som skal sørge for at forbedringer i behandlingsopplegget kan dokumenteres og benyttes andre steder.

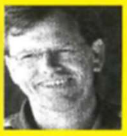

Jan Olav Johannessen
Sjefslege, Rogaland
Psykiatriske Sjukehus

FULL DISTRIBUSJON

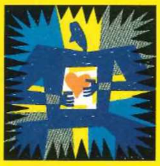

HAAVE REKLAMEBYRÅ • FOTO: SIGBJØRN SIGBJØRNSEN OG MORTEN HEISELBERG • ILL.: LASSE SKARBØVIK

søk hjelp
så fort som mulig,
da er sjansen størst for å bli frisk

Det er et utbredt problem at alvorlige psykiske problemer ikke oppdages og behandles før etter lang tids sykdom. Med grunnlag i denne kjennsgjerningen forventer vi at behandling som starter tidligere i sykdomsforløpet vil gi bedre effekt. Vi mener det er mulig å hindre, lindre eller forsinke utbruddet av alvorlige sinnslidelser dersom behandlingen starter tidlig nok.

Dette er utgangspunktet for alle våre aktiviteter.

I tillegg til opplysningsarbeid har vi gjort det mulig for alle som ønsker råd og hjelp på vegne av seg selv eller andre å ta direkte kontakt med oss på telefon.

Alle i Rogaland kan ringe og snakke med våre fagfolk.

TIPS
TIDLIG OPPDAGELSE OG
BEHANDLING AV PSYKOSER

SØR-ROGALAND 51 67 04 88 | NORD-ROGALAND 52 73 27 00

www.tips-info.com

Siste side brosjyre

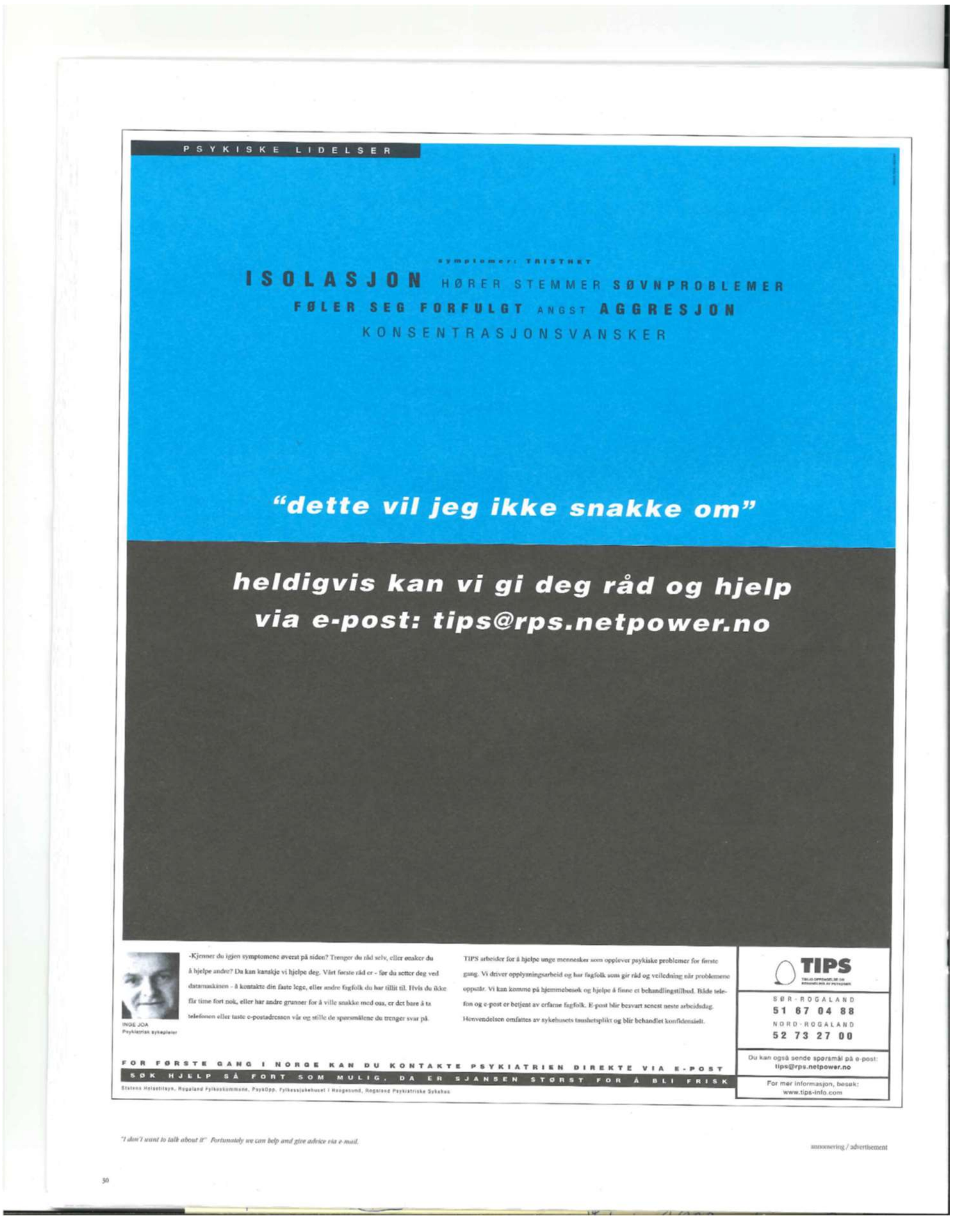
PSYKISKE LIDELSER
symptomer: TRISTHET
ISOLASJON HØRER STEMMER SØVNPROBLEMER
FØLER SEG FORFULGT ANGST AGGRESJON
KONSENTRASJONSVANSKER
"dette vil jeg ikke snakke om"
heldigvis kan vi gi deg råd og hjelp
via e-post: tips@rps.netpower.no
-Kjenner du igjen symptomene øverst på siden? Trenger du råd selv, eller ønsker du å hjelpe andre? Da kan kanskje vi hjelpe deg. Vårt første råd er - før du setter deg ved datamaskinen - å kontakte din faste lege, eller andre fagfolk du har tillit til. Hvis du ikke får time fort nok, eller har andre grunner for å ville snakke med oss, er det bare å ta telefonen eller taste e-postadressen vår og stille de spørsmålene du trenger svar på.
INGE JOA
Psykiatrisk sykepleier
TIPS arbeider for å hjelpe unge mennesker som opplever psykiske problemer for første gang. Vi driver opplysningsarbeid og har fagfolk som gir råd og veiledning når problemene oppstår. Vi kan komme på hjemmebesøk og hjelpe å finne et behandlingstilbud. Både telefon og e-post er betjent av erfarne fagfolk. E-post blir besvart senest neste arbeidsdag. Henvendelsen omfattes av sykehusets taushetsplikt og blir behandlet konfidensielt.
TIPS
SØR-ROGALAND
51 67 04 88
NORD-ROGALAND
52 73 27 00
Du kan også sende spørsmål på e-post:
tips@rps.netpower.no
For mer informasjon, besøk:
www.tips-info.com
FOR FØRSTE GANG I NORGE KAN DU KONTAKTE PSYKIATRIEN DIREKTE VIA E-POST
SØK HJELP SÅ FORT SOM MULIG, DA ER SJANSEN STØRST FOR Å BLI FRISK
Statens Helsetilsyn, Rogaland Fylkeskommune, PsykOpp, Fylkessjukehuset i Haugesund, Rogaland Psykiatriske Sykehus
"I don't want to talk about it" Fortunately we can help and give advice via e-mail.
annonsering / advertisement
30

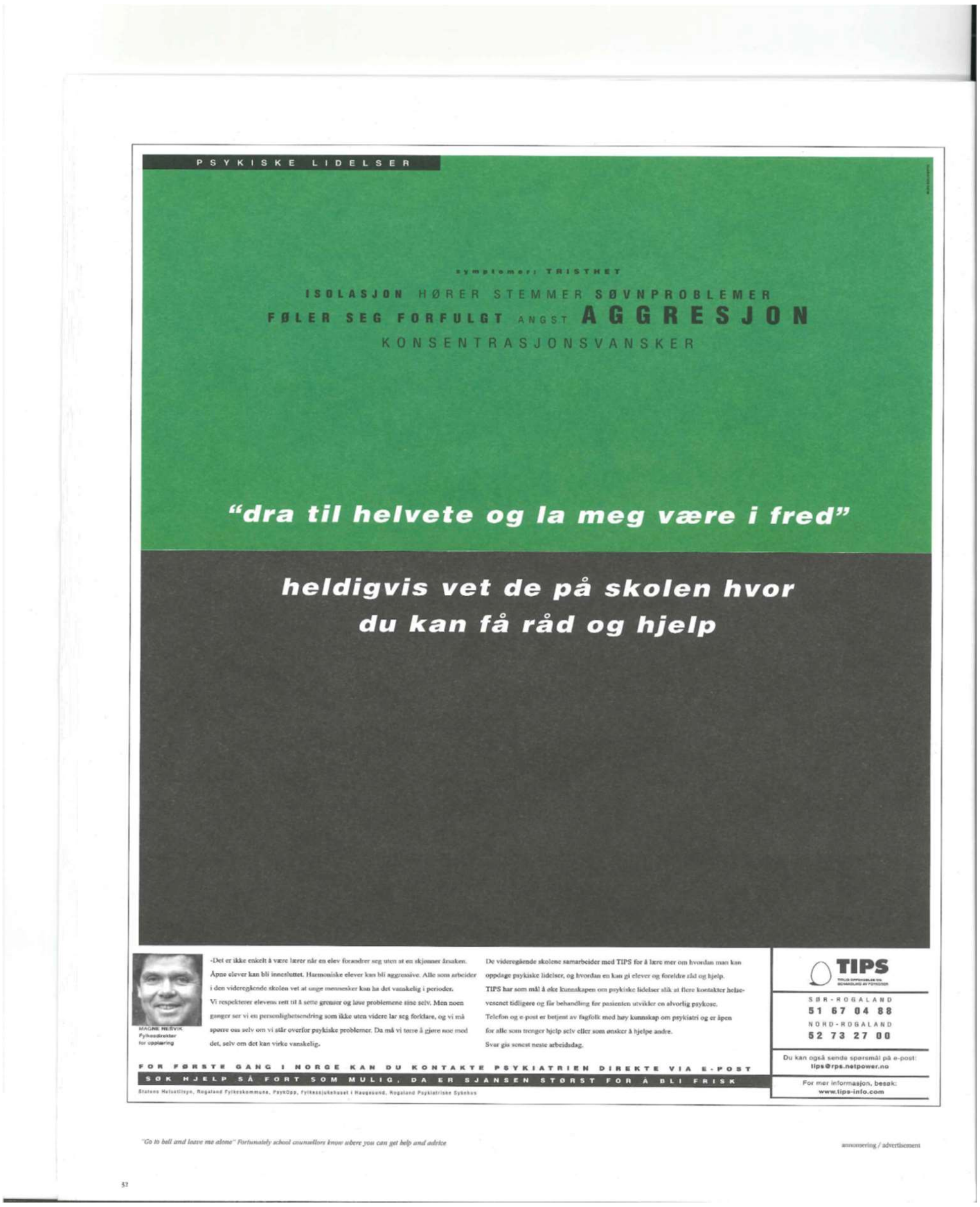

PSYKISKE LIDELSER
symptomer: TRISTHET
ISOLASJON HØRER STEMMER SØVNPROBLEMER
FØLER SEG FORFULGT ANGST AGGRESJON
KONSENTRASJONSVANSKER
"dra til helvete og la meg være i fred"
heldigvis vet de på skolen hvor du kan få råd og hjelp
MAGNE NESVIK
Fylkesdirektør for opplæring
-Det er ikke enkelt å være lærer når en elev forandrer seg uten at en skjønner årsaken. Åpne elever kan bli innesluttet. Harmoniske elever kan bli aggressive. Alle som arbeider i den videregående skolen vet at unge mennesker kan ha det vanskelig i perioder. Vi respekterer elevens rett til å sette grenser og løse problemene sine selv. Men noen ganger ser vi en personlighetsendring som ikke uten videre lar seg forklare, og vi må spørre oss selv om vi står overfor psykiske problemer. Da må vi tørre å gjøre noe med det, selv om det kan virke vanskelig.
De videregående skolene samarbeider med TIPS for å lære mer om hvordan man kan oppdage psykiske lidelser, og hvordan en kan gi elever og foreldre råd og hjelp.
TIPS har som mål å øke kunnskapen om psykiske lidelser slik at flere kontakter helsevesenet tidligere og får behandling før pasienten utvikler en alvorlig psykose.
Telefon og e-post er betjent av fagfolk med høy kunnskap om psykiatri og er åpen for alle som trenger hjelp selv eller som ønsker å hjelpe andre.
Svar gis senest neste arbeidsdag.
FOR FØRSTE GANG I NORGE KAN DU KONTAKTE PSYKIATRIEN DIREKTE VIA E-POST
SØK HJELP SÅ FORT SOM MULIG, DA ER SJANSEN STØRST FOR Å BLI FRISK
Statens Helsetilsyn, Rogaland Fylkeskommune, PsykOpp, Fylkessjukehuset i Haugesund, Rogaland Psykiatriske Sykehus
TIPS
SØR-ROGALAND
51 67 04 88
NORD-ROGALAND
52 73 27 00
Du kan også sende spørsmål på e-post:
tips@rps.netpower.no
For mer informasjon, besøk:
www.tips-info.com
"Go to hell and leave me alone" Fortunately school counsellors know where you can get help and advice
annonsering / advertisement

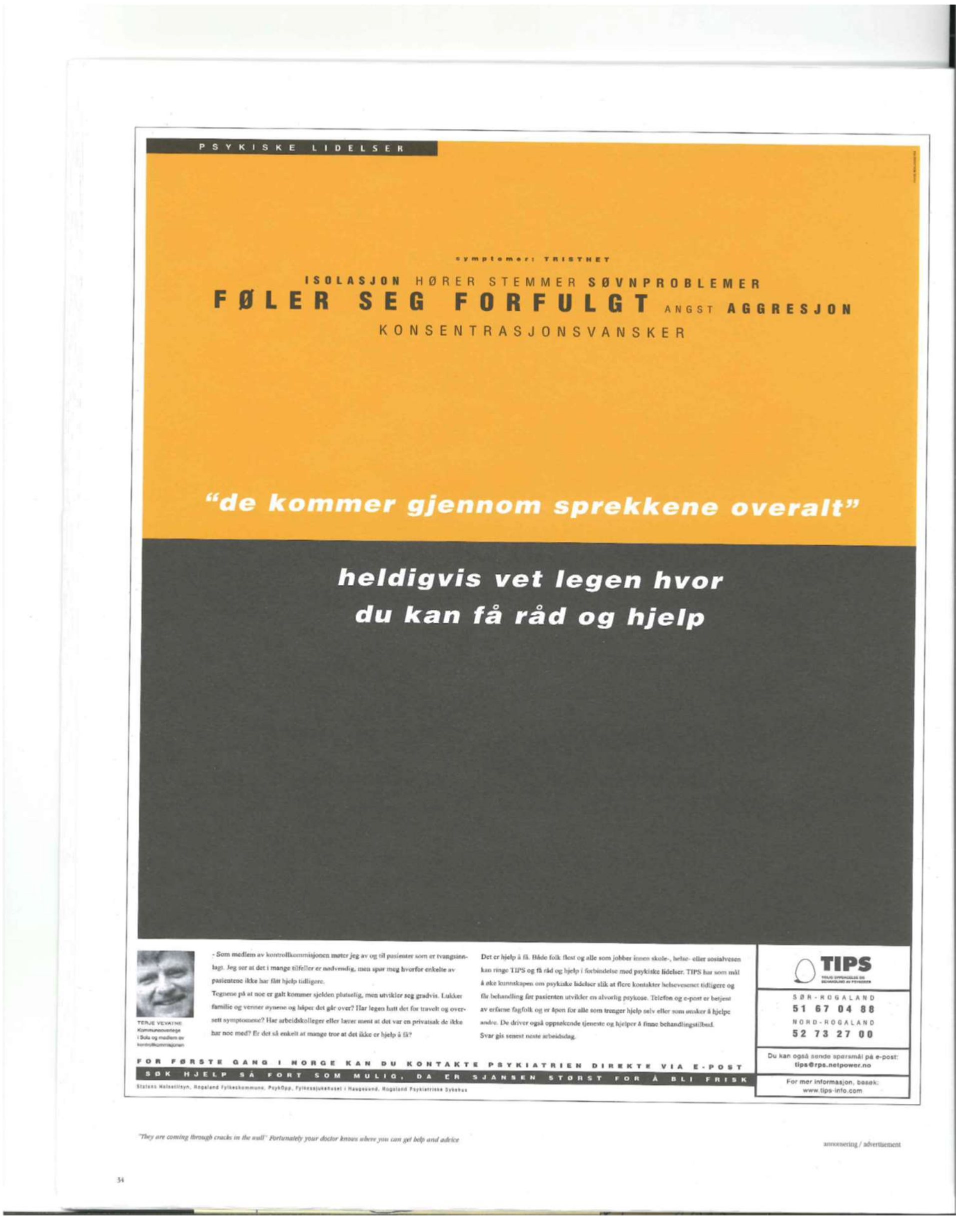
PSYKISKE LIDELSER
symptomer: TRISTHET
ISOLASJON HØRER STEMMER SØVNPROBLEMER
FØLER SEG FORFULGT ANGST AGGRESJON
KONSENTRASJONSVANSKER
"de kommer gjennom sprekkene overalt"
heldigvis vet legen hvor
du kan få råd og hjelp
- Som medlem av kontrollkommisjonen møter jeg av og til pasienter som er tvangsinnlagt. Jeg ser at det i mange tilfeller er nødvendig, men spør meg hvorfor enkelte av pasientene ikke har fått hjelp tidligere.
Tegnene på at noe er galt kommer sjelden plutselig, men utvikler seg gradvis. Lukker familie og venner øynene og håper det går over? Har legen hatt det for travelt og oversett symptomene? Har arbeidskolleger eller lærer ment at det var en privatsak de ikke har noe med? Er det så enkelt at mange tror at det ikke er hjelp å få?
TERJE VEVATNE
Kommuneoverlege i Sola og medlem av kontrollkommisjonen
Det er hjelp å få. Både folk flest og alle som jobber innen skole-, helse- eller sosialvesen kan ringe TIPS og få råd og hjelp i forbindelse med psykiske lidelser. TIPS har som mål å øke kunnskapen om psykiske lidelser slik at flere kontakter helsevesenet tidligere og får behandling før pasienten utvikler en alvorlig psykose. Telefon og e-post er betjent av erfarne fagfolk og er åpen for alle som trenger hjelp selv eller som ønsker å hjelpe andre. De driver også oppsøkende tjeneste og hjelper å finne behandlingstilbud.
Svar gis senest neste arbeidsdag.
TIPS
SØR-ROGALAND
51 67 04 88
NORD-ROGALAND
52 73 27 00
Du kan også sende spørsmål på e-post:
tips@rps.netpower.no
For mer informasjon, besøk:
www.tips-info.com
FOR FØRSTE GANG I NORGE KAN DU KONTAKTE PSYKIATRIEN DIREKTE VIA E-POST
SØK HJELP SÅ FORT SOM MULIG, DA ER SJANSEN STØRST FOR Å BLI FRISK
Statens Helsetilsyn, Rogaland Fylkeskommune, PsykOpp, Fylkessjukehuset i Haugesund, Rogaland Psykiatriske Sykehus
"They are coming through cracks in the wall" Fortunately your doctor knows where you can get help and advice
annonsering / advertisement
34

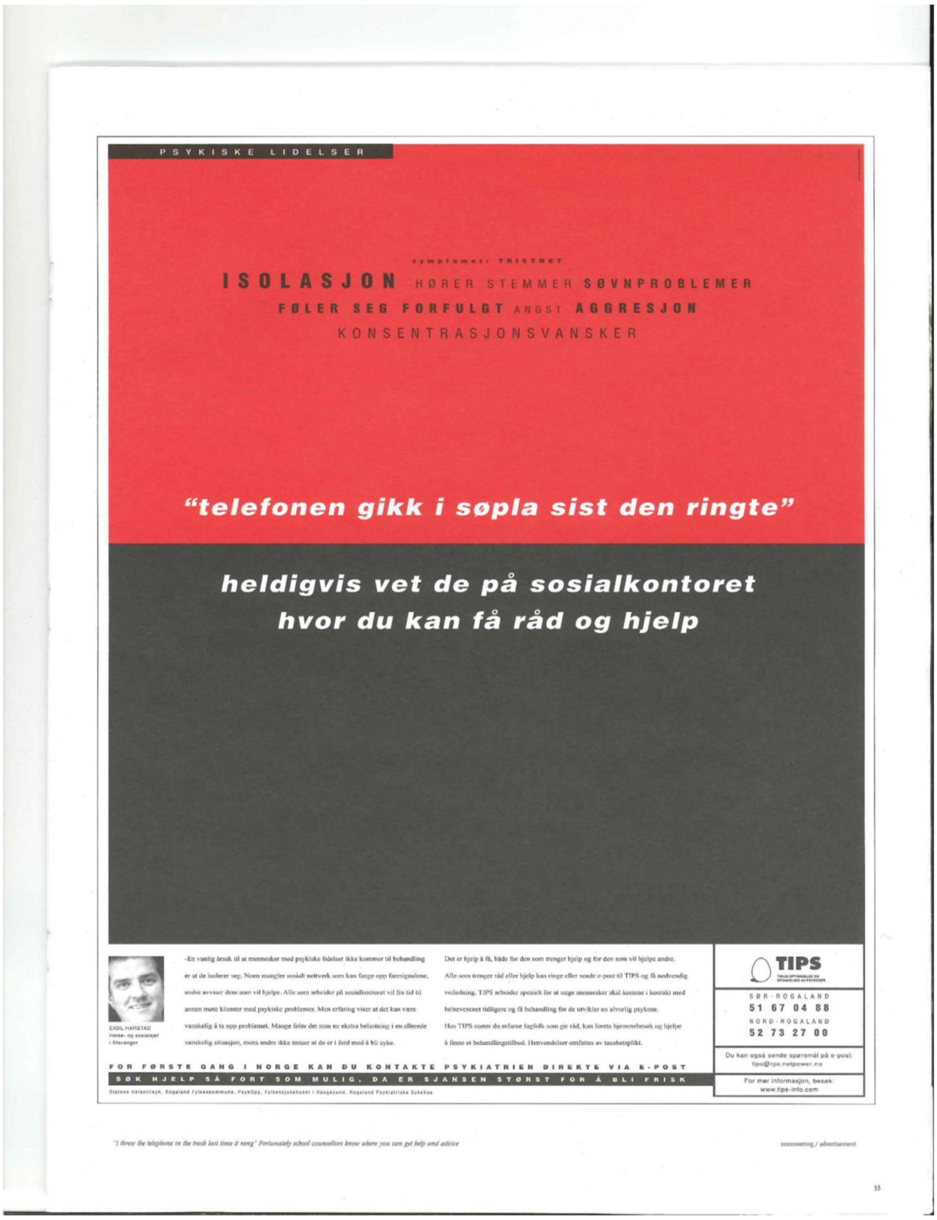

PSYKISKE LIDELSER

symptomer: TRISTHET

ISOLASJON HØRER STEMMER SØVNPROBLEMER

FØLER SEG FORFULGT ANGST AGGRESJON

KONSENTRASJONSVANSKER

"telefonen gikk i søpla sist den ringte"

heldigvis vet de på sosialkontoret hvor du kan få råd og hjelp

EIGIL HARSTAD
Helse- og sosialsjef
i Stavanger

-En vanlig årsak til at mennesker med psykiske lidelser ikke kommer til behandling er at de isolerer seg. Noen mangler sosialt nettverk som kan fange opp faresignalene, andre avviser dem som vil hjelpe. Alle som arbeider på sosialkontoret vil fra tid til annen møte klienter med psykiske problemer. Men erfaring viser at det kan være vanskelig å ta opp problemet. Mange føler det som en ekstra belastning i en allerede vanskelig situasjon, mens andre ikke innser at de er i ferd med å bli syke.

Det er hjelp å få, både for den som trenger hjelp og for den som vil hjelpe andre. Alle som trenger råd eller hjelp kan ringe eller sende e-post til TIPS og få nødvendig veiledning. TIPS arbeider spesielt for at unge mennesker skal komme i kontakt med helsevesenet tidligere og få behandling før de utvikler en alvorlig psykose. Hos TIPS møter du erfarne fagfolk som gir råd, kan foreta hjemmebesøk og hjelpe å finne et behandlingstilbud. Henvendelser omfattes av taushetsplikt.

FOR FØRSTE GANG I NORGE KAN DU KONTAKTE PSYKIATRIEN DIREKTE VIA E-POST

SØK HJELP SÅ FORT SOM MULIG, DA ER SJANSEN STØRST FOR Å BLI FRISK

Statens Helsetilsyn, Rogaland Fylkeskommune, PsykOpp, Fylkessjukehuset i Haugesund, Rogaland Psykiatriske Sykehus

TIPS

SØR-ROGALAND
51 67 04 88
NORD-ROGALAND
52 73 27 00

Du kan også sende spørsmål på e-post:
tips@rps.netpower.no

For mer informasjon, besøk:
www.tips-info.com

"I threw the telephone in the trash last time it rang" Fortunately school counsellors know where you can get help and advice

annonsering / advertisement

33

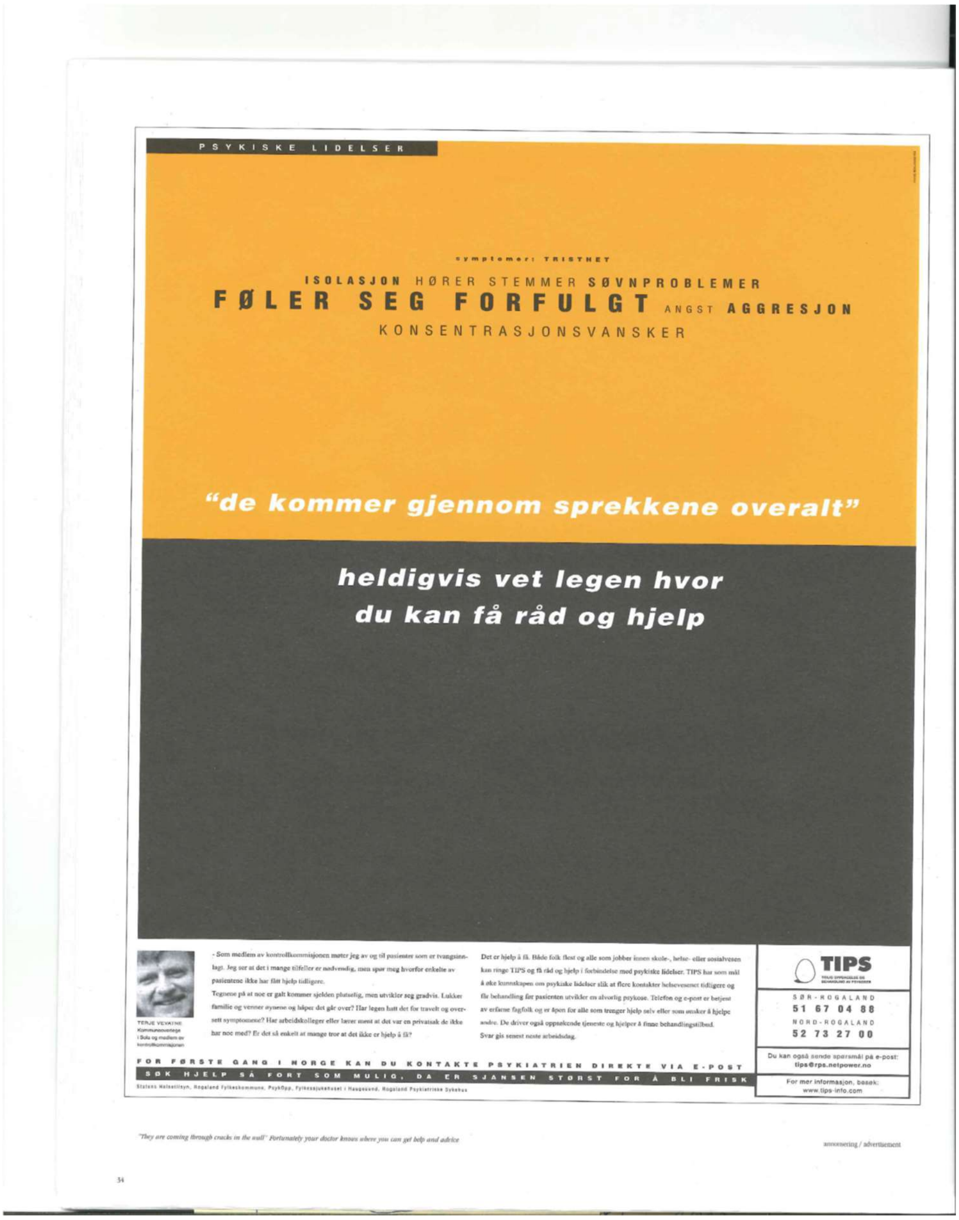

PSYKISKE LIDELSER
symptomer: TRISTHET
ISOLASJON HØRER STEMMER SØVNPROBLEMER
FØLER SEG FORFULGT ANGST AGGRESJON
KONSENTRASJONSVANSKER
"de kommer gjennom sprekkene overalt"
heldigvis vet legen hvor du kan få råd og hjelp
- Som medlem av kontrollkommisjonen møter jeg av og til pasienter som er tvangsinnlagt. Jeg ser at det i mange tilfeller er nødvendig, men spør meg hvorfor enkelte av pasientene ikke har fått hjelp tidligere.
Tegnene på at noe er galt kommer sjelden plutselig, men utvikler seg gradvis. Lukker familie og venner øynene og håper det går over? Har legen hatt det for travelt og oversett symptomene? Har arbeidskolleger eller lærer ment at det var en privatsak de ikke har noe med? Er det så enkelt at mange tror at det ikke er hjelp å få?
TERJE VEVATNE
Kommuneoverlege i Sola og medlem av kontrollkommisjonen
Det er hjelp å få. Både folk flest og alle som jobber innen skole-, helse- eller sosialvesen kan ringe TIPS og få råd og hjelp i forbindelse med psykiske lidelser. TIPS har som mål å øke kunnskapen om psykiske lidelser slik at flere kontakter helsevesenet tidligere og får behandling før pasienten utvikler en alvorlig psykose. Telefon og e-post er betjent av erfarne fagfolk og er åpen for alle som trenger hjelp selv eller som ønsker å hjelpe andre. De driver også oppsøkende tjeneste og hjelper å finne behandlingstilbud.
Svar gis senest neste arbeidsdag.
TIPS
SØR-ROGALAND
51 67 04 88
NORD-ROGALAND
52 73 27 00
Du kan også sende spørsmål på e-post:
tips@rps.netpower.no
For mer informasjon, besøk:
www.tips-info.com
FOR FØRSTE GANG I NORGE KAN DU KONTAKTE PSYKIATRIEN DIREKTE VIA E-POST
SØK HJELP SÅ FORT SOM MULIG, DA ER SJANSEN STØRST FOR Å BLI FRISK
Statens Helsetilsyn, Rogaland Fylkeskommune, PsykOpp, Fylkessjukehuset i Haugesund, Rogaland Psykiatriske Sykehus
"They are coming through cracks in the wall" Fortunately your doctor knows where you can get help and advice
annonsering / advertisement
34

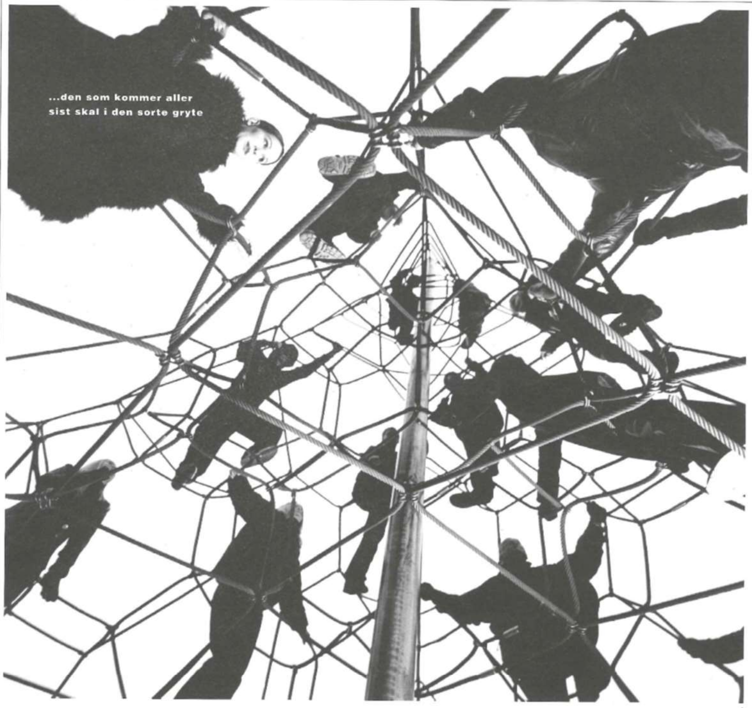

I år vil mange trenge psykiatrisk hjelp... sørg for at hjelpen blir så kortvarig som mulig

Hvert eneste år søker ca. 5000 mennesker psykiatrisk hjelp i Rogaland. De som tar kontakt så snart de første symptomene viser seg har størst sjanse for å bli friske etter kortvarig behandling.

Tidlige tegn på psykose

- *trekker seg tilbake fra venner og familie*
- *stenger seg inne, sover dårlig og spiser lite*
- *har angst*
- *slutter å vaske og stelle seg*
- *er ukonsentrerte*
- *snakker og skriver om meningsløse ting*
- *får sterke følelsesmessige reaksjoner*
- *blir uttrykksløse og reagerer ikke i det hele tatt*
- *føler seg forfulgt, eller kontrollert av stemmer utenfra*
- *tror de har magiske evner*

Alle som har mistanke om at noe alvorlig er galt, kan ringe oss og få råd og hjelp. Hvis nødvendig kan vi også hjelpe med hjemmebesøk og behandlingstilbud.
Vi bistår også lærere, helse- og sosialarbeidere som ønsker faglig veiledning.

TIPS
TIDLIG OPPDAGELSE OG BEHANDLING AV PSYKOSER

Sør-Rogaland 51 67 04 88
Nord-Rogaland 52 73 27 00
e-post: tips@rps.netpower.no, www.tips-info.com

SØK HJELP SÅ FORT SOM MULIG, DA ER SJANSEN STØRST FOR Å BLI FRISK

Statens Helsetilsyn, Rogaland Fylkeskommune, PsykOpp, Fylkessjukehuset i Haugesund, Rogaland Psykiatriske Sykehus

This year many people will need psychiatric help... make sure the period of help is as short as possible

annonsering / advertisement

den som ikke har gjemt seg nå,
den skal stå

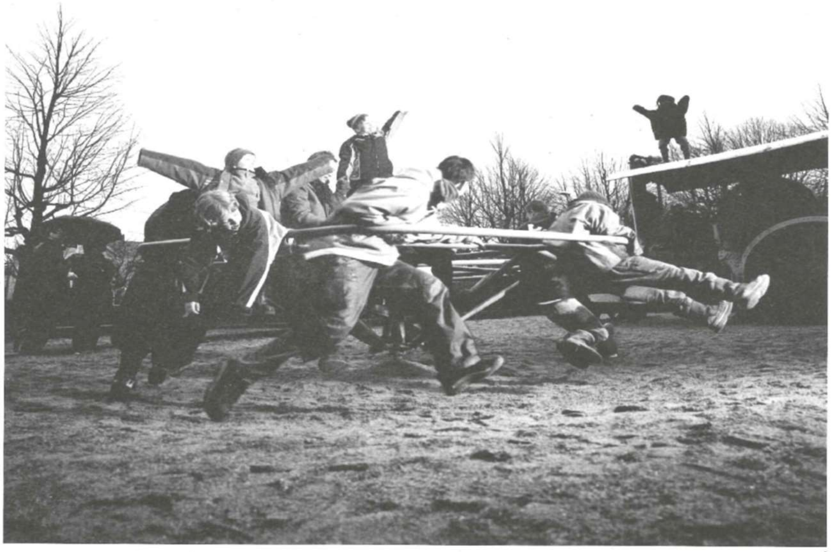

I år vil mange vegre seg mot å søke psykiatrisk hjelp... vi kan hjelpe dem som vil hjelpe andre

Mennesker som utvikler psykiske lidelser er ofte de siste som vil innrømme at noe er galt. Venner og familie som ønsker å hjelpe blir ofte avvist. Vi kan sørge for at du får de råd du trenger for å hjelpe andre.

Tidlige tegn på psykose

- *trekker seg tilbake fra venner og familie*
- *stenger seg inne, sover dårlig og spiser lite*
- *har angst*
- *slutter å vaske og stelle seg*
- *er ukonsentrerte*
- *snakker og skriver om meningsløse ting*
- *får sterke følelsesmessige reaksjoner*
- *blir uttrykksløse og reagerer ikke i det hele tatt*
- *føler seg forfulgt, eller kontrollert av stemmer utenfra*
- *tror de har magiske evner*

Alle som har mistanke om at noe alvorlig er galt, kan ringe oss og få råd og hjelp. Hvis nødvendig kan vi også hjelpe med hjemmebesøk og behandlingstilbud.
Vi bistår også lærere, helse- og sosialarbeidere som ønsker faglig veiledning.

TIPS
TIDLIG OPPDAGELSE OG BEHANDLING AV PSYKOSER

Sør-Rogaland 51 67 04 88
Nord-Rogaland 52 73 27 00
e-post: tips@rps.netpower.no, www.tips-info.com

SØK HJELP SÅ FORT SOM MULIG, DA ER SJANSEN STØRST FOR Å BLI FRISK

Statens Helsetilsyn, Rogaland Fylkeskommune, PsykOpp, Fylkessjukehuset i Haugesund, Rogaland Psykiatriske Sykehus

This year many people will hesitate getting psychiatric help... we can help those who will help others

annonsering / advertisement

40

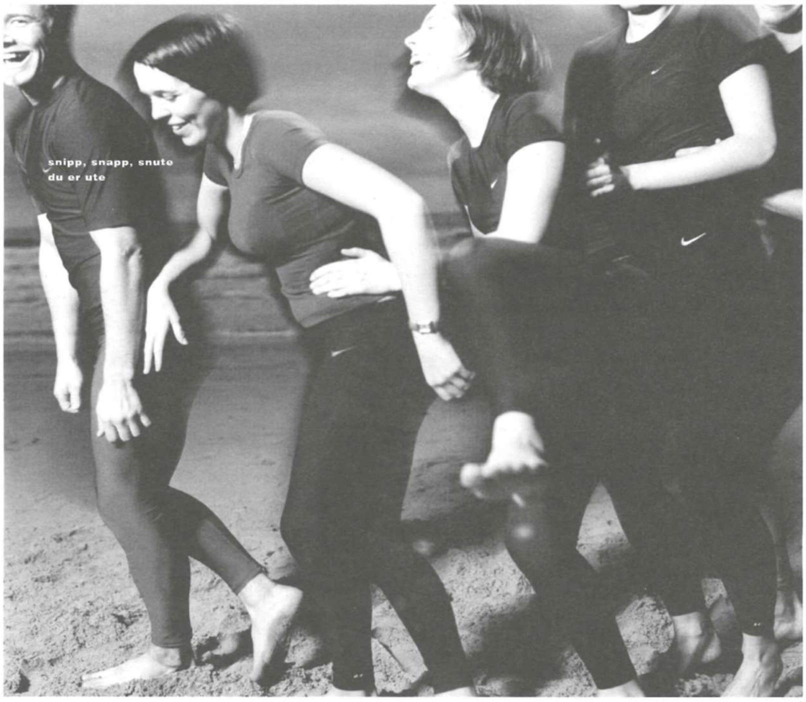

**I år vil mange tro at det er vanskelig å få psykiatrisk hjelp...
ta en telefon og la oss motbevise det**

Dessverre er det mange som unnlater å søke hjelp fordi de tror det tar så lang tid å komme til lege eller sykehus. Faktisk er det bare å ta en telefon til oss, så får du den hjelpen du behøver.

Tidlige tegn på psykose

- *trekker seg tilbake fra venner og familie*
- *stenger seg inne, sover dårlig og spiser lite*
- *har angst*
- *slutter å vaske og stelle seg*
- *er ukonsentrerte*
- *snakker og skriver om meningsløse ting*
- *får sterke følelsesmessige reaksjoner*
- *blir uttrykksløse og reagerer ikke i det hele tatt*
- *føler seg forfulgt, eller kontrollert av stemmer utenfra*
- *tror de har magiske evner*

Alle som har mistanke om at noe alvorlig er galt, kan ringe oss og få råd og hjelp. Hvis nødvendig kan vi også hjelpe med hjemmebesøk og behandlingstilbud. Vi bistår også lærere, helse- og sosialarbeidere som ønsker faglig veiledning.

TIPS
TIDLIG OPPDAGELSE OG BEHANDLING AV PSYKOSER

Sør-Rogaland 51 67 04 88
Nord-Rogaland 52 73 27 00
e-post: tips@rps.netpower.no, www.tips-info.com

SØK HJELP SÅ FORT SOM MULIG, DA ER SJANSEN STØRST FOR Å BLI FRISK

Statens Helsetilsyn, Rogaland Fylkeskommune, PsykOpp, Fylkessjukehuset i Haugesund, Rogaland Psykiatriske Sykehus

This year many people will think it is difficult to get psychiatric help... contact us, and let us prove you are wrong.

annonsering / advertisement

41

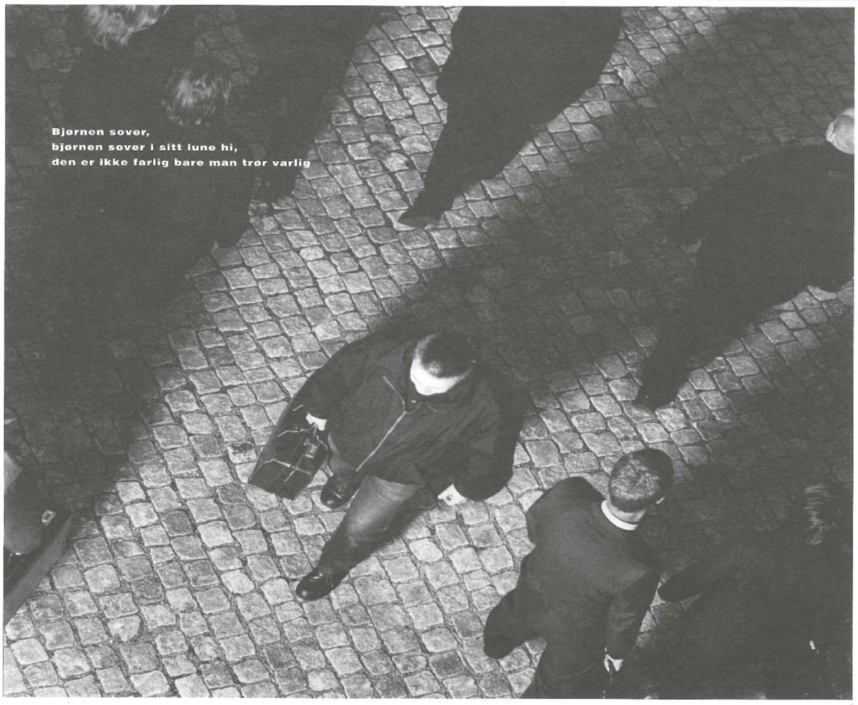

I år vil mange bli urolige for at venner og familie utvikler psykiske problemer... sørg for at uroen blir så kortvarig som mulig

Psykiske lidelser kommer ofte snikende. Den som rammes er den siste til å innse og innrømme at noe er galt. Da er det viktig at andre tar ansvar og søker hjelp.

Tidlige tegn på psykose

- *trekker seg tilbake fra venner og familie*
- *stenger seg inne, sover dårlig og spiser lite*
- *har angst*
- *slutter å vaske og stelle seg*
- *er ukonsentrerte*
- *snakker og skriver om meningsløse ting*
- *får sterke følelsesmessige reaksjoner*
- *blir uttrykksløse og reagerer ikke i det hele tatt*
- *føler seg forfulgt, eller kontrollert av stemmer utenfra*
- *tror de har magiske evner*

Alle som har mistanke om at noe alvorlig er galt, kan ringe oss og få råd og hjelp. Hvis nødvendig kan vi også hjelpe med hjemmebesøk og behandlingstilbud.
Vi bistår også lærere, helse- og sosialarbeidere som ønsker faglig veiledning.

TIPS
TIDLIG OPPDAGELSE OG BEHANDLING AV PSYKOSER

Sør-Rogaland 51 67 04 88
Nord-Rogaland 52 73 27 00
e-post: tips@rps.netpower.no, www.tips-info.com

SØK HJELP SÅ FORT SOM MULIG, DA ER SJANSEN STØRST FOR Å BLI FRISK

Statens Helsetilsyn, Rogaland Fylkeskommune, PsykOpp, Fylkessjukehuset i Haugesund, Rogaland Psykiatriske Sykehus

This year many people will worry about friends and family members developing psychiatric problems... make sure the worrying is as short as possible

annonsering / advertisement

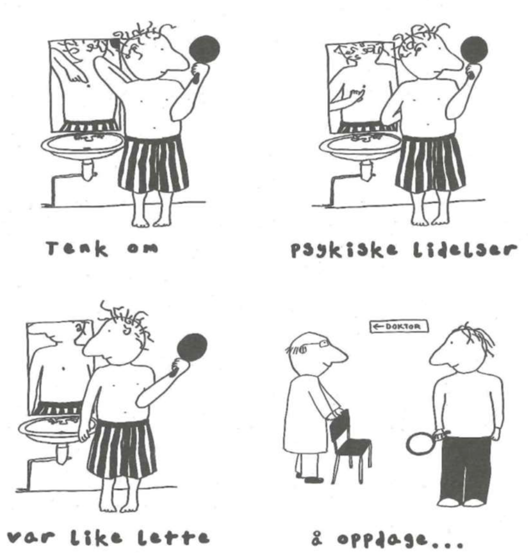

Kreft og psykiatri har en ting til felles: de er begge lidelser som var vanskelige å snakke om for bare noen få år siden. Et iherdig opplysningsarbeid har gjort at de fleste kjenner symptomene på f. eks ondartet føflekk.
Alle vet at det gjelder å komme raskt til legen for å unngå at det sprer seg. Psykiske lidelser har ikke samme "status". Fortsatt er de vanskelig å snakke om. For mange er de første symptomene lite kjent, og ikke alle er klar over at de kan stoppes hvis de behandles tidlig. Ikke alle vet hvordan de skal forholde seg til sykdommen, eller hvordan de søker hjelp. Det er hjelp å få for både den syke og pårørende.

TIPS tilbyr råd og hjelp til mennesker som rammes av alvorlige psykiske lidelser for første gang, eller som føler at eksisterende problemer blir alvorlig forverret. Alle som har mistanke om at noe er galt kan ringe oss og få råd og hjelp. I enkelte tilfeller kan vi også hjelpe med hjemmebesøk og behandlingstilbud. Vi bistår også lærere og helse- og sosialarbeidere som ønsker faglig rådgivning.

Ring hverdager fra 8 – 15. Utenom dette kan du legge igjen beskjed på telefonsvarer, så ringer vi tilbake.

Tidlige tegn på psykose

- trekker seg tilbake fra venner og familie
- stenger seg inne, sover dårlig og spiser lite
- har angst
- blir aggressiv
- slutter å vaske og stelle seg
- er ukonsentrert
- snakker og skriver om meningsløse ting
- får sterke følelsesmessige reaksjoner
- blir uttrykksløse og reagerer ikke i det hele tatt
- føler seg forfulgt, eller kontrollert av stemmer utenfra
- tror de har magiske evner

TIPS

Sør-Rogaland 51 67 04 88
Nord-Rogaland 52 73 27 00
e-post: tips@rps.netpower.no,
www.tips-info.com

SØK HJELP SÅ FORT SOM MULIG, DA ER SJANSEN STØRST FOR Å BLI FRISK

What if psychiatric illnesses were as easy to detect as...

annonsering / advertisement

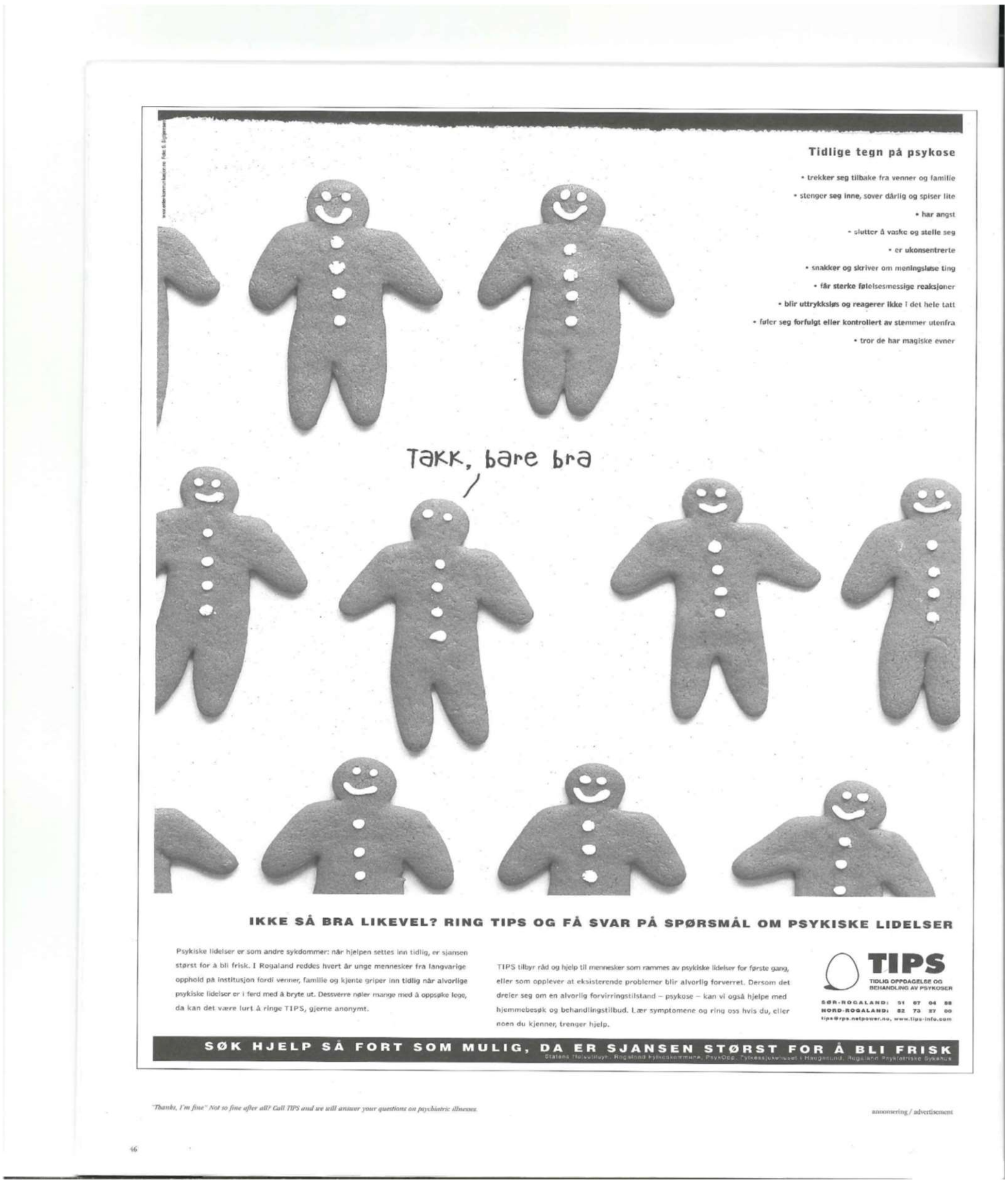
Tidlige tegn på psykose
• trekker seg tilbake fra venner og familie
• stenger seg inne, sover dårlig og spiser lite
• har angst
• slutter å vaske og stelle seg
• er ukonsentrerte
• snakker og skriver om meningsløse ting
• får sterke følelsesmessige reaksjoner
• blir uttrykksløs og reagerer ikke i det hele tatt
• føler seg forfulgt eller kontrollert av stemmer utenfra
• tror de har magiske evner
Takk, bare bra
IKKE SÅ BRA LIKEVEL? RING TIPS OG FÅ SVAR PÅ SPØRSMÅL OM PSYKISKE LIDELSER
Psykiske lidelser er som andre sykdommer: når hjelpen settes inn tidlig, er sjansen størst for å bli frisk. I Rogaland reddes hvert år unge mennesker fra langvarige opphold på institusjon fordi venner, familie og kjente griper inn tidlig når alvorlige psykiske lidelser er i ferd med å bryte ut. Dessverre nøler mange med å oppsøke lege, da kan det være lurt å ringe TIPS, gjerne anonymt.
TIPS tilbyr råd og hjelp til mennesker som rammes av psykiske lidelser for første gang, eller som opplever at eksisterende problemer blir alvorlig forverret. Dersom det dreier seg om en alvorlig forvirringstilstand – psykose – kan vi også hjelpe med hjemmebesøk og behandlingstilbud. Lær symptomene og ring oss hvis du, eller noen du kjenner, trenger hjelp.
TIPS
TIDLIG OPPDAGELSE OG BEHANDLING AV PSYKOSER
SØR-ROGALAND:
NORD-ROGALAND:
SØK HJELP SÅ FORT SOM MULIG, DA ER SJANSEN STØRST FOR Å BLI FRISK
"Thanks, I'm fine" Not so fine after all? Call TIPS and we will answer your questions on psychiatric illnesses.
annonsering / advertisement
46

OGSÅ I 2001 VIL MANGE GÅ INN I EN PSYKOSE

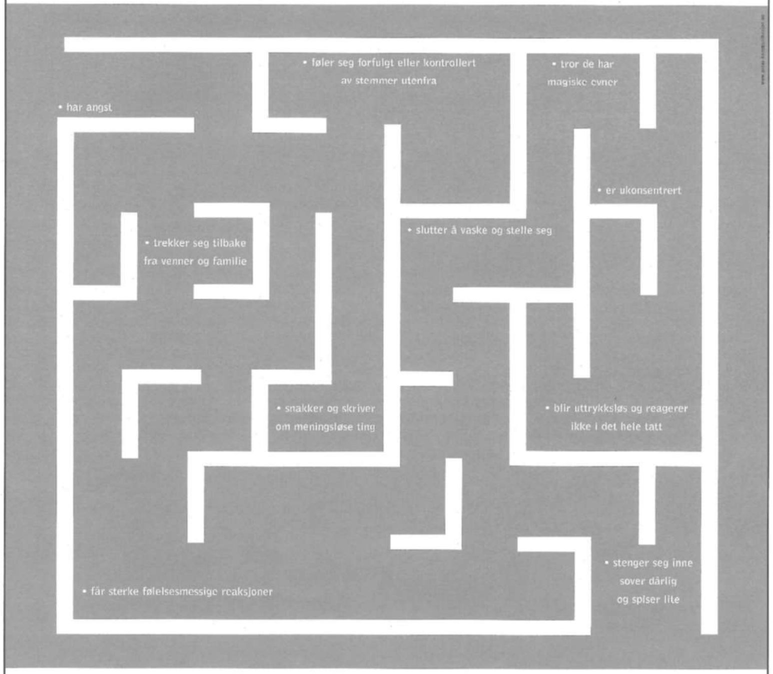

MED DIN HJELP KAN VEIEN UT BLI KORTERE

Psykiske lidelser kommer som regel gradvis. Det kan ta lang tid fra de første tegnene viser seg, til behandling blir gitt. I hvert fall hvis ingen griper inn. Ofte er det venner, familie eller kolleger som først skjønner at noe er galt. Merkelig oppførsel, uten forklarlig årsak, som varer i flere uker er et varsel om at en bør oppsøke lege. Den syke vil ofte ikke innse at noe er galt, som regel er det andre om kontakter helsevesenet. Kjenner du symptomene på psykose og husker på at det er mulig å ringe og få råd og hjelp, kan du være med å hindre at en psykose utvikler seg til å bli en livslang lidelse.

Rogaland er et foregangsfylke

I fire år har Rogaland vært forsøksfylke for tidlig oppdagelse og behandling av psykose(TIPS). Gjennom folkeopplysning, mulighet for folk flest å ringe direkte til fagfolk for råd og hjelp, samt kursing av leger, lærere, helse- og sosialarbeidere, har det vist seg at flere kommer tidligere til behandling og får bedre resultat av behandlingen. Også behandlingstilbudet har blitt bedre. Erfaringene som er gjort i Rogaland, vil bli brukt andre steder i Norge, og også utenlandske institusjoner viser interesse for det arbeidet som er gjort og de resultatene det har gitt.

Ungdom, foreldre, leger, lærere og sosialarbeidere – ring oss!

Selv om TIPS nå er over som prosjekt, vil tilbudet om råd og hjelp fortsette. Det blir fortsatt mulig å ringe fagfolk direkte. Vi vil fortsatt kunne oppsøke pasienten hjemme, på legekontor eller ta i mot på sykehuset. Vi vil fortsatt kunne tilby rask behandling. Familier til unge med psykose vil fortsatt få tilbud om flerfamiliegrupper.

Før TIPS-prosjektet startet kunne det gå fra to til fire år fra de første symptomene meldte seg til behandlingen ble satt i gang, nå er gjennomsnittet fire måneder.

Vi takker alle som har tatt ansvar og kontaktet oss.

TIPS
TIDLIG OPPDAGELSE OG BEHANDLING AV PSYKOSER

SØR-ROGALAND: 51 67 04 88
NORD-ROGALAND: 52 73 27 00
tips@rps.netpower.no, www.tips-info.com

SØK HJELP SÅ FORT SOM MULIG, DA ER SJANSEN STØRST FOR Å BLI FRISK

[illegible] Helsetilsyn, Rogaland Fylkeskommune, PsykOpp, Fylkessjukehuset i Haugesund, Rogaland Psykiatriske Sjukehus

Also in the year 2001 many will go into a psychosis. With your help the way out could shorten?

annonsering / advertisement

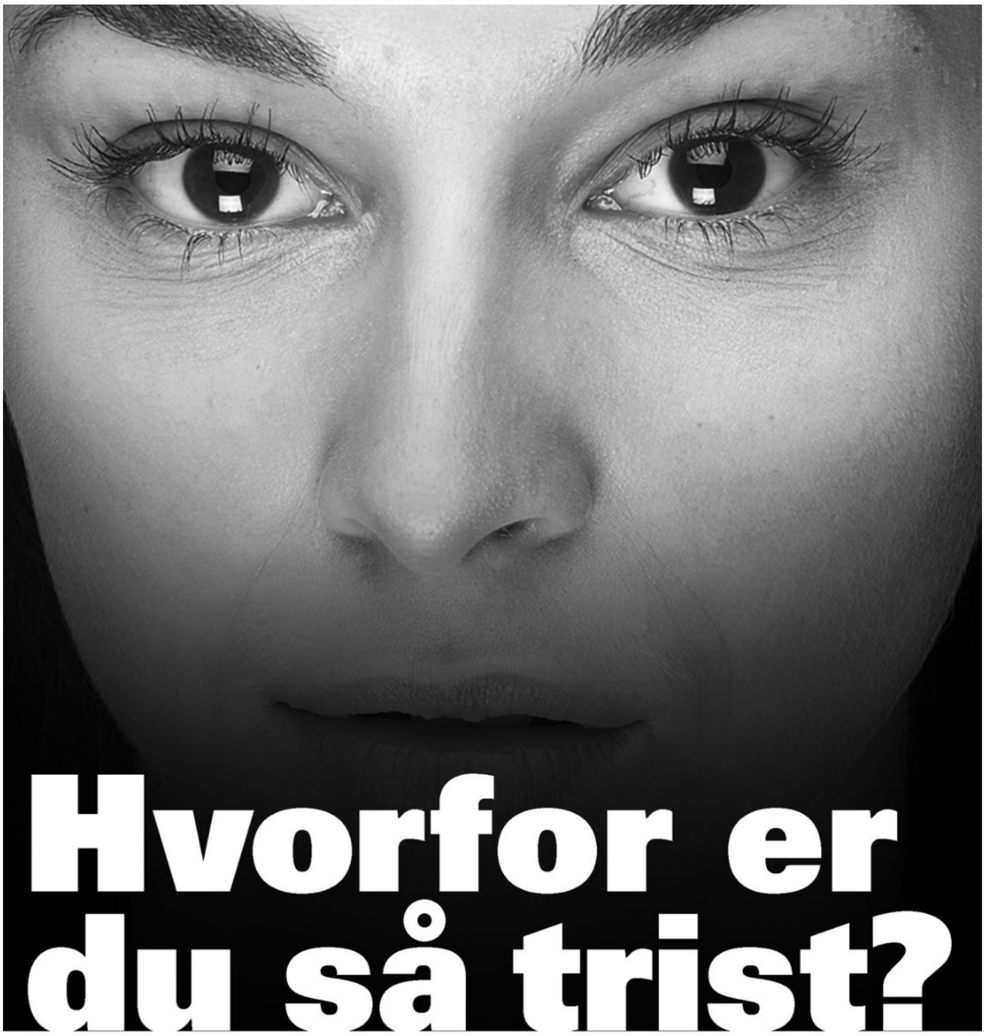

Få ting gjør oss mer urolige enn at noen vi kjenner godt forandrer seg uten at vi forstår hvorfor. Følelsen av at noe er galt, uten at du kan finne en årsak, er ubehagelig og noen ganger skremmende. Når forsøk på å nå fram med spørsmål eller nærhet blir avvist, møtt med taushet eller sinne, er det naturlig å bli engstelig. Hvis symptomer på psykiske problemer varer uker og måneder, blir stadig mer fremtredende og går ut over dagliglivet, skal en vurdere å søke hjelp. Hvis det er vanskelig å ta problemene opp med fastlegen, kan du ringe TIPS. Her møter du erfarne fagfolk som vil gi råd om hvordan du får hjelp og behandling hvis det er nødvendig.
Du kan gjerne ringe anonymt.

TIDLIGE TEGN PÅ PSYKOSE

→ isolerer seg → sover dårlig
→ tristhet → angst → konsentrasjonsvansker
→ forsømmer hygiene, jobb eller skole
→ opptatt av temaer som døden, politikk eller religion
→ store humørsvingninger → hører stemmer
→ snakker usammenhengende
→ føler seg forfulgt eller styrt av andre

Stavanger Universitetssjukehus
Helse Stavanger HF
Psykiatrisk klinikk

TIPS
TIDLIG OPPDAGELSE OG BEHANDLING AV PSYKOSER

51 51 59 59
hverdager 08.00-15.00
www.tips-info.com

SØK HJELP SÅ RASKT SOM MULIG - DA ER SJANSEN STØRST FOR Å BLI FRISK

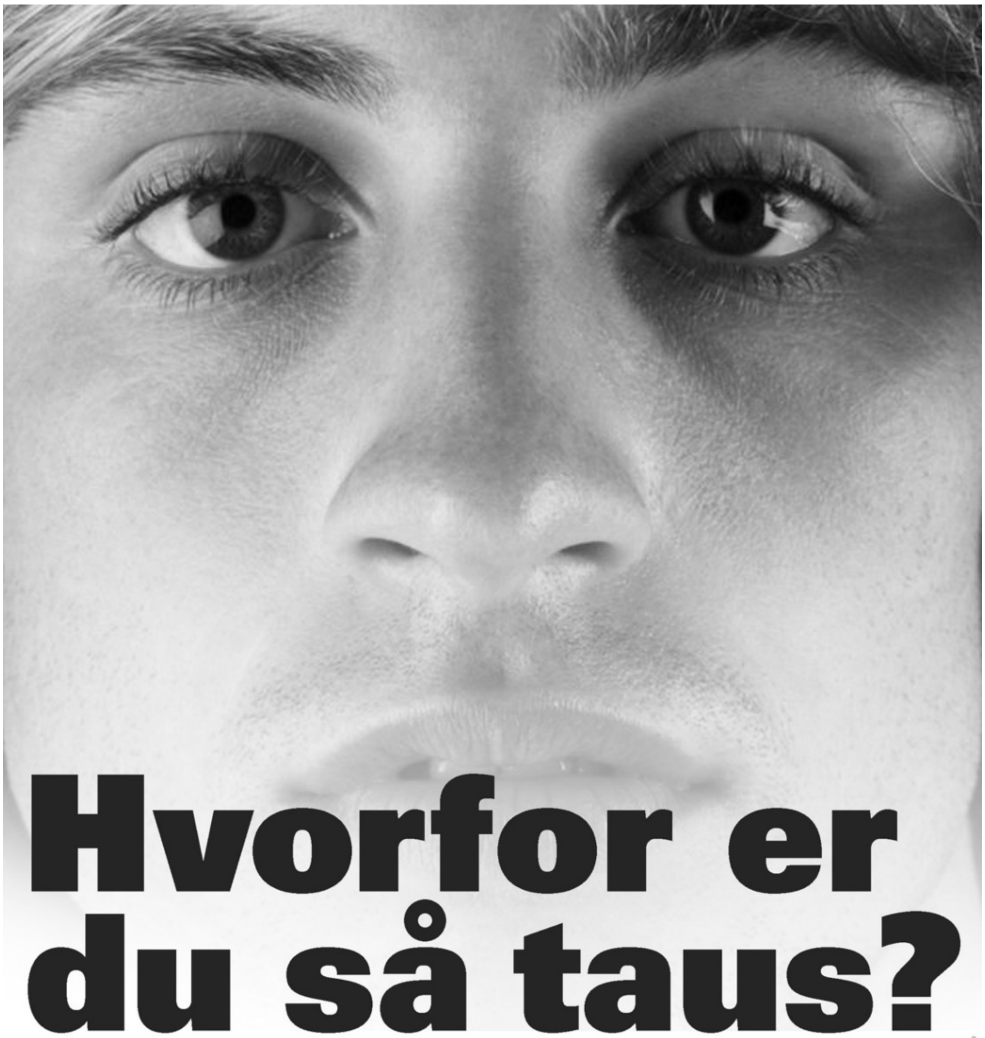

Få ting gjør oss mer urolige enn at noen vi kjenner godt forandrer seg uten at vi forstår hvorfor. Følelsen av at noe er galt, uten at du kan finne en årsak, er ubehagelig og noen ganger skremmende. Når forsøk på å nå fram med spørsmål eller nærhet blir avvist, møtt med taushet eller sinne, er det naturlig å bli engstelig. Hvis symptomer på psykiske problemer varer uker og måneder, blir stadig mer fremtredende og går ut over dagliglivet, skal en vurdere å søke hjelp. Hvis det er vanskelig å ta problemene opp med fastlegen, kan du ringe TIPS. Her møter du erfarne fagfolk som vil gi råd om hvordan du får hjelp og behandling hvis det er nødvendig.
Du kan gjerne ringe anonymt.

TIDLIGE TEGN PÅ PSYKOSE

→ isolerer seg → sover dårlig
→ tristhet → angst → konsentrasjonsvansker
→ forsømmer hygiene, jobb eller skole
→ opptatt av temaer som døden, politikk eller religion
→ store humørsvingninger → hører stemmer
→ snakker usammenhengende
→ føler seg forfulgt eller styrt av andre

Stavanger Universitetssjukehus
Helse Stavanger HF
Psykiatrisk klinikk

TIPS
TIDLIG OPPDAGELSE OG BEHANDLING AV PSYKOSER

51 51 59 59
hverdager 08.00-15.00
www.tips-info.com

SØK HJELP SÅ RASKT SOM MULIG - DA ER SJANSEN STØRST FOR Å BLI FRISK

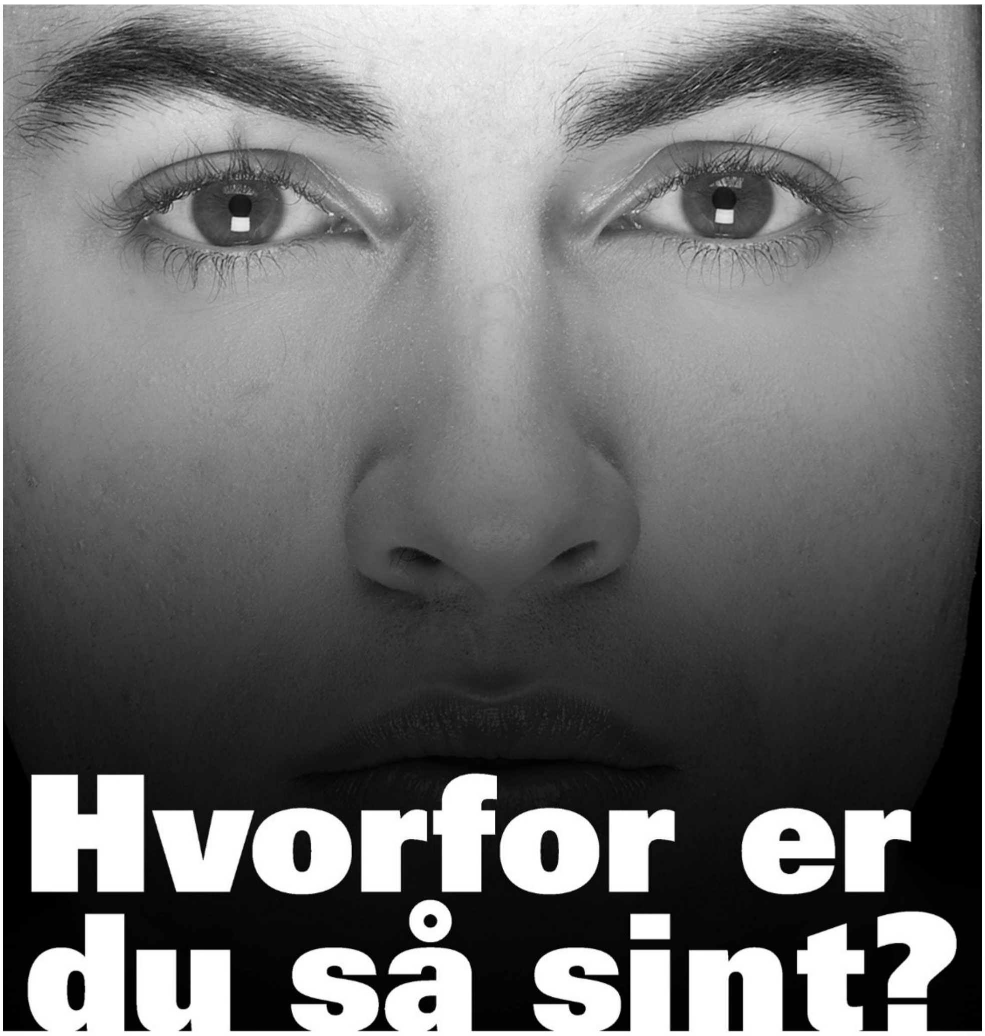

Få ting gjør oss mer urolige enn at noen vi kjenner godt forandrer seg uten at vi forstår hvorfor. Følelsen av at noe er galt, uten at du kan finne en årsak, er ubehagelig og noen ganger skremmende. Når forsøk på å nå fram med spørsmål eller nærhet blir avvist, møtt med taushet eller sinne, er det naturlig å bli engstelig. Hvis symptomer på psykiske problemer varer uker og måneder, blir stadig mer fremtredende og går ut over dagliglivet, skal en vurdere å søke hjelp. Hvis det er vanskelig å ta problemene opp med fastlegen, kan du ringe TIPS. Her møter du erfarne fagfolk som vil gi råd om hvordan du får hjelp og behandling hvis det er nødvendig.
Du kan gjerne ringe anonymt.

TIDLIGE TEGN PÅ PSYKOSE

→ isolerer seg → sover dårlig
→ tristhet → angst → konsentrasjonsvansker
→ forsømmer hygiene, jobb eller skole
→ opptatt av temaer som døden, politikk eller religion
→ store humørsvingninger → hører stemmer
→ snakker usammenhengende
→ føler seg forfulgt eller styrt av andre

Stavanger Universitetssjukehus
Helse Stavanger HF
Psykiatrisk klinikk

TIPS
TIDLIG OPPDAGELSE OG BEHANDLING AV PSYKOSER

51 51 59 59
hverdager 08.00-15.00
www.tips-info.com

SØK HJELP SÅ RASKT SOM MULIG - DA ER SJANSEN STØRST FOR Å BLI FRISK

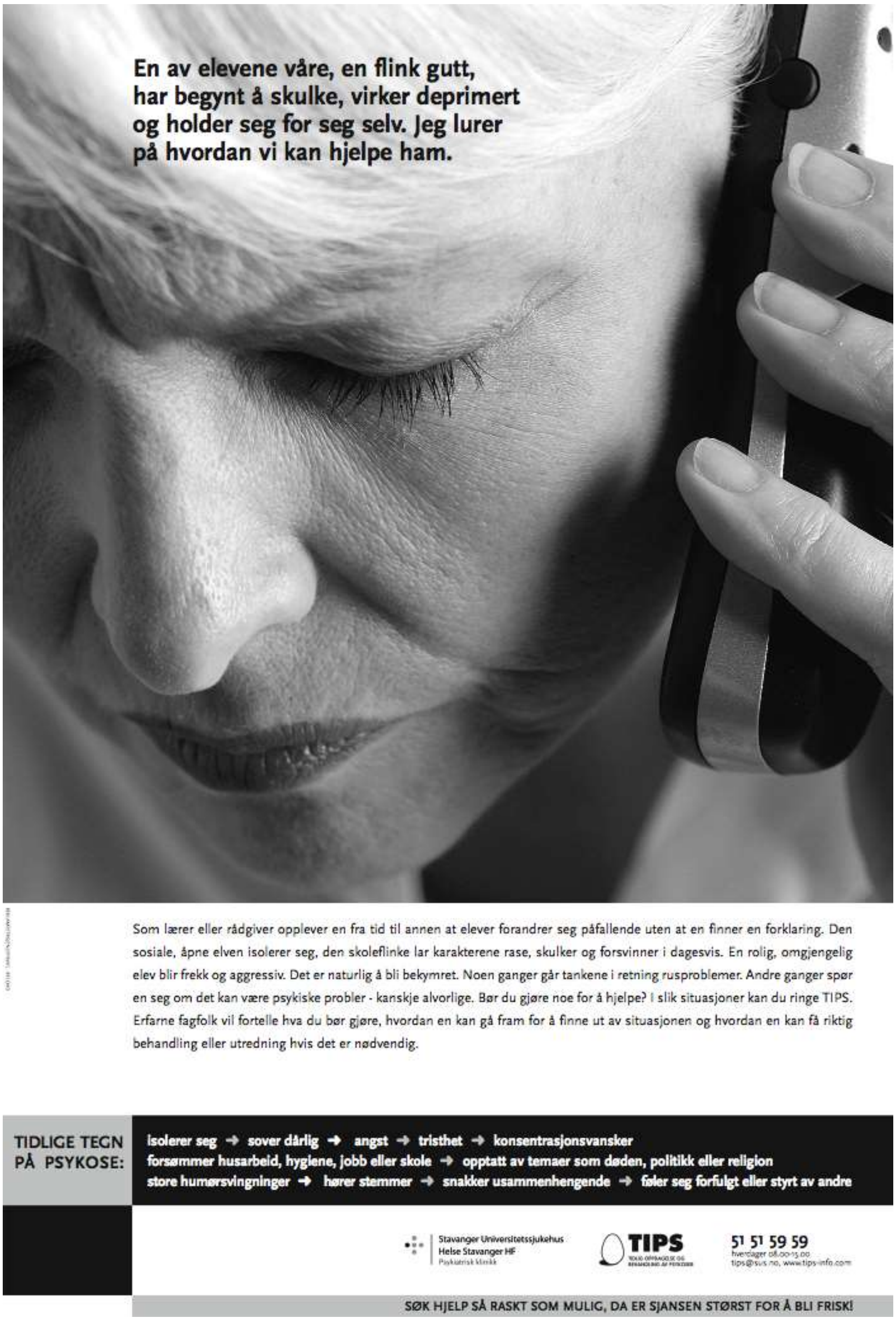

En av elevene våre, en flink gutt, har begynt å skulke, virker deprimert og holder seg for seg selv. Jeg lurer på hvordan vi kan hjelpe ham.
Som lærer eller rådgiver opplever en fra tid til annen at elever forandrer seg påfallende uten at en finner en forklaring. Den sosiale, åpne elven isolerer seg, den skoleflinke lar karakterene rase, skulker og forsvinner i dagesvis. En rolig, omgjengelig elev blir frekk og aggressiv. Det er naturlig å bli bekymret. Noen ganger går tankene i retning rusproblemer. Andre ganger spør en seg om det kan være psykiske probler - kanskje alvorlige. Bør du gjøre noe for å hjelpe? I slik situasjoner kan du ringe TIPS. Erfarne fagfolk vil fortelle hva du bør gjøre, hvordan en kan gå fram for å finne ut av situasjonen og hvordan en kan få riktig behandling eller utredning hvis det er nødvendig.
TIDLIGE TEGN PÅ PSYKOSE:
isolerer seg → sover dårlig → angst → tristhet → konsentrasjonsvansker
forsømmer husarbeid, hygiene, jobb eller skole → opptatt av temaer som døden, politikk eller religion
store humørsvingninger → hører stemmer → snakker usammenhengende → føler seg forfulgt eller styrt av andre
Stavanger Universitetssjukehus
Helse Stavanger HF
TIPS
51 51 59 59
tips@sus.no, www.tips-info.com
SØK HJELP SÅ RASKT SOM MULIG, DA ER SJANSEN STØRST FOR Å BLI FRISK!

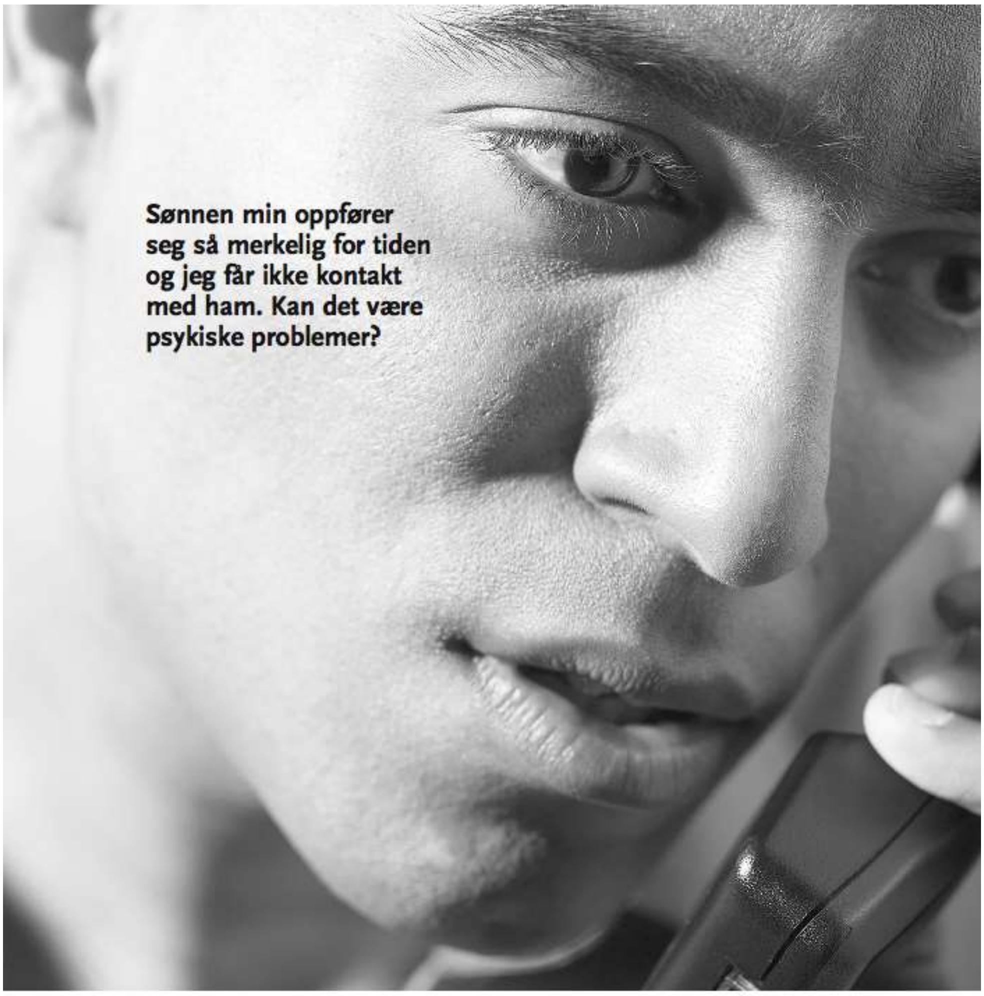

At ungdom forandrer seg, er naturlig. Når tenåringen forandrer seg merkbart på kort tid - blir gjerne foreldrene bekymret. Hvis undommen isolerer seg, er taus og virker deprimert er det naturlig å være på vakt, særlig hvis tilstanden varer i uker eller måneder. En leter gjerne etter forklaringer i noe som har skjedd; som kjærlighetssorg, skilsmisse i familien, nederlag på skolen eller på idrettsbanen. Har ungdommen store humørsvingninger, eller symptomer du har vanskelig for å forstå, er det lurt å søke råd. Foreldre blir naturlig nok redd for at de opplever tegn på rusproblemer eller en alvorlig psykisk lidelse. Ved en psykose har han eller hun ofte problemer med å sette ord på det på det som skjer. I en slik situasjon kan du ringe TIPS. Erfarne fagfolk vil fortelle hva du bør gjøre, hvordan dere får riktig behandling eller få et tilbud om videre utredning.

TIDLIGE TEGN PÅ PSYKOSE:

isolerer seg → sover dårlig → angst → tristhet → konsentrasjonsvansker
forsømmer husarbeid, hygiene, jobb eller skole → opptatt av temaer som døden, politikk eller religion
store humørsvingninger → hører stemmer → snakker usammenhengende → føler seg forfulgt eller styrt av andre

Stavanger Universitetssjukehus
Helse Stavanger HF
Psykiatrisk klinikk

TIPS

51 51 59 59
hverdager 08.00-15.00
tips@sus.no, www.tips-info.com

SØK HJELP SÅ RASKT SOM MULIG, DA ER SJANSEN STØRST FOR Å BLI FRISK!

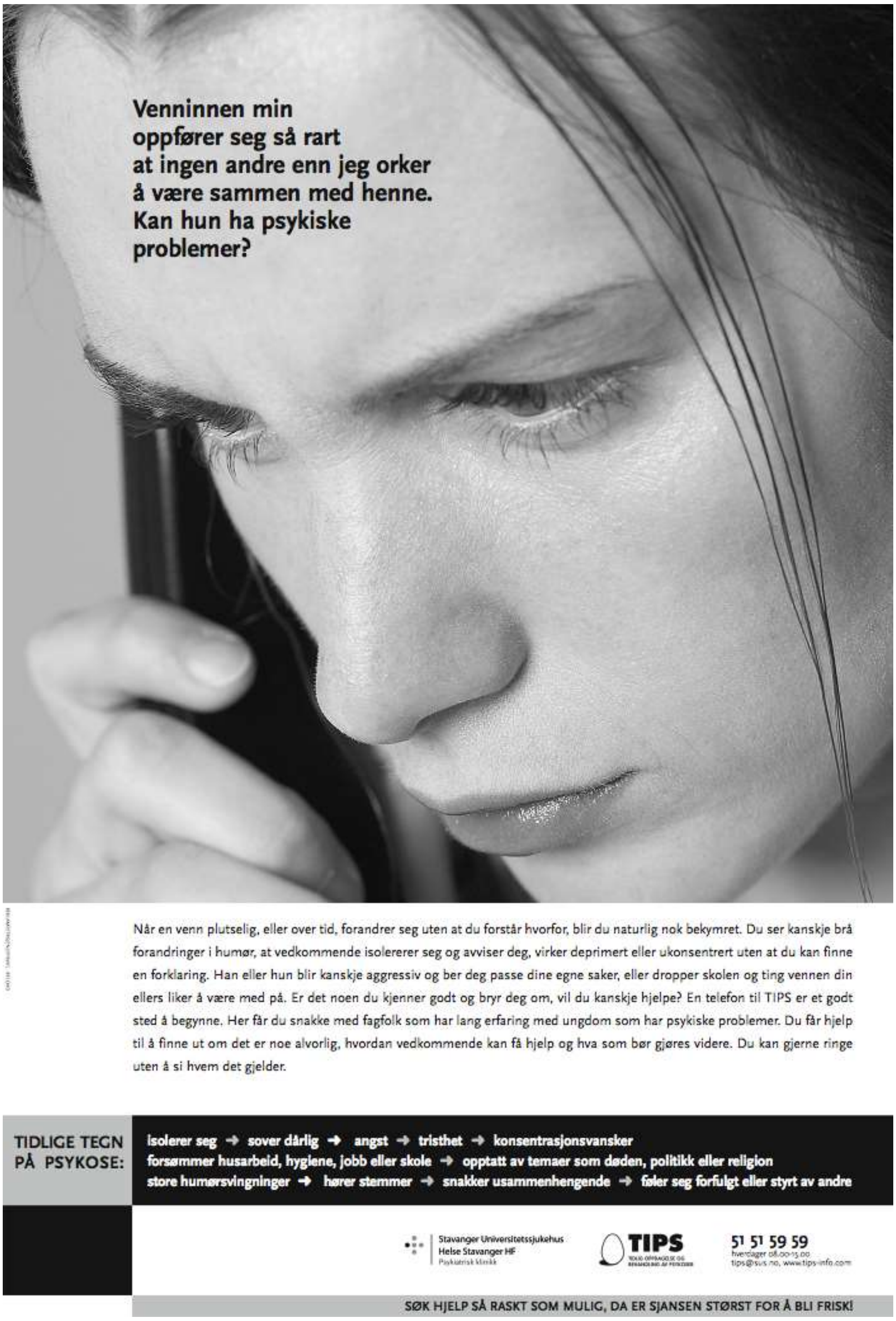

Venninnen min
oppfører seg så rart
at ingen andre enn jeg orker
å være sammen med henne.
Kan hun ha psykiske
problemer?
Når en venn plutselig, eller over tid, forandrer seg uten at du forstår hvorfor, blir du naturlig nok bekymret. Du ser kanskje brå forandringer i humør, at vedkommende isolerer seg og avviser deg, virker deprimert eller ukonsentrert uten at du kan finne en forklaring. Han eller hun blir kanskje aggressiv og ber deg passe dine egne saker, eller dropper skolen og ting vennen din ellers liker å være med på. Er det noen du kjenner godt og bryr deg om, vil du kanskje hjelpe? En telefon til TIPS er et godt sted å begynne. Her får du snakke med fagfolk som har lang erfaring med ungdom som har psykiske problemer. Du får hjelp til å finne ut om det er noe alvorlig, hvordan vedkommende kan få hjelp og hva som bør gjøres videre. Du kan gjerne ringe uten å si hvem det gjelder.
TIDLIGE TEGN PÅ PSYKOSE:
isolerer seg → sover dårlig → angst → tristhet → konsentrasjonsvansker
forsømmer husarbeid, hygiene, jobb eller skole → opptatt av temaer som døden, politikk eller religion
store humørsvingninger → hører stemmer → snakker usammenhengende → føler seg forfulgt eller styrt av andre
Stavanger Universitetssjukehus
Helse Stavanger HF
Psykiatrisk klinikk
TIPS
51 51 59 59
hverdager 08.00-15.00
tips@sus.no, www.tips-info.com
SØK HJELP SÅ RASKT SOM MULIG, DA ER SJANSEN STØRST FOR Å BLI FRISK!

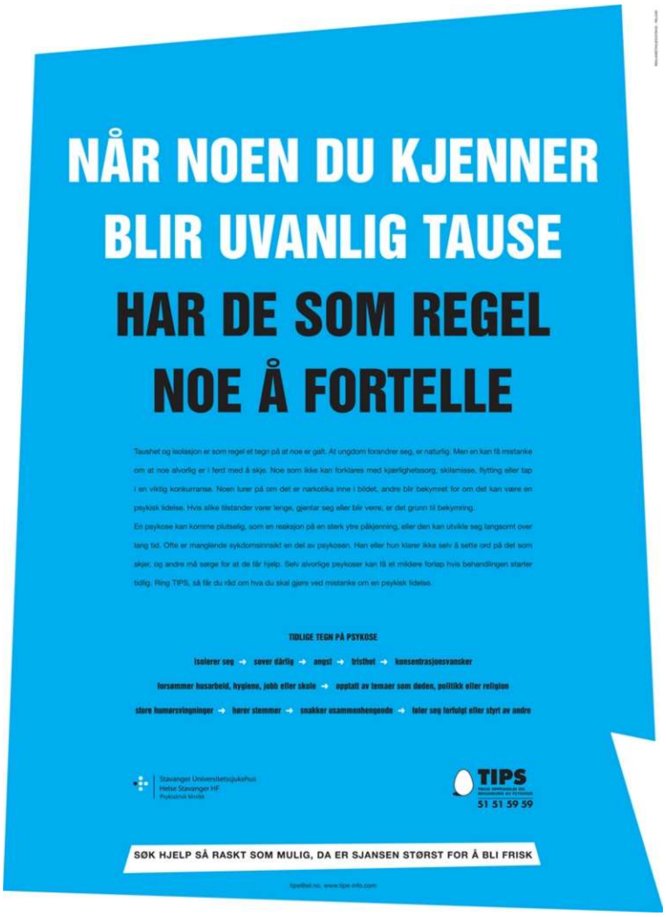
NÅR NOEN DU KJENNER
BLIR UVANLIG TAUSE
HAR DE SOM REGEL
NOE Å FORTELLE
Taushet og isolasjon er som regel et tegn på at noe er galt. At ungdom forandrer seg, er naturlig. Men en kan få mistanke om at noe alvorlig er i ferd med å skje. Noe som ikke kan forklares med kjærlighetssorg, skilsmisse, flytting eller tap i en viktig konkurranse. Noen lurer på om det er narkotika inne i bildet, andre blir bekymret for om det kan være en psykisk lidelse. Hvis slike tilstander varer lenge, gjentar seg eller blir verre, er det grunn til bekymring.
En psykose kan komme plutselig, som en reaksjon på en sterk ytre påkjenning, eller den kan utvikle seg langsomt over lang tid. Ofte er manglende sykdomsinnsikt en del av psykosen. Han eller hun klarer ikke selv å sette ord på det som skjer, og andre må sørge for at de får hjelp. Selv alvorlige psykoser kan få et mildere forløp hvis behandlingen starter tidlig. Ring TIPS, så får du råd om hva du skal gjøre ved mistanke om en psykisk lidelse.
TIDLIGE TEGN PÅ PSYKOSE
isolerer seg → sover dårlig → angst → tristhet → konsentrasjonsvansker
forsømmer husarbeid, hygiene, jobb eller skole → opptatt av temaer som døden, politikk eller religion
store humørsvingninger → hører stemmer → snakker usammenhengende → føler seg forfulgt eller styrt av andre
Stavanger Universitetssjukehus
Helse Stavanger HF
TIPS
51 51 59 59
SØK HJELP SÅ RASKT SOM MULIG, DA ER SJANSEN STØRST FOR Å BLI FRISK

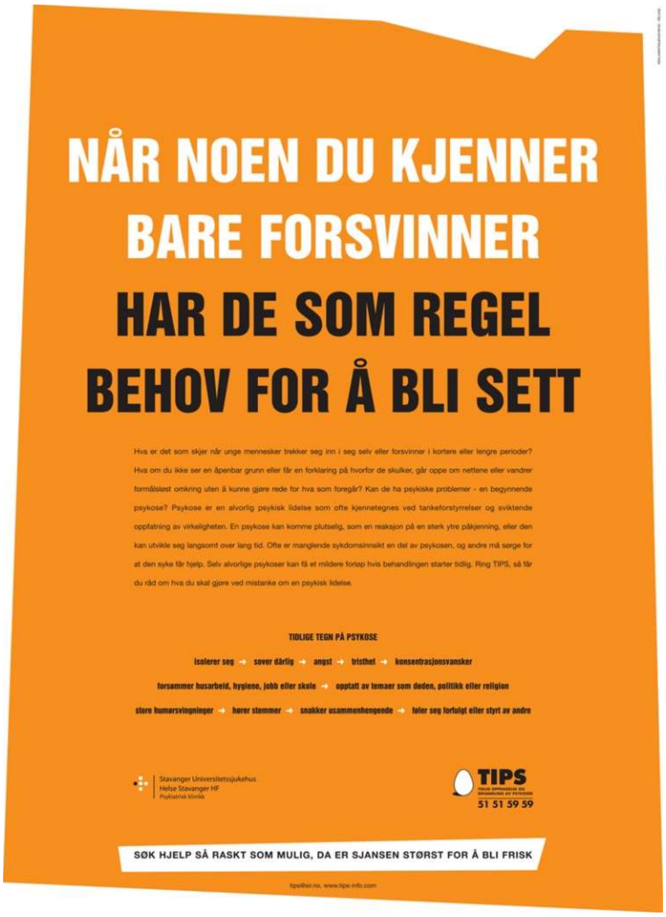
NÅR NOEN DU KJENNER BARE FORSVINNER
HAR DE SOM REGEL BEHOV FOR Å BLI SETT
Hva er det som skjer når unge mennesker trekker seg inn i seg selv eller forsvinner i kortere eller lengre perioder? Hva om du ikke ser en åpenbar grunn eller får en forklaring på hvorfor de skulker, går oppe om nettene eller vandrer formålsløst omkring uten å kunne gjøre rede for hva som foregår? Kan de ha psykiske problemer - en begynnende psykose? Psykose er en alvorlig psykisk lidelse som ofte kjennetegnes ved tankeforstyrrelser og sviktende oppfatning av virkeligheten. En psykose kan komme plutselig, som en reaksjon på en sterk ytre påkjenning, eller den kan utvikle seg langsomt over lang tid. Ofte er manglende sykdomsinnsikt en del av psykosen, og andre må sørge for at den syke får hjelp. Selv alvorlige psykoser kan få et mildere forløp hvis behandlingen starter tidlig. Ring TIPS, så får du råd om hva du skal gjøre ved mistanke om en psykisk lidelse.
TIDLIGE TEGN PÅ PSYKOSE
isolerer seg → sover dårlig → angst → tristhet → konsentrasjonsvansker
forsømmer husarbeid, hygiene, jobb eller skole → opptatt av temaer som døden, politikk eller religion
store humørsvingninger → hører stemmer → snakker usammenhengende → føler seg forfulgt eller styrt av andre
Stavanger Universitetssjukehus
Helse Stavanger HF
Psykiatrisk klinikk
TIPS
51 51 59 59
SØK HJELP SÅ RASKT SOM MULIG, DA ER SJANSEN STØRST FOR Å BLI FRISK

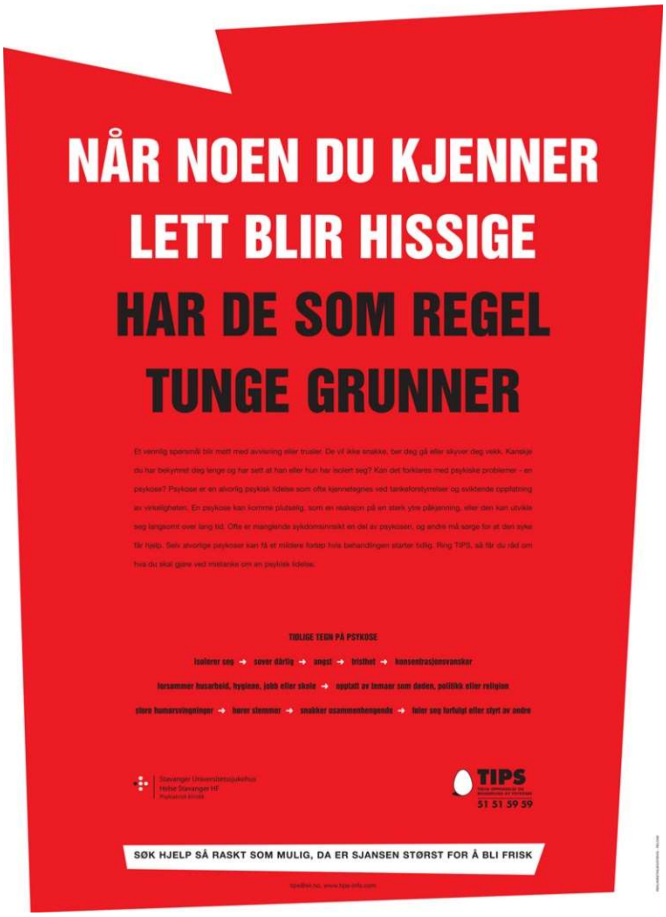
NÅR NOEN DU KJENNER
LETT BLIR HISSIGE
HAR DE SOM REGEL
TUNGE GRUNNER
Et vennlig spørsmål blir møtt med avvisning eller trusler. De vil ikke snakke, ber deg gå eller skyver deg vekk. Kanskje du har bekymret deg lenge og har sett at han eller hun har isolert seg? Kan det forklares med psykiske problemer - en psykose? Psykose er en alvorlig psykisk lidelse som ofte kjennetegnes ved tankeforstyrrelser og sviktende oppfatning av virkeligheten. En psykose kan komme plutselig, som en reaksjon på en sterk ytre påkjenning, eller den kan utvikle seg langsomt over lang tid. Ofte er manglende sykdomsinnsikt en del av psykosen, og andre må sørge for at den syke får hjelp. Selv alvorlige psykoser kan få et mildere forløp hvis behandlingen starter tidlig. Ring TIPS, så får du råd om hva du skal gjøre ved mistanke om en psykisk lidelse.
TIDLIGE TEGN PÅ PSYKOSE
isolerer seg → sover dårlig → angst → tristhet → konsentrasjonsvansker
forsømmer husarbeid, hygiene, jobb eller skole → opptatt av temaer som døden, politikk eller religion
store humørsvingninger → hører stemmer → snakker usammenhengende → føler seg forfulgt eller styrt av andre
Stavanger Universitetssjukehus
Helse Stavanger HF
TIPS
51 51 59 59
SØK HJELP SÅ RASKT SOM MULIG, DA ER SJANSEN STØRST FOR Å BLI FRISK

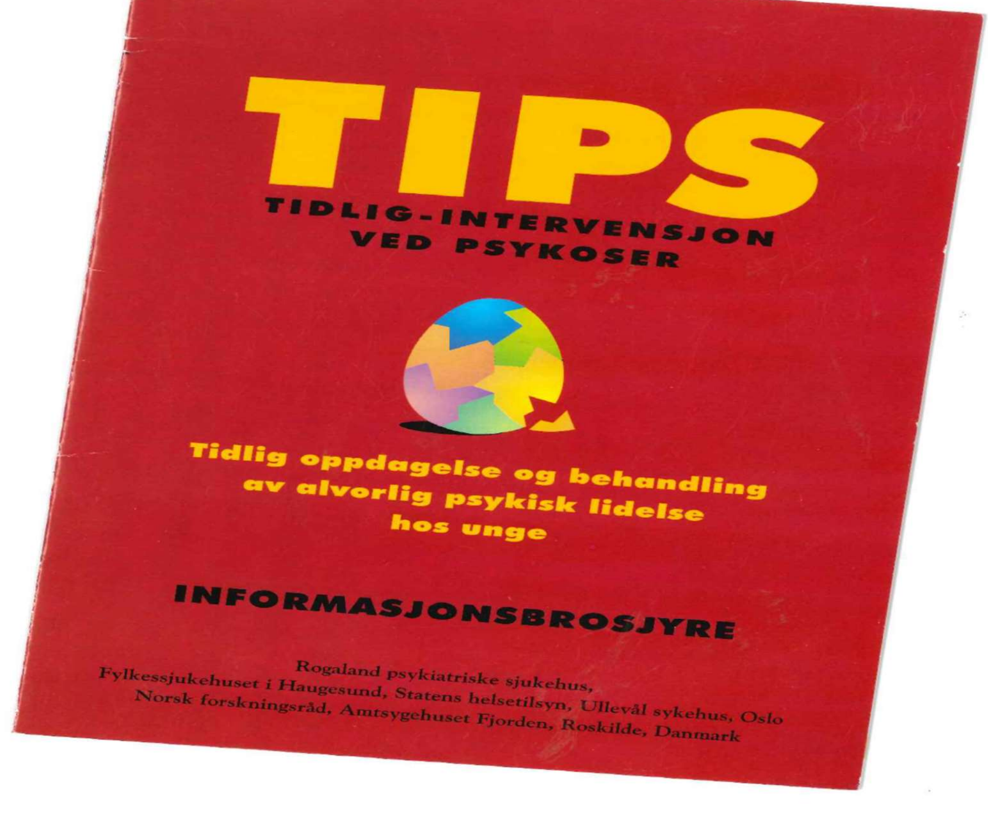
TIPS

TIDLIG-INTERVENSJON
VED PSYKOSER

Tidlig oppdagelse og behandling
av alvorlig psykisk lidelse
hos unge

INFORMASJONSBROSJYRE

Rogaland psykiatriske sjukehus,
Fylkessjukehuset i Haugesund, Statens helsetilsyn, Ullevål sykehus, Oslo
Norsk forskningsråd, Amtsygehuset Fjorden, Roskilde, Danmark

Denne brosjyren er en kortfattet informasjon om TIPS beregnet på publikum, pasienter og deres pårørende. Se ellers side 11 i denne brosjyren om aktuell litteratur/materiell i forbindelse med TIPS.

HVA ER PSYKOSER?

Psykoser kan betraktes som alvorlige "forvirringstilstander", og betegner egentlig svært dyptgående nervøse sammenbrudd. Hos unge mennesker kan slike sammenbrudd sette merker for livet, spesielt dersom det går lang tid fra sykdommen starter til pasienten får behandling.

Vi betrakter psykosen som en *prosess*, hvor det psykotiske *gjennombrudd* eller *sammenbrudd* er et stadium i sykdomsutviklingen. **Før** psykosen inntrer har pasienten ofte hatt mer generelle og uspesifike tegn på psykisk lidelse, som f.eks. søvnforstyrrelser, angst, depresjon og isolasjon.

Det vi vet er at det tar svært lang tid fra en person utvikler en slik alvorlig "forvirringstilstand" for første gang, til vedkommende får behandling. I Rogaland, som i landet forøvrig, kan det gå flere år fra de første sykdomstegn viser seg til pasienten får behandling i det psykiske helsevernet. Vi snakker om en *forsinkelse i behandlingen*. I gjennomsnitt er denne ca to år.

Vi mener at det er mulig å ***hindre, mildne eller forsinke*** utbruddet av en psykose dersom behandlingen starter på et tidligere tidspunkt i sykdomsutviklingen. Vi tror også at fremtidsutsiktene, *prognosen*, blir mye bedre når behandlingen settes inn tidlig. Selve behandlingen blir også sannsynligvis enklere og mer kortvarig.

Styringsgruppen for TIPS:

Per Vaglum (Universitetet i Oslo), leder, Jan Olav Johannessen (Rogaland psykiatriske sjukehus), nestleder/sekretær, Frode Larsen (Ullevål/Oslo kommune), Inger Kari Nærheim (Rogaland fylkeskommune), Ellen Hagemo (Stetens helsetilsyn), Thomas McGlashan (Yale, USA), Erik Simonsen (Danmark).

HVA ER TIPS?

TIPS (tidlig intervensjon ved psykose) står for tidlig oppdagelse og behandling ved slike alvorlige psykiske lidelser som beskrevet på side 2. TIPS retter seg spesielt inn mot unge mennesker som er i ferd med å utvikle slike lidelser for første gang. Hovedmålet er å **forkorte** den tiden det tar fra pasienter med psykose utvikler alvorlige symptomer til behandlingen starter.

Som en del av prosjektet vil pasientene bli tilbudt den behandling som betraktes som den riktigste og mest effektive. Hovedtrekkene i denne behandlingen beskrives i denne brosjyren.

Gjennom TIPS-prosjektet søker vi også å videreutvikle de psykiatriske helsetjenestene slik at vi kan gi hjelpen når den trengs mest og virker best, dvs. **før og i tilslutning til** det første utbruddet av alvorlig psykisk lidelse.

TIPS er et samarbeid mellom:

- Rogaland psykiatriske sjukehus
- Fylkessjukehuset i Haugesund, psykiatrisk avdeling
- Ullevål sykehus i Oslo, klinikk for psykiatri
- Amtssygehuset Fjorden, Roskilde, Danmark
- Yale Psychiatric Institute, New Haven, Connecticut, USA

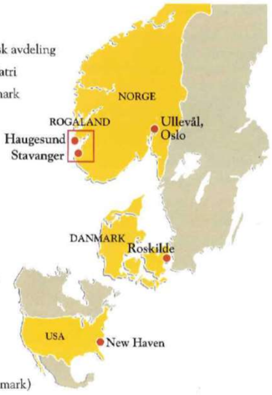

Økonomisk støttes prosjektet av:

- Norsk forskningsråd (Norge)
- Statens helsetilsyn (Norge)
- Rogaland fylkeskommune (Norge)
- Oslo kommune (Norge)
- Rogaland psykiatriske sjukehus (Norge)
- Fylkessjukehuset i Haugesund (Norge)
- Helsefonden (Danmark)
- Lægevidenskabelig Forskning ved Sygehusene i Region 3 (Danmark)
- Jansen Cilag og Lundbeck Pharma (Danmark)

VIKTIGE OPPGAVER FOR TIPS

1. Helsetjenesteutvikling

Gjennom en bevisst satsing på behandling i forbindelse med førstegangs-episoder av psykoser hos unge, søker en å hindre at pasienter blir kronisk syke og å bedre prognosen. TIPS innebærer en bevisst prioritering av behandlings-ressursene mot akutt sykdomsfase.

2. Kompetanseutvikling og metodeutvikling

En søker å utvikle kompetanse og metode innenfor behandling og publikums kjennskap til hvor man søker hjelp og på hvilket grunnlag man søker hjelp.

3. Informasjons-/opplysningsvirksomhet

a. Publikum

Via informasjonskampanjer i media; aviser, radio, kino og ulike informasjons-tiltak, søker en å bedre befolkningens kunnskapsnivå omkring tidlige tegn på alvorlig psykisk lidelse. En søker å opplyse om hvordan en kan få hjelp dersom en har problemer selv, eller kjenner noen som trenger utredning eller behandling.

b. Skoler

Vi vet at lærere, spesielt i den videregående skolen, er de som først oppdager tegn på at «noe er galt». TIPS tilbyr undervisning for sosiallærere, rådgivere, helsesøstre og andre som jobber i f.eks. oppfølgingstjenesten i den videregående skolen. I samarbeid med pedagogisk psykologisk rådgivningstjeneste vil alle lærere i den videregående skolen i Rogaland bli undervist i tidlige tegn på alvorlig psykisk lidelse hos unge, og hvor man henvender seg dersom man ønsker råd eller veiledning i forhold til enkelte elever.

c. Primærhelsetjenesten

Gjennom kurs for alle allmennleger og andre grupper av helsepersonell i primærhelsetjenesten, søker en å øke kunnskapen og bevissthetsnivået vedrørende tegn på psykose hos unge. Spesielt ønsker en å understreke tidsfaktoren og viktigheten av ikke å tape tid. Undervisningen består av seminarer med foredrag, visning av videoer og innføring av spesielt utarbeidede TIPS-manualer (treningsinstrumenter) som hjelpemidler i det å systematisk vurdere om en ny pasient har en begynnende psykose. Alt undervisningsmateriell er kanalisert og tilgjengelig gjennom Psykiatrisk Opplysningsfond, Breigaten 12, 4013 Stavanger.

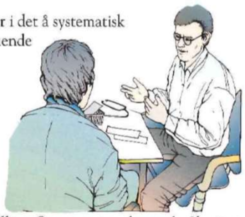

4. Samarbeid skole/ psykiatri

I det forebyggende helsearbeidet vil den videregående skolen ha en spesiell stilling. Som nevnt under punkt 3b, viser gode vitenskapelige undersøkelser at lærerne i videregående skoler er de første som oppdager tegn på at «noe er galt» hos elevene. Det er viktig med et tett, åpent og forpliktende samarbeid mellom skoler, skolemyndigheter, helsetjenester og helsemyndigheter med tanke på å utvikle gode samarbeidsrelasjoner og felles kunnskapsutvikling omkring psykiske lidelser hos unge. Fylkesopplæringskontoret og fylkeshelsesjefen i Rogaland er samarbeidspartnere i TIPS-prosjektet.

5. Samarbeid primærhelsetjeneste/psykiatri

En viktig årsak til at VUP (Varighet av Ubehandlet Psykose) er lang, dvs. at det skjer en **forsinkelse** fra unge mennesker begynner å utvikle psykiske problemer til behandling starter, er manglende samarbeid mellom primærhelse-tjenesten og spesialisthelsetjenesten. TIPS-prosjektet søker å legge til rette for en forpliktende, strukturert og jevnlig kontakt mellom primærhelsetjenesten og annenlinjetjenesten, og å gi prioritet til denne pasientgruppen i annenlinje-tjenesten. I tillegg til undervisning i forhold til primærhelsetjenesten, innebærer prosjektet regelmessige møter mellom representanter fra primærhelsetjeneste og spesialisthelsetjeneste.

6. Kvalitetssikring av behandlingen

Som ledd i kompetanseutvikling og metodeutvikling, søker en å tilby disse pasientene den beste behandlingen. Det er meningsløst å drive et tidlig interven-sjonsarbeid dersom pasientene f.eks. settes på venteliste. Vi garanterer derfor utredning innen 24 timer etter første henvendelse. Dersom pasienten har en psykose, garanterer vi behandling innen en uke. Den behandlingen som tilbys er basert på følgende hovedelementer:

• Medikamenter • Psykoterapi • Familiearbeid

Medikamenter

Gjennom systematisk arbeid med førstegangspsykoser er det de senere år blitt klart at medikamentdosene som har vært brukt opp til vår tid, ofte har vært for høye, og at en spesielt ved førstegangssykdom skal være svært forsiktig med de tradisjonelle antipsykotika (medisiner mot psykose). Som ledd i det arbeid som allerede er gjort, har vi redusert anbefalt dose av medisiner betraktelig. Alle pasienter som inkluderes i prosjektet vil bli tilbudt en standard behandlingspakke hvor lavest mulig medikamentdose er en del.

Psykoterapi

Psykoterapi av unge mennesker generelt, og psykoser/psykosenære tilstander spesielt, stiller store krav til behandleren. Det nytter lite kun å sitte på kontoret å vente på at pasienten skal komme. Behandlingen krever en aktiv innstilling med evne og vilje til å ta stort ansvar for pasienten. Samtidig representerer disse pasientene store behandlingsmessige utfordringer, og vi vil gjerne at våre mest erfarne psykoterapeuter skal ha et spesielt ansvar for denne pasientgruppen. Behandlingspakken sikrer psykoterapi i minst to år, en gang ukentlig som et minimum.

Familiearbeid

Vi vet at et godt familiearbeid kanskje er det mest effektive element i en slik behandlingspakke. Selv om denne kunnskapen har vært tilgjengelig i 15 år nå, har det vært svært vanskelig å få integrert familiearbeidet som en del av det løpende tilbudet i psykiatriske avdelinger. Et overordnet mål med TIPS-prosjektet er å legge til rette for at familiearbeid går inn i standardtilbudet fra avdelingen i forhold til pasient og familie. Familiearbeidet er tredelt:

- **Familie TIPS-dag**

  Gjennom en hel seminardag gis familiene oversikt over psykiatriske lidelser, årsaksforhold, behandlingsformer, prognose osv.

- **Individuelle familietilbud**

  Alle familiene vil få tilbud om samtaler ved behandlerne.

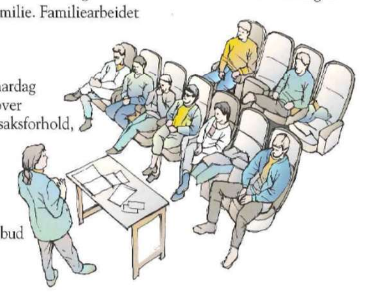

- **Multifamiliegrupper**

En svært effektiv metode som familiene setter stor pris på er deltakelse i flerfamiliegrupper. I samarbeid med professor William McFarlane fra Maine i USA, har vi utviklet et spesielt tilbud til familiene til pasienter som har en førstegangspsykose.

7. Evaluerings-/forskningsvirksomhet

Det er viktig at et så stort forsøk som her gjøres også har en evaluerings/-kvalitetssikringsside. I samarbeid med Ullevål sektor i Oslo (Universitetet i Oslo), Roskilde i Danmark og Yale Psychiatric Institute i USA, har TIPS-forsøket også en forskningsside. Vi skal sammenligne Rogaland fylke med Ullevål sektor i Oslo og Roskilde i Danmark. Rogaland fylke blir «forsøksfylke» hvor vi setter mye inn på å redusere VUP (varighet av ubehandlet psykose). Vi tror at dersom vi klarer å redusere VUP, vil kort- og langtidsutsiktene til pasientene bli bedre. I Ullevål og i Roskilde skal man ikke gjøre noe spesielt for å komme tidligere til med behandling. Behandlingen er lik på alle stedene. På denne måten kan vi kontrollere for om det har noen betydning at vi klarer å redusere VUP. Forskningsprosjektet vil vare til år 2005.

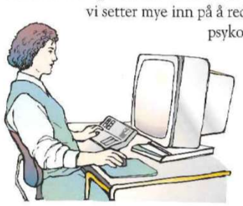

8. Samarbeid

Rogaland fylkeskommune, Oslo kommune, Roskilde Amt og det norske Helse- og Sosialdepartement har alle gått inn med støtte til prosjektet. Totalt innebære satsingen tilførsel av ca. 50 mill. kroner til arbeidet med tidlig oppdagelse og behandling av psykoser hos unge i en femårs periode. TIPS er på mange måter et nybrottsarbeid, men bygger likevel på tidligere erfaringer gjort i England, Australia og også her i landet. Disse erfaringene peker alle i retning av at tidlig intervensjon er en riktig strategi.

Struktur

Oppdagelsesteam (OT)

For å komme tidligere til med behandlingen i Rogaland har vi etablert et oppdagelsesteam (OT) som består av i alt fem fagpersoner i full stilling som skal ha som hovedoppgave å utrede pasienter som enten har utviklet en psykose eller som er i ferd med å utvikle en slik tilstand. OT har vakt fem dager i uken mellom 0800 og 1530. Utover disse tider er det mulig å legge igjen melding på telefonsvareren.

Teamet er delt i to. I nordfylket (Haugesundsregionen) ledes teamet av sjefpsykolog Sigurd Mardal, og i sørfylket (Stavanger-Sandnes-regionen) av seksjonsoverlege på poliklinikken i Stavanger, psykiater Marthe Horneland.

Alle, både pasienter, pårørende, allmennleger, lærere, bekjente osv., kan i prinsippet kontakte OT. OT vil i en del tilfeller gi råd pr. telefon, noen ganger tilby time til utredning på sykehuset, men gjør også hjemmebesøk eller kan har samtaler med pårørende på legens eller skolens kontor.

Evalueringsteam (ET)

De pasientene som, etter å ha vært vurdert av oppdagelsesteamet, blir inkludert i TIPS-forsøket, vil bli tilbudt en omfattende utrednings- og behandlingspakke. Flere fagpersoner ved Rogaland psykiatriske sjukehus, Ullevål sjukehus og i Roskilde, har fått spesialtrening i slik utredning. Utredningen innebærer ingen spesielle nye forsøk, men legger vekt på en god diagnostikk, slik at pasienten kan tilbys den beste behandling. Pasientene vil bli fulgt opp i fem år.

Prosjektkoordinatorer:
Dr. Ingrid Melle (Oslo)
Dr. Ulrik Haahr (Danmark)
Dr. Tor .K. Larsen (Rogaland)

Aktuell litteratur/materiell i forbindelse med TIPS

- **TIPS-Stor manual med skåringsskjema**
- **TIPS- Studieprotokoll**
- **TIPS- Skåringsinstrumenter**
- **TIPS- Registreringsskjema hovedbehandler**
- **TIPS-Tidlig intervensjon ved psykose** Undervisningssett/lysark
- **TIPS- informasjonsbrosjyre publikum**
- **Hva er det med Monica?** TIPS-video 1
- **Begynnende psykose** TIPS-video 2
- **Det er noe som ikke er som det skal være** - Brosjyre
- **Pakke 1 (Skolepakke)** 1 stk. Undervisningssett, 1 stk video "Hva er det med Monica" og 50 stk. "Det er noe som ikke er som det skal være"
- **Pakke 2 (Legepakke)** 1 stk. Undervisningssett, 1 stk. video "Begynnende psykose" og 1 stk. manual med skåringsskjema
- **Scizofreni** Opplysningshefte for pasienter, pårørende og publikum (Psykiater Gerg-Ragna Bloch Thorsen)

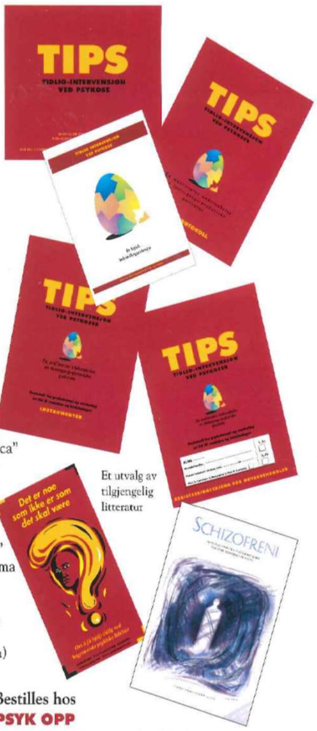

Et utvalg av tilgjengelig litteratur

Bestilles hos
PSYK OPP
Breigt. 21, 4006 Stavanger, tlf. 51 89 03 50 E-post. psykopp@online.no

TIPS

TIDLIG-INTERVENSJON VED PSYKOSER

Det er et stort problem at mange pasienter som utvikler psykoser ikke oppdages og får behandling før etter lang tids sykdom. Med grunnlag i denne kjennsgjerningen forventer vi at behandlingen som gis kan få bedre effekt dersom vi kan komme tidligere til i sykdomsforløpet.
TIPS-prosjektet har dette som utgangspunkt.

Prosjektet engasjerer en stor gruppe erfarne klinikere over lang tid. Dette innebærer en betydelig satsing på behandlingen av pasienter i de aktuelle regionene. TIPS-prosjektet er helsetjenesteutvikling i praksis.

Knyttet til TIPS-prosjektet er også en forskningsdel hvor vi løpende vil evaluere resultatene av denne spesielle satsingen.

Vi håper at TIPS-prosjektet kan peke på betydningen av tidlig intervensjon ved alvorlige "forvirringstilstander" som psykoser, og at modellen kan bidra til en videreutvikling av vår psykiatriske helsetjeneste.

DESIGN ELLEN JEPSON

BAKGRUNN FOR TIPS

De alvorlige "forvirringstilstandene", psykosene, som f.eks. schizofreni, representerer behandlingsmessig den største utfordringen i psykiatrien. Dette er tilstander hvor den som rammes ofte har det vondt svært lenge, og sykdommen medfører alvorlige følger for den enkelte pasient og for familien. Vi vet at det likevel er mulig å tilby effektiv behandling og at mange kan bli helbredet.

De psykiske lidelsene, og spesielt **schizofreni**, utgjør enorme belastninger for behandlingsapparatet og påfører samfunnet store kostnader. Man har anslått at schizofreni alene koster det norske samfunn opp til 5 milliarder kroner pr. år og er den mest kostbare enkeltlidelse uansett kategori. Schizofreni alene koster samfunnet mer enn alle hjertesykdommer, kreftsykdommer eller andre sammenlignbare sykdomsgrupper.

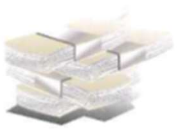

Samfunnskostnader kreft

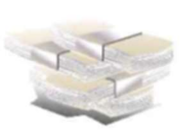

Samfunnskostnader hjertelidelser

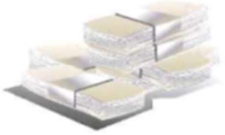

Samfunnskostnader schizofreni

En har ved Rogaland psykiatriske sjukehus i flere tiår arbeidet med systematisering av behandlingstilbudet til unge schizofrene. På tross av den store lidelsen knyttet til tilstanden, og den belastning det innebærer for den enkelte og samfunnet, har det dessverre lett for å bli slik at denne pasientgruppen blir nedprioritert.

I utgangspunktet er schizofreni en sjeldent forekommende sykdom. I en by som Stavanger med 100.000 innbyggere vil vi få ca. 10 nye tilfeller pr. år. Dersom vi tilbyr disse en omfattende behandling i akuttfasen, vil vi kunne redusere senfølgene av sykdommen betraktelig.

Undersøkelser fra Rogaland psykiatriske sjukehus (TIPS-1 prosjektet 1992-1996) viste at varighet av ubehandlet psykoser (VUP) var 2,1 år fra psykosen startet til adekvat behandling ble gitt. Hovedårsaken til at pasientene kom så sent til behandling var flere. Bl.a. ble de ikke oppfattet som alvorlig mentalt syke når de første gang kom i kontakt med primærhelsetjenesten. Det så også ut til at annenlinjetjenesten, representert ved de psykiatriske poliklinikkene og det psykiatriske sjukehuset, ikke var flinke nok til å stille diagnosene så tidlig som mulig, og derigjennom tilby adekvat behandling.

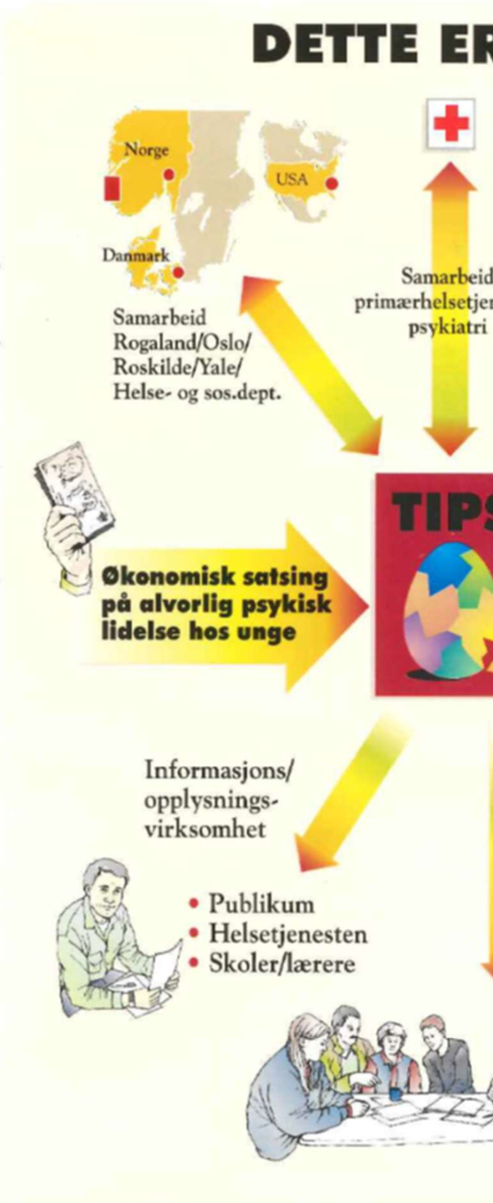

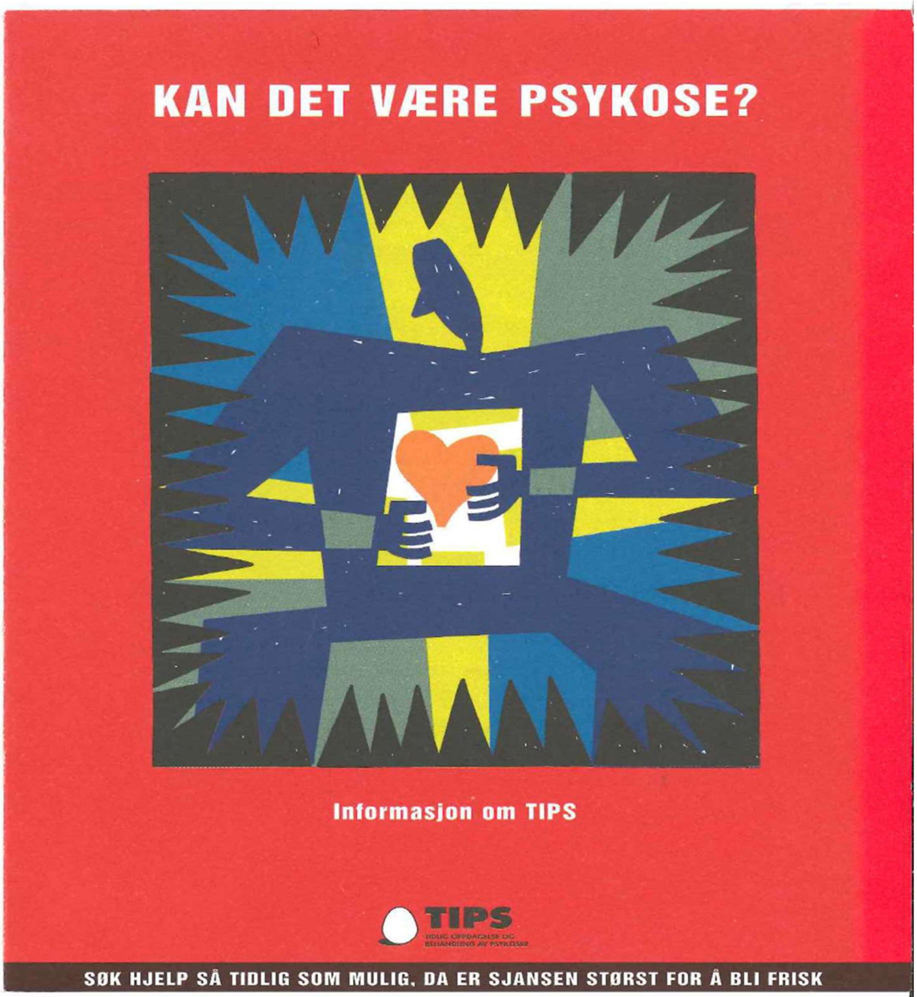
KAN DET VÆRE PSYKOSE?
Informasjon om TIPS
TIPS
SØK HJELP SÅ TIDLIG SOM MULIG, DA ER SJANSEN STØRST FOR Å BLI FRISK

Brosjyre s. 62-65

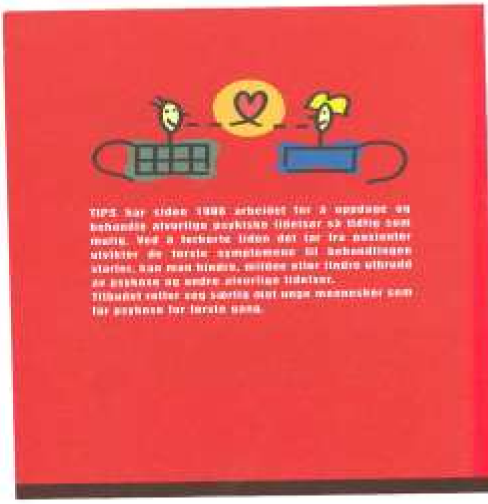

Folkeopplysning

Forutsetningen for å søke hjelp tidlig, er at folk flest kjenner symptomene på sykdommen. TIPS har drevet opplysningsarbeid helt siden starten, med avisannonser, brosjyrer, radio-, TV- og internettannonsering.
Målet var å gjøre hjelpetjenesten kjent, fortelle om symptomer, ta livet av myter omkring psykiatrien og - selvsagt - få folk til å søke hjelp. Gjennom hjelpetelefonen får både publikum, allmennleger, lærere, helse- og sosialarbeidere direkte kontakt med fagfolk som gir hjelp eller råd om hva de skal foreta seg.

Styrket hjelpeapparat

Før TIPS ble allment kjent, hadde man siden 1993/94 gjennomført forstudier og utarbeidet diagnoseverktøy og undervisningsopplegg for allmennleger, lærere, helse- og sosialarbeidere. Særlig elever i den videregående skole er en viktig målgruppe, og det lages egne kampanjer for disse.
Som et ledd i prosjektet blir pasientene tilbudt den behandling som betraktes som den riktigste og mest effektive.

Tidlig OppdagelsesTeam (OT)

Når noen ringer TIPS, vil de møte en medarbeider i OppdagelsesTeamet(OT). OT består av psykiatere, psykologer og psykiatriske sykepleiere. Dersom det er grunn til å mistenke en mulig psykoseproblematikk, vil OT-representanten møte pasienten, eller den som har henvist pasienten. Vi tilstreber at dette møtet holdes der det passer best, enten dette er hjemme, på skolen, hos allmennlegen eller på sosialkontoret.
OppdagelsesTeamet ble etablert i januar 1997 i Rogaland med to team, et i nord- og et i sør-fylket. Ved Stavanger Universitetssjukehus, Psykiatrisk klinikk er oppdagelsesteamet lokalisert ved akutt-mottakspost. I Helse Fonna er oppdagelsesteamet lokalisert ved Haugesund sjukehus. Også Helse Øst har etablert et ressurssenter for tidlig oppdagelse ved Ullevål.

24 timers utredningsgaranti

OppdagelsesTeamet gir en "24 timers utredningsgaranti". OT utfører blant annet PANSS-intervju. Dette intervjuet vil kunne avdekke tilstedeværelse av psykosesymptomer som vrangforestillinger, tankeforstyrrelser, hallusinasjoner, samt at en vurderer negative symptomer som passivitet, isolasjon og tilbaketrekking. I de tilfeller utredningen konkluderer med at det sannsynligvis foreligger en psykotisk lidelse, vil pasienten bli henvist til behandling og en mer omfattende, vitenskapelig basert utredning og diagnostisering.

Forskning

God behandling er basert på solid kunnskap og ikke bare synsing og ideologiske overbevisnin[illegible]
Forskningen styrker vår evne til å være kritiske, og den hjelper oss til å utvikle bedre behandlings[illegible]deller for pasientene.

Forskningsdelen er konsentrert om effekten av tidlig intervensjon - oppdagelse og behandling. Funn[illegible] i Tidlig Oppdagelsesektoren i Rogaland, er at vi som det første kliniske tidlig intervsjonsprosjek[illegible] verden (!), har lykkes i å komme svært tidlig til med behandling. Varighet av ubehandlet psykose [illegible] snitt 5 uker. Når vi sammenlikner med sektorene som ikke hadde tidlig-oppdagelse i denne perio[illegible] (Oslo og Roskilde i Danmark) finner vi at pasientene som oppdages tidlig, har mindre symptome[illegible] bedre behandlingseffekt i løpet av de 3 første månedene. Så langt har vi lært at det er mulig å kom[illegible] svært tidlig til med behandling selv om lidelsen heter schizofreni eller alvorlig psykose.
I årene som kommer skal vi se på langtidsforløpet av pasientene som ble inkludert i TIPS. Vi drive[illegible] nye prosjekter knyttet opp til denne organisasjonen. TIPS-II prosjektet er en ny studie på første ga[illegible] psykoser hvor vi studerer hva som skjer med varighet av ubehandlet psykose når vi ikke lenger [illegible] intensive mediakampanjer. TOPP-prosjektet er en studie på mennesker med mulig prepsykose. SNA[illegible] prosjektet er en studie på språk og psykose som gjennomføres i samarbeid med senteret [illegible] Leseforskning ved UiS og Universitet i Lund, Sverige.

TIPS er et samarbeid mellom: Stavanger Universitetssjukehus, psykiatrisk klinikk, Psykiatrisk kli[illegible] Sjukehuset i Haugesund, Helse Fonna, Ullevål sykehus i Oslo, klinikk for psykiatri, Amtssygehu[illegible] Fjorden, Roskilde, Danmark og Yale Psychiatric Institute, New Haven, Connecticut, USA.

Folkehelse og samfunnsøkonomi

De alvorligste psykiske lidelsene, og spesielt schizofreni, er svært ressurskrevende for behandlings[illegible]paratet. Man har anslått at schizofreni alene koster det norske samfunn opp til 5 milliarder kroner [illegible] år, og det er den mest kostbare enkeltlidelse uansett kategori. Schizofreni koster samfunnet mer [illegible] alle hjertesykdommer, kreftsykdommer eller andre sammenlignbare sykdomsgrupper.

I en by som Stavanger med 100.000 innbyggere vil vi få ca. 70 nye tilfeller pr. år med førstega[illegible] psykose. Dersom vi tilbyr disse en omfattende behandling i akuttfasen, vil vi kunne redusere sen[illegible]gene av sykdommen betraktelig og samtidig redusere utgiftene til behandling.

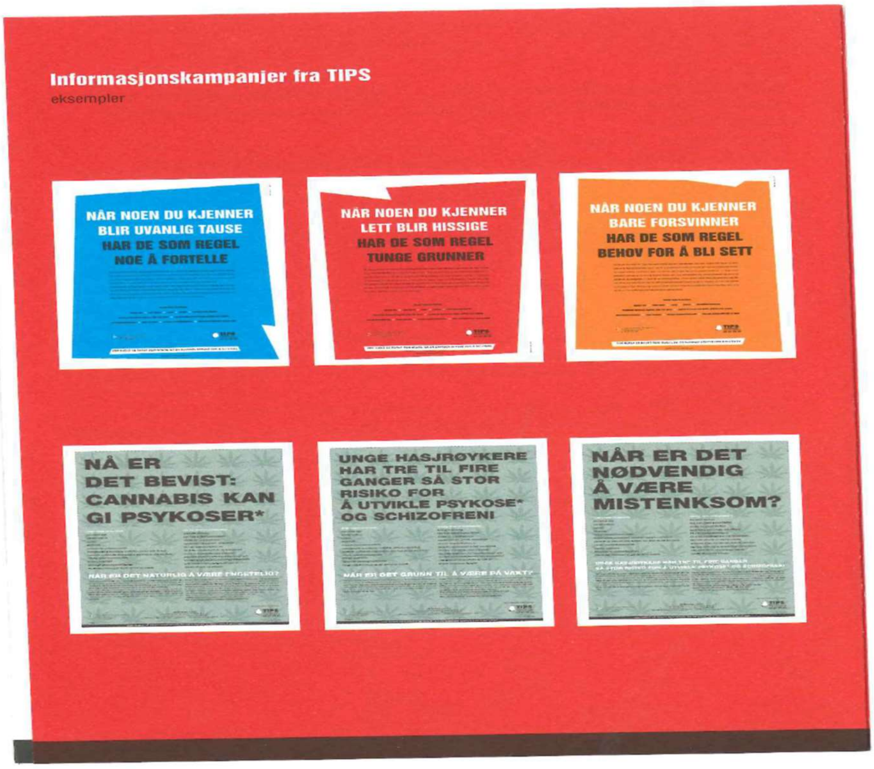
Informasjonskampanjer fra TIPS
eksempler
NÅR NOEN DU KJENNER BLIR UVANLIG TAUSE HAR DE SOM REGEL NOE Å FORTELLE
NÅR NOEN DU KJENNER LETT BLIR HISSIGE HAR DE SOM REGEL TUNGE GRUNNER
NÅR NOEN DU KJENNER BARE FORSVINNER HAR DE SOM REGEL BEHOV FOR Å BLI SETT
NÅ ER DET BEVIST: CANNABIS KAN GI PSYKOSER*
UNGE HASJRØYKERE HAR TRE TIL FIRE GANGER SÅ STOR RISIKO FOR Å UTVIKLE PSYKOSE* OG SCHIZOFRENI
NÅR ER DET NØDVENDIG Å VÆRE MISTENKSOM?

Takk til alle som deltar

Alle som på en eller annen måte bidrar til at TIPS øker sitt erfaringsgrunnlag er med på å gjøre tjenestene innen psykiatrien bedre. Dette inkluderer både de som benytter seg av tjenestene og de som bidrar til at mennesker får tidlig hjelp.

Vil du vite mer?

En viktig side av prosjektet har vært å få resultatene publisert og diskutert i nasjonale og internasjonale fora. I løpet av de siste årene er det publisert totalt ca 70 artikler/bokkapitler fra TIPS-prosjektet, vi har to nye artikler i trykken, fire innsendt for vurdering og flere er under utarbeidelse. Den siste artikkelen er publisert i augustnummeret 2005 av British Journal of Psychiatry og i år kommer det en artikkel om tidlig behandling og redusjon av suicidalitet i American Journal of Psychiatry (mai-nummeret). En oversikt over artikler og andre publikasjoner finnes på www.tips-info.com

Med hilsen

Jan Olav Johannessen
Sjeflege
Psykiatrisk Klinikk

T.K. Larsen
1. amanuensis dr med.
TIPS

Inge Joa
Koordinator
TIPS 2

Karl Lie
psykiatrisk sykepleier

Rune Kvebæk
psykiatrisk sykepleier

Nord Rogaland

Helse Fonna

TIPS

52 73 27 00
hverdager 08.00 - 15.00

Sør Rogaland

Stavanger Universitetssjukehus
Helse Stavanger HF
Psykiatrisk klinikk

TIPS

51 51 59 59
hverdager 08.00 - 15.00

www.tips-info.com

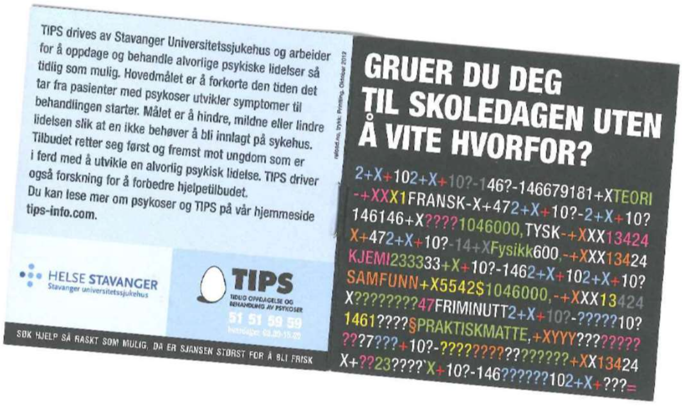
TIPS drives av Stavanger Universitetssjukehus og arbeider for å oppdage og behandle alvorlige psykiske lidelser så tidlig som mulig. Hovedmålet er å forkorte den tiden det tar fra pasienter med psykoser utvikler symptomer til behandlingen starter. Målet er å hindre, mildne eller lindre lidelsen slik at en ikke behøver å bli innlagt på sykehus. Tilbudet retter seg først og fremst mot ungdom som er i ferd med å utvikle en alvorlig psykisk lidelse. TIPS driver også forskning for å forbedre hjelpetilbudet.
Du kan lese mer om psykoser og TIPS på vår hjemmeside tips-info.com.
HELSE STAVANGER
Stavanger universitetssjukehus
TIPS
TIDLIG OPPDAGELSE OG BEHANDLING AV PSYKOSER
51 51 59 59
SØK HJELP SÅ RASKT SOM MULIG, DA ER SJANSEN STØRST FOR Å BLI FRISK
GRUER DU DEG TIL SKOLEDAGEN UTEN Å VITE HVORFOR?
2+X+102+X+10?-146?-146679181+XTEORI
-+XXX1FRANSK-X+472+X+10?-2+X+10?
146146+X????1046000,TYSK-+XXX13424
X+472+X+10?-14+XFysikk600,-+XXX13424
KJEMI233333+X+10?-1462+X+102+X+10?
SAMFUNN+X5542$1046000,-+XXX13424
X????????47FRIMINUTT2+X+10?-?????10?
1461????§PRAKTISKMATTE,+XYYY????????
????7???+10?-?????????????????+XX13424
X+??23????`X+10?-146??????102+X+???=

MANGE GRUER SEG TIL SKOLEDAGEN

Manger unge føler at de sliter psykisk i perioder. Heldigvis er det forbigående for de fleste, og problemene har som oftest en naturlig forklaring. Det kan være problemer med venner og familie eller at en er skolelei. Da hjelper det å snakke med dine nærmeste, eller læreren hvis det har med skolen å gjøre.

Noen ganger er det vanskelig å sette ord på de følelsene en har, og vanskelig å forklare hvordan en egentlig har det. Resultatet blir gjerne at en isolerer seg, blir taus og irritabel, eller sliter med tunge tanker. Når slike symptomer varer i uker eller måneder, kan det være starten på alvorlige psykiske lidelser. Da er det viktig å snakke med fagfolk.

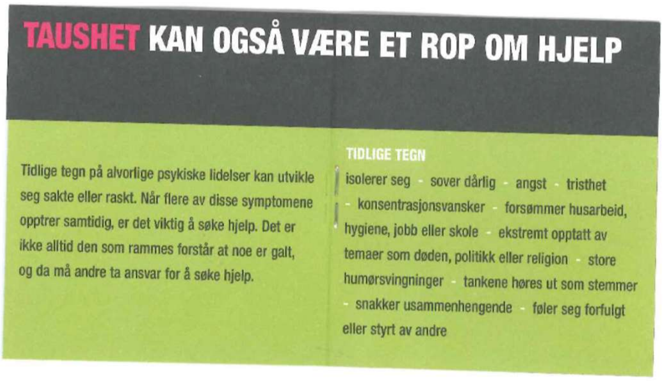

TAUSHET KAN OGSÅ VÆRE ET ROP OM HJELP

Tidlige tegn på alvorlige psykiske lidelser kan utvikle seg sakte eller raskt. Når flere av disse symptomene opptrer samtidig, er det viktig å søke hjelp. Det er ikke alltid den som rammes forstår at noe er galt, og da må andre ta ansvar for å søke hjelp.

TIDLIGE TEGN

isolerer seg · sover dårlig · angst · tristhet · konsentrasjonsvansker · forsømmer husarbeid, hygiene, jobb eller skole · ekstremt opptatt av temaer som døden, politikk eller religion · store humørsvingninger · tankene høres ut som stemmer · snakker usammenhengende · føler seg forfulgt eller styrt av andre

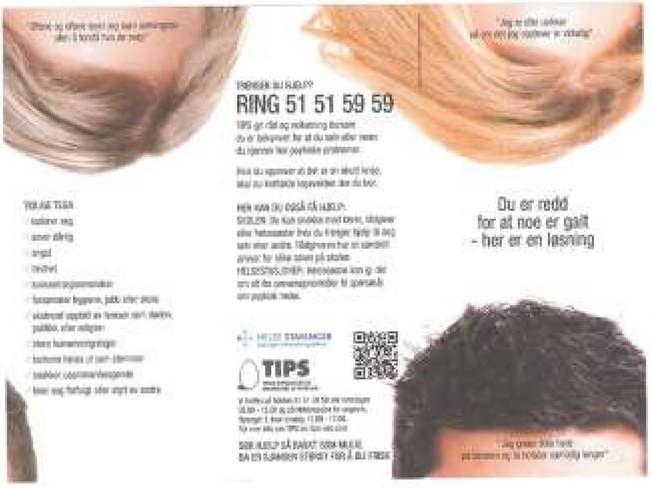

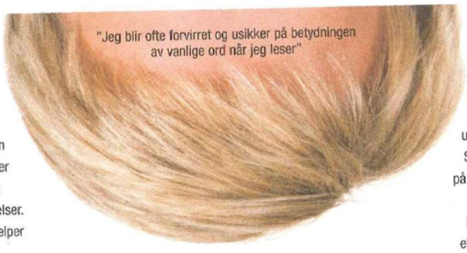

Mange unge sliter psykisk. For de fleste er det forbigående, og problemene har en naturlig forklaring. Det kan være konflikter med venner og familie, en dårlig periode på skolen eller det kan handle om skuffelser. Det er normalt å møte motstand. Ofte hjelper det å snakke med sine nærmeste eller en lærer hvis det handler om skolen.

Hvis en har det slik i flere uker kan det være grunn til bekymring. Slike opplevelser kan være tidlige tegn på en psykisk lidelse og bør undersøkes.

Psykiske lidelser kan utvikle seg sakte eller raskt. Når flere slike symptomer er til stede samtidig er det lurt å søke hjelp.

En del opplever også underlige og kanskje skremmende tanker, inntrykk og følelser som de ikke tør å fortelle til andre, selv ikke til sin egen familie.

Slike opplevelser kommer ofte i perioder der en opplever mye stress og sover dårligere enn vanlig. Tunge tanker er ofte vanskelige å dele med andre. Resultatet blir ofte at en isolerer seg, blir taus og irritabel.

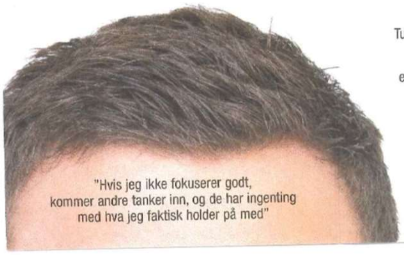

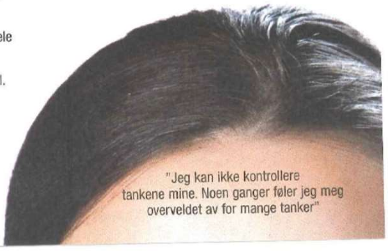

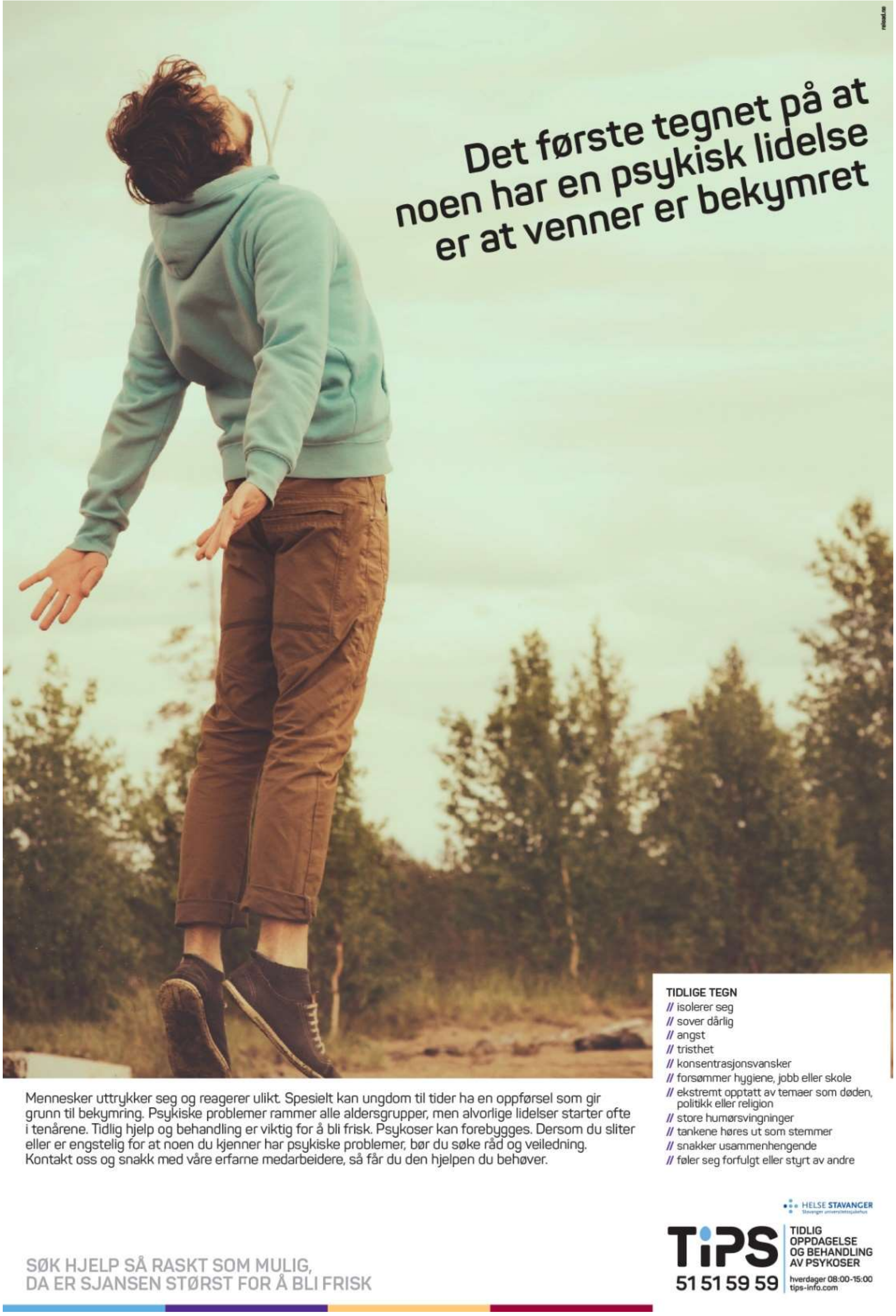
Det første tegnet på at
noen har en psykisk lidelse
er at venner er bekymret
TIDLIGE TEGN
// isolerer seg
// sover dårlig
// angst
// tristhet
// konsentrasjonsvansker
// forsømmer hygiene, jobb eller skole
// ekstremt opptatt av temaer som døden, politikk eller religion
// store humørsvingninger
// tankene høres ut som stemmer
// snakker usammenhengende
// føler seg forfulgt eller styrt av andre
Mennesker uttrykker seg og reagerer ulikt. Spesielt kan ungdom til tider ha en oppførsel som gir grunn til bekymring. Psykiske problemer rammer alle aldersgrupper, men alvorlige lidelser starter ofte i tenårene. Tidlig hjelp og behandling er viktig for å bli frisk. Psykoser kan forebygges. Dersom du sliter eller er engstelig for at noen du kjenner har psykiske problemer, bør du søke råd og veiledning. Kontakt oss og snakk med våre erfarne medarbeidere, så får du den hjelpen du behøver.
SØK HJELP SÅ RASKT SOM MULIG,
DA ER SJANSEN STØRST FOR Å BLI FRISK
HELSE STAVANGER
TiPS
TIDLIG OPPDAGELSE OG BEHANDLING AV PSYKOSER
51 51 59 59
hverdager 08:00-15:00
tips-info.com

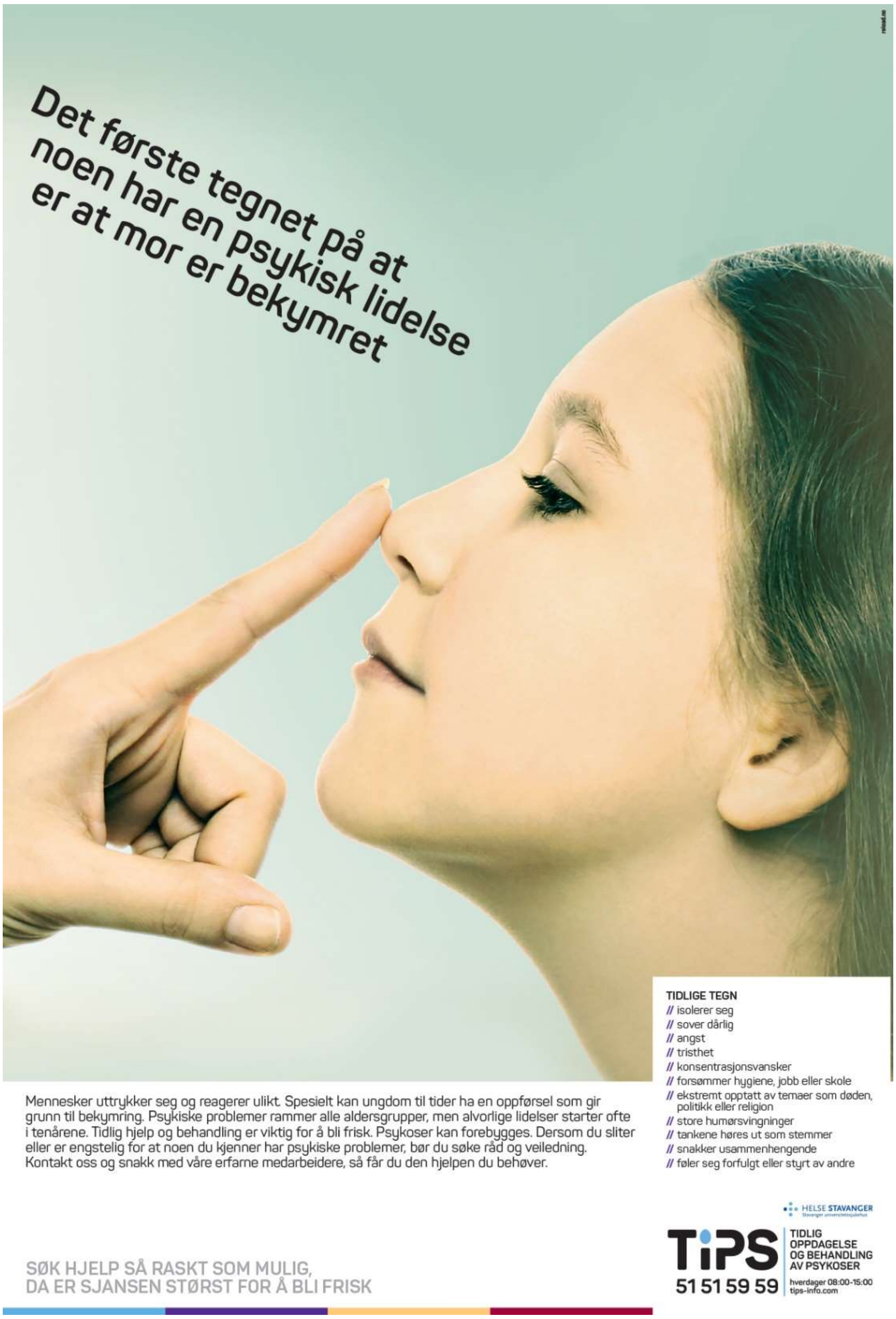
Det første tegnet på at
noen har en psykisk lidelse
er at mor er bekymret
TIDLIGE TEGN
// isolerer seg
// sover dårlig
// angst
// tristhet
// konsentrasjonsvansker
// forsømmer hygiene, jobb eller skole
// ekstremt opptatt av temaer som døden, politikk eller religion
// store humørsvingninger
// tankene høres ut som stemmer
// snakker usammenhengende
// føler seg forfulgt eller styrt av andre
Mennesker uttrykker seg og reagerer ulikt. Spesielt kan ungdom til tider ha en oppførsel som gir grunn til bekymring. Psykiske problemer rammer alle aldersgrupper, men alvorlige lidelser starter ofte i tenårene. Tidlig hjelp og behandling er viktig for å bli frisk. Psykoser kan forebygges. Dersom du sliter eller er engstelig for at noen du kjenner har psykiske problemer, bør du søke råd og veiledning. Kontakt oss og snakk med våre erfarne medarbeidere, så får du den hjelpen du behøver.
HELSE STAVANGER
TIPS
TIDLIG OPPDAGELSE OG BEHANDLING AV PSYKOSER
51 51 59 59
hverdager 08:00-15:00
tips-info.com
SØK HJELP SÅ RASKT SOM MULIG,
DA ER SJANSEN STØRST FOR Å BLI FRISK

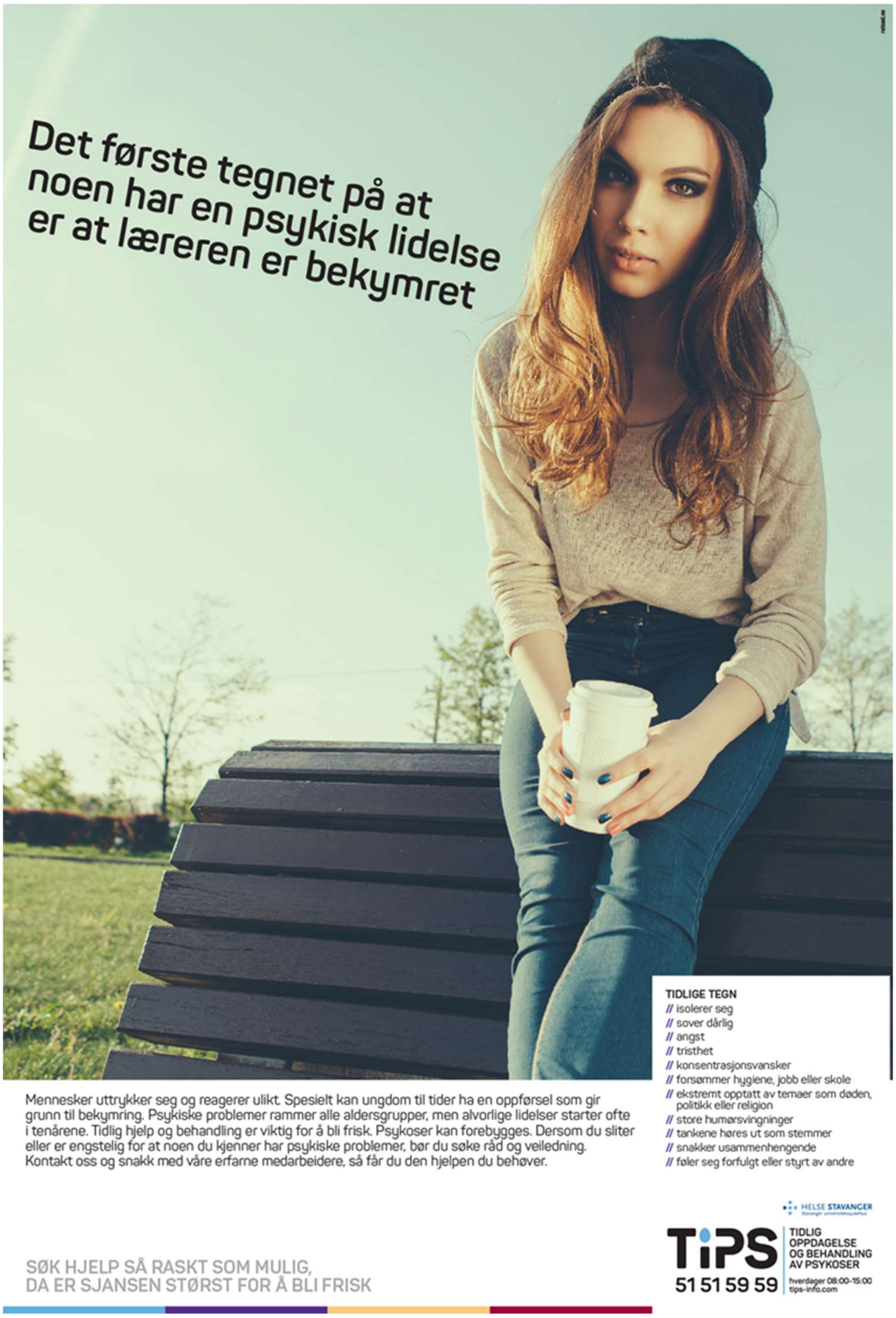
Det første tegnet på at noen har en psykisk lidelse er at læreren er bekymret
TIDLIGE TEGN
// isolerer seg
// sover dårlig
// angst
// tristhet
// konsentrasjonsvansker
// forsømmer hygiene, jobb eller skole
// ekstremt opptatt av temaer som døden, politikk eller religion
// store humørsvingninger
// tankene høres ut som stemmer
// snakker usammenhengende
// føler seg forfulgt eller styrt av andre
Mennesker uttrykker seg og reagerer ulikt. Spesielt kan ungdom til tider ha en oppførsel som gir grunn til bekymring. Psykiske problemer rammer alle aldersgrupper, men alvorlige lidelser starter ofte i tenårene. Tidlig hjelp og behandling er viktig for å bli frisk. Psykoser kan forebygges. Dersom du sliter eller er engstelig for at noen du kjenner har psykiske problemer, bør du søke råd og veiledning. Kontakt oss og snakk med våre erfarne medarbeidere, så får du den hjelpen du behøver.
HELSE STAVANGER
TIPS
TIDLIG OPPDAGELSE OG BEHANDLING AV PSYKOSER
51 51 59 59
hverdager 08:00-15:00
tips-info.com
SØK HJELP SÅ RASKT SOM MULIG, DA ER SJANSEN STØRST FOR Å BLI FRISK

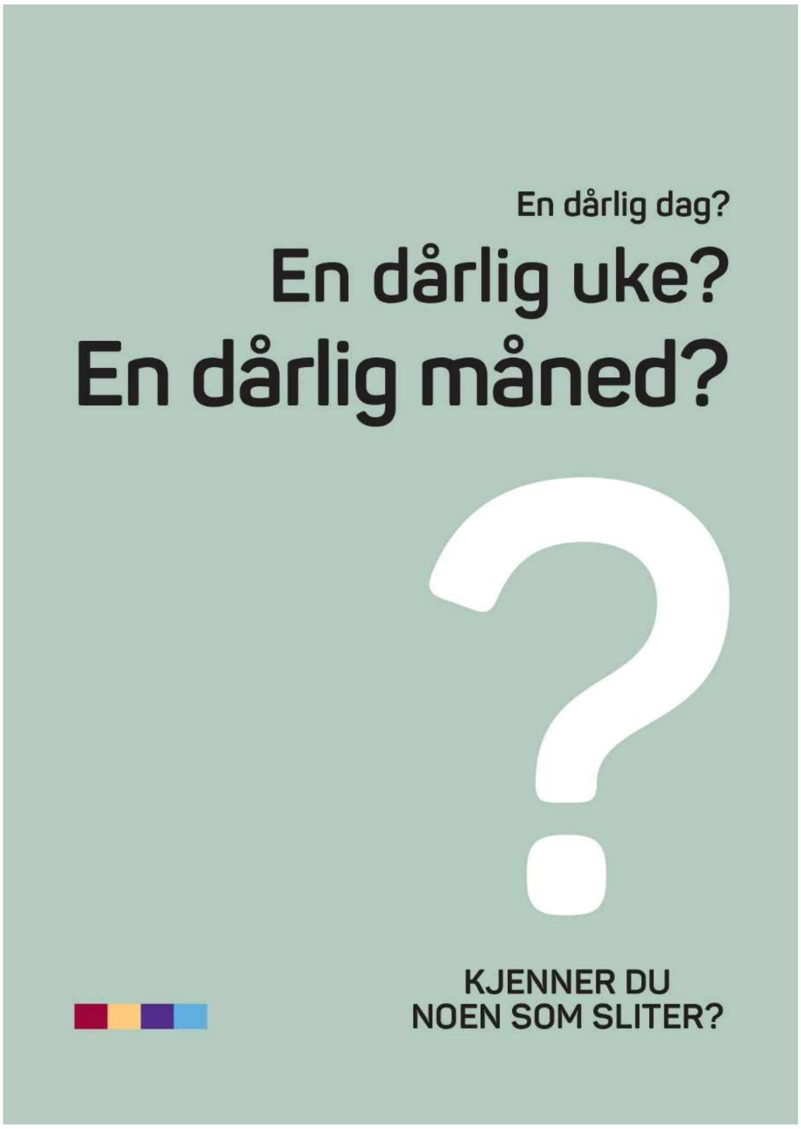
En dårlig dag?
En dårlig uke?
En dårlig måned?
KJENNER DU
NOEN SOM SLITER?

Har du det bra?

I følge Ungdata-undersøkelsen er dagens ungdom mer veltilpasset, ruser seg mindre og klarer seg bedre enn sin foreldregenerasjon. Samtidig har de utfordringer. De nevner en følelse av krav både i forhold til skole og hvordan man skal ta seg ut i forhold til omverdenen. Det er et krav om å fremstå vellykket og ikke være svak. Helsedirektoratet anslår at om lag 70 000 barn og unge har psykiske lidelser som krever behandling. Dessverre tar de som har problemer sjelden kontakt selv. De vil ikke skille seg ut og skjuler problemene.

Det er vanlig å skille mellom psykiske plager og psykiske lidelser. Med psykiske plager mener vi tilstander som oppleves som belastende, men ikke i så stor grad at de karakteriseres som diagnoser. Det er belastninger som går over. Betegnelsen psykiske lidelser brukes bare når bestemte diagnostiske kriterier er oppfylt. 15–20 % av barn mellom tre og 18 år har nedsatt funksjon på grunn av symptomer på psykiske lidelser som angst, depresjon og atferdsforstyrrelser. Cirka halvparten har så alvorlige symptomer at det tilfredsstiller kravene til en psykiatrisk diagnose. De aller fleste av disse trenger behandling.

Endret oppførsel som bør gi grunn til å søke hjelp

// Trekker seg tilbake fra familie og venner
// Isolerer seg
// Sover dårlig og spiser lite
// Er ekstremt opptatt av et spesielt tema, som f. eks. døden, politikk eller religion
// Forsømmer personlig hygiene
// Oppnår dårligere resultater på skolen
// Har problemer med å konsentrere seg og huske ting
// Snakker om, eller skriver ting, som ikke gir noen mening
// Får panikk, er ekstremt engstelig, merkbart deprimert eller har selvmordstanker
// Mister variasjon i humøret
// Mangler uttrykk for følelser
// Har markerte svingninger i humøret
// Har upassende følelsesmessige reaksjoner i forhold til alder
// Hører stemmer som ingen andre kan høre
// Tror, uten grunn, at andre lager komplott mot, spionerer på eller følger etter en
// Tror at en blir skadet, eller påvirket til å gjøre ting mot ens vilje, av fjernsyn, djevelen etc.
// Tror at en har spesielle evner
// Tror at tankene blir påvirket eller at en kan påvirke tankene til andre

(Spørsmål til TIPS: Hører noen av bildene til teksten på de to siste sidene?)

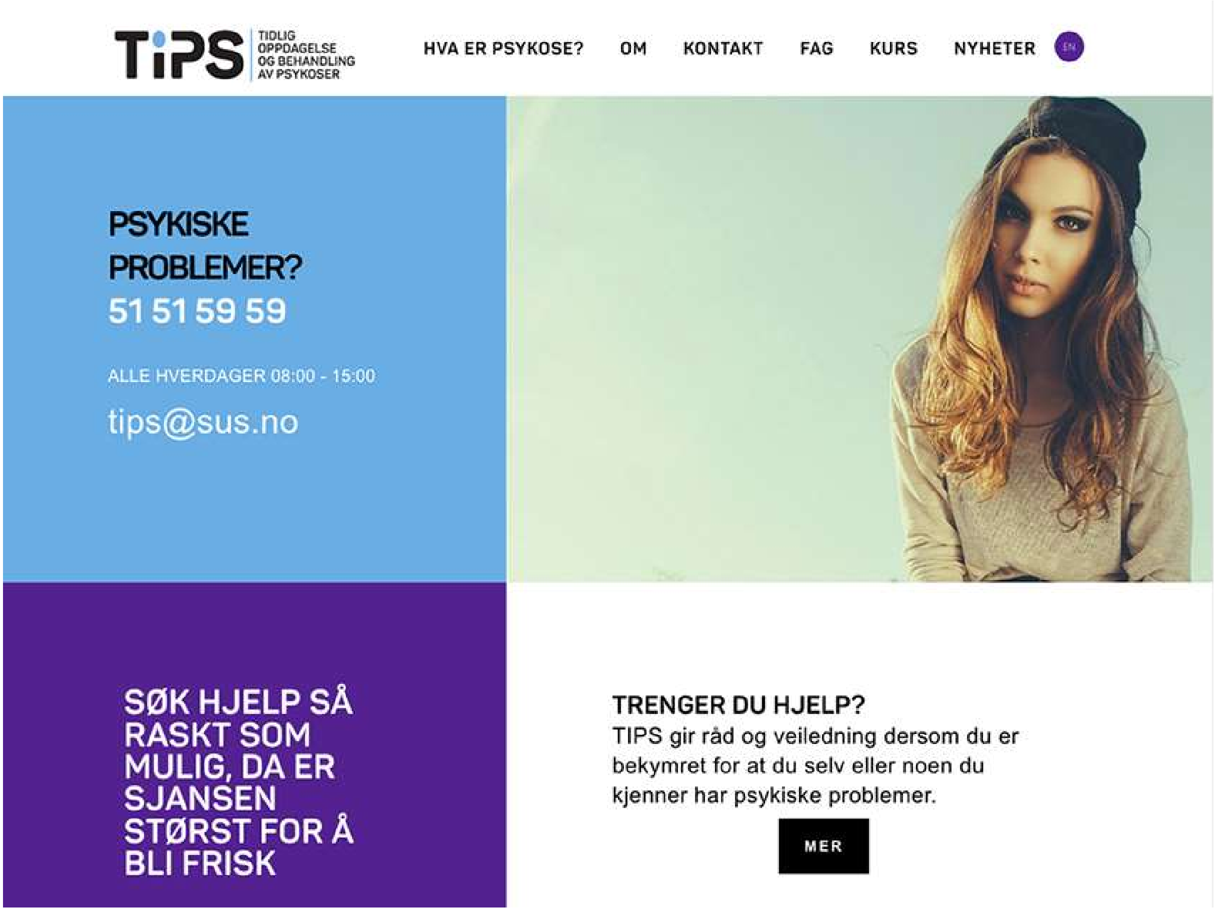

(hjemmeside TIPS info)

TIPS TIDLIG OPPDAGELSE OG BEHANDLING AV PSYKOSER

HVA ER PSYKOSE? OM KONTAKT FAG KURS NYHETER EN

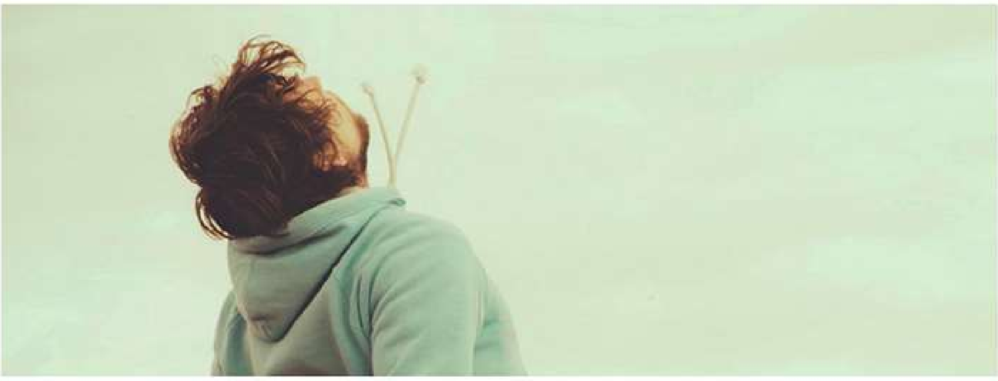

Hva er psykose?

HVA ER PSYKOSE?

Psykoser kan betraktes som alvorlige «forvirringstilstander», og betegner egentlig svært dyptgående nervøse sammenbrudd.

Hos unge mennesker kan slike sammenbrudd sette merker for livet, spesielt dersom det går lang tid fra sykdommen starter til pasienten får behandling. Husk alle har en psykisk helse!

PSYKISK HELSE

Vi betrakter psykosen som en prosess, hvor det psykotiske gjennombrudd eller01 sammenbrudd er et stadium i sykdomsutviklingen. Før psykosen inntrer har pasienten ofte hatt mer generelle og uspesifike tegn på psykisk lidelse, som f.eks. søvnforstyrrelser, angst, depresjon og isolasjon.

(hjemmeside TIPS info)

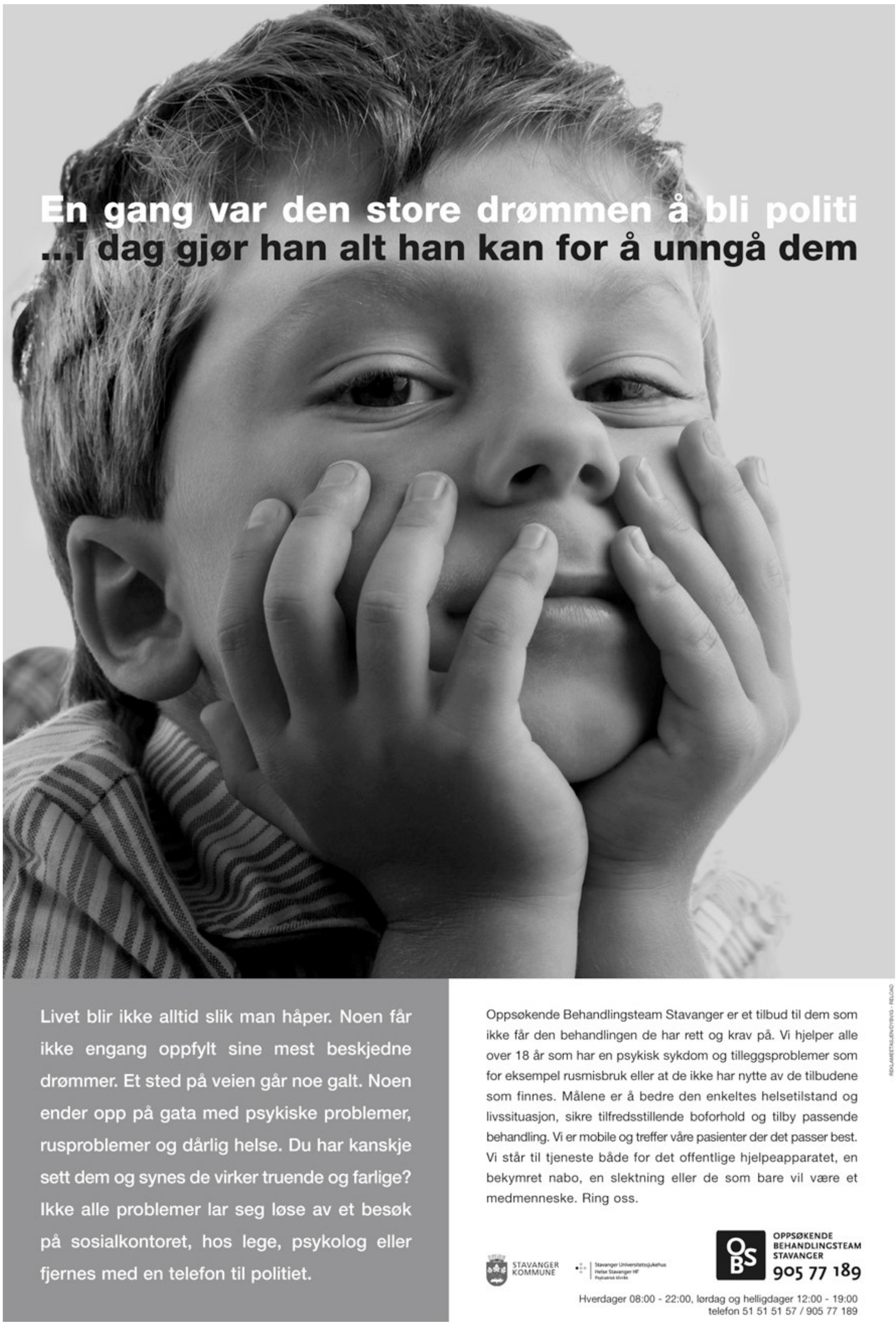
En gang var den store drømmen å bli politi
...i dag gjør han alt han kan for å unngå dem
Livet blir ikke alltid slik man håper. Noen får ikke engang oppfylt sine mest beskjedne drømmer. Et sted på veien går noe galt. Noen ender opp på gata med psykiske problemer, rusproblemer og dårlig helse. Du har kanskje sett dem og synes de virker truende og farlige? Ikke alle problemer lar seg løse av et besøk på sosialkontoret, hos lege, psykolog eller fjernes med en telefon til politiet.
Oppsøkende Behandlingsteam Stavanger er et tilbud til dem som ikke får den behandlingen de har rett og krav på. Vi hjelper alle over 18 år som har en psykisk sykdom og tilleggsproblemer som for eksempel rusmisbruk eller at de ikke har nytte av de tilbudene som finnes. Målene er å bedre den enkeltes helsetilstand og livssituasjon, sikre tilfredsstillende boforhold og tilby passende behandling. Vi er mobile og treffer våre pasienter der det passer best. Vi står til tjeneste både for det offentlige hjelpeapparatet, en bekymret nabo, en slektning eller de som bare vil være et medmenneske. Ring oss.
STAVANGER KOMMUNE
Stavanger Universitetssjukehus
OPPSØKENDE BEHANDLINGSTEAM STAVANGER
905 77 189
Hverdager 08:00 - 22:00, lørdag og helligdager 12:00 - 19:00
telefon 51 51 51 57 / 905 77 189

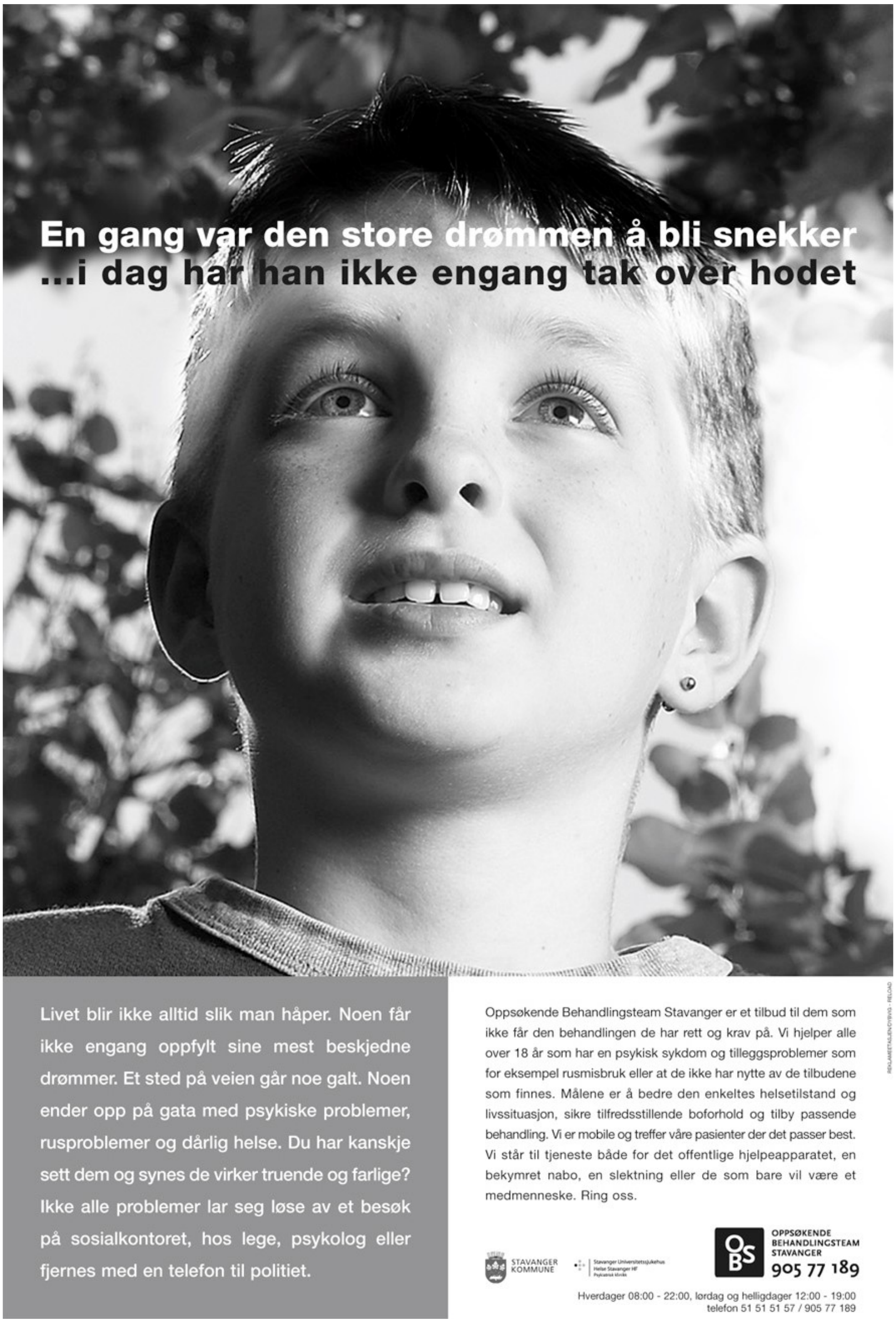

Spørsmål til TIPS: finnes det mye annet merket OBS?

(Ikke merket TIPS: inkludere/ekskludere?)

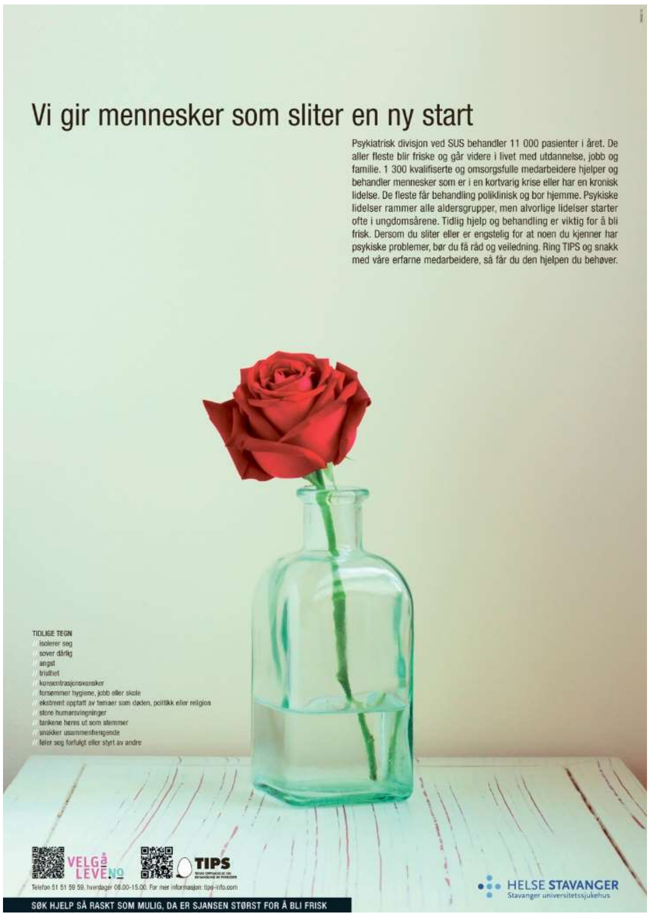

Vi gir mennesker som sliter en ny start
Psykiatrisk divisjon ved SUS behandler 11 000 pasienter i året. De aller fleste blir friske og går videre i livet med utdannelse, jobb og familie. 1 300 kvalifiserte og omsorgsfulle medarbeidere hjelper og behandler mennesker som er i en kortvarig krise eller har en kronisk lidelse. De fleste får behandling poliklinisk og bor hjemme. Psykiske lidelser rammer alle aldersgrupper, men alvorlige lidelser starter ofte i ungdomsårene. Tidlig hjelp og behandling er viktig for å bli frisk. Dersom du sliter eller er engstelig for at noen du kjenner har psykiske problemer, bør du få råd og veiledning. Ring TIPS og snakk med våre erfarne medarbeidere, så får du den hjelpen du behøver.
TIDLIGE TEGN
// isolerer seg
// sover dårlig
// angst
// tristhet
// konsentrasjonsvansker
// forsømmer hygiene, jobb eller skole
// ekstremt opptatt av temaer som døden, politikk eller religion
// store humørsvingninger
// tankene høres ut som stemmer
// snakker usammenhengende
// føler seg forfulgt eller styrt av andre
VELGå LEVE.NO
TIPS
Telefon 51 51 59 59, hverdager 08.00-15.00. For mer informasjon: tips-info.com
SØK HJELP SÅ RASKT SOM MULIG, DA ER SJANSEN STØRST FOR Å BLI FRISK
HELSE STAVANGER
Stavanger universitetssjukehus
