# Supplementary material for: “We all have a responsibility”: a narrative discourse analysis of an information campaign targeting help-seeking in first episode psychosis
Source: Int J Ment Health Syst. 2019 May 9;13:32. doi: 10.1186/s13033-019-0289-4 (PMC6507175; doi:10.1186/s13033-019-0289-4)
Supplement: Supplementary file 8 — Additional file 8. Data material part 2. Overview over data used in the analysis. [file 13033_2019_289_MOESM8_ESM.pdf]

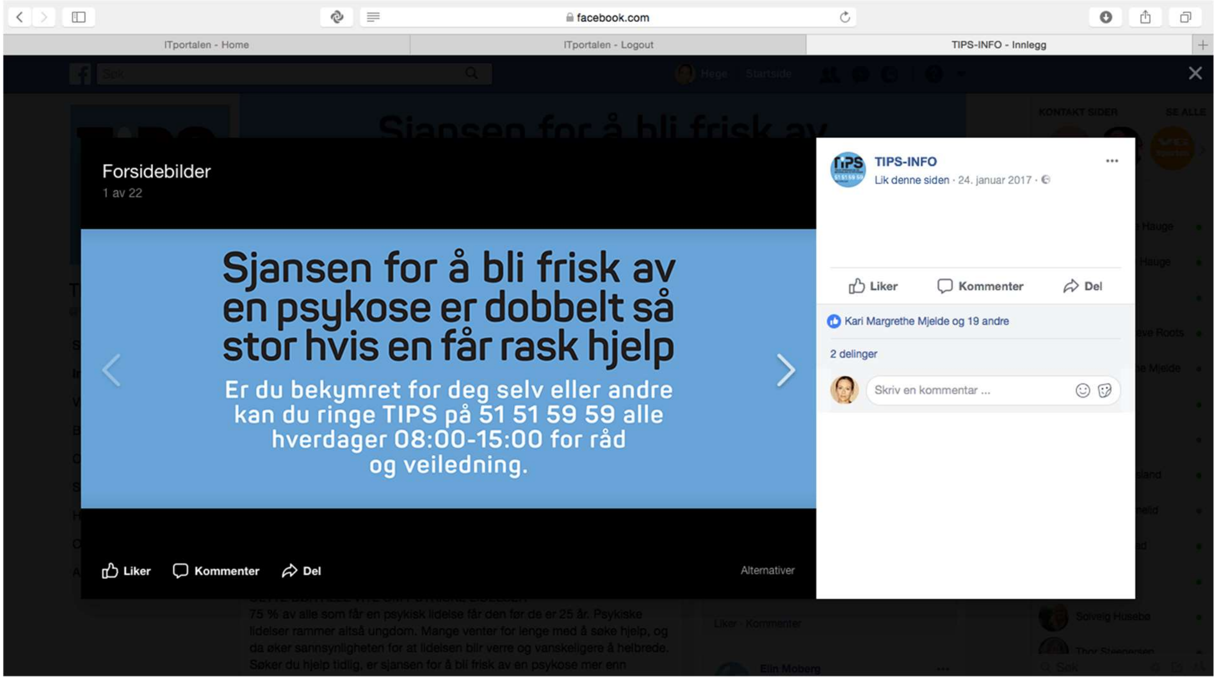
facebook.com
ITportalen - Home
ITportalen - Logout
TIPS-INFO - Innlegg
Forsidebilder
1 av 22
Sjansen for å bli frisk av en psykose er dobbelt så stor hvis en får rask hjelp
Er du bekymret for deg selv eller andre kan du ringe TIPS på 51 51 59 59 alle hverdager 08:00-15:00 for råd og veiledning.
Liker
Kommenter
Del
Alternativer
TIPS-INFO
Lik denne siden · 24. januar 2017
Liker
Kommenter
Del
Kari Margrethe Mjelde og 19 andre
2 delinger
Skriv en kommentar ...

TIPS-INFO
28. oktober 2017 ·

DETTE BØR ALLE VITE OM PSYKISKE LIDELSER
75 % av alle som får en psykisk lidelse får den før de er 25 år. Psykiske lidelser rammer altså ungdom. Mange venter for lenge med å søke hjelp, og da øker sannsynligheten for at lidelsen blir verre og vanskeligere å helbrede. Søker du hjelp tidlig, er sjansen for å bli frisk av en psykose mer enn dobbelt så stor! Er du bekymret for deg selv eller andre kan du ringe TIPS på 51 51 59 59 alle hverdager 08:00-15:00 for råd og veiledning.
SØK HJELP SÅ RASKT SOM MULIG,
DA ER SJANSEN STØRST FOR Å BLI FRISK

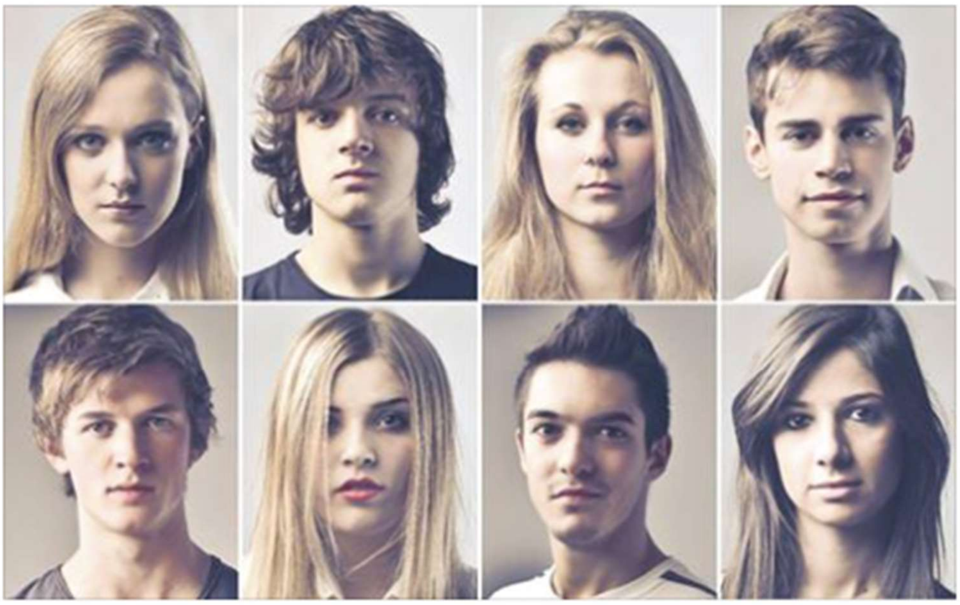

TIPS-INFO
Lokal bedrift

Send melding

Liker Kommenter Del

TIPS-INFO
2. januar ·

Vi har nettopp gått gjennom en tid med store forventninger, mye stress og vært tett innpå hverandre. Noen kan få det det vanskelig. Den som sliter, sliter kanskje litt ekstra? Noen trenger kanskje hjelp, men du vet ikke helt hva du skal gjøre? Er du bekymret for deg selv eller andre kan du ringe TIPS på 51 51 59 59 alle hverdager 08:00-15:00 for råd og veiledning.
SØK HJELP SÅ RASKT SOM MULIG,
DA ER SJANSEN STØRST FOR Å BLI FRISK

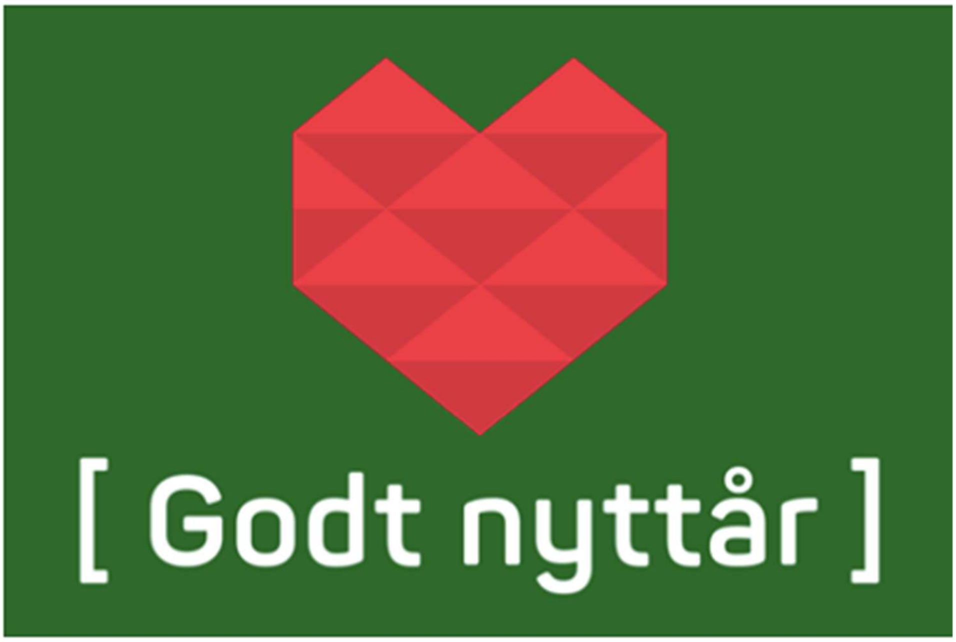

TIPS-INFO
Lokal bedrift

Send melding

Liker Kommenter Del

TIPS-INFO
26. desember 2017 ·

Vi ønsker alle en fortsatt god jul…men, for mange nådde den kanskje ikke helt opp til forventningene? Store forventninger. Mye stress. Tett innpå hverandre. Så får noen det vanskelig… Den som sliter, sliter kanskje litt ekstra? Noen trenger kanskje hjelp, men du vet ikke helt hva du skal gjøre? Er du bekymret for deg selv eller andre kan du ringe TIPS på 51 51 59 59 alle hverdager i romjulen 08:00-15:00 for råd og veiledning.
SØK HJELP SÅ RASKT SOM MULIG,
DA ER SJANSEN STØRST FOR Å BLI FRISK

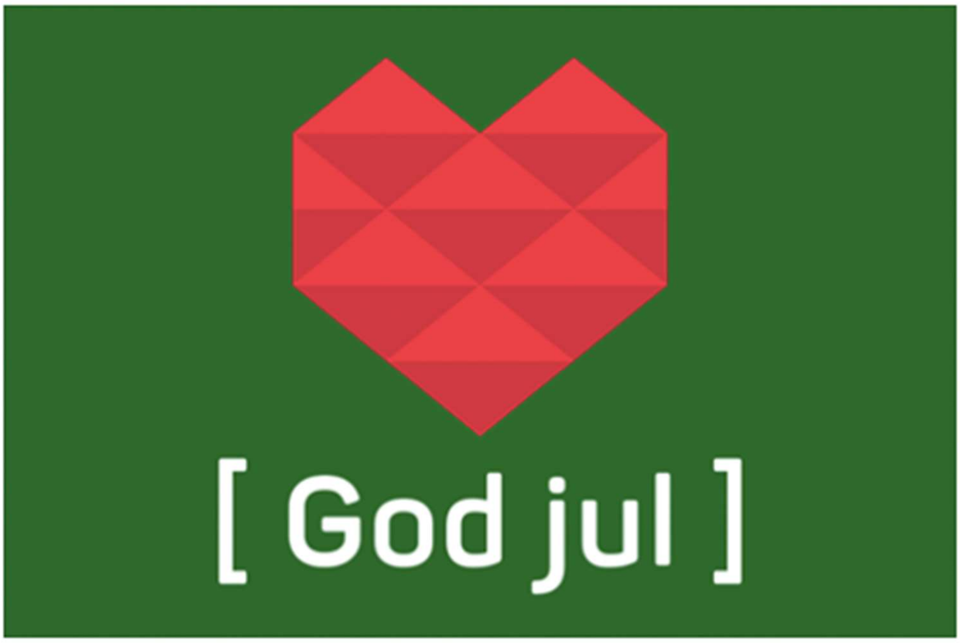

TIPS-INFO
Lokal bedrift

Send melding

TIPS-INFO

24. desember 2017 ·

Prognose er en forventning om hvordan noe vil utvikle seg. Innen helsefag er en på jakt etter det som øker sannsynligheten for å bli frisk. En vil en basere seg på tidligere erfaring, forskning og ulike tiltak som fremmer helse. Tidlig oppdagelse og behandling av psykose gir god prognose. Er du bekymret for deg selv eller andre kan du ringe TIPS på 51 51 59 59 alle hverdager 08:00-15:00 for råd og veiledning.
SØK HJELP SÅ RASKT SOM MULIG,
DA ER SJANSEN STØRST FOR Å BLI FRISK

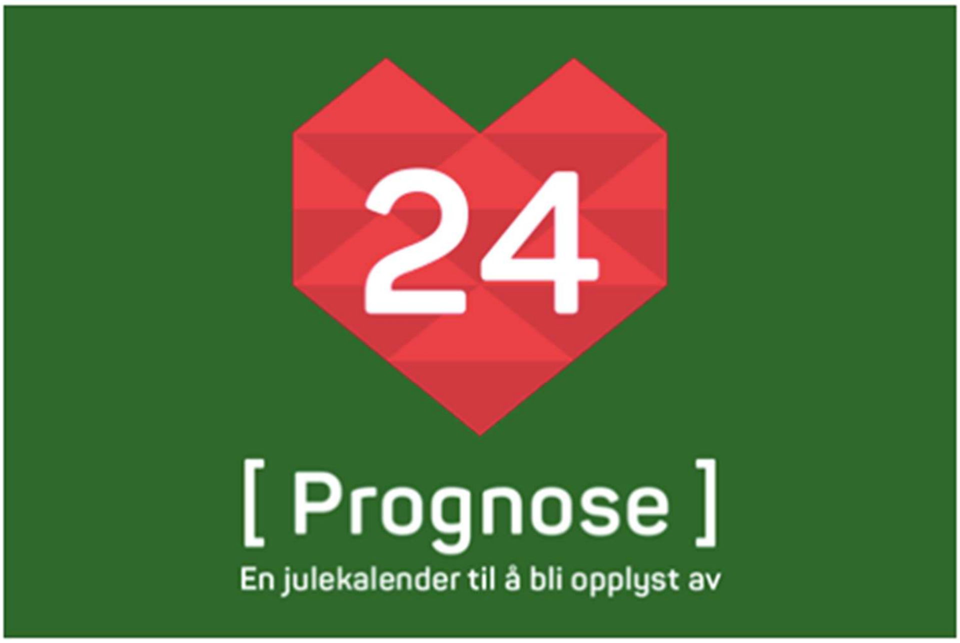

TIPS-INFO

Lokal bedrift

Send melding

Liker Kommenter Del

TIPS-INFO
23. desember 2017 ·

Tidlig intervensjon betyr å gripe inn tidlig når en er bekymret for et annet menneske. I psykisk helsearbeid betyr det å oppdage og behandle lidelser så raskt som mulig. Sjansen for å bli helt frisk fra f.eks. en psykose, øker jo tidligere en får behandling. Psykiske lidelser oppstår først og fremst i ungdomsårene. Er du bekymret for deg selv eller andre kan du ringe TIPS på 51 51 59 59 alle hverdager 08:00-15:00 for råd og veiledning.
SØK HJELP SÅ RASKT SOM MULIG,
DA ER SJANSEN STØRST FOR Å BLI FRISK

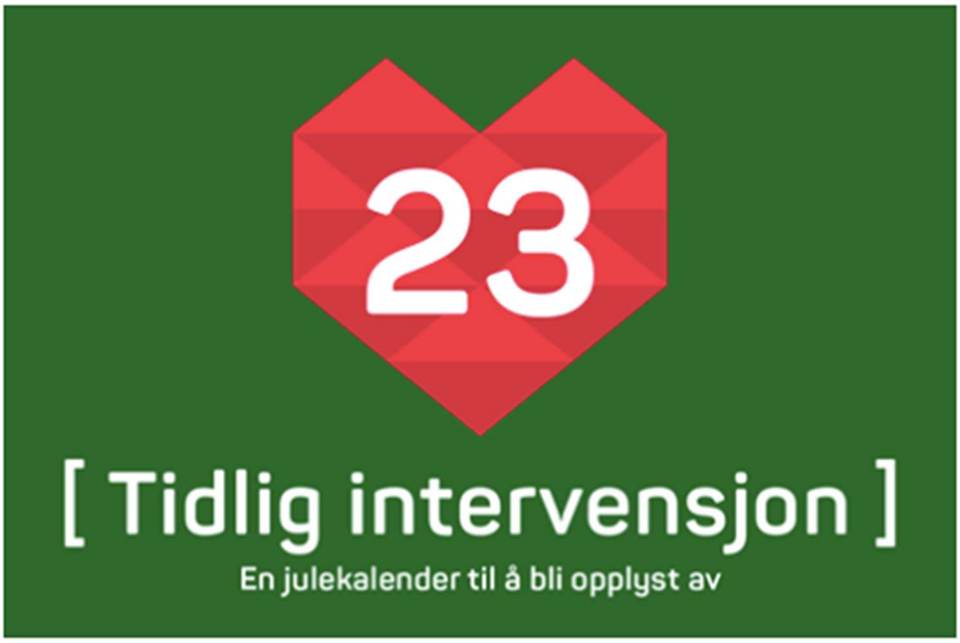

TIPS-INFO
Lokal bedrift

Send melding

[illegible]

TIPS-INFO

22. desember 2017 ·

Psykoser kan betraktes som alvorlige «forvirringstilstander», og betegner egentlig svært dyptgående nervøse sammenbrudd. De tydeligste symptomer er hallusinasjoner, vrangforestillinger og forfølgelsesideer. Angst, isolasjon, forvirring er ofte tidlige tegn, men vil ikke nødvendigvis utvikle seg til psykose. Det er likevel viktig å søke tidlig hjelp, da sjansen for å stanse utviklingen er størst når psykosen oppdages og behandles tidlig. Er du bekymret for deg selv eller andre kan du ringe TIPS på 51 51 59 59 alle hverdager 08:00-15:00 for råd og veiledning.
SØK HJELP SÅ RASKT SOM MULIG,
DA ER SJANSEN STØRST FOR Å BLI FRISK

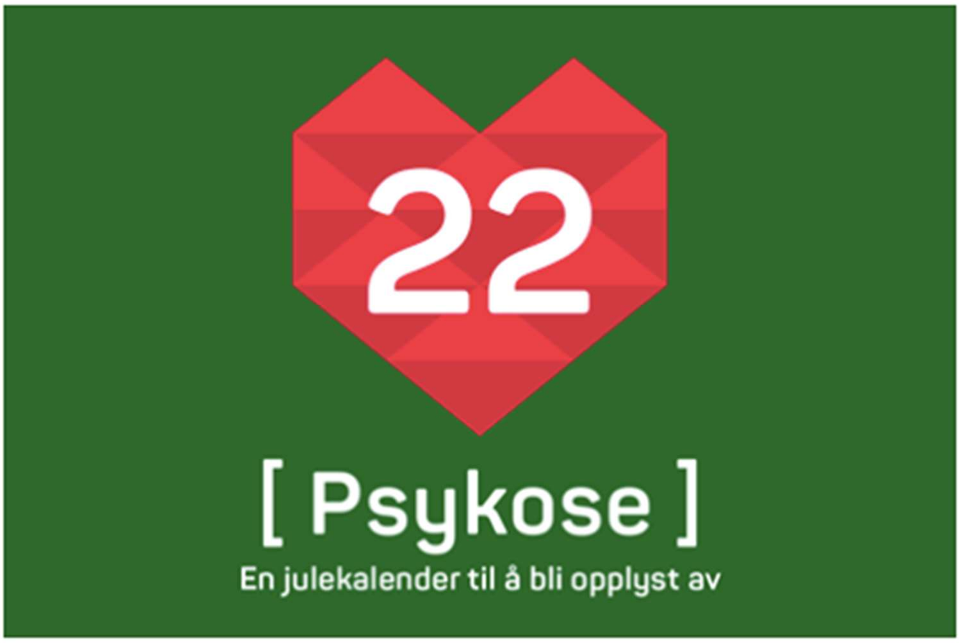

TIPS-INFO

Send melding

TIPS-INFO
21. desember 2017 ·

Aggresjon forklares gjerne som oppførsel der hensikten å ydmyke, skade eller påføre smerte. Frustrasjon, irritasjon og raseri er ulike varianter av aggresjon. Aggresjon uten en åpenbar årsak kan være et signal om at noe er i veien. Tilsynelatende umotivert aggresjon kan være blant tidlige symptomer på psykose. Er du bekymret for deg selv eller andre kan du ringe TIPS på 51 51 59 59 alle hverdager 08:00-15:00 for råd og veiledning.
SØK HJELP SÅ RASKT SOM MULIG,
DA ER SJANSEN STØRST FOR Å BLI FRISK

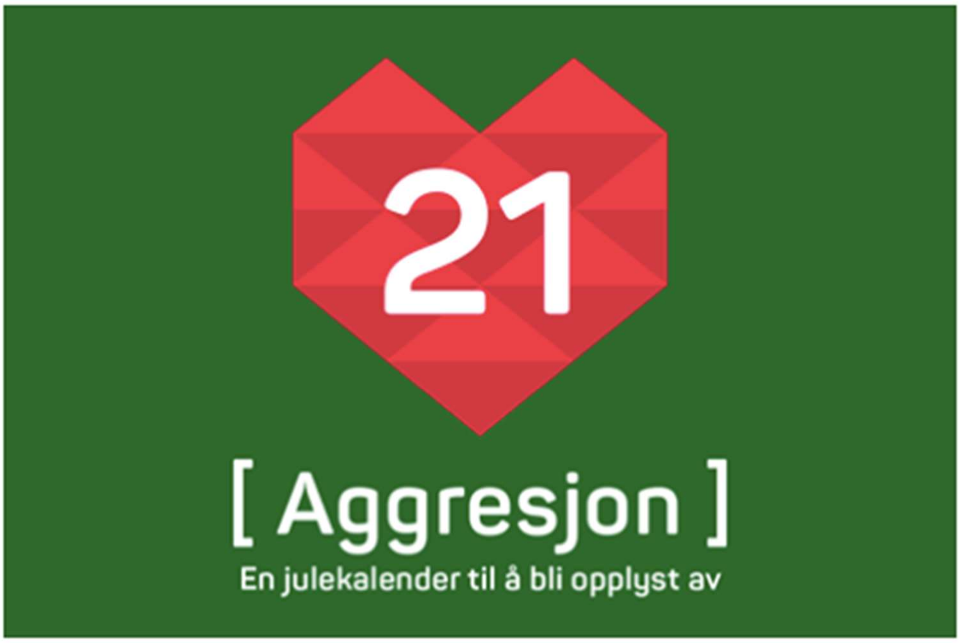

TIPS-INFO
Lokal bedrift

Send melding

Liker Kommenter Del

TIPS-INFO
20. desember 2017 ·

Positive symptomer kan forklares med at noe kommer i tillegg til vanlige følelser og sanseopplevelser. Det kan være sterke, umotiverte følelsesutbrudd, panikk, hallusinasjoner og storhetstanker. Positive symptomer er typiske for psykoselidelser. Er du bekymret for deg selv eller andre kan du ringe TIPS på 51 51 59 59 alle hverdager 08:00-15:00 for råd og veiledning.
SØK HJELP SÅ RASKT SOM MULIG,
DA ER SJANSEN STØRST FOR Å BLI FRISK

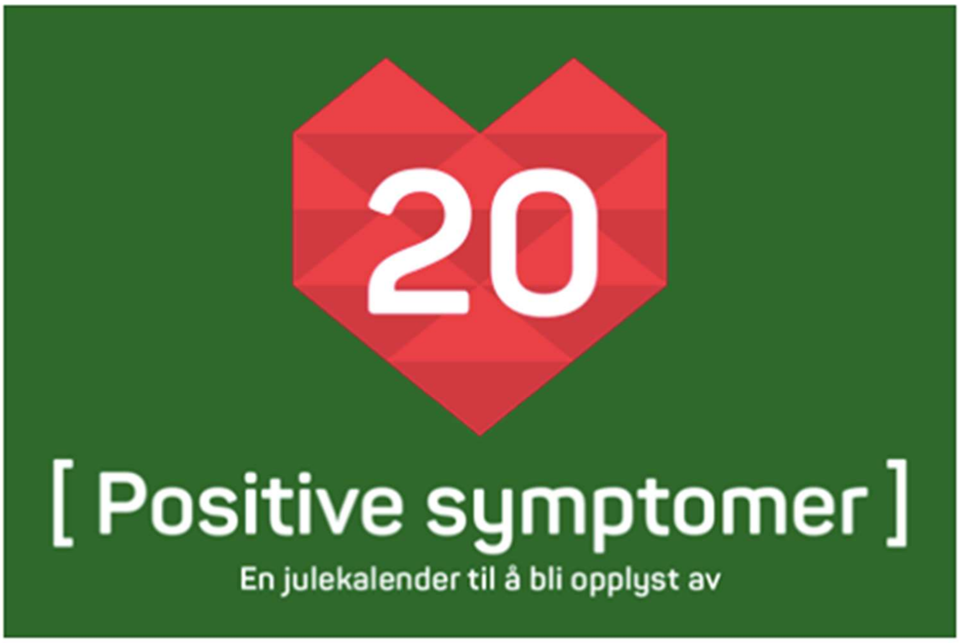

TIPS-INFO
Lokal bedrift

Send melding

Liker Kommenter Del

TIPS-INFO
19. desember 2017 ·

Negative symptomer kan vi forklare med at noe forsvinner. En mister evnen til å oppleve og uttrykke følelser, f.eks. ingen glede eller sinne. En kan bli likegyldig, tiltaksløs og miste engasjement. Kan være de første tegnene på en alvorlig psykisk lidelse. Psykoser starter gjerne med negative symptomer. Er du bekymret for deg selv eller andre kan du ringe TIPS på 51 51 59 59 alle hverdager 08:00-15:00 for råd og veiledning.
SØK HJELP SÅ RASKT SOM MULIG,
DA ER SJANSEN STØRST FOR Å BLI FRISK

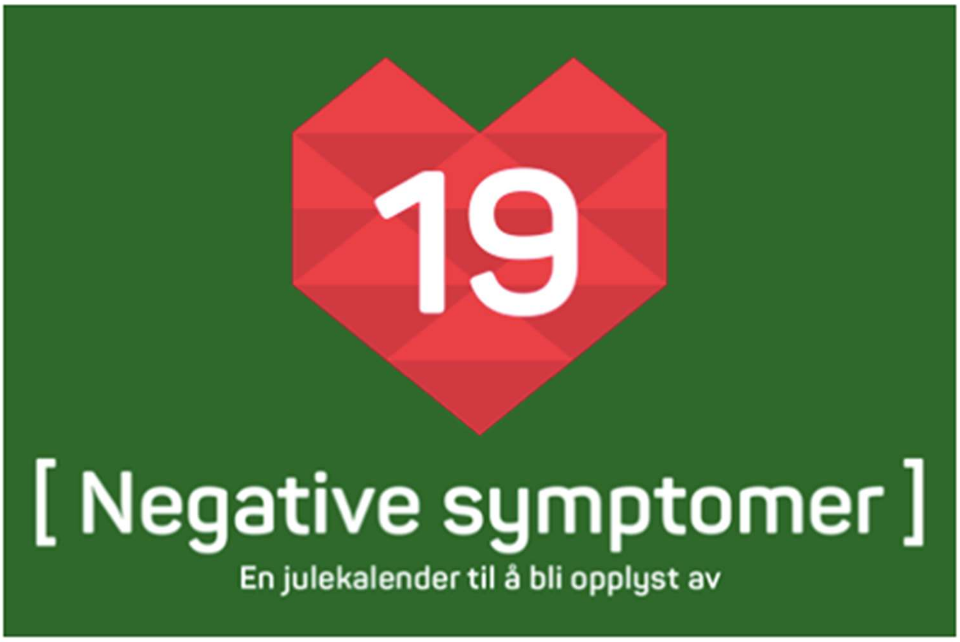

TIPS-INFO
Lokal bedrift

Send melding

TIPS-INFO

18. desember 2017 ·

Avmakt er følelsen av å mangle evne eller krefter til å endre på en situasjon. Det er ikke en diagnose, men vi tar det med i kalenderen for å minne om at det er viktig å søke hjelp tidlig, og at de aller fleste som får en psykisk lidelse blir friske. Avmakt kan være en del av en psykisk lidelse og hindre noen i å oppsøke hjelp. Er du bekymret for venner, kjente eller familie kan du ringe TIPS på 51 51 59 59 alle hverdager 08:00-15:00 for råd og veiledning.
SØK HJELP SÅ RASKT SOM MULIG,
DA ER SJANSEN STØRST FOR Å BLI FRISK

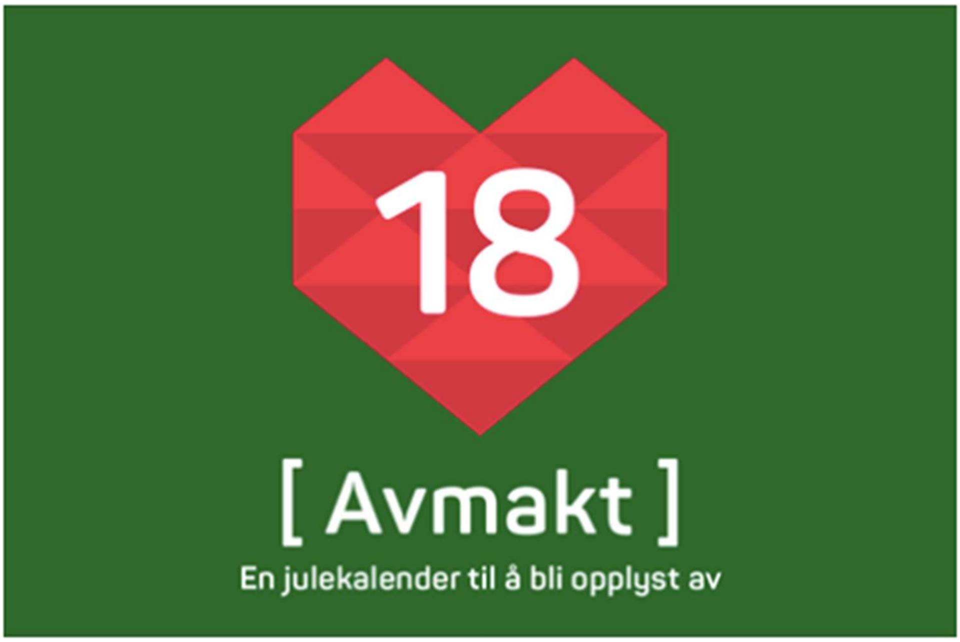

TIPS-INFO

Lokal bedrift

Send melding

TIPS-INFO
17. desember 2017 ·

Tankepåvirkning er opplevelsen at tankene blir styrt utenfra, at en blir gitt eller tatt fra tanker. En kan føle at andre kan lese tankene. Noen kaller det tankekjør, da ofte i betydningen at tankene spinner raskt og ukontrollert. Tankepåvirkning forekommer ofte ved psykoser. Er du bekymret for deg selv eller andre kan du ringe TIPS på 51 51 59 59 alle hverdager 08:00-15:00 for råd og veiledning.
SØK HJELP SÅ RASKT SOM MULIG,
DA ER SJANSEN STØRST FOR Å BLI FRISK

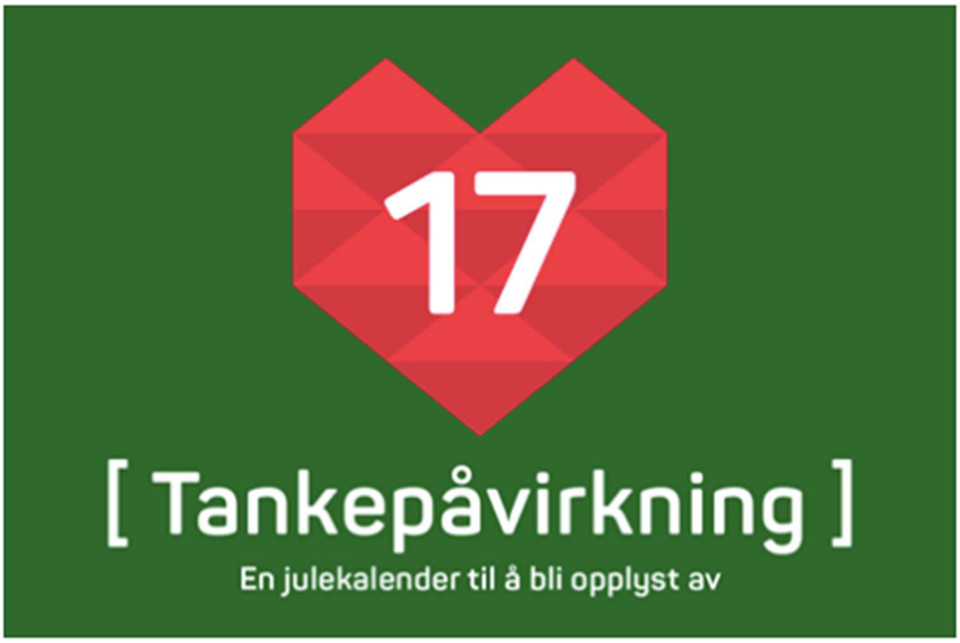

TIPS-INFO
Lokal bedrift

Send melding

Liker Kommenter Del

TIPS-INFO
16. desember 2017 ·

Traume er en skade. Psykisk traume er naturlige reaksjoner på unaturlige hendelser. Vi kjenner det fra bl.a. krig, tortur og overgrep. Gjentatte eller langvarige traumer kan føre til psykiske forstyrrelser som preger og ødelegger livskvaliteten. Traumer kan også forårsake psykoser. Er du bekymret for deg selv eller andre kan du ringe TIPS på 51 51 59 59 alle hverdager 08:00-15:00 for råd og veiledning.
SØK HJELP SÅ RASKT SOM MULIG,
DA ER SJANSEN STØRST FOR Å BLI FRISK

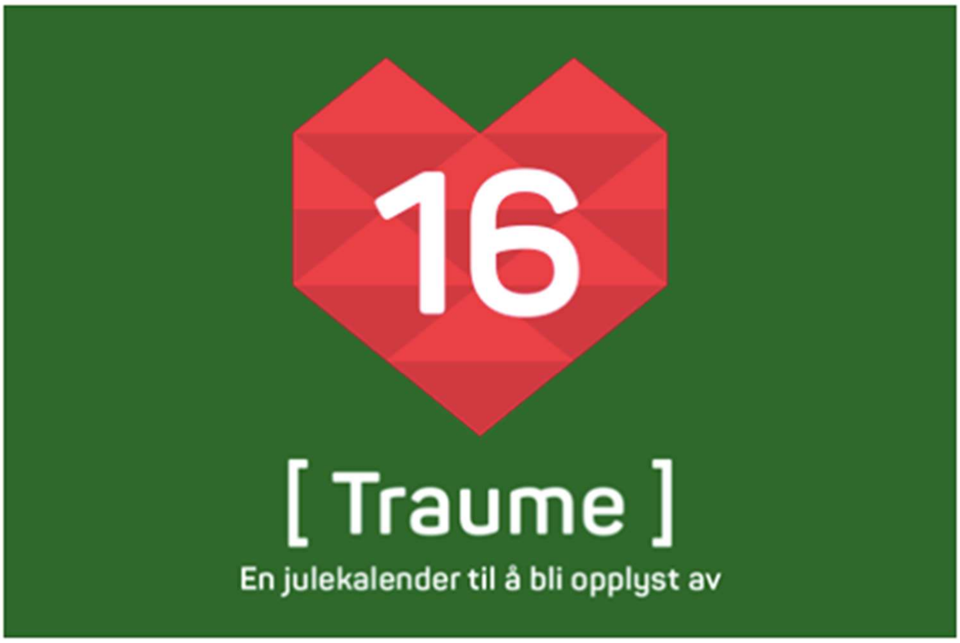

TIPS-INFO
Lokal bedrift

Send melding

TIPS-INFO
15. desember 2017 ·

Storhetstanker er en forstilling om å ha spesielle evner eller oppgaver å utføre. En kan tro at en har magiske evner, er en viktig religiøs leder, skal utføre fantastiske bragder, som f.eks. å bygge gullslott. Storhetstanker opptrer ved flere ulike psykiske lidelser, men er særlig tydelig ved psykoser. Er du bekymret for deg selv eller andre kan du ringe TIPS på 51 51 59 59 alle hverdager 08:00-15:00 for råd og veiledning.
SØK HJELP SÅ RASKT SOM MULIG,
DA ER SJANSEN STØRST FOR Å BLI FRISK

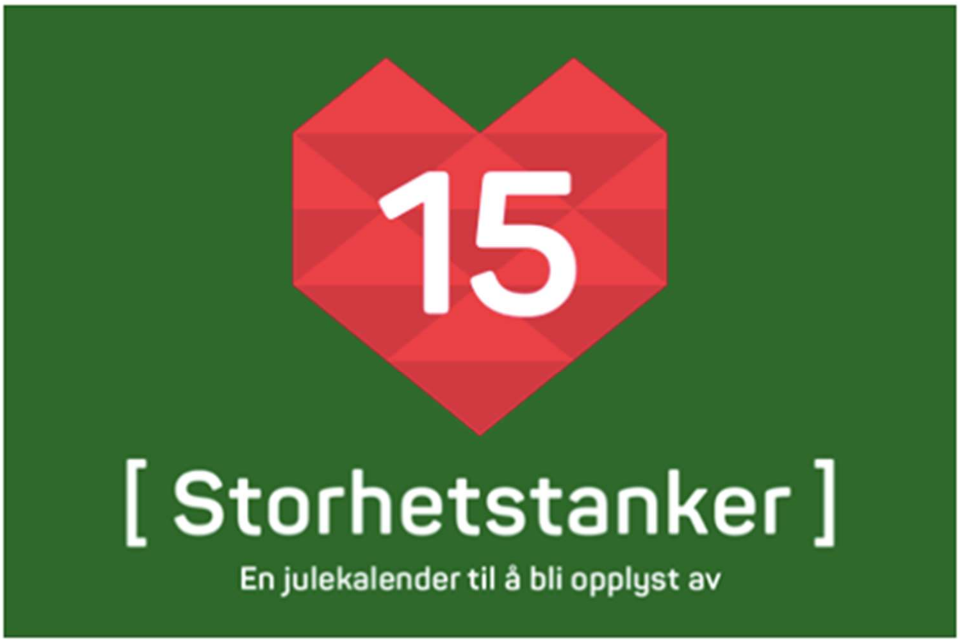

TIPS-INFO
Lokal bedrift

Send melding

TIPS-INFO
14. desember 2017 ·

Paranoia er en tilstand som preges av at tanker og følelser er bundet opp til en forestilling om at en er utsatt for forfølgelse eller en sammensvergelse. Noen lever greit med lidelsen så lenge den ikke blir utfordret, andre blir sterkt preget av sine vrangforestillinger. Paranoia kan også være et tegn på en psykoselidelse, særlig hos yngre mennesker. Er du bekymret for deg selv eller andre kan du ringe TIPS på 51 51 59 59 alle hverdager 08:00-15:00 for råd og veiledning.
SØK HJELP SÅ RASKT SOM MULIG,
DA ER SJANSEN STØRST FOR Å BLI FRISK

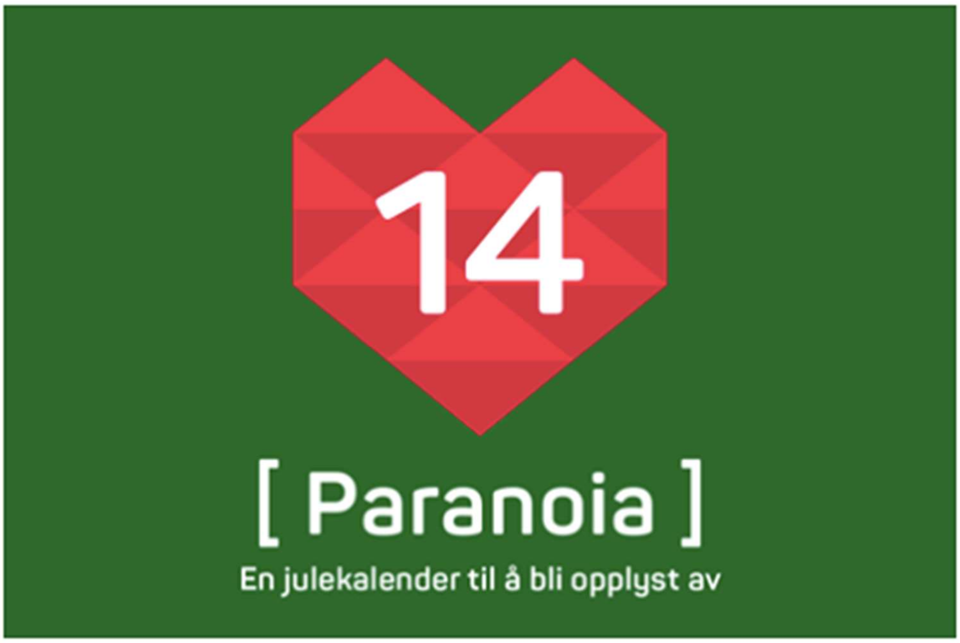

TIPS-INFO
Lokal bedrift

Send melding

TIPS-INFO

13. desember 2017 ·

Stress er ikke bare en forbigående travelhet. Stress som et negativt fenomen oppstår når en opplever at krav og forventninger overstiger egen kapasitet og mestring og kan være en del av flere psykiske og fysiske lidelser. Stress kan også utløse psykoser. Er du bekymret for deg selv eller andre kan du ringe TIPS på 51 51 59 59 alle hverdager 08:00-15:00 for råd og veiledning.
SØK HJELP SÅ RASKT SOM MULIG,
DA ER SJANSEN STØRST FOR Å BLI FRISK

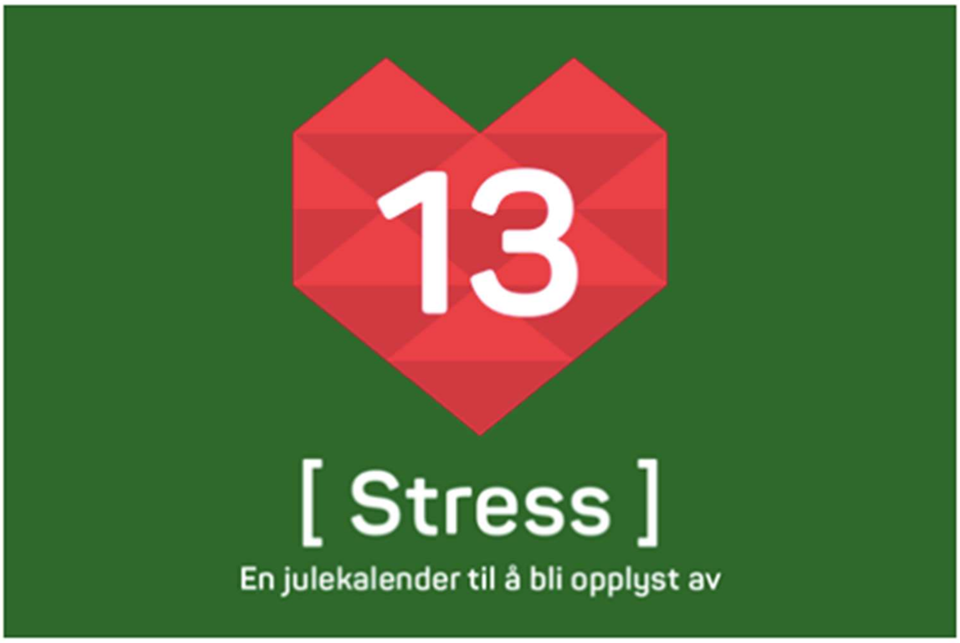

TIPS-INFO

Lokal bedrift

Send melding

[illegible]

TIPS-INFO

12. desember 2017 ·

Isolasjon er ikke det samme som ensomhet. Når en trekker seg tilbake, unngår kontakt med venner, kjente og familie, uteblir fra jobb og skole fordi en har ubehag, føler seg nedfor og deprimert kan det være et tegn på andre problemer. At en isolerer seg kan være et av de tidlige tegnene på psykoselidelser. Er du bekymret for deg selv eller andre kan du ringe TIPS på 51 51 59 59 alle hverdager 08:00-15:00 for råd og veiledning.
SØK HJELP SÅ RASKT SOM MULIG,
DA ER SJANSEN STØRST FOR Å BLI FRISK

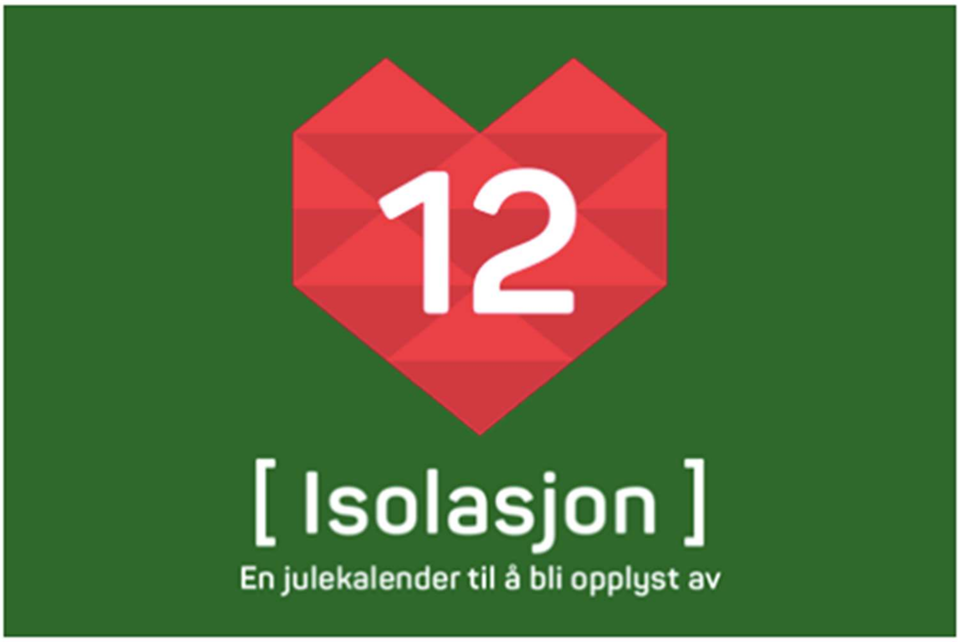

TIPS-INFO

Lokal bedrift

Send melding

Liker Kommenter Del

TIPS-INFO
11. desember 2017 ·

Tvangstanker beskriver påtrengende og uønskete tanker som stadig gjentar seg og som er vanskelige å kontrollere. Tvangstanker kan føre til handlinger som en føler seg tvunget til å utføre. Psykoselidelser kan ha tvangstanker som en del av symptomene. Er du bekymret for deg selv eller andre kan du ringe TIPS på 51 51 59 59 alle hverdager 08:00-15:00 for råd og veiledning.
SØK HJELP SÅ RASKT SOM MULIG,
DA ER SJANSEN STØRST FOR Å BLI FRISK

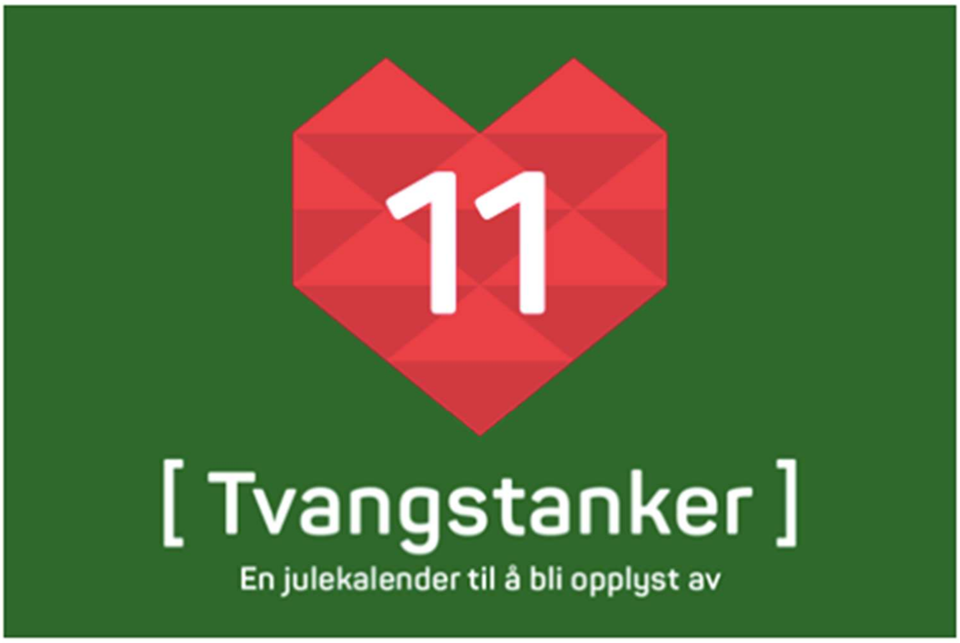

TIPS-INFO
Lokal bedrift

Send melding

Liker Kommenter Del

TIPS-INFO

10. desember 2017 ·

Stemmehøring forklares gjerne med at man «hører» tanker som virkelige stemmer som kommer utenfra. Slike «stemmer» kan være gode, slemme eller nøytrale. Noen lever greit med slike «stemmer», men for de fleste er de svært plagsomme.Stemmehøring er et typisk tegn på en psykoselidelse. Er du bekymret for deg selv eller andre kan du ringe TIPS på 51 51 59 59 alle hverdager 08:00-15:00 for råd og veiledning.

SØK HJELP SÅ RASKT SOM MULIG,

DA ER SJANSEN STØRST FOR Å BLI FRISK

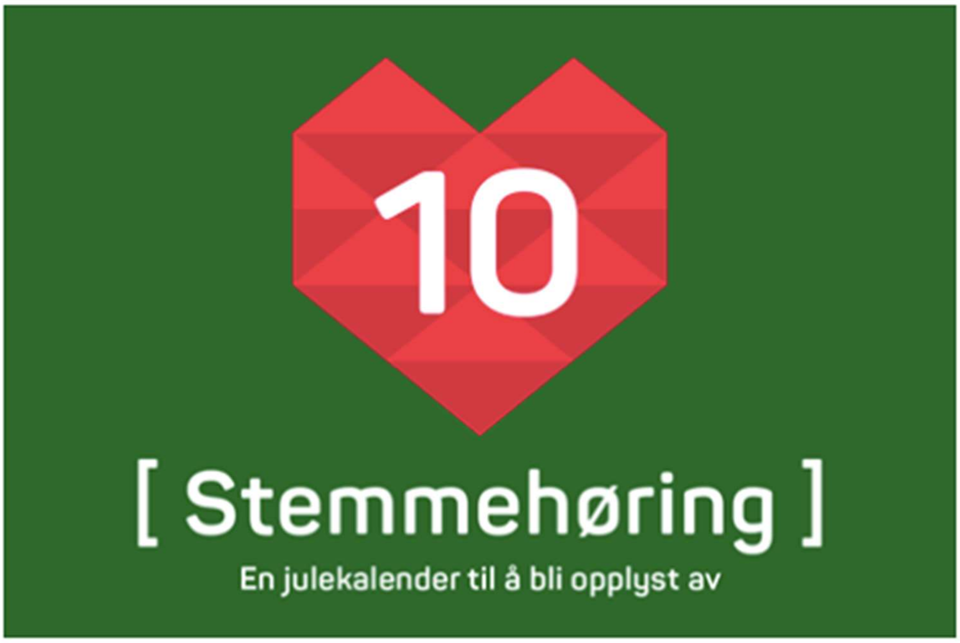

TIPS-INFO

Lokal bedrift

Send melding

TIPS-INFO
9. desember 2017 ·

Hallusinasjoner er sanseopplevelser som ikke er skapt av ytre sanseinntrykk. Eksempler er at en ser, hører, smaker eller lukter ting som ingen andre opplever. Hallusinasjoner kan utløses av rusmidler, men er ofte tegn på en psykose. Er du bekymret for deg selv eller andre kan du ringe TIPS på 51 51 59 59 alle hverdager 08:00-15:00 for råd og veiledning.
SØK HJELP SÅ RASKT SOM MULIG,
DA ER SJANSEN STØRST FOR Å BLI FRISK

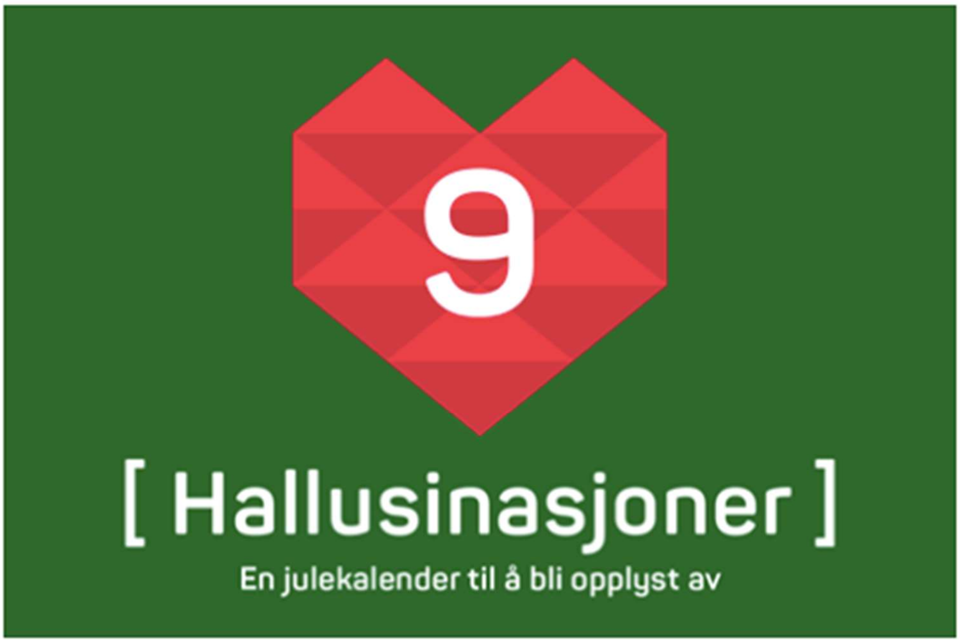

TIPS-INFO
Lokal bedrift

Send melding

Liker Kommenter Del

TIPS-INFO

8. desember 2017 ·

Depresjon er en tilstand med interesse- og gledesløshet, tretthet og tunge tanker. Ofte med lav selvfølelse, triste tanker og pessimisme. Tanker om selvmord er ikke uvanlig. Kan oppleves som en fysisk smerte. Depresjon kan også være et tegn på en psykoselidelse. Er du bekymret for deg selv eller andre kan du ringe TIPS på 51 51 59 59 alle hverdager 08:00-15:00 for råd og veiledning.
SØK HJELP SÅ RASKT SOM MULIG,
DA ER SJANSEN STØRST FOR Å BLI FRISK

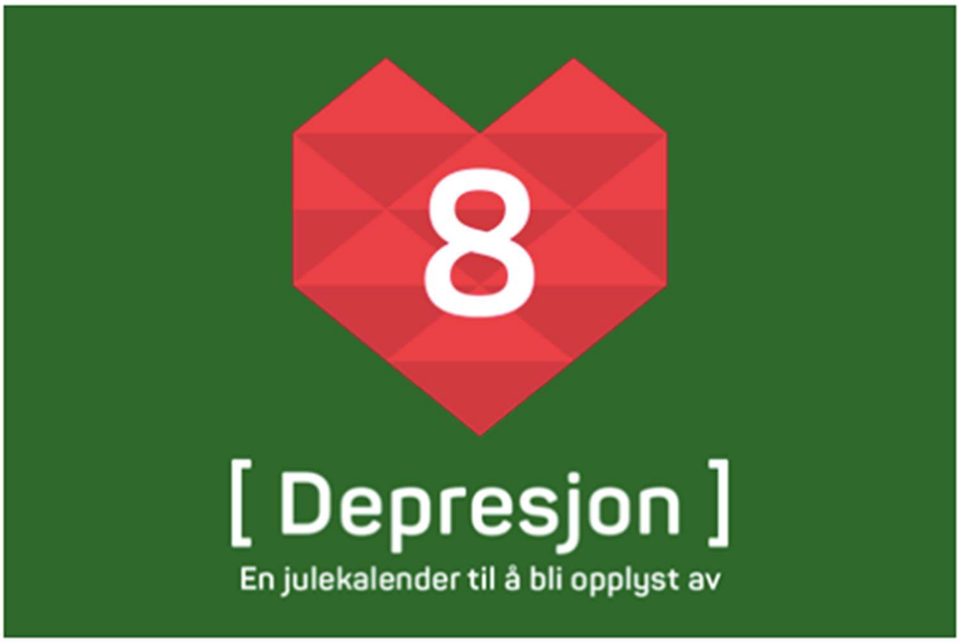

TIPS-INFO

Lokal bedrift

Send melding

Liker Kommenter Del

TIPS-INFO

7. desember 2017 ·

Mani er en tilstand der stemnings- og energinivå er langt over normalen. Kommer gjerne i perioder på uker og måneder, med uvettig bruk av penger, urealistiske planer, storslagne prosjekter og manglende selvkritikk. Mani kan avløses av depresjon eller gå over i psykose. Er du bekymret for deg selv eller andre kan du ringe TIPS på 51 51 59 59 alle hverdager 08:00-15:00 for råd og veiledning.
SØK HJELP SÅ RASKT SOM MULIG,
DA ER SJANSEN STØRST FOR Å BLI FRISK

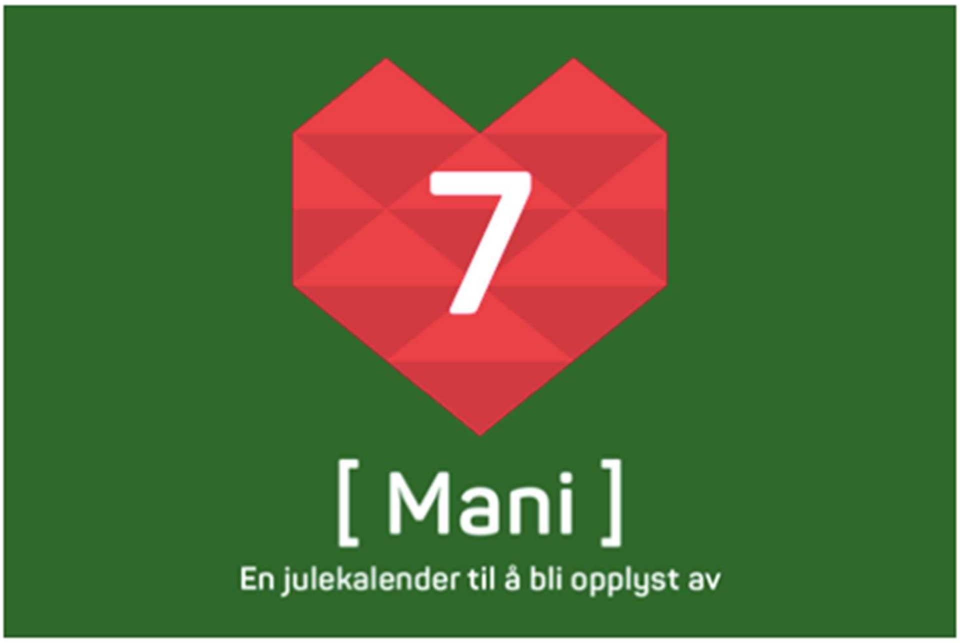

TIPS-INFO

Lokal bedrift

Send melding

Liker Kommenter Del

TIPS-INFO

6. desember 2017 ·

Sterke følelser kan være en naturlig ting: gledelig overrasket, rettferdig harme, kjærlighetserklæring, grensesetting. Sterke følelsesutbrudd uten en naturlig årsak kan være tegn på en begynnende psykisk lidelse. Er du bekymret for deg selv eller andre kan du ringe TIPS på 51 51 59 59 alle hverdager 08:00-15:00 for råd og veiledning.
SØK HJELP SÅ RASKT SOM MULIG,
DA ER SJANSEN STØRST FOR Å BLI FRISK

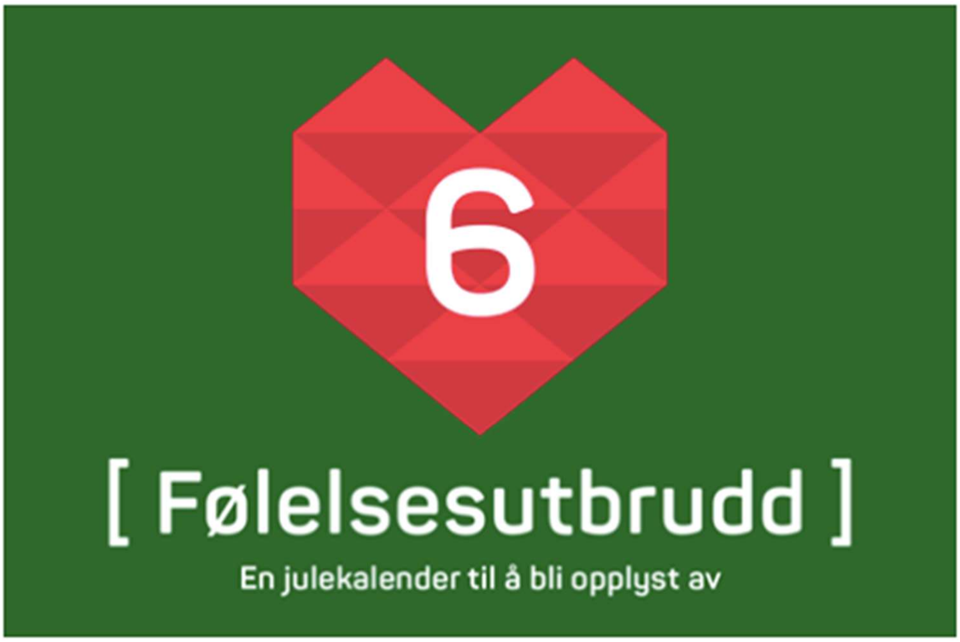

TIPS-INFO

Lokal bedrift

Send melding

Liker Kommenter Del

TIPS-INFO ...

5. desember 2017 ·

Humørsvingninger er raske skiftninger i humøret. Lei seg, glad, sint, oppgitt eller opplagt uten helt å vite hvorfor. Det kan ha fysiske eller psykiske årsaker, som for eksempel hormonelle endringer, kjemiske ubalanser, eller alvorlig stress. Det kan også være tegn på en begynnende psykisk lidelse. Er du bekymret for deg selv eller andre kan du ringe TIPS på 51 51 59 59 alle hverdager 08:00-15:00 for råd og veiledning.
SØK HJELP SÅ RASKT SOM MULIG,
DA ER SJANSEN STØRST FOR Å BLI FRISK

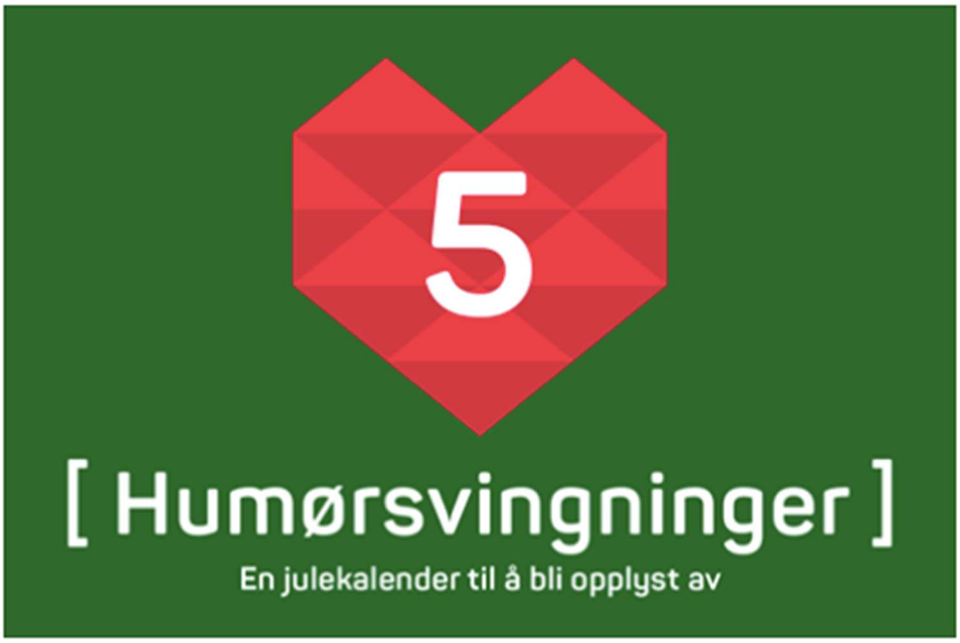

TIPS-INFO

Lokal bedrift

Send melding

Liker Kommenter Del

TIPS-INFO
4. desember 2017 ·

Tristhet er en nedstemt sinnsstemning, bedrøvet og sørgmodig. Det kan være en naturlig reaksjon på ubehagelige opplevelser eller vanskelige perioder i livet. Tristhet kan også være tegn på en begynnende psykisk lidelse. Er du bekymret for deg selv eller andre kan du ringe TIPS på 51 51 59 59 alle hverdager 08:00-15:00 for råd og veiledning.
SØK HJELP SÅ RASKT SOM MULIG,
DA ER SJANSEN STØRST FOR Å BLI FRISK

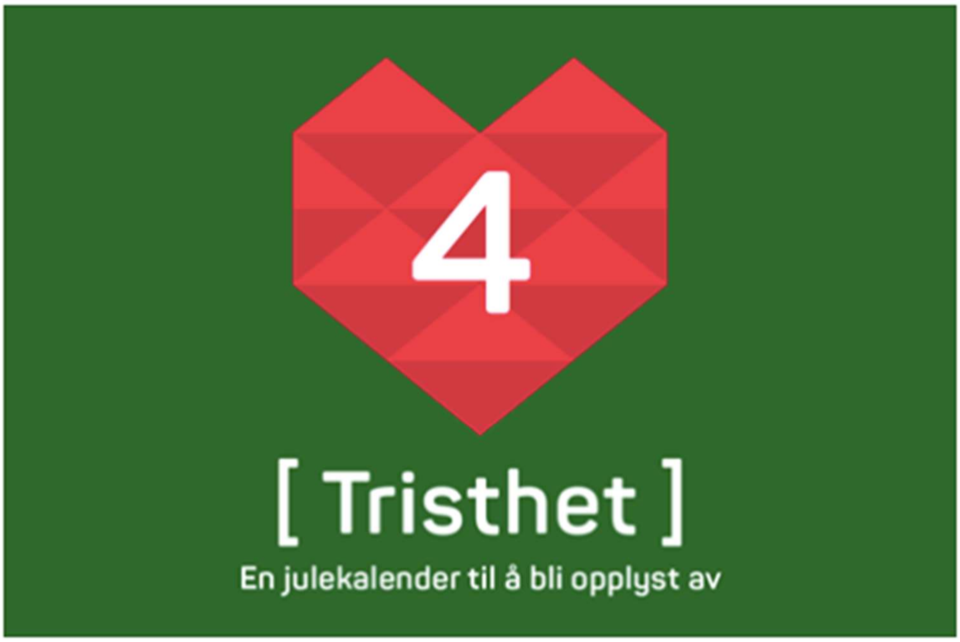

TIPS-INFO
Lokal bedrift

Send melding

Liker Kommenter Del

TIPS-INFO
3. desember 2017 ·

Angst er et sterkt ubehag uten en åpenbar årsak. Symptomer kan være uro, redsel, hjertebank, pustevansker, konsentrasjonsvansker og tristhet. Angst kan også være tegn på en begynnende psykisk lidelse. Er du bekymret for deg selv eller andre kan du ringe TIPS på 51 51 59 59 alle hverdager 08:00-15:00 for råd og veiledning.
SØK HJELP SÅ RASKT SOM MULIG,
DA ER SJANSEN STØRST FOR Å BLI FRISK

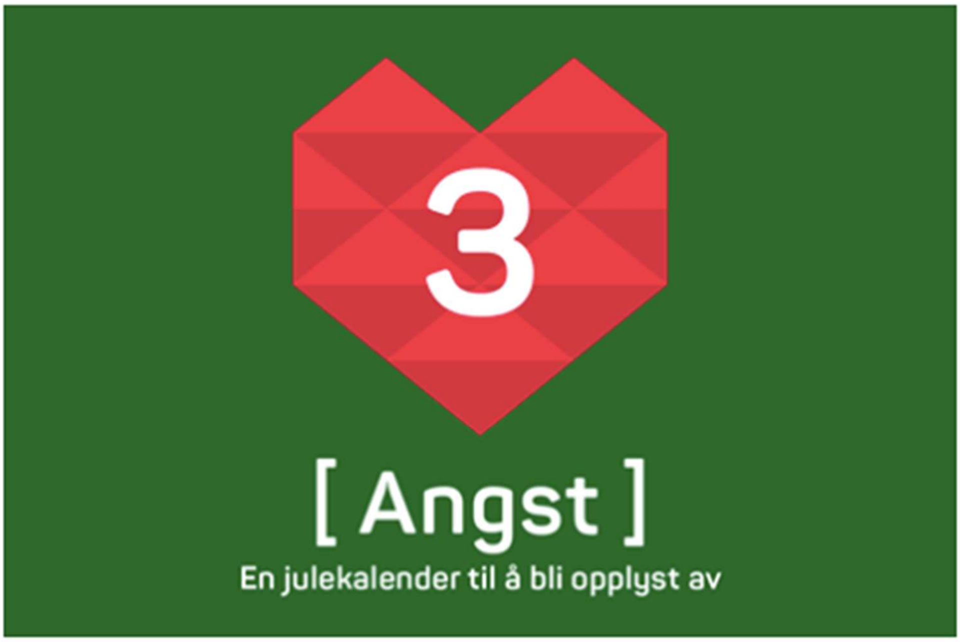

TIPS-INFO
Lokal bedrift

Send melding

Liker Kommenter Del

TIPS-INFO
2. desember 2017 ·

Dårlig søvn kalles insomni på fagspråket. 10 % av befolkningen har alvorlige og langvarige søvnproblemer. Insomni kan også være tegn på en begynnende psykisk lidelse. Er du bekymret for deg selv eller andre kan du ringe TIPS på 51 51 59 59 alle hverdager 08:00-15:00 for råd og veiledning.
SØK HJELP SÅ RASKT SOM MULIG,
DA ER SJANSEN STØRST FOR Å BLI FRISK

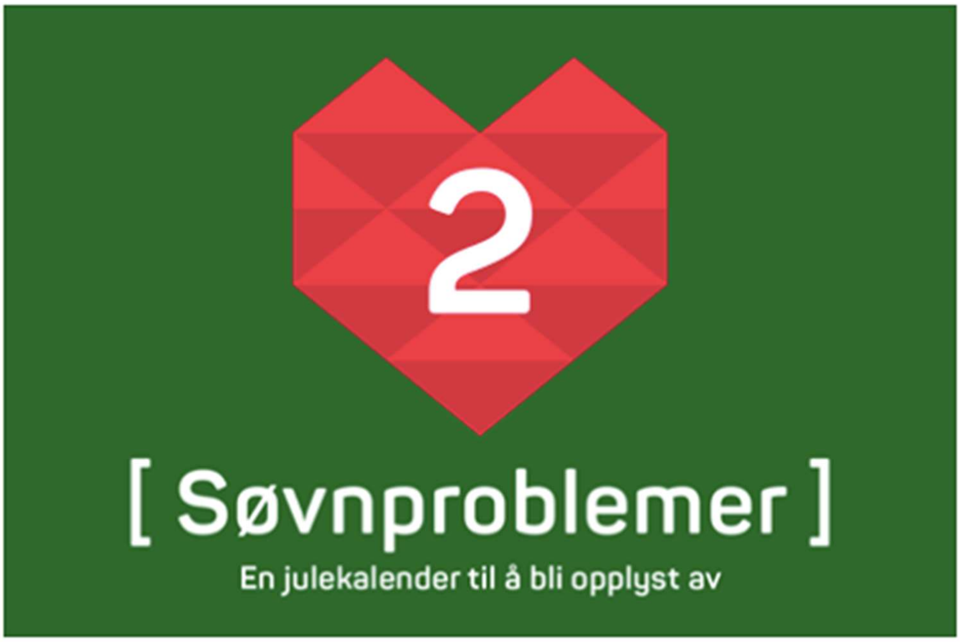

TIPS-INFO
Lokal bedrift

Send melding

Liker Kommenter Del

TIPS-INFO

1. desember 2017 ·

Sykdomstegn. Kan være av fysisk eller psykisk karakter. Følg med på vår julekalender, så blir du opplyst om symptomer på psykiske lidelser. Er du bekymret for deg selv eller andre kan du ringe TIPS på 51 51 59 59 alle hverdager 08:00-15:00 for råd og veiledning.
SØK HJELP SÅ RASKT SOM MULIG,
DA ER SJANSEN STØRST FOR Å BLI FRISK

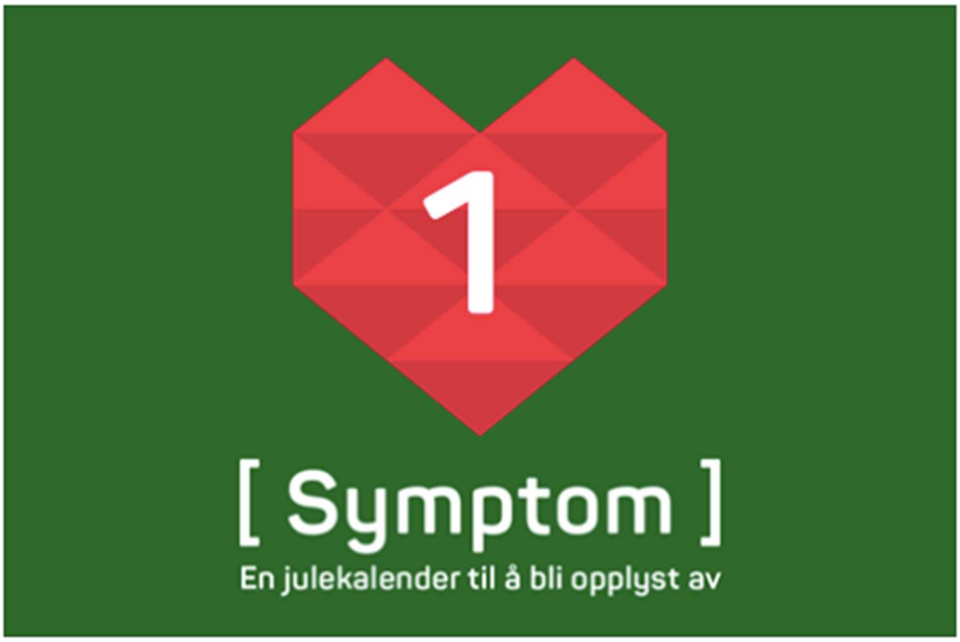

TIPS-INFO

Lokal bedrift

Send melding

Liker Kommenter Del

TIPS-INFO og 8 andre

TIPS-INFO
29. juni 2017 ·

Ferier kan være krevende. Ensomhet. Tosomhet. Venner og familie tett på. Hverdagens rutiner blir satt til side. For noen oppstår gnisninger. Noen skjuler problemer, andre åpner seg. Hvis du opplever at utfordringene er større enn det som kan løses i en fortrolig samtale, kan du be oss om råd. Ta en telefon, gjerne anonymt – det er hjelp å få!

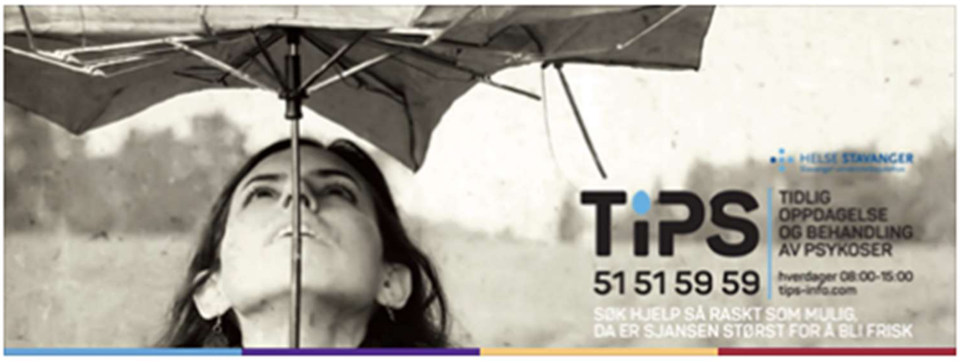

Liker Kommenter Del

TIPS-INFO
3. mai 2017 ·

Mai er russetid, men det er også innspurten til eksamen, så det er mye press og stress. For enkelte blir det for mye. Noen sliter med psykiske problemer. Stort sett forbigående, men for noen få kan det være alvorlig. Hvis du sliter, eller ser andre som gjør det, er det hjelp å få. Ring TIPS for råd og hjelp, for deg selv eller andre. Du kan være anonym om du ønsker det.

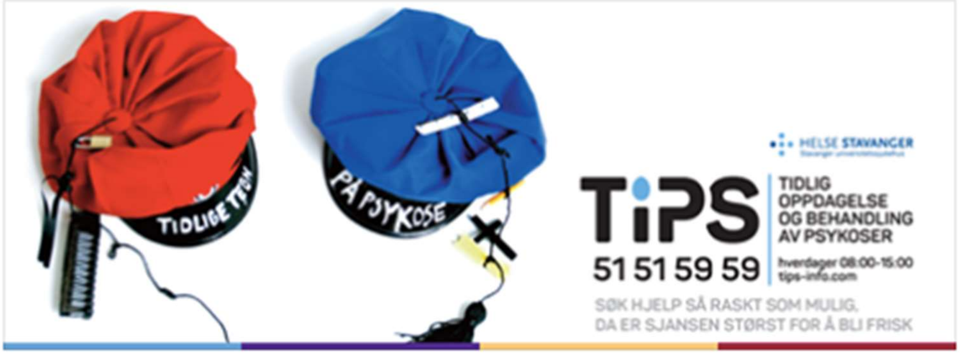

Liker Kommenter Del

TIPS-INFO

9. mars 2017 ·

DETTE BØR ALLE VITE OM PSYKISKE LIDELSER
75 % av alle som får en psykisk lidelse får den før de er 25 år. Psykiske lidelser rammer altså ungdom. Mange venter for lenge med å søke hjelp, og da øker sannsynligheten for at lidelsen blir verre og vanskeligere å helbrede. Søker du hjelp tidlig, er sjansen for å bli frisk av en psykose mer enn dobbelt så stor! Er du bekymret for deg selv eller andre kan du ringe TIPS på 51 51 59 59 alle hverdager 08:00-15:00 for råd og veiledning.
SØK HJELP SÅ RASKT SOM MULIG,
DA ER SJANSEN STØRST FOR Å BLI FRISK

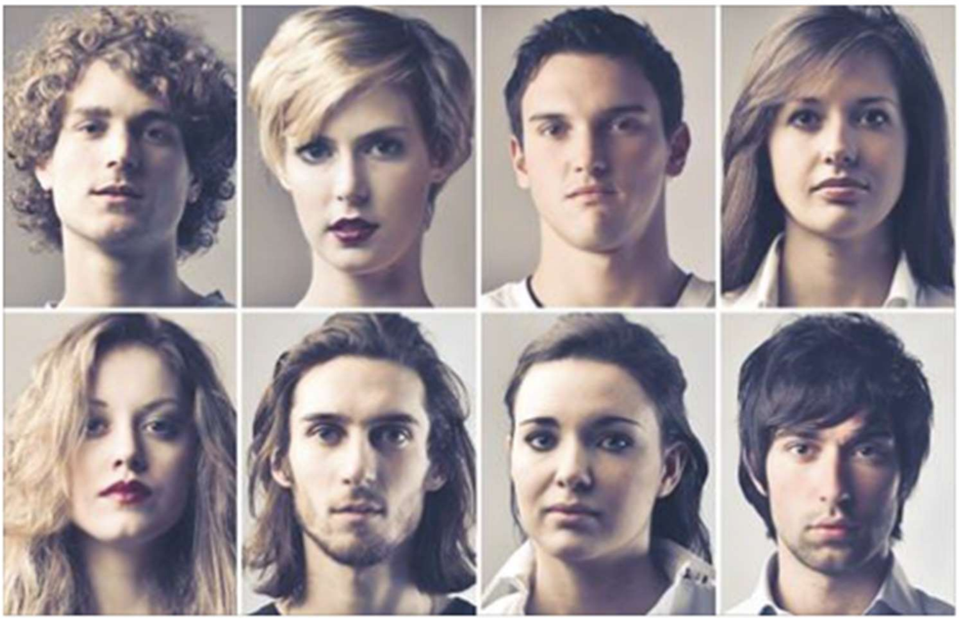

TIPS-INFO

Finn ut mer

TIPS-INFO
25. januar 2017 ·

DETTE BØR ALLE VITE OM PSYKISKE LIDELSER
I disse dager sender NRK en serie om unge mennesker med schizofreni. Den heter «Stemmene i hodet», og vi oppfordrer alle til å se den! 75 % av alle som får en psykisk lidelse får den før de er 25 år. Psykiske lidelser rammer altså ungdom. Mange venter for lenge med å søke hjelp, og da øker sannsynligheten for at lidelsen blir verre og vanskeligere å helbrede. Søker du hjelp tidlig, er sjansen for å bli frisk av en psykose mer enn dobbelt så stor! Er du bekymret for deg selv eller andre kan du ringe TIPS på 51 51 59 59 alle hverdager 08:00-15:00 for råd og veiledning.
SØK HJELP SÅ RASKT SOM MULIG,
DA ER SJANSEN STØRST FOR Å BLI FRISK

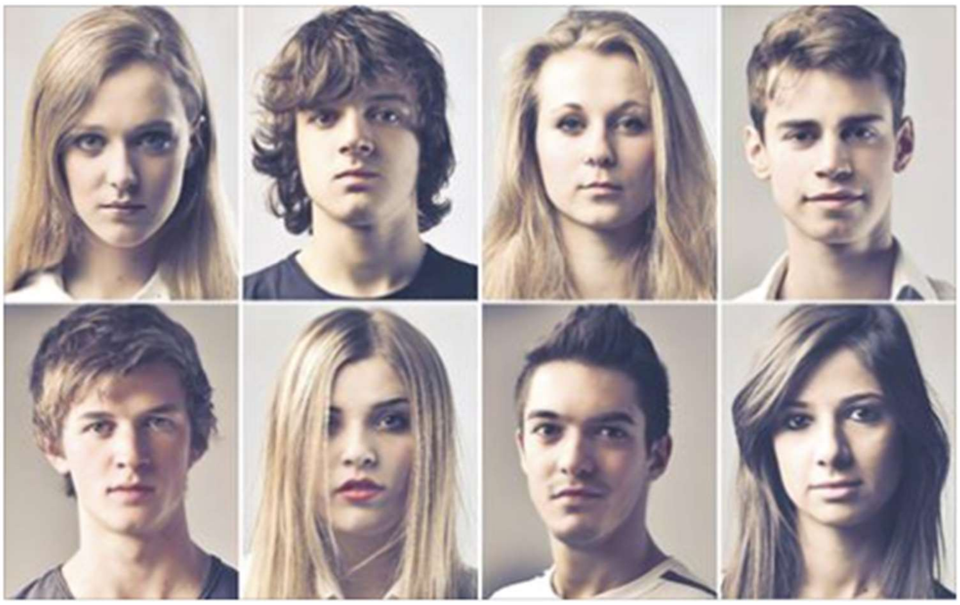

Liker Kommenter Del

TIPS-INFO
25. januar 2017 ·

Kanskje noen har spørsmål om psykoser etter å ha sett "Stemmene i hodet" på NRK i kveld? Våre erfarne fagfolk gir råd, hjelp og veiledning på telefon 51 51 59 59 fra 08:00 til 15:00 alle hverdager.

To av åtte tenåringer skjuler at de sliter med psykiske problemer. Kan du se hvem?

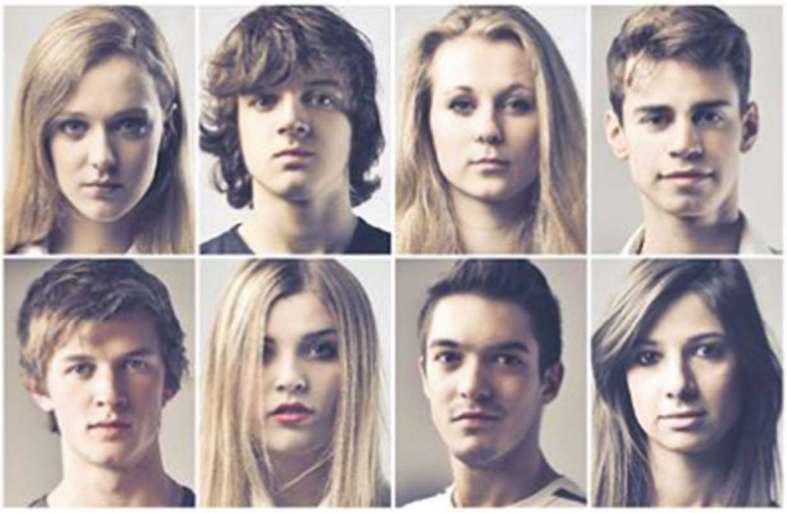

TEGN PÅ PSYKISKE LIDELSER	HVA DU BØR GJØRE
Isolerer seg<br>Sover dårlig<br>Angst	Dette er tegn som kan være forbigående, men varer de i flere uker bør du ringe for å få råd.
Tristhet<br>Konsentrasjonsvansker<br>Forsømmer hygiene, jobb eller skole	Problemene går ut over jobb og skole. Venner og familie blir bekymret. Ring for å få råd for deg selv eller noen du kjenner.
Store humørsvingninger<br>Tankene høres ut som stemmer	Her er lidelsen blitt alvorlig og eneste mulighet er å søke profesjonell hjelp.

Store humørsvingninger
Tankene høres ut som stemmer
Her er lidelsen blitt alvorlig og eneste mulighet er å søke profesjonell hjelp.

Snakker usammenhengende<br>Føler seg forfulgt eller styrt av andre<br>Ekstremt opptatt av temaer som døden, politikk eller religion	Ringer du TIPS kan vi bl.a. bistå med hjemmebesøk og sørge for at den det gjelder får den hjelp som er nødvendig for å bli frisk.

Det kan være vanskelig å vite når en bør søke hjelp. Ved en mistanke om at det kan være en psykisk lidelse bør en kontakte TIPS for råd og veiledning så fort som mulig. Les mer om psykoser, hjelp og behandling på våre nettsider tips-info.com.

TIPS
51 51 59 59
TIDLIG OPPDAGELSE OG BEHANDLING AV PSYKOSER

Dette bør alle vite om psykiske lidelser

"Stemmene i hodet" starter i kveld på NRK1 kl 22.15.

I kveld sender NRK det første programmet i en serie om unge mennesker med schizofreni. Den er sterk og gripende. Den heter «Stemmene i hodet», og vi oppfordrer alle til å se den! Schizofreni er en psykoselidelse, og som du kan se, er det en alvorlig sykdom.

Dette visste du sannsynligvis ikke: 75 % av alle som får en psykisk lidelse får den før de er 25 år. Psykiske lidelser rammer altså ungdom. Først en dårlig nyhet: Mange venter for lenge med å søke hjelp, og da øker sannsynligheten for at lidelsen blir verre og vanskeligere å helbrede. Den gode nyheten er: Hvis du søker hjelp tidlig, er sjansen for å bli frisk av en psykose mer enn dobbelt så stor! Visste du at de aller fleste som får behandling fortsetter å bo hjemme og gå på jobb eller skole.

Liker Kommenter Del

TIPS-INFO har oppdatert forsidebildet sitt.

24. januar 2017 ·

Sjansen for å bli frisk av en psykose er dobbelt så stor hvis en får rask hjelp

Er du bekymret for deg selv eller andre kan du ringe TIPS på 51 51 59 59 alle hverdager 08:00-15:00 for råd og veiledning.

Liker Kommenter Del

TIPS-INFO har oppdatert forsidebildet sitt.

16. januar 2017 ·

[Er du bekymret for deg selv eller andre kan du ringe TIPS på 51 51 59 59 alle hverdager 08:00-15:00 for råd og veiledning.]

Liker Kommenter Del

TIPS-INFO
4. januar 2017 ·

GODT NYTTÅR!
Vi har nettopp gått gjennom en tid med store forventninger, mye stress og vært tett innpå hverandre. Noen kan få det det vanskelig. Den som sliter, sliter kanskje litt ekstra? Noen trenger kanskje hjelp, men du vet ikke helt hva du skal gjøre? Er du bekymret for deg selv eller andre kan du ringe TIPS på 51 51 59 59 alle hverdager 08:00-15:00 for råd og veiledning.
SØK HJELP SÅ RASKT SOM MULIG,
DA ER SJANSEN STØRST FOR Å BLI FRISK

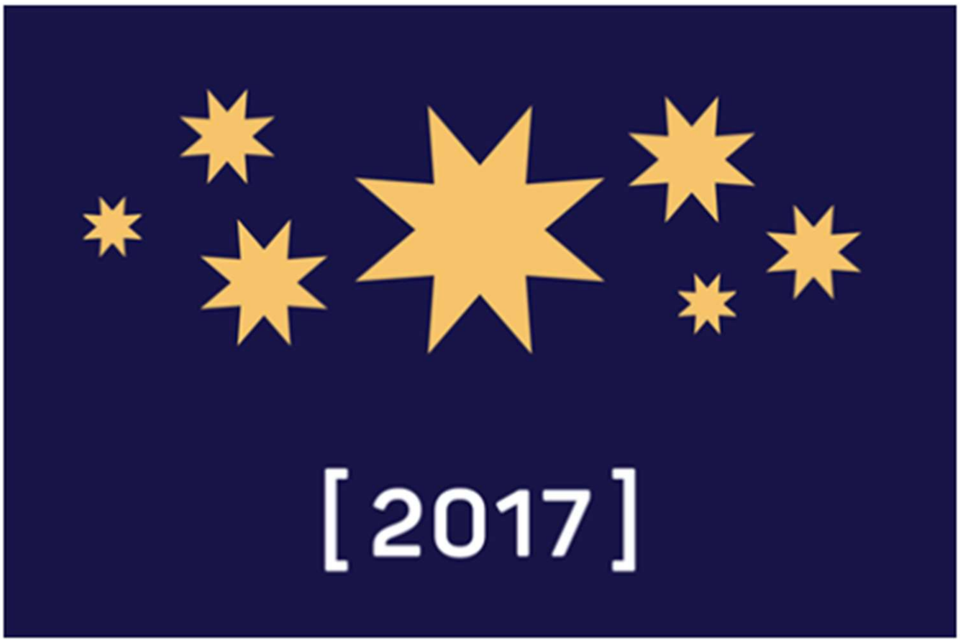

Liker Kommenter Del

152 Kronologisk

TIPS-INFO
27. desember 2016 ·

Vi ønsker alle en god jul…men, så nådde den kanskje ikke helt opp til forventningene? Store forventninger. Mye stress. Tett innpå hverandre. Så får noen det vanskelig… Den som sliter, sliter kanskje litt ekstra? Noen trenger kanskje hjelp, men du vet ikke helt hva du skal gjøre? Er du bekymret for deg selv eller andre kan du ringe TIPS på 51 51 59 59 alle hverdager i romjulen 08:00-15:00 for råd og veiledning.
SØK HJELP SÅ RASKT SOM MULIG,
DA ER SJANSEN STØRST FOR Å BLI FRISK

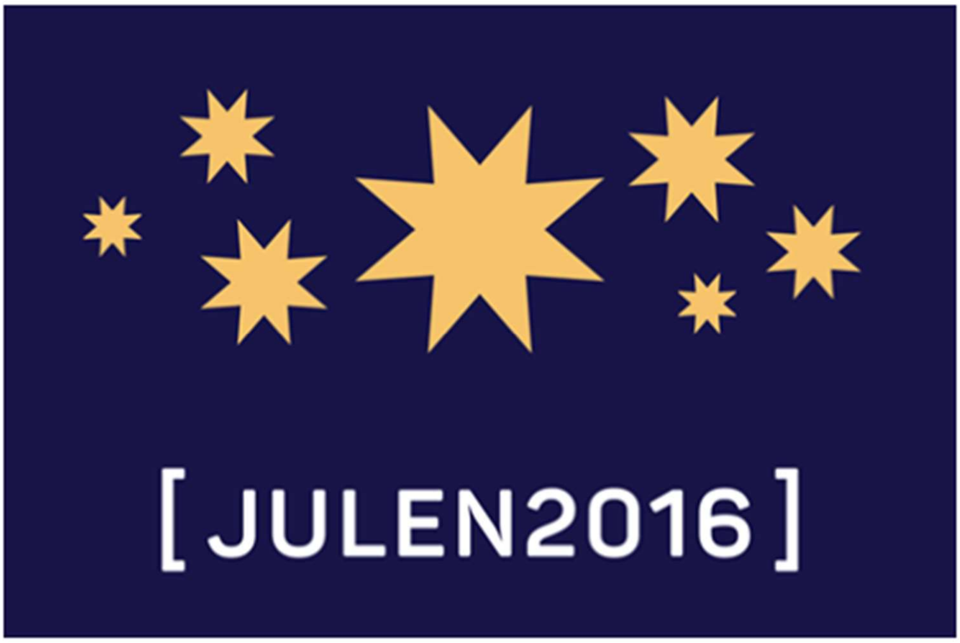

Liker Kommenter Del

TIPS-INFO har oppdatert forsidebildet sitt.
24. desember 2016 ·

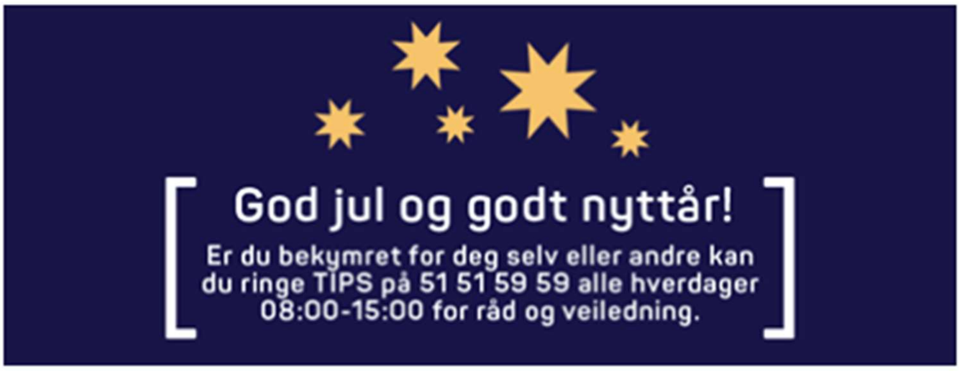

Liker Kommenter Del

TIPS-INFO
24. desember 2016 ·

Prognose er en forventning om hvordan noe vil utvikle seg. Innen helsefag er en på jakt etter det som øker sannsynligheten for å bli frisk. En vil en basere seg på tidligere erfaring, forskning og ulike tiltak som fremmer helse. Tidlig oppdagelse og behandling av psykose gir god prognose. Er du bekymret for deg selv eller andre kan du ringe TIPS på 51 51 59 59 alle hverdager 08:00-15:00 for råd og veiledning.
SØK HJELP SÅ RASKT SOM MULIG,
DA ER SJANSEN STØRST FOR Å BLI FRISK

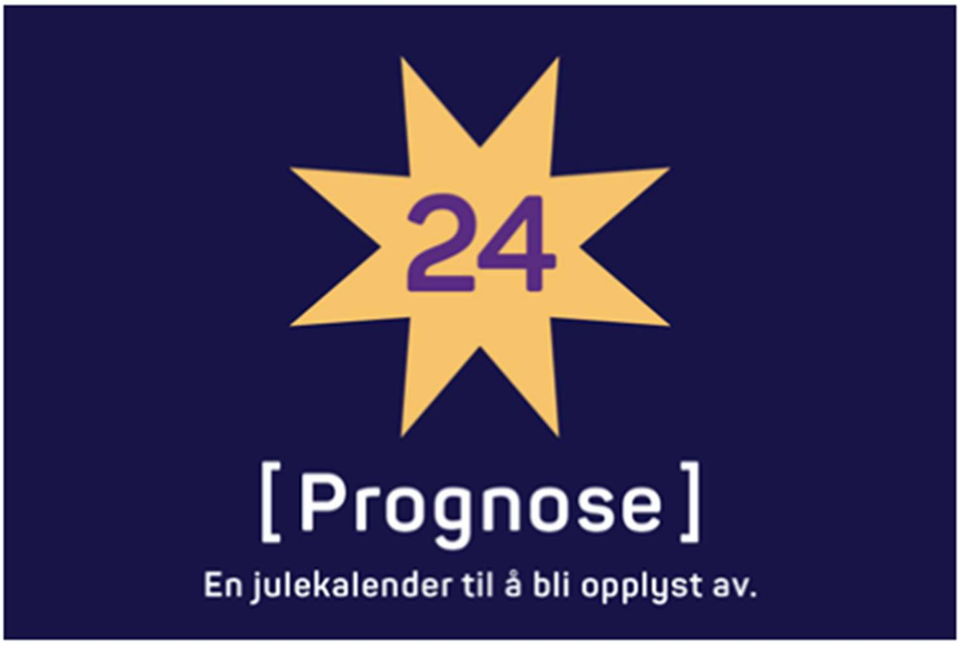

Liker Kommenter Del

TIPS-INFO
23. desember 2016 ·

Tidlig intervensjon betyr å gripe inn tidlig når en er bekymret for et annet menneske. I psykisk helsearbeid betyr det å oppdage og behandle lidelser så raskt som mulig. Sjansen for å bli helt frisk fra f.eks. en psykose, øker jo tidligere en får behandling. Psykiske lidelser oppstår først og fremst i ungdomsårene. Er du bekymret for deg selv eller andre kan du ringe TIPS på 51 51 59 59 alle hverdager 08:00-15:00 for råd og veiledning.
SØK HJELP SÅ RASKT SOM MULIG,
DA ER SJANSEN STØRST FOR Å BLI FRISK

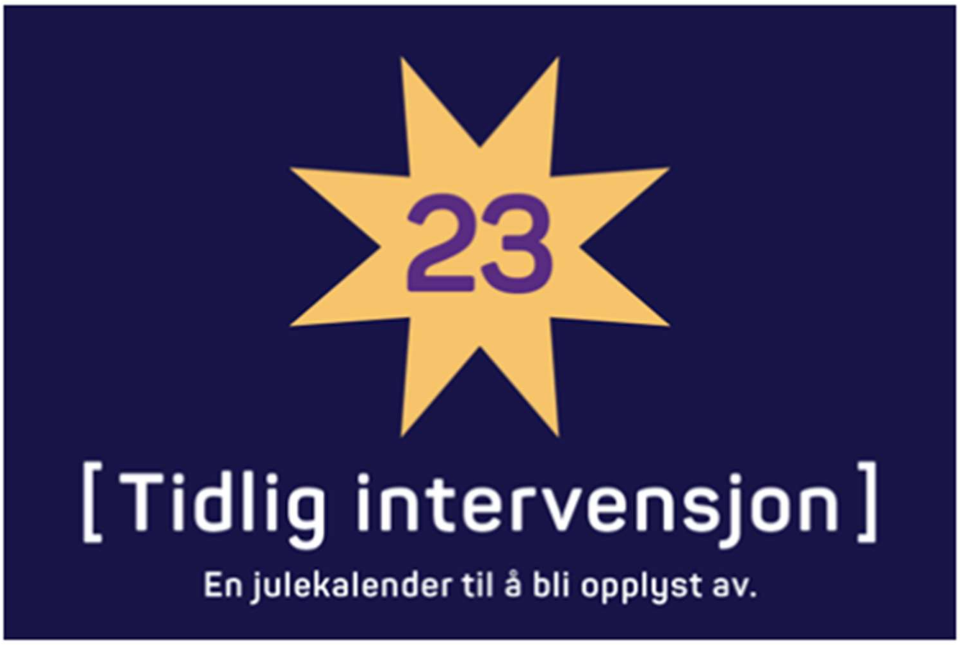

Liker Kommenter Del

TIPS-INFO

22. desember 2016 ·

Psykoser kan betraktes som alvorlige «forvirringstilstander», og betegner egentlig svært dyptgående nervøse sammenbrudd. De tydeligste symptomer er hallusinasjoner, vrangforestillinger og forfølgelsesideer. Angst, isolasjon, forvirring er ofte tidlige tegn, men vil ikke nødvendigvis utvikle seg til psykose. Det er likevel viktig å søke tidlig hjelp, da sjansen for å stanse utviklingen er størst når psykosen oppdages og behandles tidlig. Er du bekymret for deg selv eller andre kan du ringe TIPS på 51 51 59 59 alle hverdager 08:00-15:00 for råd og veiledning.

SØK HJELP SÅ RASKT SOM MULIG,

DA ER SJANSEN STØRST FOR Å BLI FRISK

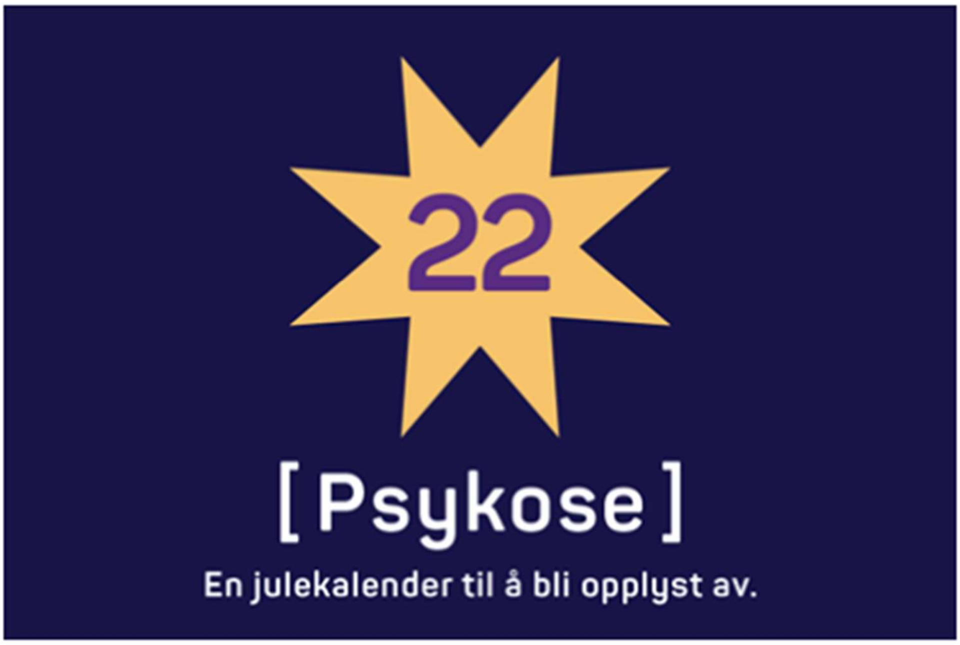

TIPS-INFO
21. desember 2016 ·

Aggresjon forklares gjerne som oppførsel der hensikten å ydmyke, skade eller påføre smerte. Frustrasjon, irritasjon og raseri er ulike varianter av aggresjon. Aggresjon uten en åpenbar årsak kan være et signal om at noe er i veien. Tilsynelatende umotivert aggresjon kan være blant tidlige symptomer på psykose. Er du bekymret for deg selv eller andre kan du ringe TIPS på 51 51 59 59 alle hverdager 08:00-15:00 for råd og veiledning.
SØK HJELP SÅ RASKT SOM MULIG,
DA ER SJANSEN STØRST FOR Å BLI FRISK

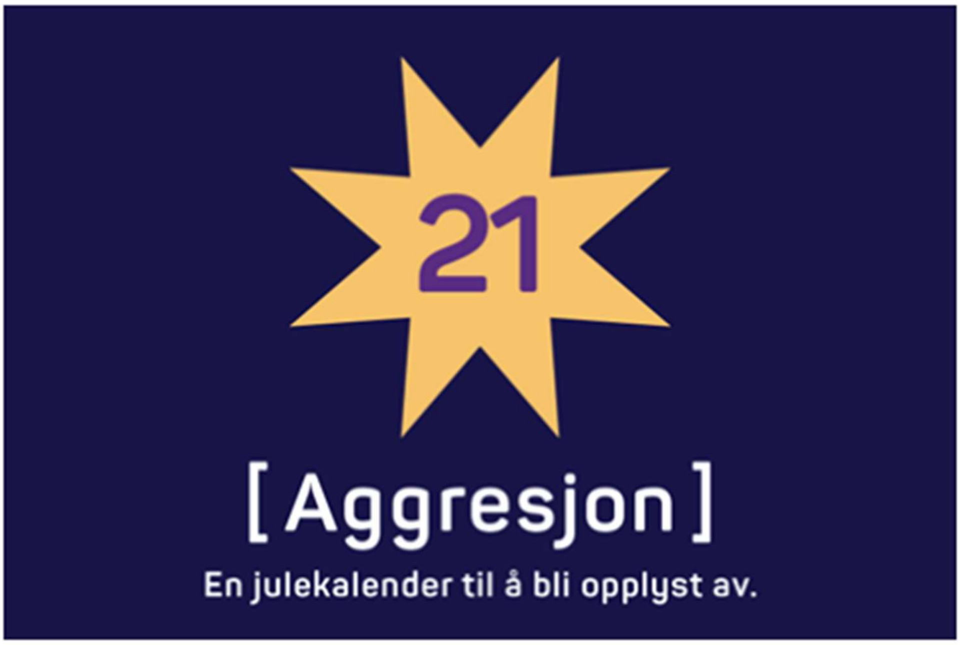

Liker Kommenter Del

TIPS-INFO

20. desember 2016 ·

20.12.2016 00:21

Positive s[illegible]orklares med at noe kommer i tillegg til vanlige følelser og sanseopplevelser. Det kan være sterke, umotiverte følelsesutbrudd, panikk, hallusinasjoner og storhetstanker. Positive symptomer er typiske for psykoselidelser. Er du bekymret for deg selv eller andre kan du ringe TIPS på 51 51 59 59 alle hverdager 08:00-15:00 for råd og veiledning.
SØK HJELP SÅ RASKT SOM MULIG,
DA ER SJANSEN STØRST FOR Å BLI FRISK

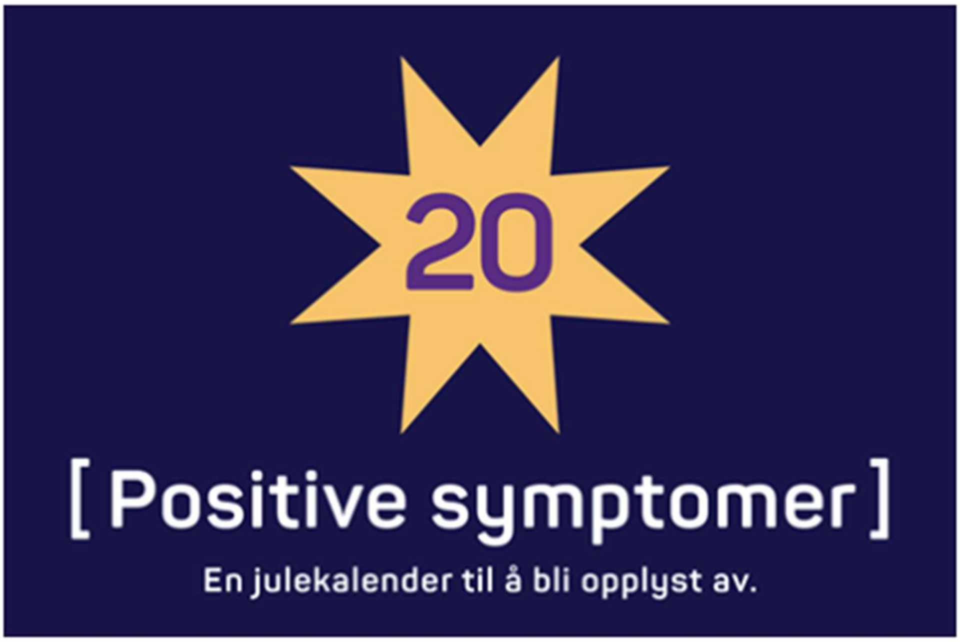

Liker Kommenter Del

TIPS-INFO
19. desember 2016 ·

Negative symptomer kan vi forklare med at noe forsvinner. En mister evnen til å oppleve og uttrykke følelser, f.eks. ingen glede eller sinne. En kan bli likegyldig, tiltaksløs og miste engasjement. Kan være de første tegnene på en alvorlig psykisk lidelse. Psykoser starter gjerne med negative symptomer. Er du bekymret for deg selv eller andre kan du ringe TIPS på 51 51 59 59 alle hverdager 08:00-15:00 for råd og veiledning.
SØK HJELP SÅ RASKT SOM MULIG,
DA ER SJANSEN STØRST FOR Å BLI FRISK

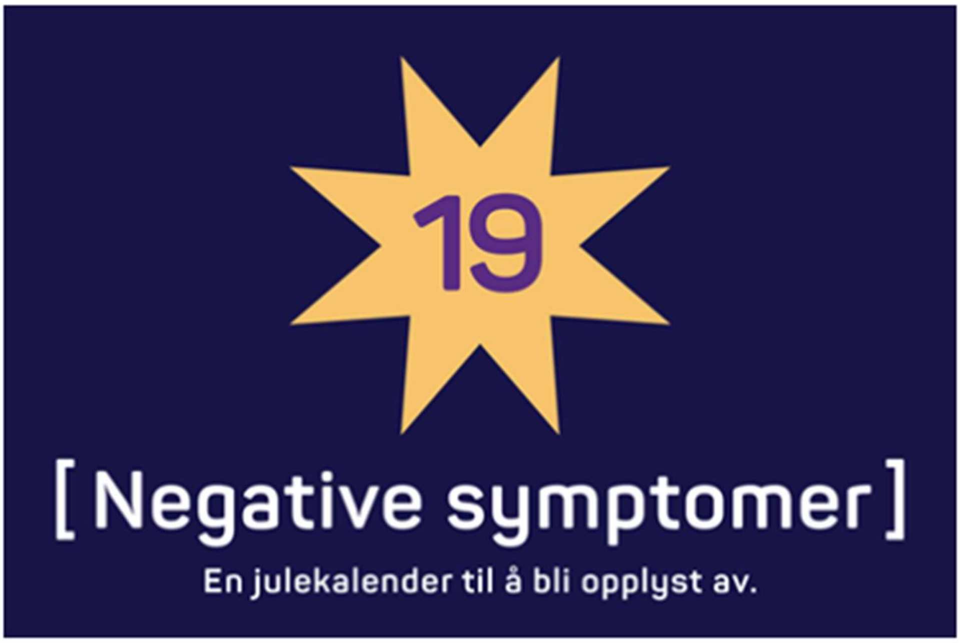

Liker Kommenter Del

TIPS-INFO
18. desember 2016 ·

Avmakt er følelsen av å mangle evne eller krefter til å endre på en situasjon. Det er ikke en diagnose, men vi tar det med i kalenderen for å minne om at det er viktig å søke hjelp tidlig, og at de aller fleste som får en psykisk lidelse blir friske. Avmakt kan være en del av en psykisk lidelse og hindre noen i å oppsøke hjelp. Er du bekymret for venner, kjente eller familie kan du ringe TIPS på 51 51 59 59 alle hverdager 08:00-15:00 for råd og veiledning.
SØK HJELP SÅ RASKT SOM MULIG,
DA ER SJANSEN STØRST FOR Å BLI FRISK

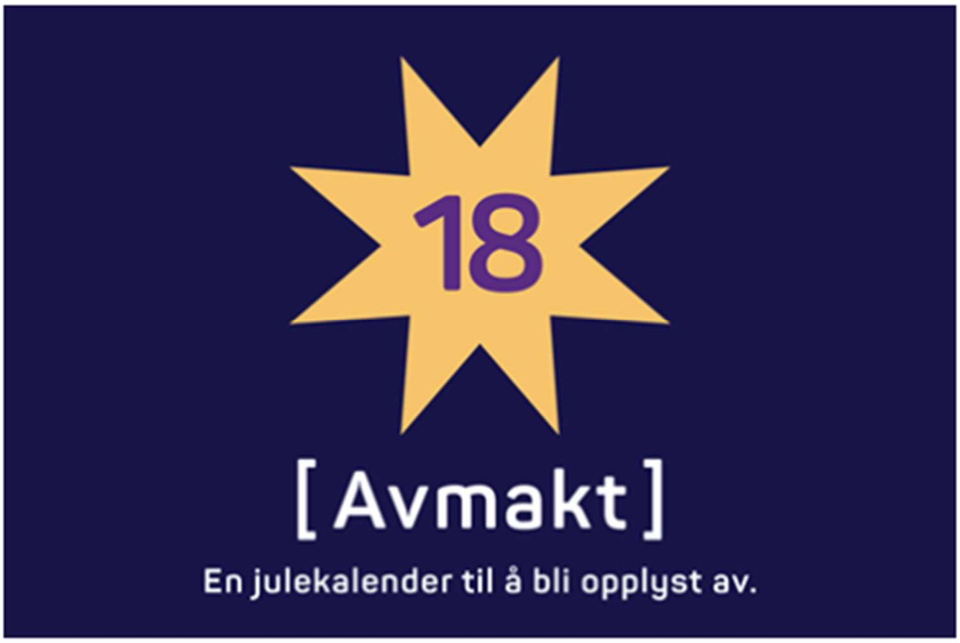

Liker Kommenter Del

TIPS-INFO

17. desember 2016 ·

Tankepåvirkning er opplevelsen at tankene blir styrt utenfra, at en blir gitt eller tatt fra tanker. En kan føle at andre kan lese tankene. Noen kaller det tankekjør, da ofte i betydningen at tankene spinner raskt og ukontrollert. Tankepåvirkning forekommer ofte ved psykoser. Er du bekymret for deg selv eller andre kan du ringe TIPS på 51 51 59 59 alle hverdager 08:00-15:00 for råd og veiledning.
SØK HJELP SÅ RASKT SOM MULIG,
DA ER SJANSEN STØRST FOR Å BLI FRISK

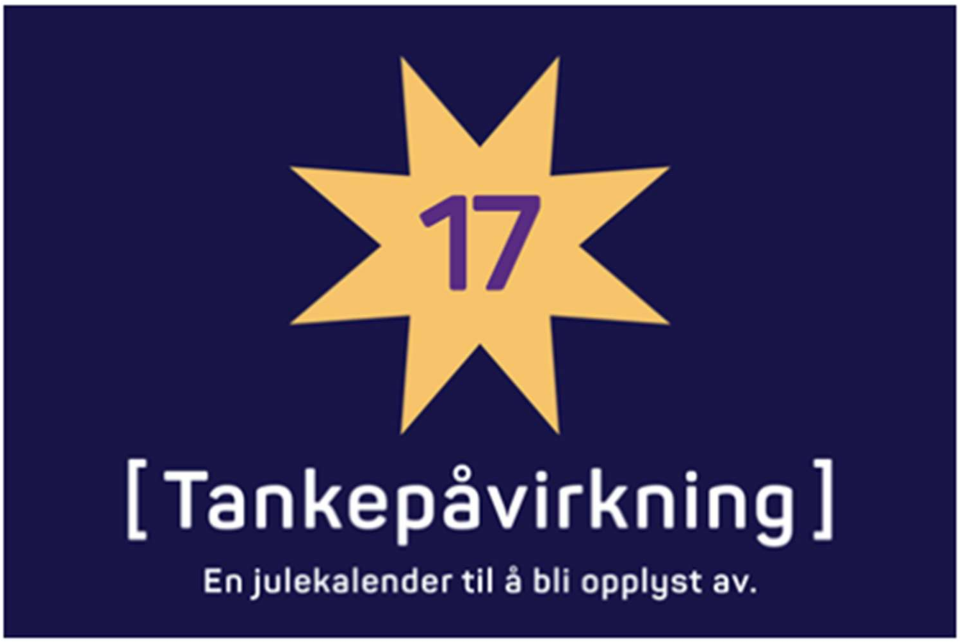

Liker Kommenter Del

TIPS-INFO
16. desember 2016 ·

Traume er en skade. Psykisk traume er naturlige reaksjoner på unaturlige hendelser. Vi kjenner det fra bl.a. krig, tortur og overgrep. Gjentatte eller langvarige traumer kan føre til psykiske forstyrrelser som preger og ødelegger livskvaliteten. Traumer kan også forårsake psykoser. Er du bekymret for deg selv eller andre kan du ringe TIPS på 51 51 59 59 alle hverdager 08:00-15:00 for råd og veiledning.
SØK HJELP SÅ RASKT SOM MULIG,
DA ER SJANSEN STØRST FOR Å BLI FRISK

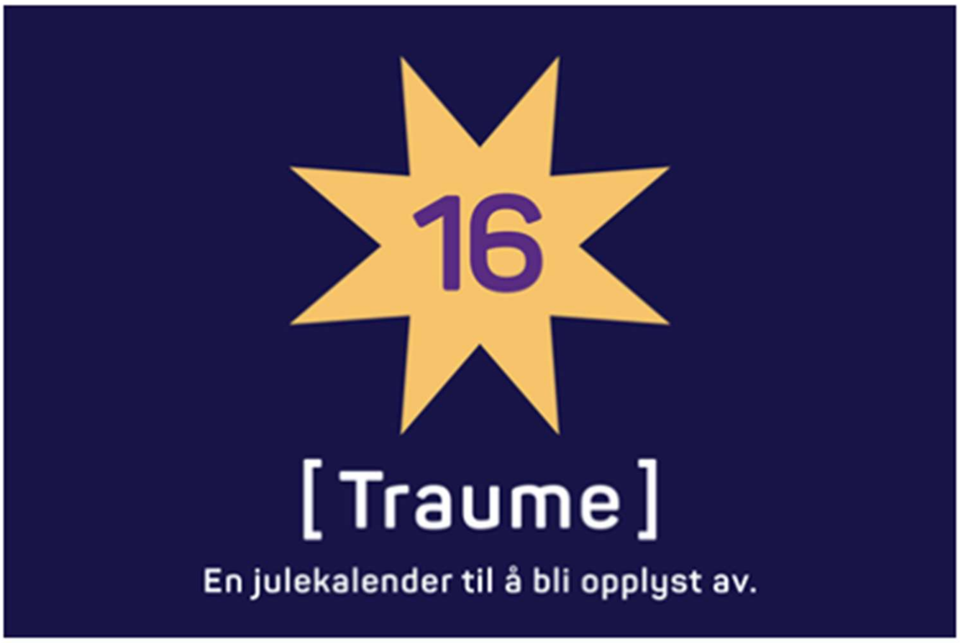

Liker Kommenter Del

TIPS-INFO
15. desember 2016 ·

Storhetstanker er en forstilling om å ha spesielle evner eller oppgaver å utføre. En kan tro at en har magiske evner, er en viktig religiøs leder, skal utføre fantastiske bragder, som f.eks. å bygge gullslott. Storhetstanker opptrer ved flere ulike psykiske lidelser, men er særlig tydelig ved psykoser. Er du bekymret for deg selv eller andre kan du ringe TIPS på 51 51 59 59 alle hverdager 08:00-15:00 for råd og veiledning.
SØK HJELP SÅ RASKT SOM MULIG,
DA ER SJANSEN STØRST FOR Å BLI FRISK

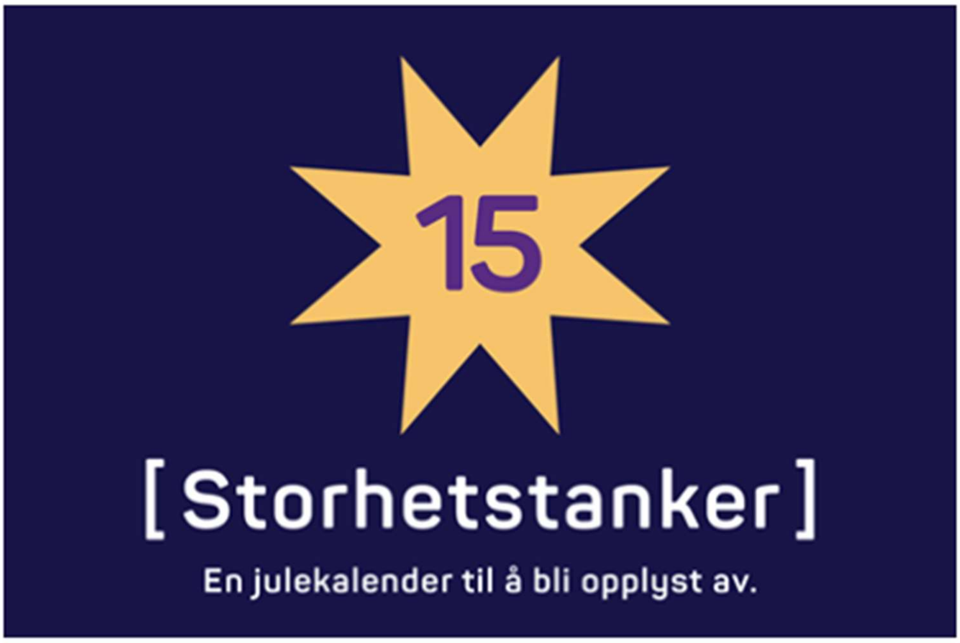

Liker Kommenter Del

TIPS-INFO

14. desember 2016 ·

Paranoia er en tilstand som preges av at tanker og følelser er bundet opp til en forestilling om at en er utsatt for forfølgelse eller en sammensvergelse. Noen lever greit med lidelsen så lenge den ikke blir utfordret, andre blir sterkt preget av sine vrangforestillinger. Paranoia kan også være et tegn på en psykoselidelse, særlig hos yngre mennesker. Er du bekymret for deg selv eller andre kan du ringe TIPS på 51 51 59 59 alle hverdager 08:00-15:00 for råd og veiledning.

SØK HJELP SÅ RASKT SOM MULIG,
DA ER SJANSEN STØRST FOR Å BLI FRISK

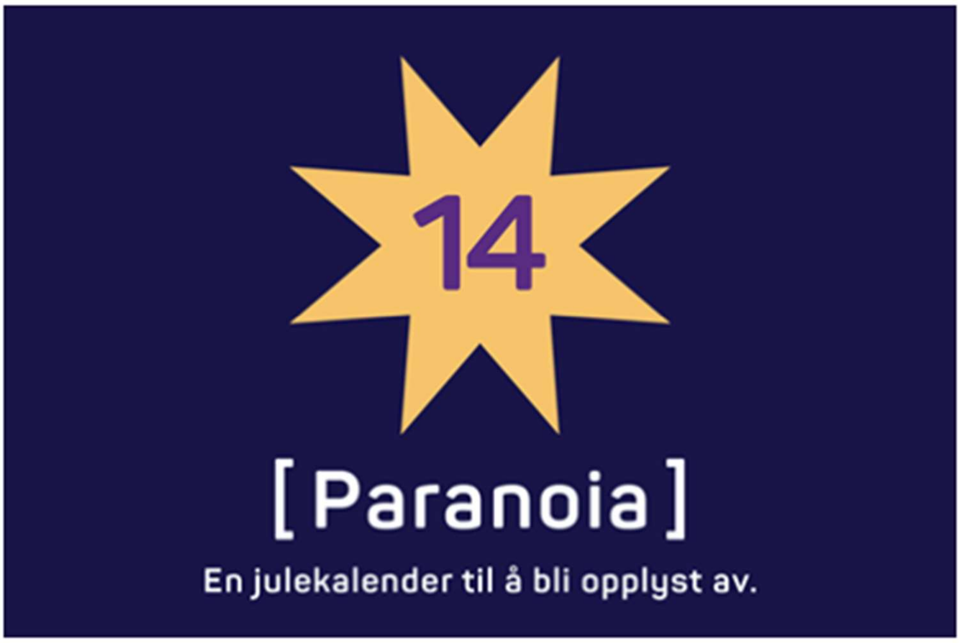

Liker Kommenter Del

TIPS-INFO

13. desember 2016 ·

Stress er ikke bare en forbigående travelhet. Stress som et negativt fenomen oppstår når en opplever at krav og forventninger overstiger egen kapasitet og mestring og kan være en del av flere psykiske og fysiske lidelser. Stress kan også utløse psykoser. Er du bekymret for deg selv eller andre kan du ringe TIPS på 51 51 59 59 alle hverdager 08:00-15:00 for råd og veiledning.
SØK HJELP SÅ RASKT SOM MULIG,
DA ER SJANSEN STØRST FOR Å BLI FRISK

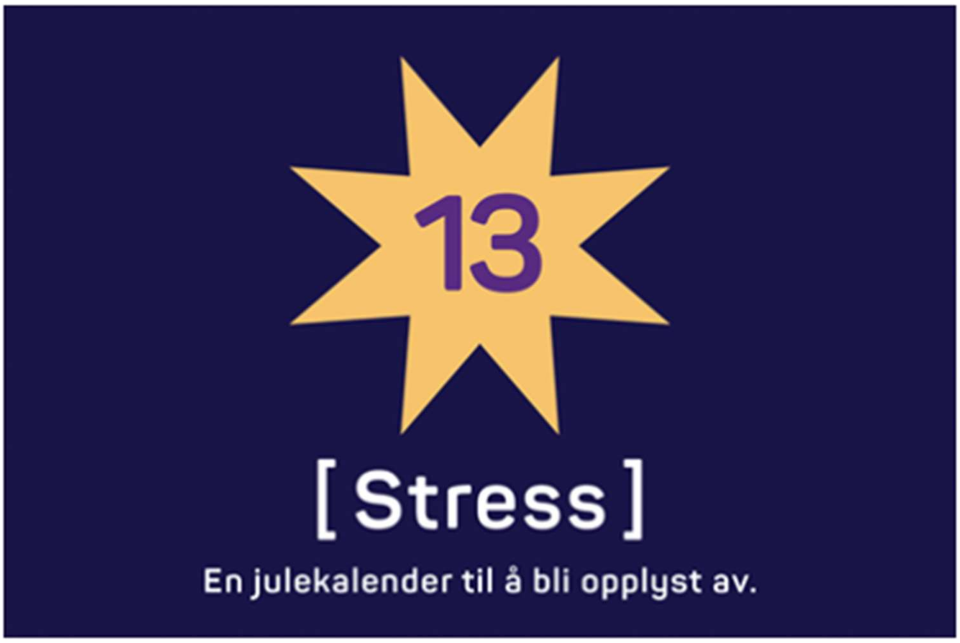

Liker Kommenter Del

TIPS-INFO

12. desember 2016 ·

Isolasjon er ikke det samme som ensomhet. Når en trekker seg tilbake, unngår kontakt med venner, kjente og familie, uteblir fra jobb og skole fordi en har ubehag, føler seg nedfor og deprimert kan det være et tegn på andre problemer. At en isolerer seg kan være et av de tidlige tegnene på psykoselidelser. Er du bekymret for deg selv eller andre kan du ringe TIPS på 51 51 59 59 alle hverdager 08:00-15:00 for råd og veiledning.
SØK HJELP SÅ RASKT SOM MULIG,
DA ER SJANSEN STØRST FOR Å BLI FRISK

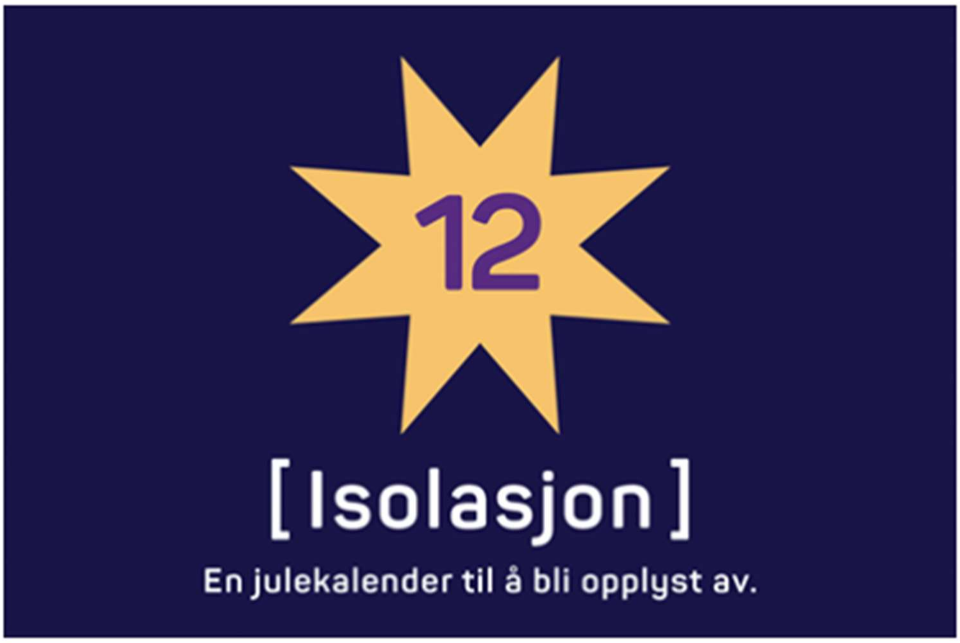

Liker Kommenter Del

TIPS-INFO

11. desember 2016 ·

Tvangstanker beskriver påtrengende og uønskete tanker som stadig gjentar seg og som er vanskelige å kontrollere. Tvangstanker kan føre til handlinger som en føler seg tvunget til å utføre. Psykoselidelser kan ha tvangstanker som en del av symptomene. Er du bekymret for deg selv eller andre kan du ringe TIPS på 51 51 59 59 alle hverdager 08:00-15:00 for råd og veiledning.
SØK HJELP SÅ RASKT SOM MULIG,
DA ER SJANSEN STØRST FOR Å BLI FRISK

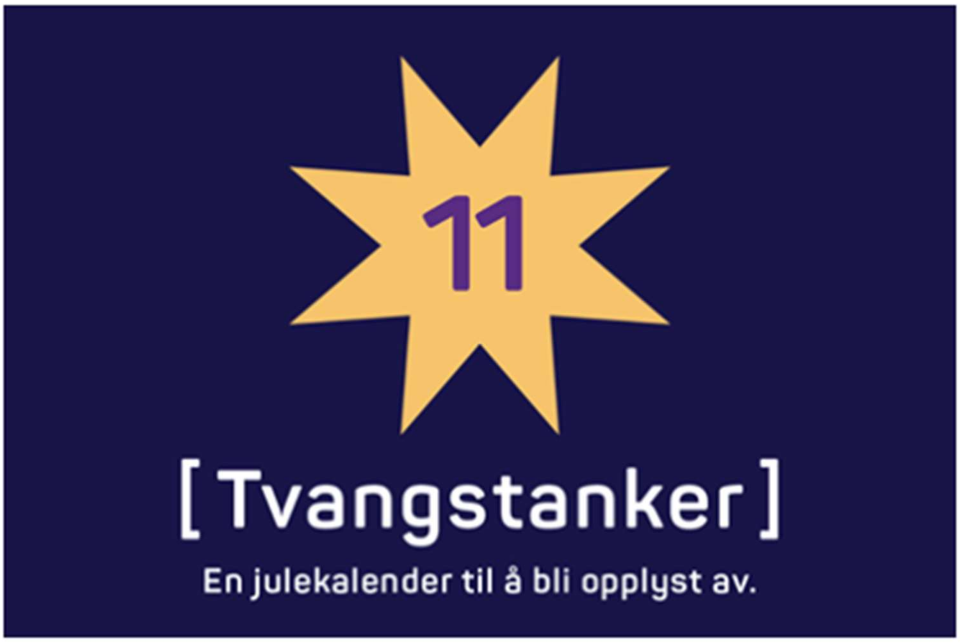

Liker Kommenter Del

TIPS-INFO
10. desember 2016 ·

Stemmehøring forklares gjerne med at man «hører» tanker som virkelige stemmer som kommer utenfra. Slike «stemmer» kan være gode, slemme eller nøytrale. Noen lever greit med slike «stemmer», men for de fleste er de svært plagsomme.Stemmehøring er et typisk tegn på en psykoselidelse.
Er du bekymret for deg selv eller andre kan du ringe TIPS på 51 51 59 59 alle hverdager 08:00-15:00 for råd og veiledning.
SØK HJELP SÅ RASKT SOM MULIG,
DA ER SJANSEN STØRST FOR Å BLI FRISK

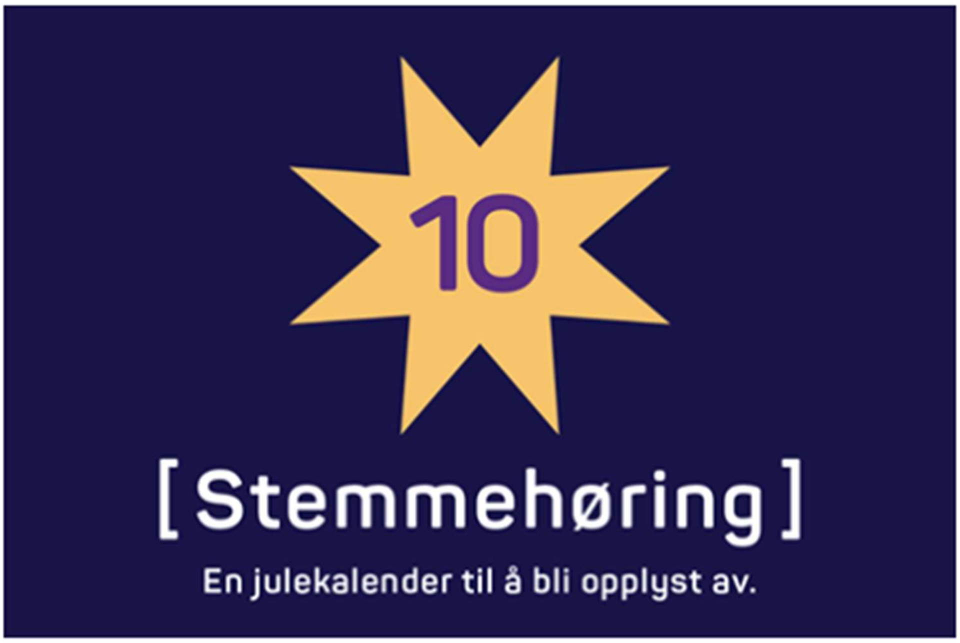

Liker Kommenter Del

TIPS-INFO
9. desember 2016 ·

Hallusinasjoner er sanseopplevelser som ikke er skapt av ytre sanseinntrykk. Eksempler er at en ser, hører, smaker eller lukter ting som ingen andre opplever. Hallusinasjoner kan utløses av rusmidler, men er ofte tegn på en psykose. Er du bekymret for deg selv eller andre kan du ringe TIPS på 51 51 59 59 alle hverdager 08:00-15:00 for råd og veiledning.
SØK HJELP SÅ RASKT SOM MULIG,
DA ER SJANSEN STØRST FOR Å BLI FRISK

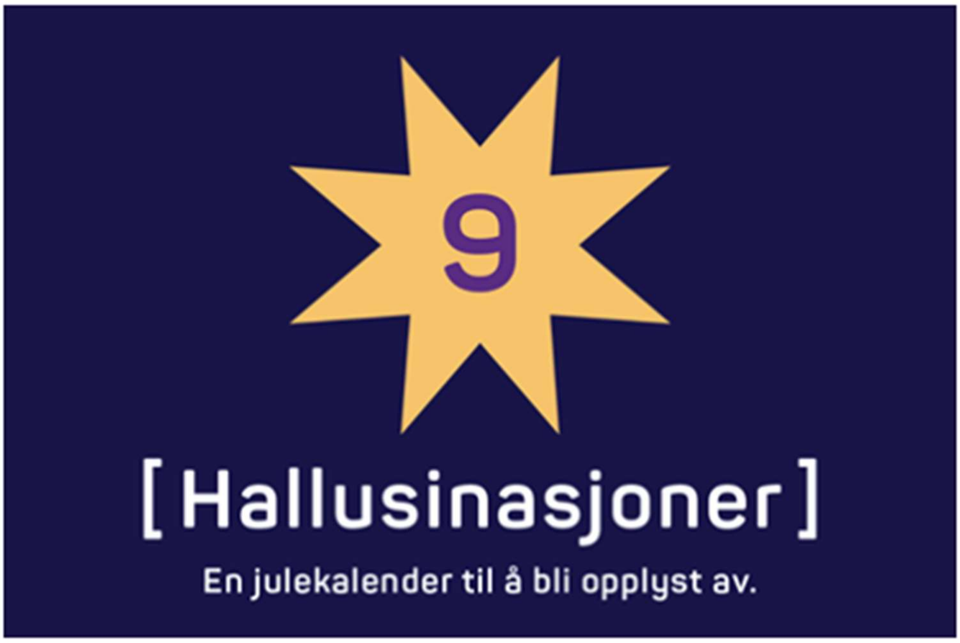

Liker Kommenter Del

TIPS-INFO
8. desember 2016 ·

Depresjon er en tilstand med interesse- og gledesløshet, tretthet og tunge tanker. Ofte med lav selvfølelse, triste tanker og pessimisme. Tanker om selvmord er ikke uvanlig. Kan oppleves som en fysisk smerte. Depresjon kan også være et tegn på en psykoselidelse. Er du bekymret for deg selv eller andre kan du ringe TIPS på 51 51 59 59 alle hverdager 08:00-15:00 for råd og veiledning.
SØK HJELP SÅ RASKT SOM MULIG,
DA ER SJANSEN STØRST FOR Å BLI FRISK

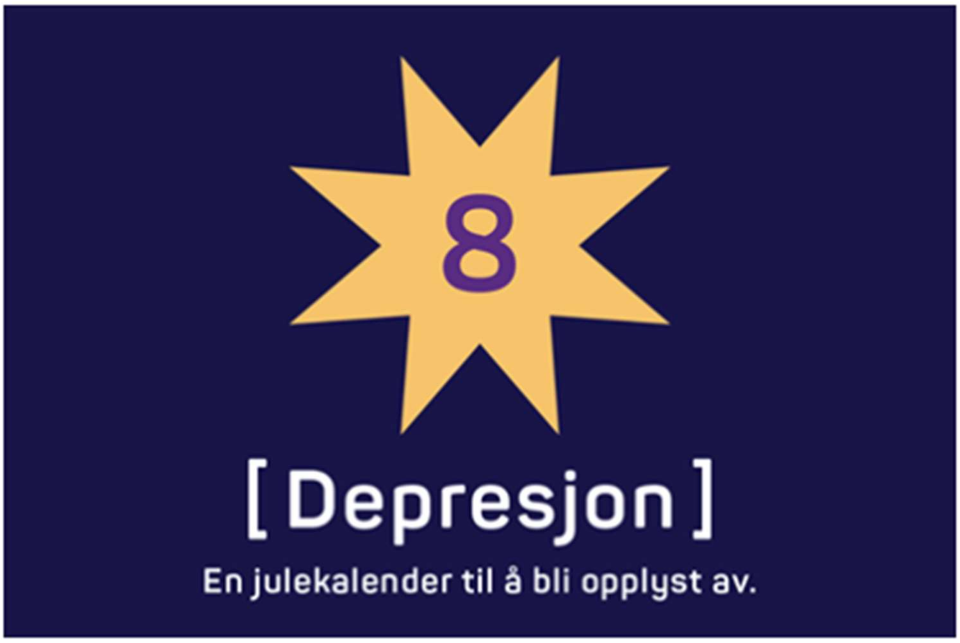

Liker Kommenter Del

TIPS-INFO
7. desember 2016 ·

Mani er en tilstand der stemnings- og energinivå er langt over normalen. Kommer gjerne i perioder på uker og måneder, med uvettig bruk av penger, urealistiske planer, storslagne prosjekter og manglende selvkritikk. Mani kan avløses av depresjon eller gå over i psykose. Er du bekymret for deg selv eller andre kan du ringe TIPS på 51 51 59 59 alle hverdager 08:00-15:00 for råd og veiledning.
SØK HJELP SÅ RASKT SOM MULIG,
DA ER SJANSEN STØRST FOR Å BLI FRISK

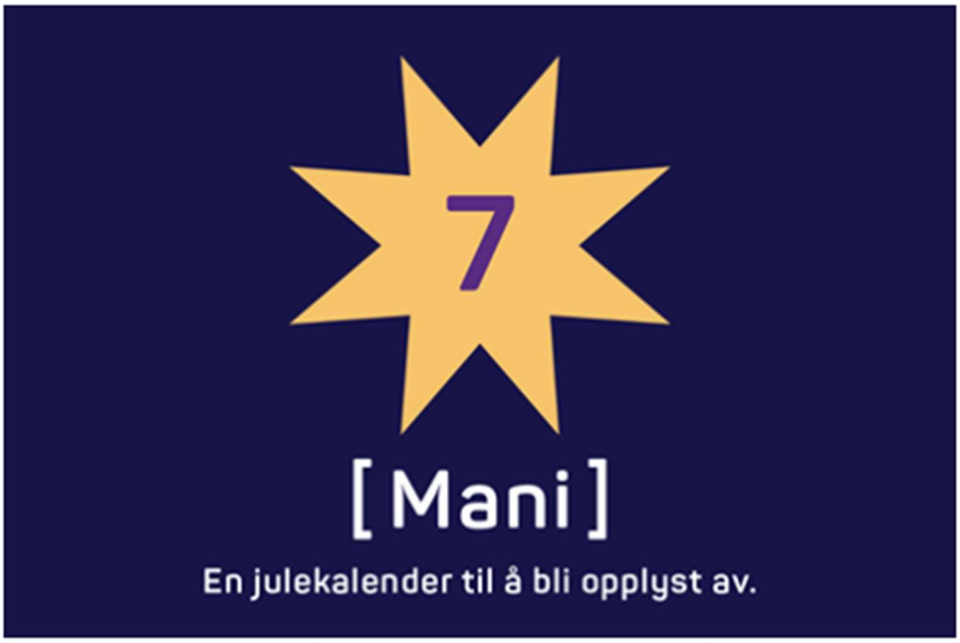

Liker Kommenter Del

TIPS-INFO
6. desember 2016 ·

Sterke følelser kan være en naturlig ting: gledelig overrasket, rettferdig harme, kjærlighetserklæring, grensesetting. Sterke følelsesutbrudd uten en naturlig årsak kan være tegn på en begynnende psykisk lidelse. Er du bekymret for deg selv eller andre kan du ringe TIPS på 51 51 59 59 alle hverdager 08:00-15:00 for råd og veiledning.
SØK HJELP SÅ RASKT SOM MULIG,
DA ER SJANSEN STØRST FOR Å BLI FRISK

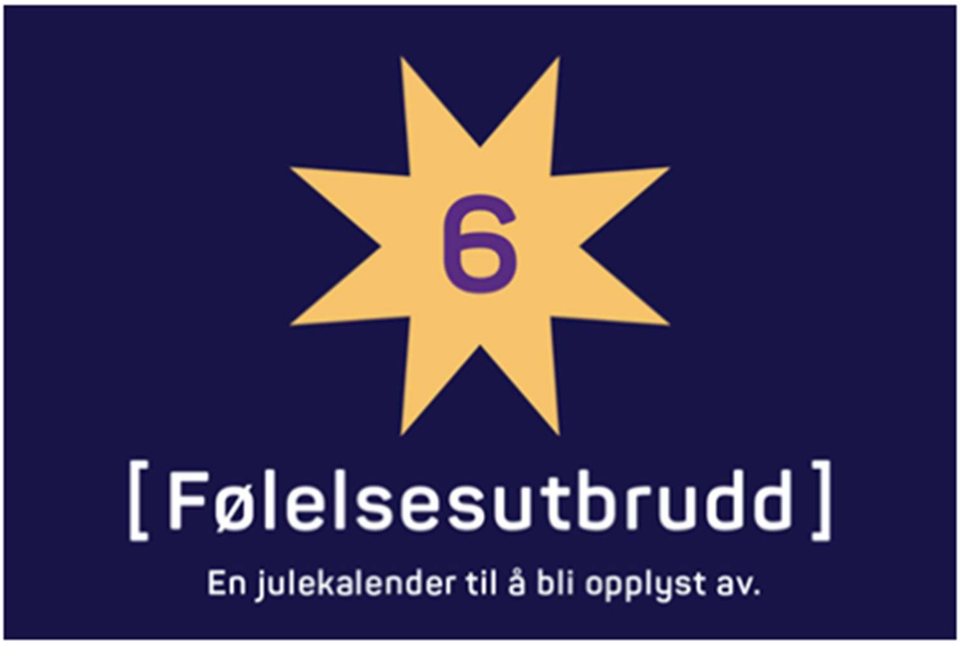

Liker Kommenter Del

TIPS-INFO
5. desember 2016 ·

Humørsvingninger er raske skiftninger i humøret. Lei seg, glad, sint, oppgitt eller opplagt uten helt å vite hvorfor. Det kan ha fysiske eller psykiske årsaker, som for eksempel hormonelle endringer, kjemiske ubalanser, eller alvorlig stress. Det kan også være tegn på en begynnende psykisk lidelse.
Er du bekymret for deg selv eller andre kan du ringe TIPS på 51 51 59 59 alle hverdager 08:00-15:00 for råd og veiledning.
SØK HJELP SÅ RASKT SOM MULIG,
DA ER SJANSEN STØRST FOR Å BLI FRISK

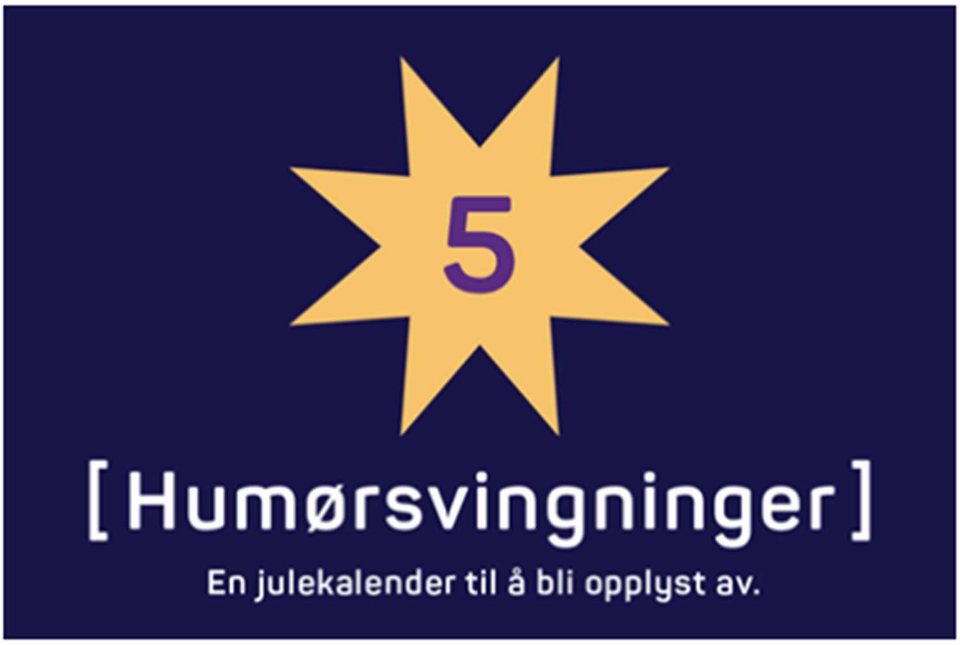

Liker Kommenter Del

TIPS-INFO
4. desember 2016 ·

Tristhet er en nedstemt sinnsstemning, bedrøvet og sørgmodig. Det kan være en naturlig reaksjon på ubehagelige opplevelser eller vanskelige perioder i livet. Tristhet kan også være tegn på en begynnende psykisk lidelse. Er du bekymret for deg selv eller andre kan du ringe TIPS på 51 51 59 59 alle hverdager 08:00-15:00 for råd og veiledning.
SØK HJELP SÅ RASKT SOM MULIG,
DA ER SJANSEN STØRST FOR Å BLI FRISK

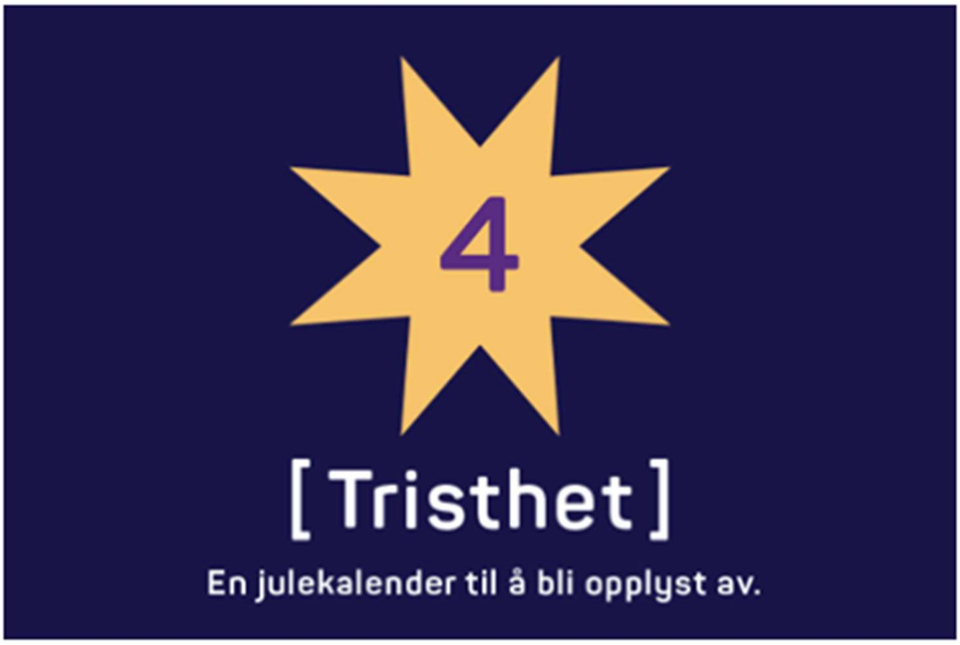

Liker Kommenter Del

TIPS-INFO
3. desember 2016 ·

Angst er et sterkt ubehag uten en åpenbar årsak. Symptomer kan være uro, redsel, hjertebank, pustevansker, konsentrasjonsvansker og tristhet. Angst kan også være tegn på en begynnende psykisk lidelse. Er du bekymret for deg selv eller andre kan du ringe TIPS på 51 51 59 59 alle hverdager 08:00-15:00 for råd og veiledning.
SØK HJELP SÅ RASKT SOM MULIG, DA ER SJANSEN STØRST FOR Å BLI FRISK

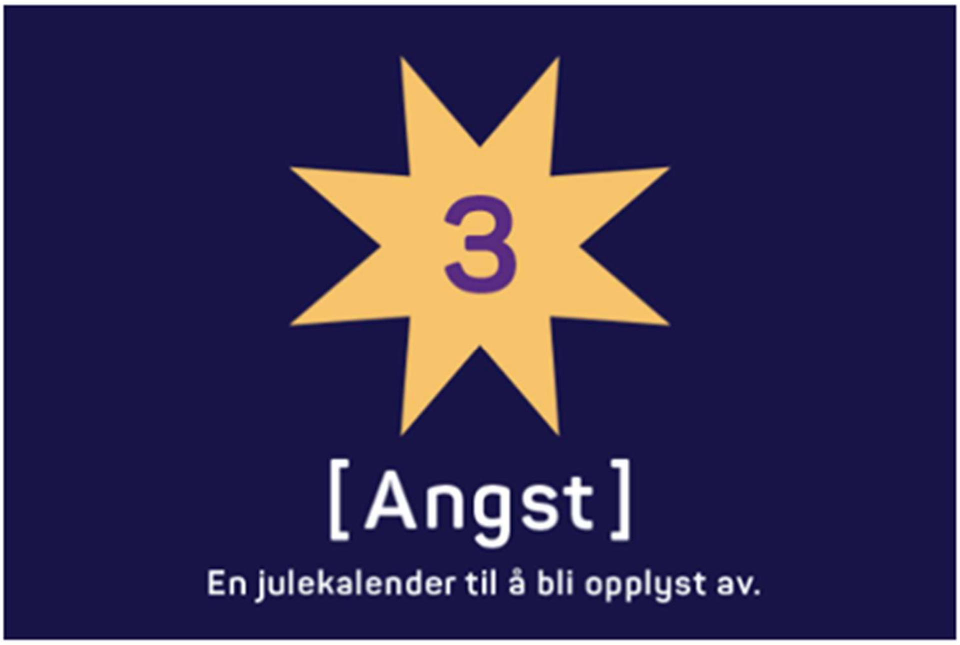

Liker Kommenter Del

TIPS-INFO har oppdatert forsidebildet sitt.
2. desember 2016 ·

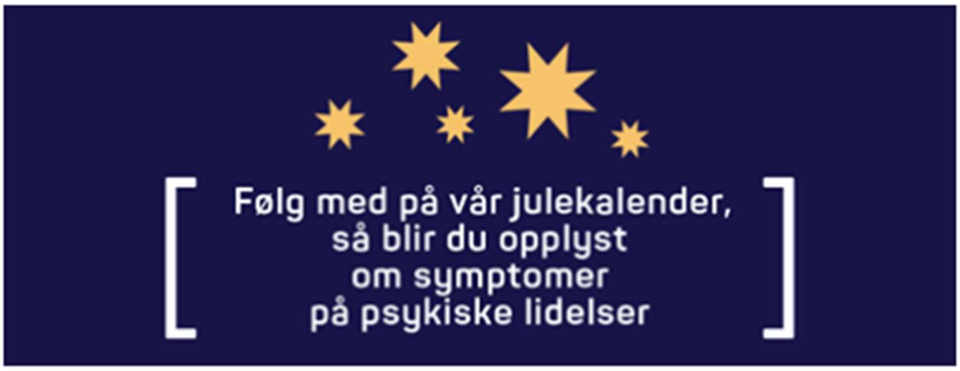

Liker Kommenter Del

TIPS-INFO

2. desember 2016 ·

Dårlig søvn kalles insomni på fagspråket. 10 % av befolkningen har alvorlige og langvarige søvnproblemer. Insomni kan også være tegn på en begynnende psykisk lidelse. Er du bekymret for deg selv eller andre kan du ringe TIPS på 51 51 59 59 alle hverdager 08:00-15:00 for råd og veiledning. SØK HJELP SÅ RASKT SOM MULIG, DA ER SJANSEN STØRST FOR Å BLI FRISK

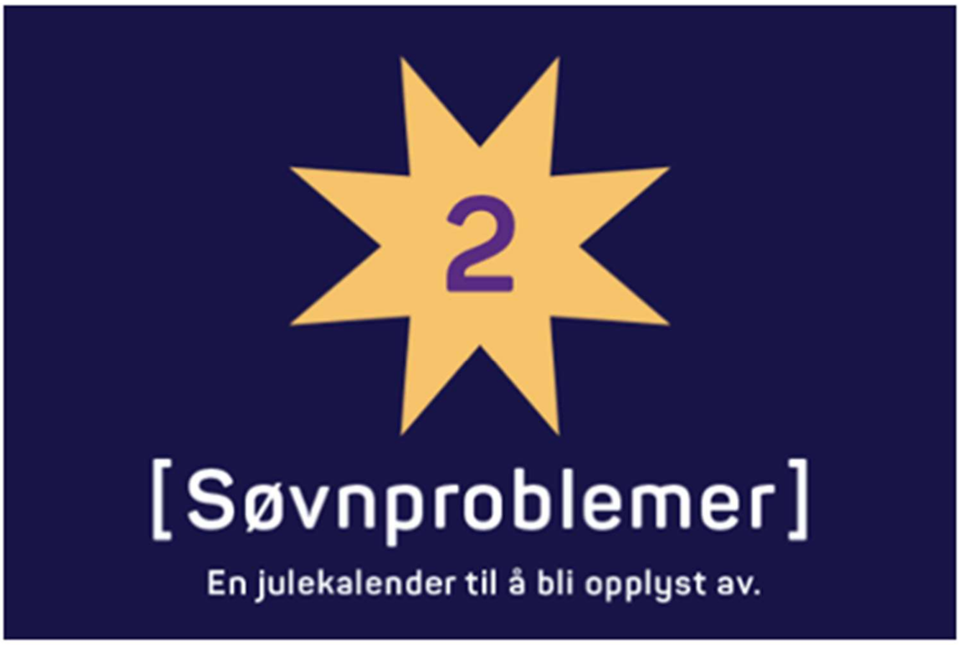

Liker Kommenter Del

TIPS-INFO
1. desember 2016 ·

Sykdomstegn. Kan være av fysisk eller psykisk karakter. Følg med på vår julekalender, så blir du opplyst om symptomer på psykiske lidelser. Er du bekymret for deg selv eller andre kan du ringe TIPS på 51 51 59 59 alle hverdager 08:00-15:00 for råd og veiledning.
SØK HJELP SÅ RASKT SOM MULIG, DA ER SJANSEN STØRST FOR Å BLI FRISK

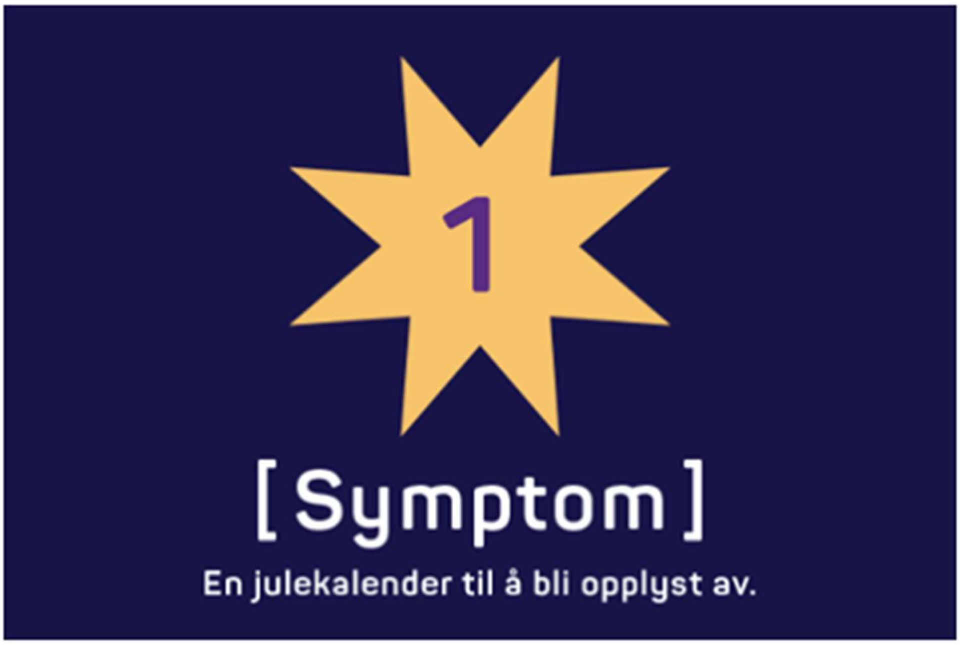

Liker Kommenter Del

TIPS-INFO
1. juli 2016 ·

Nyttige råd

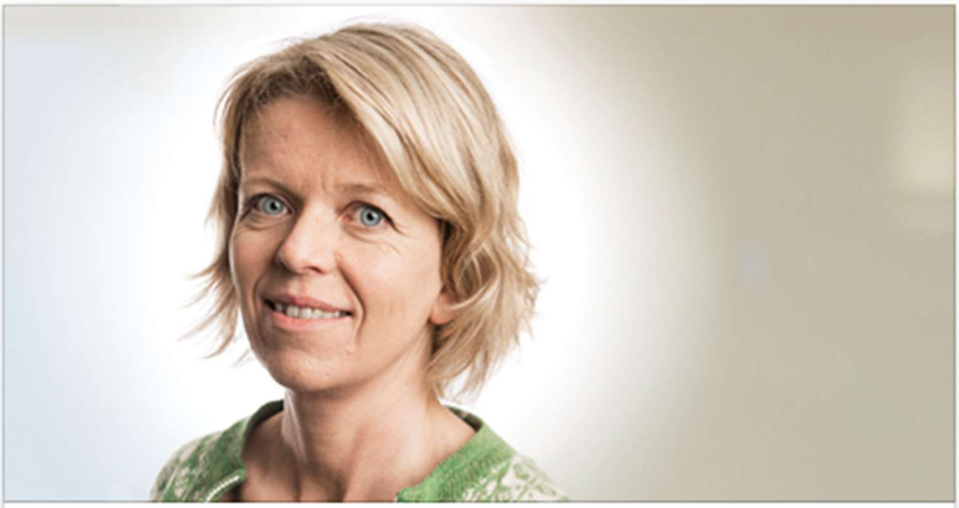

Hvordan snakke med ungdom om hvordan de egentlig har det?

«Ungdommen nå til dags har aldri vært bedre» sto med store bokstaver i Stavanger Aftenblad for en tid tilbake. Av Kristin Hatløy, psykiatrisk sykepleier, TIPS…

BLOGGIVEST.COM

Liker Kommenter Del

TIPS-INFO har oppdatert forsidebildet sitt.
18. desember 2015 ·

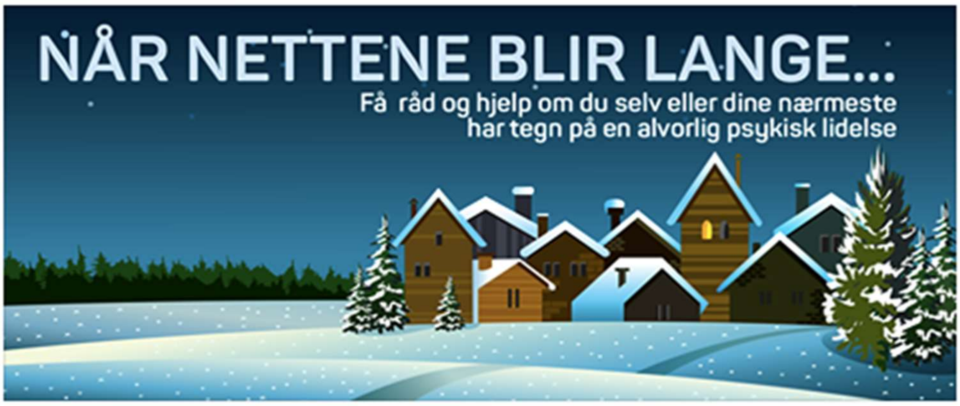

Liker Kommenter Del

TIPS-INFO
13. august 2015 ·

Den Schizofrene Ophelia?

http://www.tips-info.com/forsvinner-schizofreni/

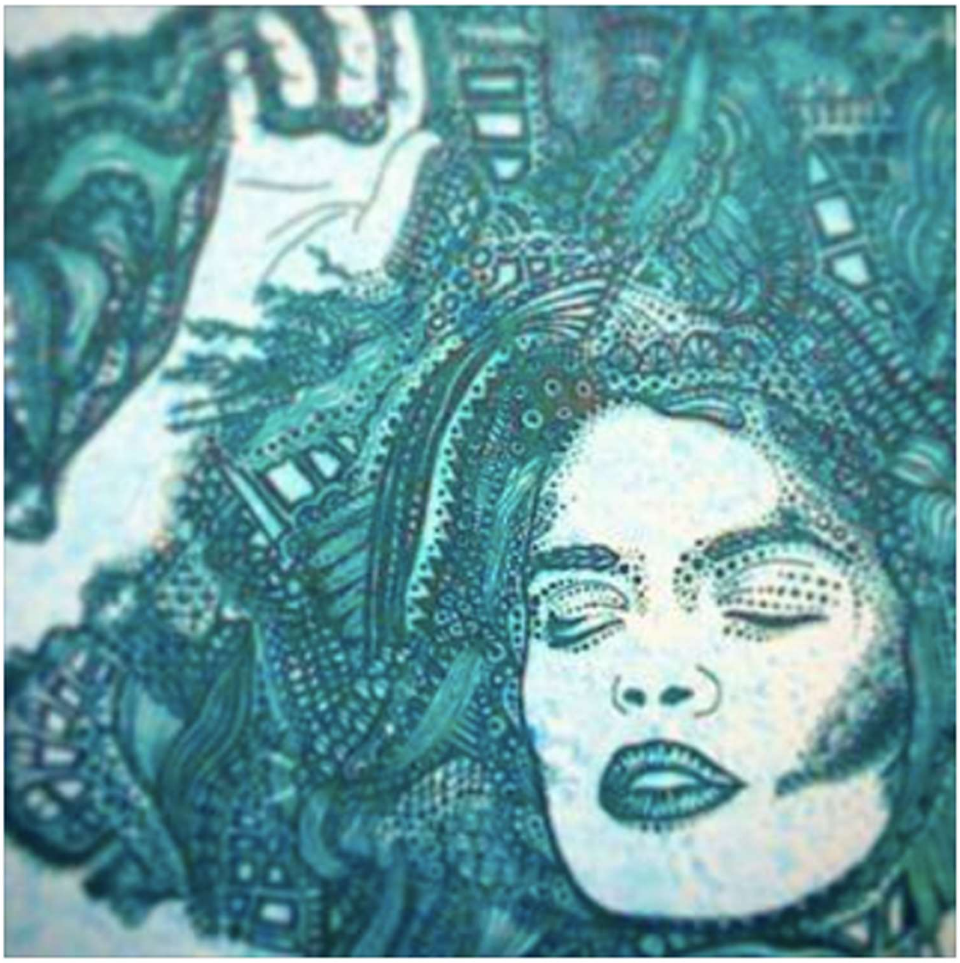

Liker Kommenter Del

TIPS-INFO har delt en lenke.
12. august 2015 ·

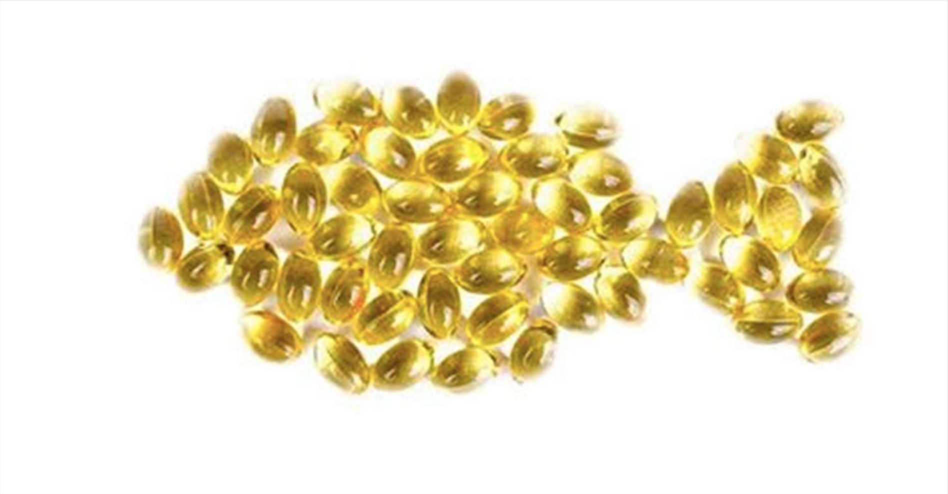

Ingen grunn til å slutte å ta Omega 3! | TIPS

Vi bruker cookies på Tips-info.com for å gi deg den beste onlineopplevelsen. Ved å bruke vårt nettsted samtykker du til vår bruk av cookies i samsvar med vår cookiespolicy. Close

TIPS-INFO.COM

Liker Kommenter Del

TIPS-INFO har oppdatert forsidebildet sitt.
6. juli 2015 ·

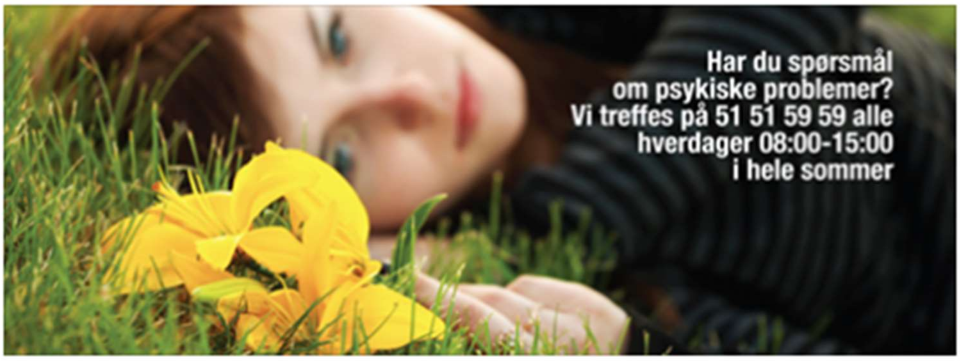

Liker Kommenter Del

TIPS-INFO har delt en lenke.

1. juni 2015 ·

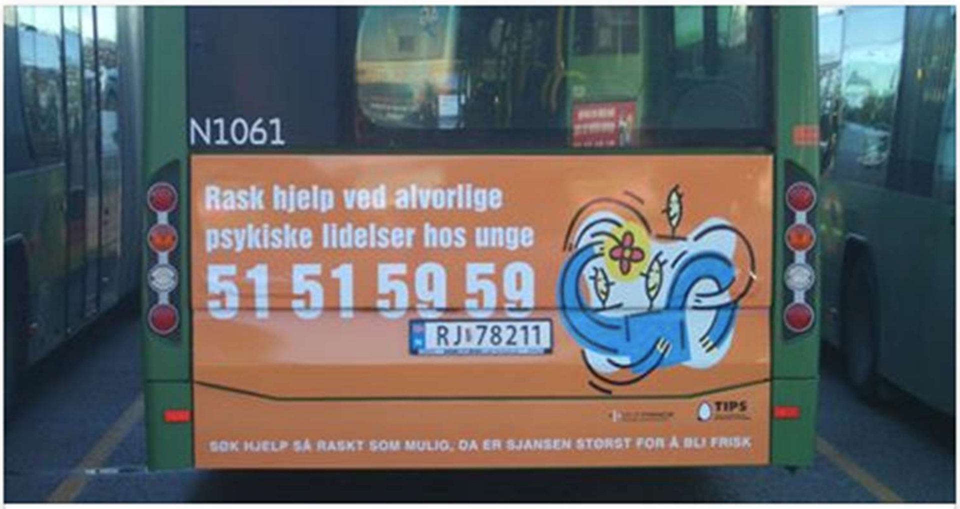

TIPS i nye lokaler | TIPS

TIPS har levd en omflakkende tilværelse siden prosjektet startet for fullt i 1997. Fra høyhuset til studentrommet har det tidvis vært trangt. Siden 2002 fikk vi noe mer permanente kontorer i AMC2 sine lokaler.

TIPS-INFO.COM

TIPS-INFO har oppdatert forsidebildet sitt.

27. februar 2015 ·

For mail, telefon eller chat følg denne tråden http://hjelp.tips-info.com/

Se oversettelse

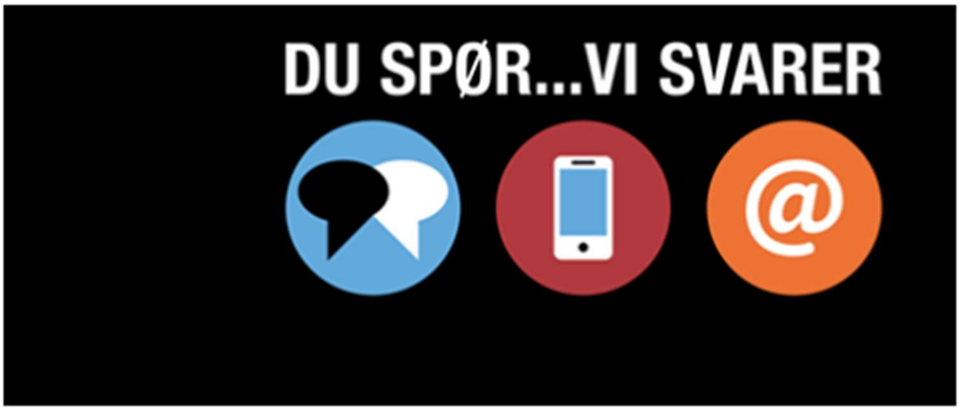

Liker Kommenter Del

TIPS-INFO har oppdatert forsidebildet sitt.

10. desember 2014 ·

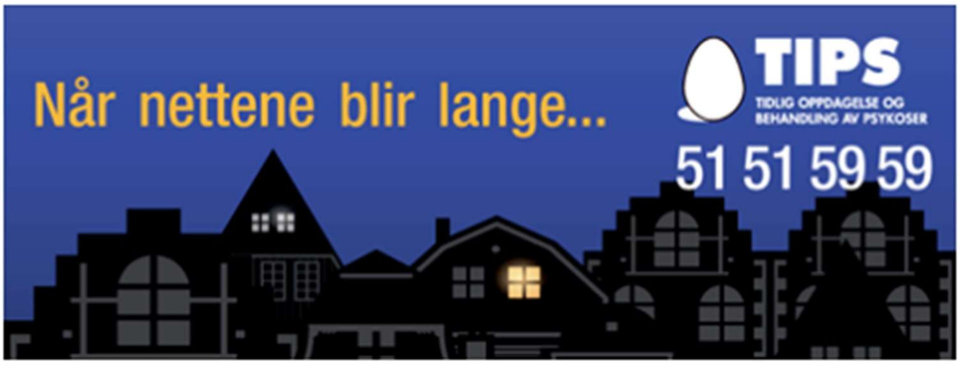

Liker Kommenter Del

TIPS-INFO har lagt til et nytt bilde.
15. oktober 2014 ·

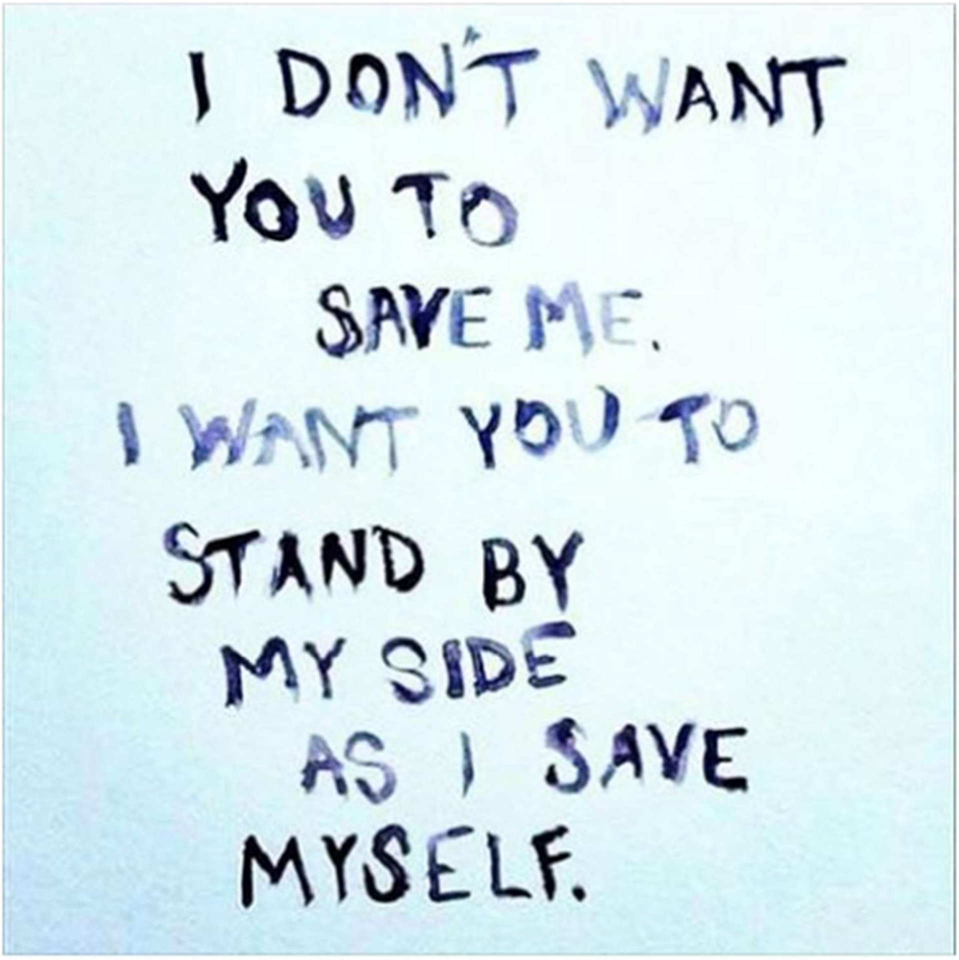

Liker Kommenter Del

TIPS-INFO
9. september 2014 ·

Ta en titt på- Trenger du hjelp ?- på våre hjemmesider. Inneholder ulike type informasjon om ulike tilbud, samt gir en oversikt over andre lav terskel utredningsteam rundt i Norge.

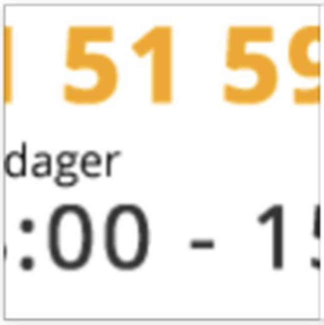

Trenger du hjelp? | TIPS
SKOLEN Lærer, rådgiver og helsesøster kan hjelpe deg hvis du trenger råd for deg selv eller andre. Rådgiveren har et særskilt ansvar for slike saker på skolen.
TIPS-INFO.COM

Liker Kommenter Del

TIPS-INFO har oppdatert forsidebildet sitt.
5. mai 2014 ·

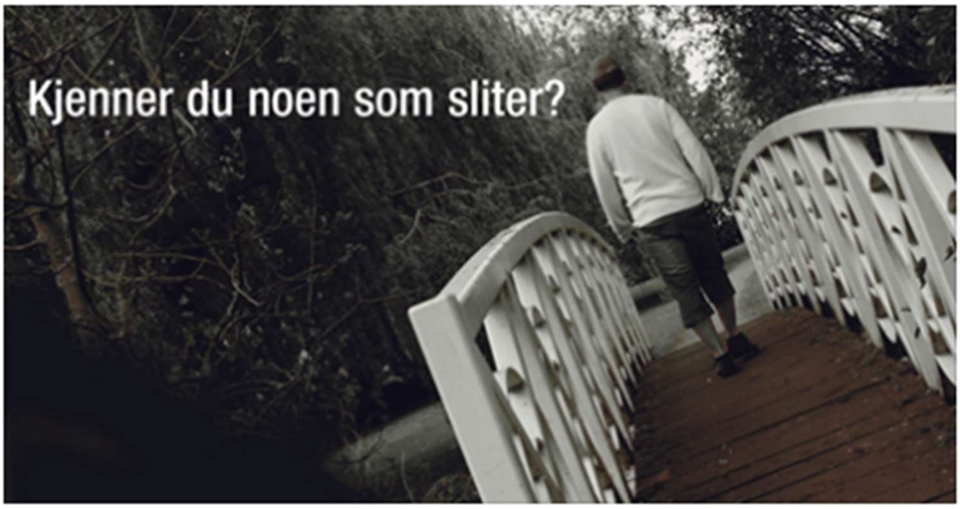

Liker Kommenter Del

TIPS-INFO har oppdatert forsidebildet sitt.
8. januar 2014 ·

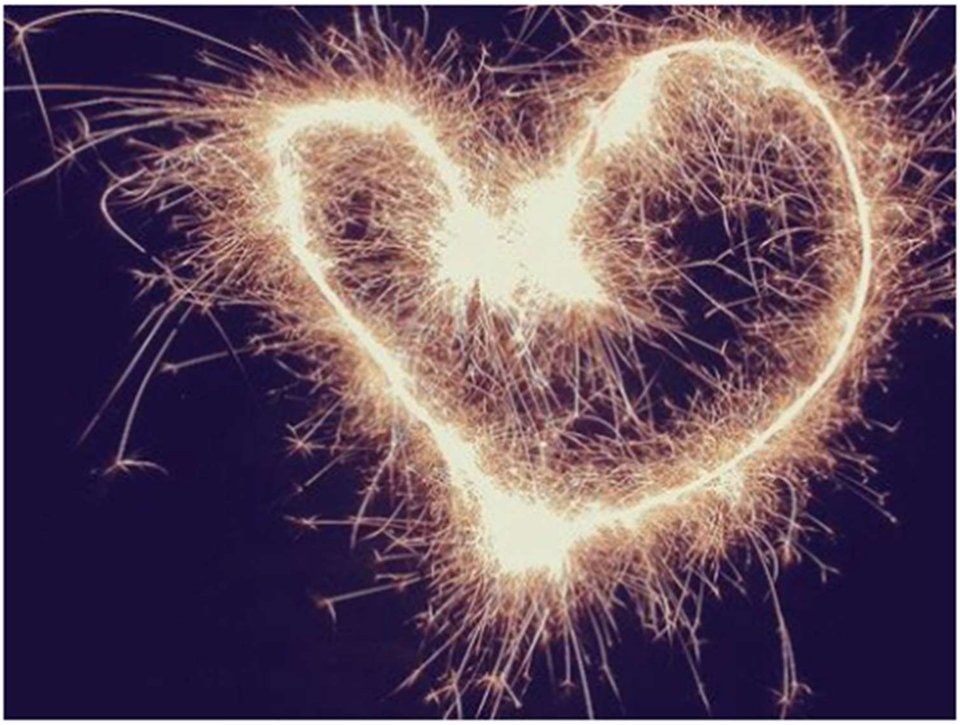

Liker   Kommenter   Del

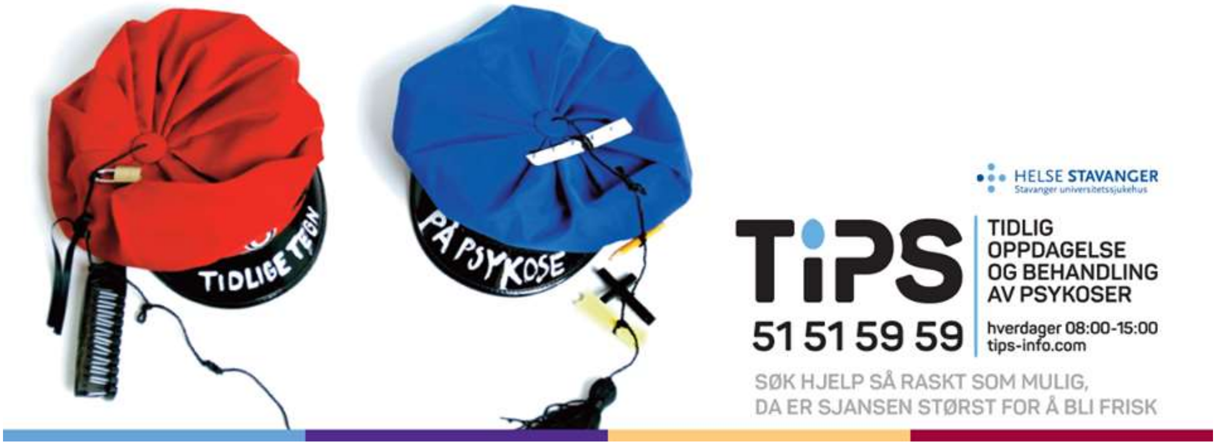

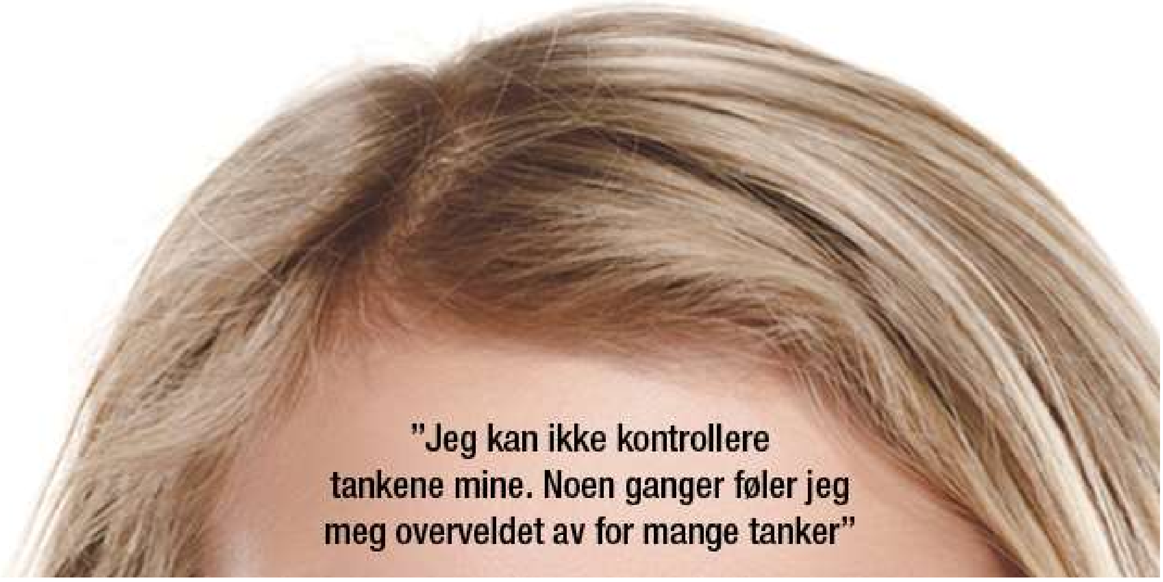
”Jeg kan ikke kontrollere
tankene mine. Noen ganger føler jeg
meg overveldet av for mange tanker”

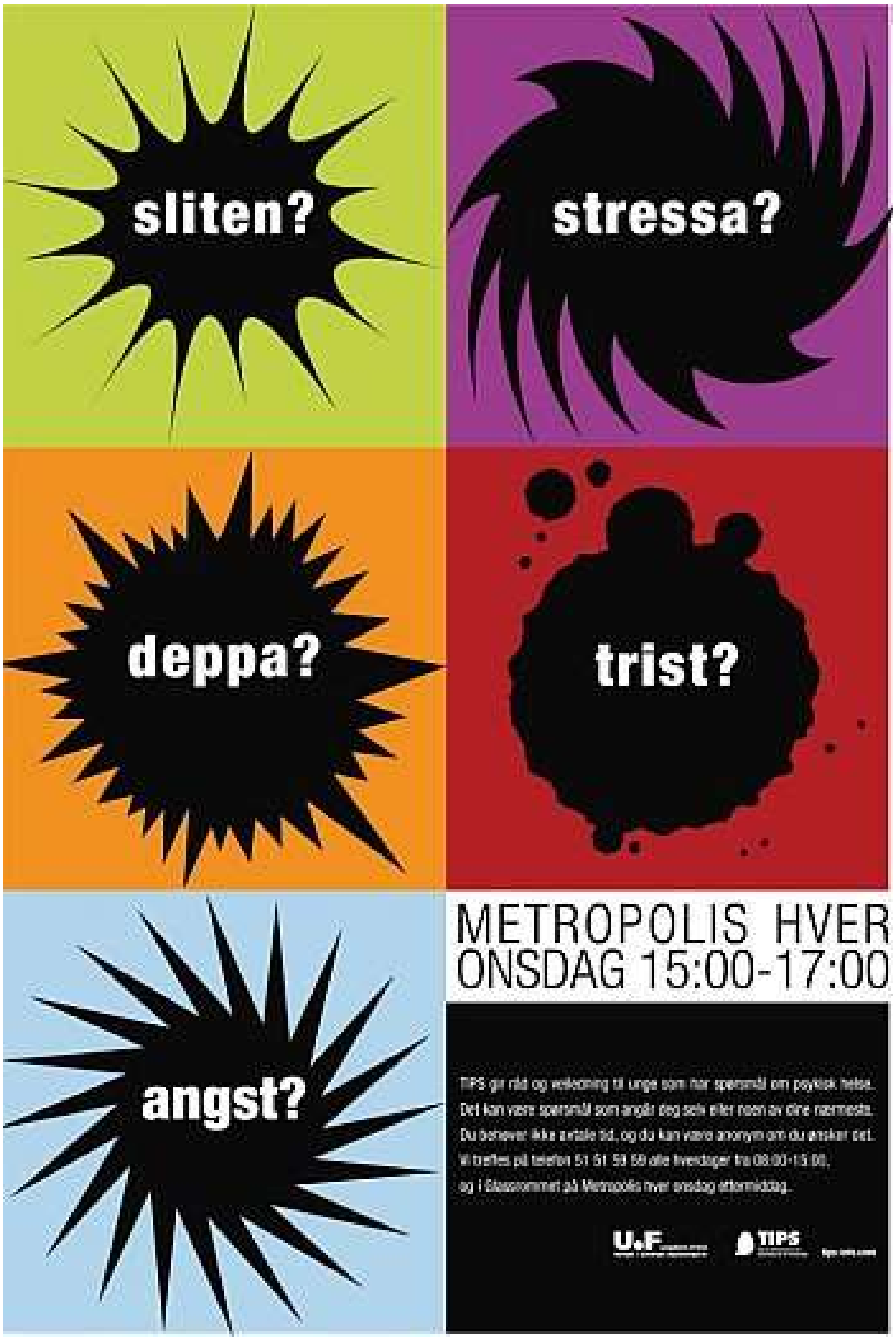
sliten?
stressa?
deppa?
trist?
angst?
METROPOLIS HVER
ONSDAG 15:00-17:00
TIPS gir råd og veiledning til unge som har spørsmål om psykisk helse.
Det kan være spørsmål som angår deg selv eller noen av dine nærmeste.
Du behøver ikke avtale tid, og du kan være anonym om du ønsker det.
Vi treffes på telefon 51 51 59 59 alle hverdager fra 08.00-15.00,
og i Glassrommet på Metropolis hver onsdag ettermiddag.
TIPS

TIPS

TIDLIG OPPDAGELSE OG
BEHANDLING AV PSYKOSER

51 51 59 59

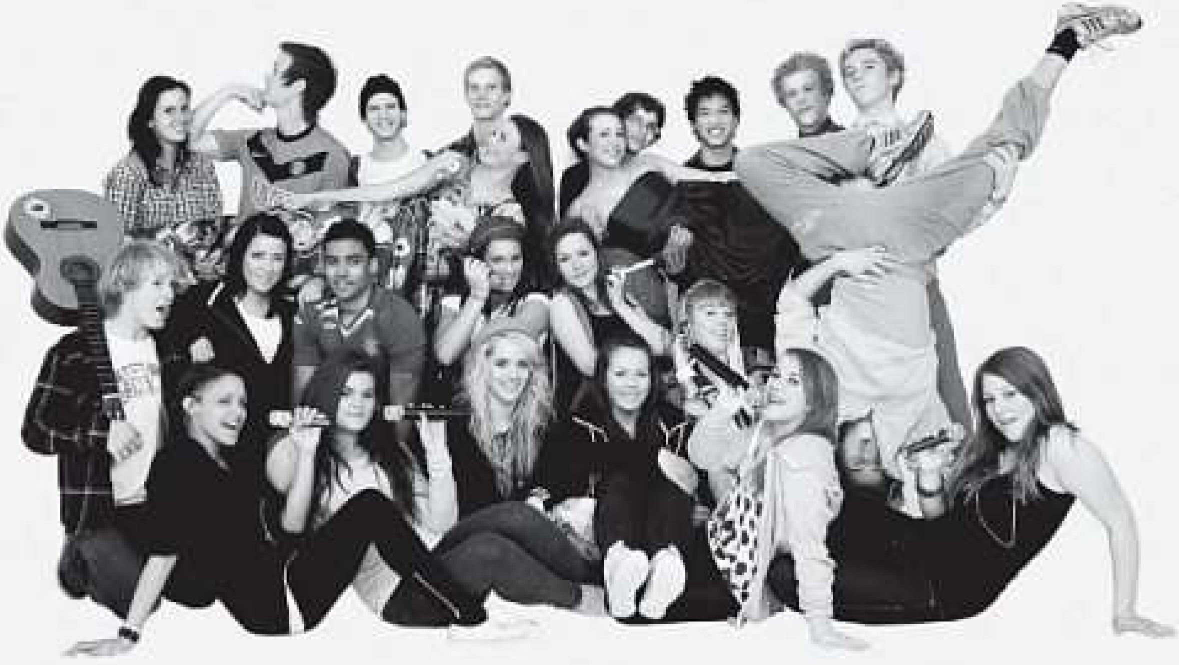

Ungdommen nå til dags!

I morgen kan du se en annen side av årets russ. Vi skal vise at vi kan mer enn å feste. Vi fyller Tjodhallen med et forrykende show og en flott russefest med kunst og underholdning på programmet. Inntekten fra dette går til UNICEF som bruker pengene til å bygge skoler for barn i det sørlige Afrika. I løpet av russetiden skal vi samle inn mye penger til dette prosjektet!

Russefeiringen skal være et minne for livet -et godt minne. Og for de fleste blir det akkurat det. Visst kan det gå litt vilt for seg innimellom - men vi passer på hverandre så godt vi kan. April og mai er russetid, men det er også innspurten til eksamen, så det er dager med press og stress. Det kan bli mye på en gang.

For enkelte kan det bli for mye. Noen sliter med psykiske problemer. Stort sett forbigående, men for noen få kan det være alvorlig. Kombinasjonen av stort press, festing og lite søvn kan utløse alvorlige tilstander. Hvis du sliter, eller ser andre som gjør det, er det hjelp å få. Ring TIPS for råd og hjelp, for deg selv eller andre. Du kan være anonym om du ønsker det.

Søk hjelp så raskt som mulig, da er sjansen størst for å bli frisk

KULTURRUSS

TIRSDAG 16. MARS, KL. 11.00, 13.30 OG 17.00, TJODHALLEN UIS. KR 30 (ELEV/STUDENT), KR 50 (VOKSNE).

TIDLIGE TEGN PÅ PSYKISKE PROBLEMER

- Isolerer seg
- Sover dårlig
- Angst
- Tristhet
- Konsentrasjonsvansker
- Forsømmer husarbeid, hygiene, jobb eller skole
- Ekstremt opptatt av temaer som døden, politikk eller religion
- Store humørsvingninger
- Tankene høres ut som stemmer
- Snakker usammenhengende
- Føler seg forfulgt eller styrt av andre

TIPS

51 51 59 59

hverdager [illegible]

www.tips-info.com

TIPS-INFO
16. desember 2009 ·

Skolekampanjen 2009

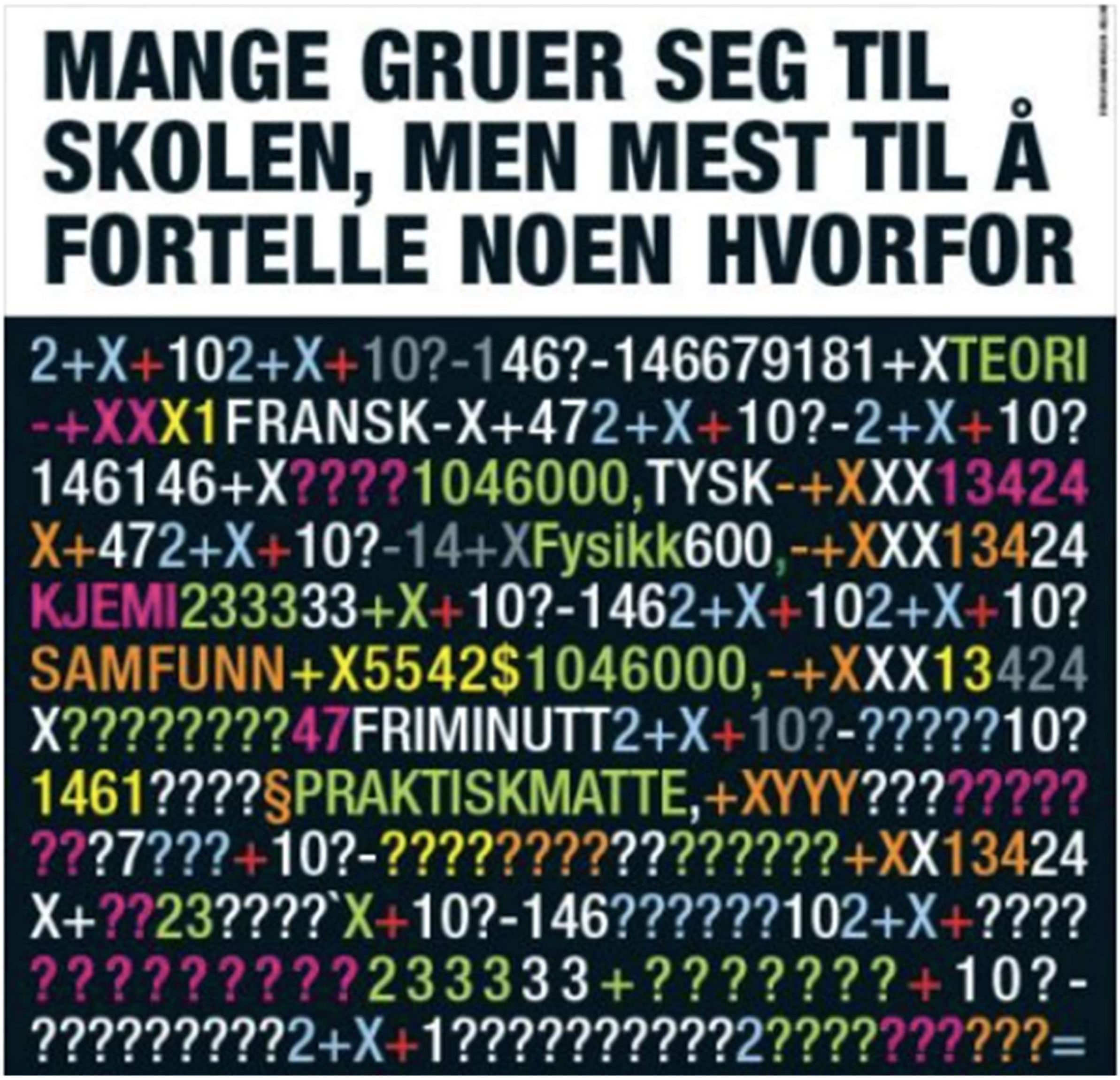

TIPS-INFO har lagt til et nytt bilde.
29. desember 2009 ·

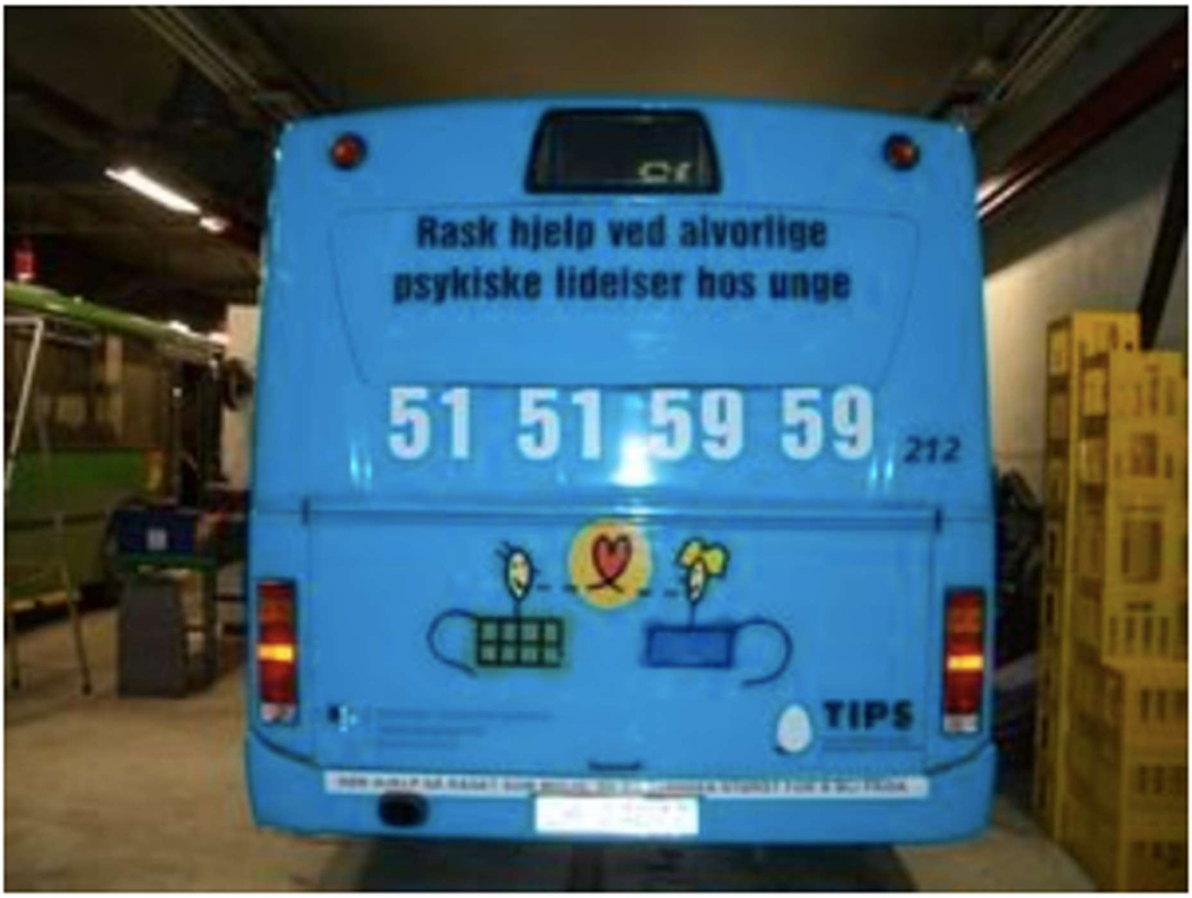

6 1 kommentar

Liker Kommenter Del

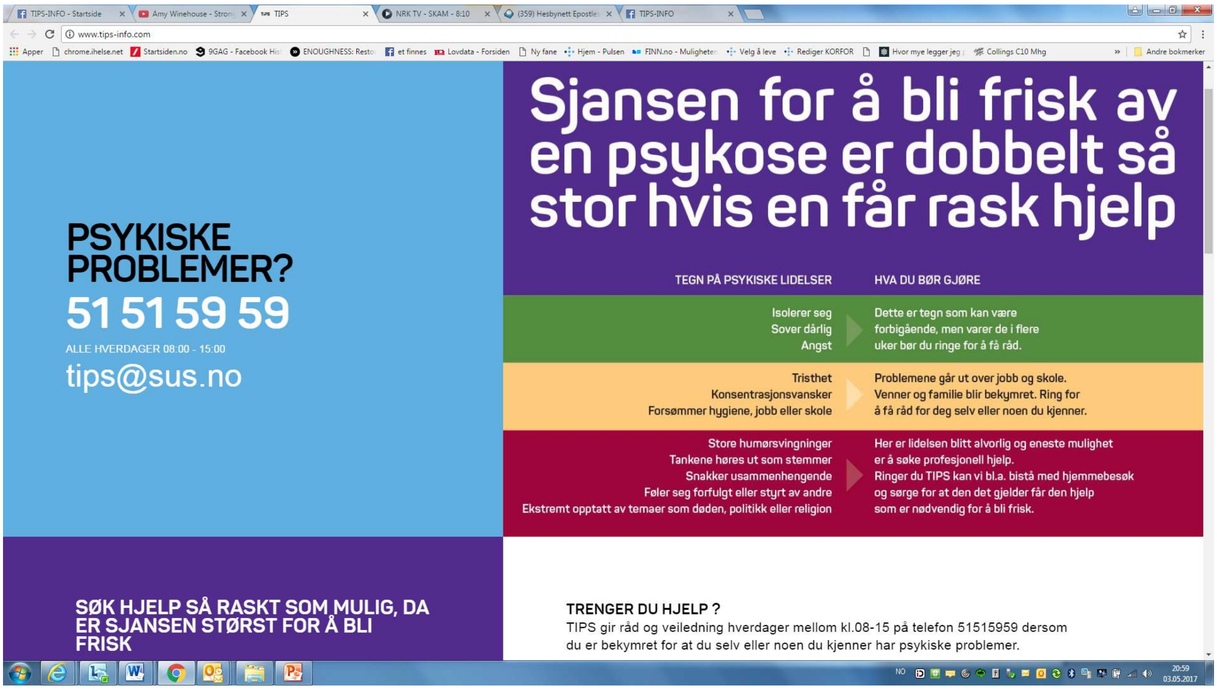

(fra ppt TIPS)

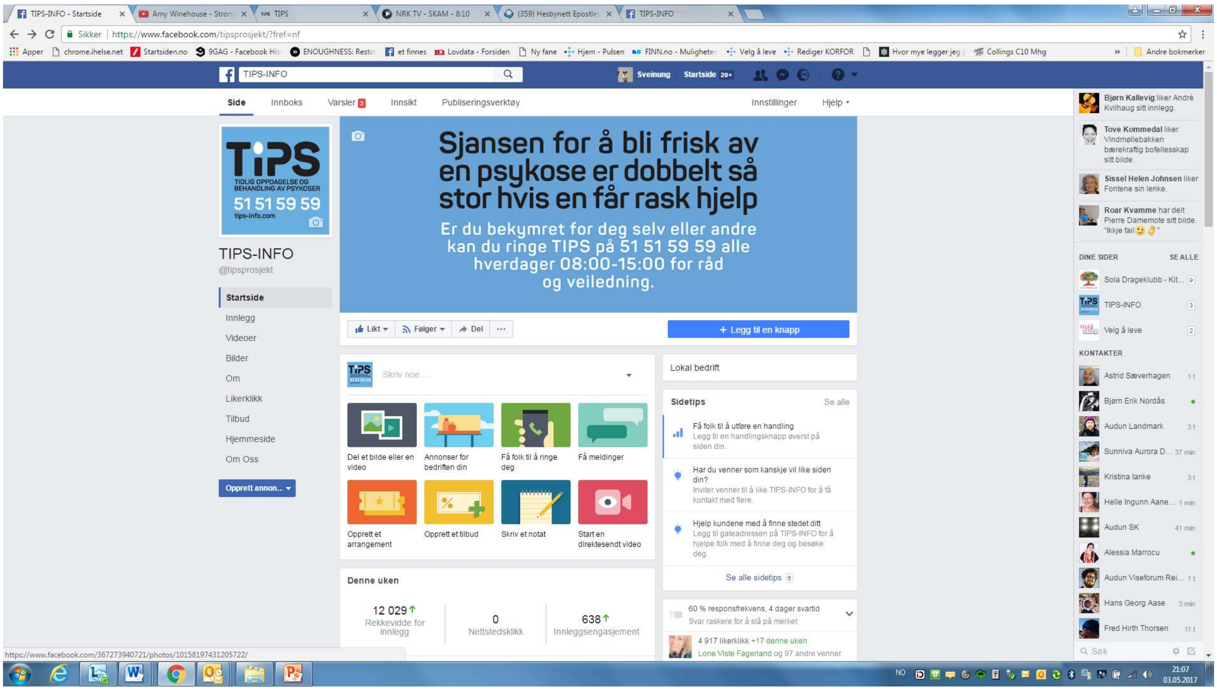

(fra ppt TIPS)

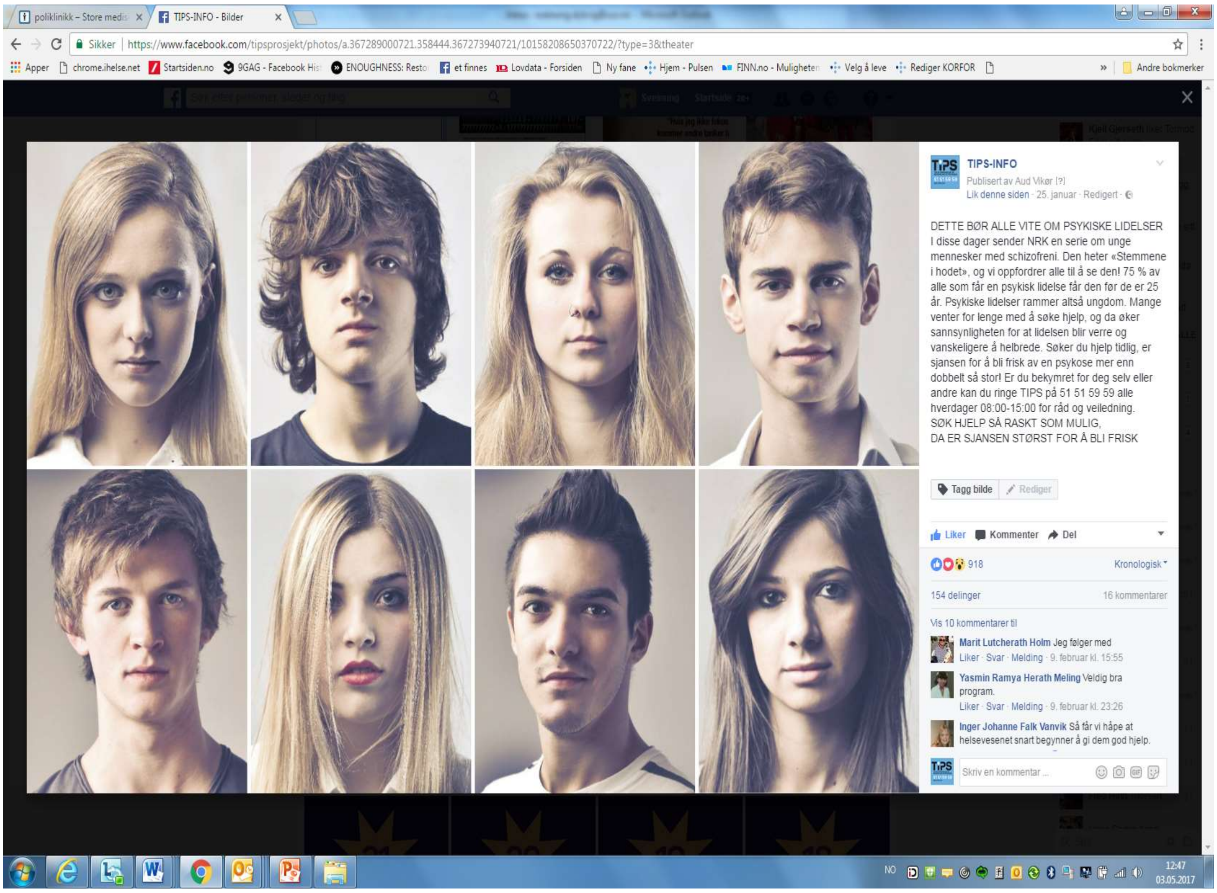

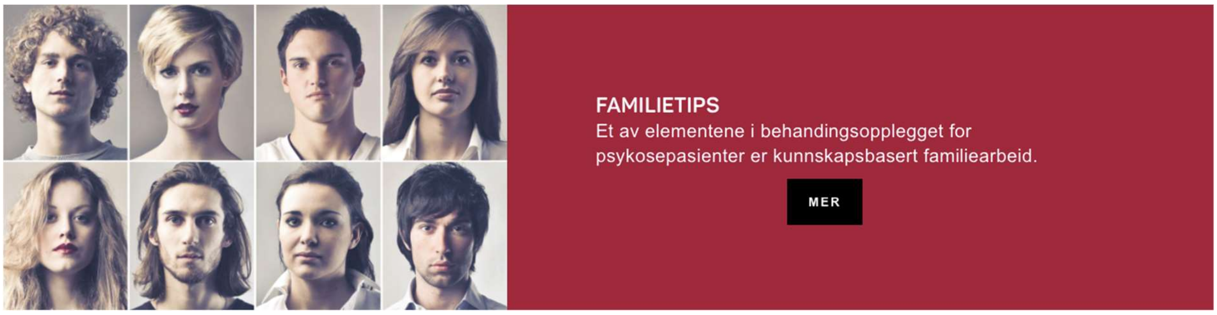

(fra hjemmesiden)

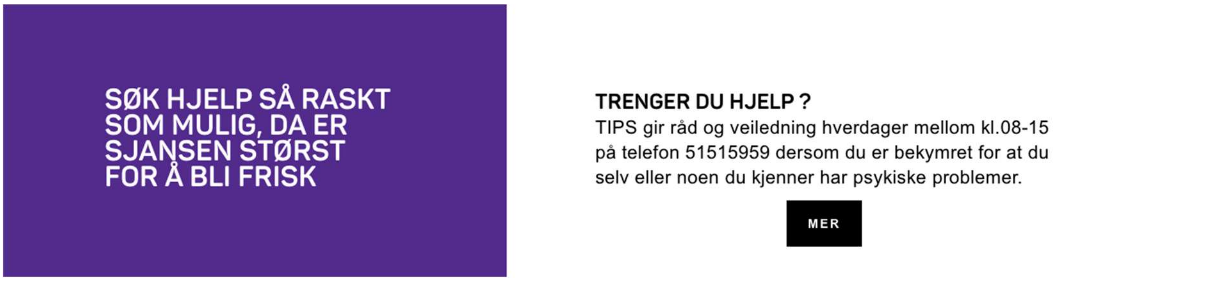

(fra hjemmesiden)

Usikker på dette, inkludere?

https://elaering-psykose.psykopp.no (merket TIPS? Ikke i utgangspunktet, men på slutten står det at det er et samarbeidsprosjekt mellom TIPS og Helsedirektoratet) lenke på facebook + hjemmeside
